# Photoredox-Catalyzed Site-Selective Intermolecular C(sp^3^)–H Alkylation of Tetrahydrofurfuryl Alcohol Derivatives

**DOI:** 10.1021/acs.orglett.4c04439

**Published:** 2025-01-14

**Authors:** Reiji Abe, Kazunori Nagao, Tomohiro Seki, Dai Hata, Yusuke Sasaki, Hirohisa Ohmiya

**Affiliations:** † Institute for Chemical Research, Kyoto University, Uji, Kyoto 611-0011, Japan; ‡ Research, Takeda Pharmaceutical Company Limited, Fujisawa, Kanagawa 251-8555, Japan

## Abstract

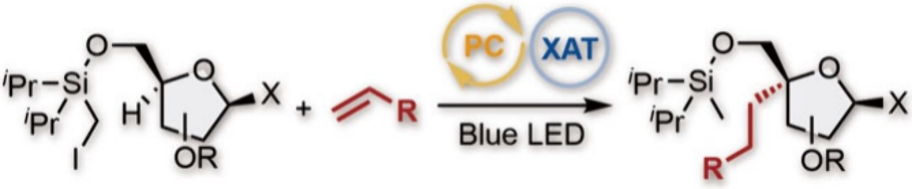

4′-Selective alkylation of nucleosides has been recognized as one of the ideal and straightforward approaches to chemically modified nucleosides, but such a transformation has been scarce and less explored. In this Letter, we combine a visible-light-mediated photoredox catalysis and hydrogen atom transfer (HAT) auxiliary to achieve β-C(sp^3^)–H alkylation of alcohol on tetrahydrofurfuryl alcohol scaffolds and exploit it for 4′-selective alkylation of nucleosides. The reaction involves an intramolecular 1,5-HAT process and stereocontrolled Giese addition of the resultant radicals.

Over the past several decades, chemically modified nucleosides have gained much attention in drug discovery. Specifically, 4′-carbon-substituted nucleosides are one of the important classes of them and are found in nucleoside analogue reverse transcriptase inhibitors (NRTIs)^1^ and oligonucleotide therapeutics^2^ (Scheme 1A, left). However, synthetic approaches to such nucleosides have conventionally relied on ionic functionalization of the 4′-position of nucleosides. For example, in the chemical synthesis of Islatravir, one of representative NRTI for HIV treatment, ethynyl group at 4′-position of a nucleoside scaffold is introduced via alkynylation of dihydroxyacetone or ketones derived from 2-deoxy-D-ribose.^3^ The synthesis of locked nucleic acid (LNA) involves aldol reaction of the aldehyde derived from nucleosides with formaldehyde.^4^ However, these synthetic routes require appropriate oxidation and reduction steps to introduce carbon fragments in an ionic manner, resulting in preactivation of natural nucleosides or preparation from non-nucleoside substrates.

**Scheme 1:**
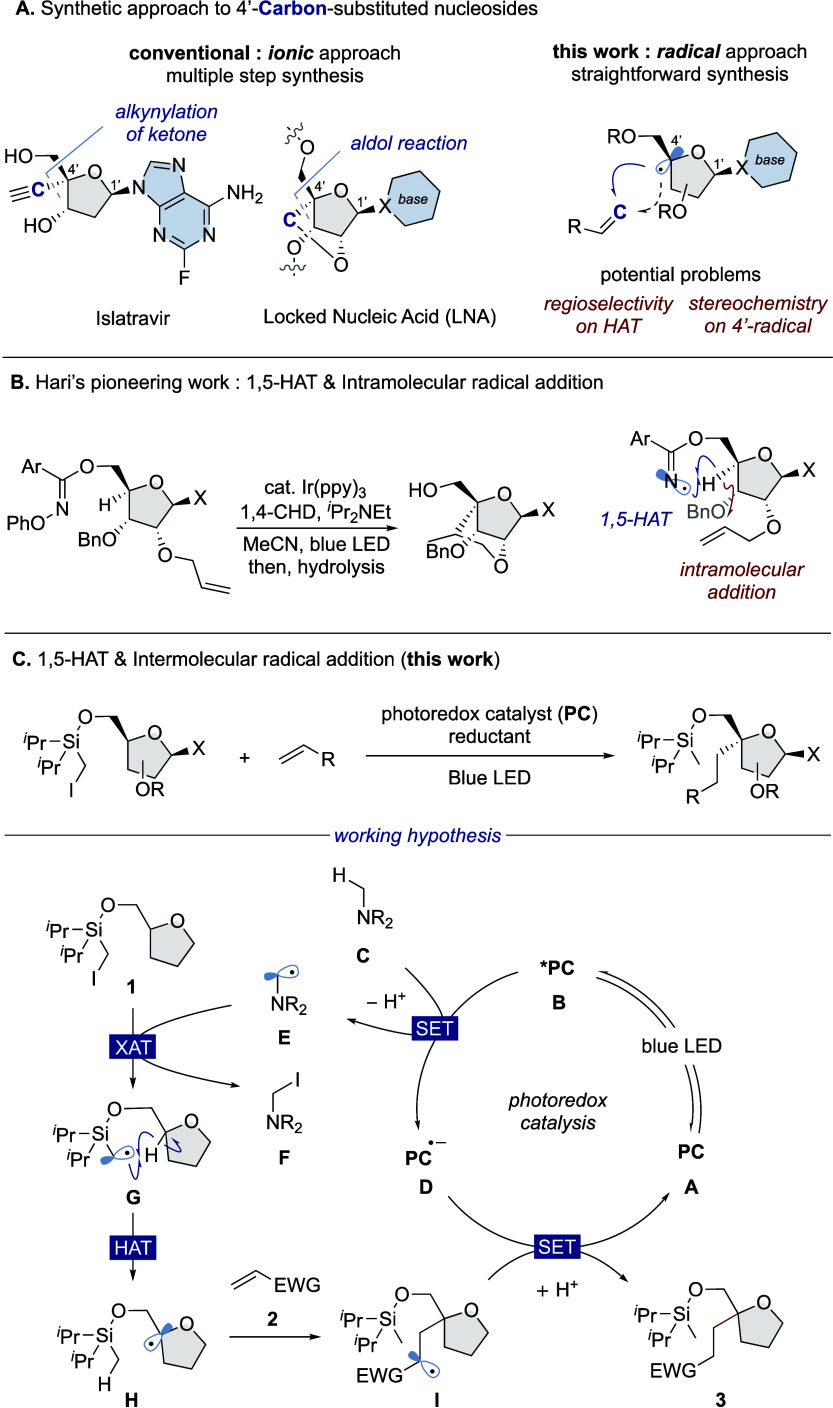
Synthetic Approaches to 4′-Carbon-Substituted Nucleosides

Recently, hydrogen atom transfer (HAT) from organic molecules has emerged as a powerful and straightforward process to generate a carbon-centered radical on aliphatic chains that can be exploited for diverse functionalizations.^5^ Therefore, radical functionalization of natural nucleosides through the 4′-selective HAT process has become an efficient way to access 4′-carbon-substituted nucleosides (Scheme 1A, right).^6^ However, this approach has two intrinsic problems, with respect to the regioselectivity of HAT and the stereochemistry of radical functionalization. Since furanose sugar scaffolds contain several weak C(sp^3^)–H bonds connected to the hydroxy group, regioselective HAT at the 4′-position remains an unavoidable problem. Additionally, we anticipated that the stereochemistry of the 4′-position would be lost after the HAT process and that the stereochemical course of the radical reaction would be also perceived as another problem with this strategy. Pioneeringly, Hari and co-workers demonstrated catalytic generation of a 4′-carbon-centered radical on nucleoside scaffolds through intramolecular 1,5-HAT by an iminyl radical generated on a 5′-*O*-substituent (Scheme 1B).^7^ The generated 4′-carbon radical was added to an alkene group at the 2′- or 3′-position to afford the corresponding bridged nucleosides. Although this report demonstrated that positioning of an appropriate HAT auxiliary on furanose sugar scaffolds leads to 4′-selective radical generation and intramolecular functionalization, an intermolecular radical functionalization was not explored. Therein, we commenced our investigation of intermolecular C4′-selective alkylation of nucleosides.

According to Hari’s report, we explored a photoredox-catalyzed Giese addition of *O*-imidoyl-substituted tetrahydrofurfuryl alcohol and ethyl acrylate (see Supporting Information). However, the reaction did not produce the desired product at all. Therefore, we aimed to identify a suitable HAT auxiliary that is easily installed and removed. After our literature survey, an iodomethylsilyl group was found to be a suitable HAT auxiliary, which undergoes 1,5-HAT in the tetrahydrofurfuryl alcohol moiety.^8^ Based on this finding, we created our working hypothesis on site-selective intermolecular C(sp^3^)–H alkylation of tetrahydrofurfuryl alcohol derivatives utilizing a visible-light-mediated photoredox catalysis and iodomethylsilyl-type HAT auxiliary (Scheme 1C). First, blue light irradiation converts a ground state (**A**) of the photoredox catalyst to the excited state (**B**), which can oxidize an alkylamine reductant (**C**) to the corresponding radical cation. The subsequent deprotonation of the radical cation occurs to afford an α-aminoalkyl radical (**E**),^9^ which acts as a halogen atom transfer (XAT)^10^ reagent and abstracts an iodine atom from the iodomethylsilyl group of tetrahydrofurfuryl alcohol substrate **1**. The resultant α-silylmethyl radical (**G**) undergoes 1,5-HAT to form a *tertiary* alkyl radical (**H**). The radical addition of **H** to an electron-deficient alkene **2** generates a prolonged radical (**I**), which can be reduced by a radical anion form of a photoredox catalyst (**D**) and then protonated to produce **3**.

According to our working hypothesis, we commenced our investigation of intermolecular C(sp^3^)–H alkylation of *O*-(iodomethyl)diisopropylsilyl-substituted tetrahydrofurfuryl alcohol **1a** with ethyl acrylate **2a** under visible-light irradiation conditions. Then, we found that the desired reaction proceeded in the presence of Ir[dF(CF_3_)ppy]_2_(dtbbpy)PF_6_ (2 mol %) and pentamethylpiperidine (PMP) in a mixed solvent system of acetonitrile (MeCN) and water under blue LED irradiation to afford C(sp^3^)–H alkylation product **3aa** with an alkylated product on HAT auxiliary group **3aa-1** and dehalogenative protonation product **1a-1** (Table 1, entry 1).

**Table 1:**
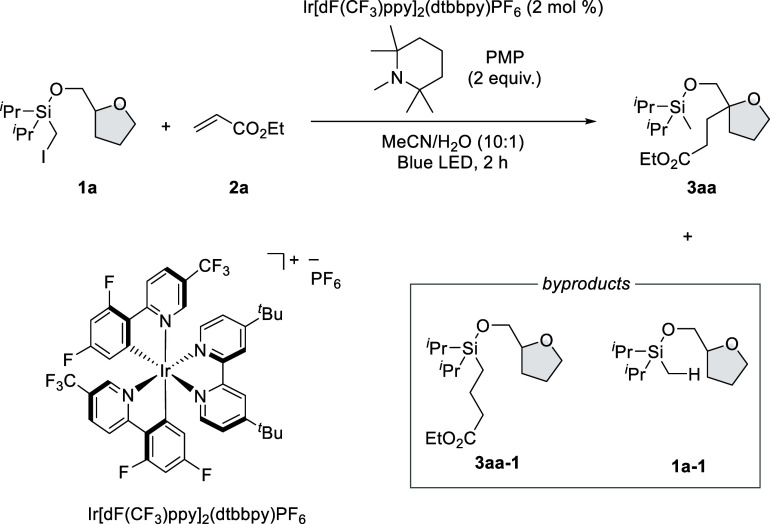
Screening of Reaction Conditions on C(sp^3^)–H Alkylation of **1a**

Entry	Change from standard conditions	Yield of **3aa** (%)^b^	Yield of **3aa-1** (%)^b^	Yield of **1a-1** (%)^b^
1	none	68 (67)^c^	15	9
2	4CzIPN instead of Ir[dF(CF_3_)ppy]_2_(dtbbpy)PF_6_	44	6	16
3	Ir(ppy)_2_(dtbbpy)PF_6_ instead of Ir[dF(CF_3_)ppy]_2_(dtbbpy)PF_6_	66	13	14
4	Ir(ppy)_3_ instead of Ir[dF(CF_3_)ppy]_2_(dtbbpy)PF_6_	22	0	16
5	Et_3_N instead of PMP	63	12	18
6	Et^*i*^Pr_2_N instead of PMP	65	11	17
7	Bn_3_N instead of PMP	0	0	0
8	DMSO instead of MeCN	42	10	11
9	DMF instead of MeCN	37	8	4
10	acetone instead of MeCN	51	9	15
11	THF instead of MeCN	30	5	41
12	toluene instead of MeCN	45	7	24
13	only MeCN (0.09 M)	24	0	22
14	MeCN/H_2_O (10:1, 0.1 M) 10-fold diluted	27	30	8
15	without light	0	0	0
16	without Ir[dF(CF_3_)ppy]_2_(dtbbpy)PF_6_	0	0	0
17	without PMP	0	0	0

a Reaction was carried out with **1a** (0.1 mmol), **2a** (0.15 mmol), photoredox catalyst (0.002 mmol), and PMP (0.2 mmol) in solvent (10 mL) and water (1 mL) under blue LED irradiation for 2 h.

b ^1^H NMR yield based on **1a**.

c Isolated yield.

The effects of each reaction component were summarized in Table 1. When other photoredox catalysts, such as 4CzIPN^11^ and Ir(ppy)_2_(dtbbpy)PF_6_, were tested, **3aa** was obtained in moderate and comparable yields. 4CzIPN (*E*_red_* = 1.35 V vs SCE)^11^ and Ir(ppy)_2_(dtbbpy)PF_6_ (*E*_red_* = 0.92 V vs SCE)^12^ are sufficient for the single electron oxidation of PMP (*E*_ox_ = 0.78 V vs SCE).^13^ On the other hand, the reaction with Ir(ppy)_3_ resulted in low conversion due to the insufficient oxidation ability of Ir(ppy)_3_ (*E*_red_* = 0.31 V vs SCE)^14^ (entry 4). Although triethylamine (*E*_ox_ = 0.77 V vs SCE)^15^ and diisopropylethylamine (*E*_ox_ = 0.67 V vs SCE)^15^ exhibited comparable reactivities with PMP as an amine reductant, tribenzylamine (*E*_ox_ = 1.18 V vs SCE)^15^ did not work at all (entries 5–7). The effects of the reaction solvents were also evaluated. Other solvents, such as DMSO, DMF, acetones, THF, and toluene, were found to be effective (entries 8–12). However, a significant amount of byproduct **1a-1** was obtained with the use of THF as solvent. It might be due to the competing intermolecular HAT reaction between **G** and THF. Water was necessary to promote the reaction in high efficiency (entry 13). When the reaction was conducted under concentrated conditions, the yield of **3aa-1** was increased (entry 14). This result indicated that low concentration would be important for 1,5-HAT over Giese addition on α-silyl radical intermediate **G** (Scheme 1C). Control experiments revealed that light irradiation, photoredox catalyst, and PMP are essential for this reaction (entries 15–17).

With the optimized reaction conditions in hand, we aimed to investigate the substrate scope of this reaction. First, the scope of alkenes was examined using **1a** (Figure 1, top). Various electron-deficient alkenes were demonstrated and used as an alkylation reagent. The reactions with benzyl acrylate, methyl vinyl ketone, and cyclopentenone afforded the corresponding alkylation products in high yields (**3ab**–**3ad**). A broad range of functional groups including amide, nitrile, sulfone, and phosphonate were compatible (**3ae**–**3ah**). Additionally, 4-vinylpyridine also acts as a radical acceptor (**3ai**). When the reaction with phenylallyl sulfone was conducted, the C(sp^3^)–H allylation product was obtained, albeit in low yield (**3aj**).

**Figure 1:**
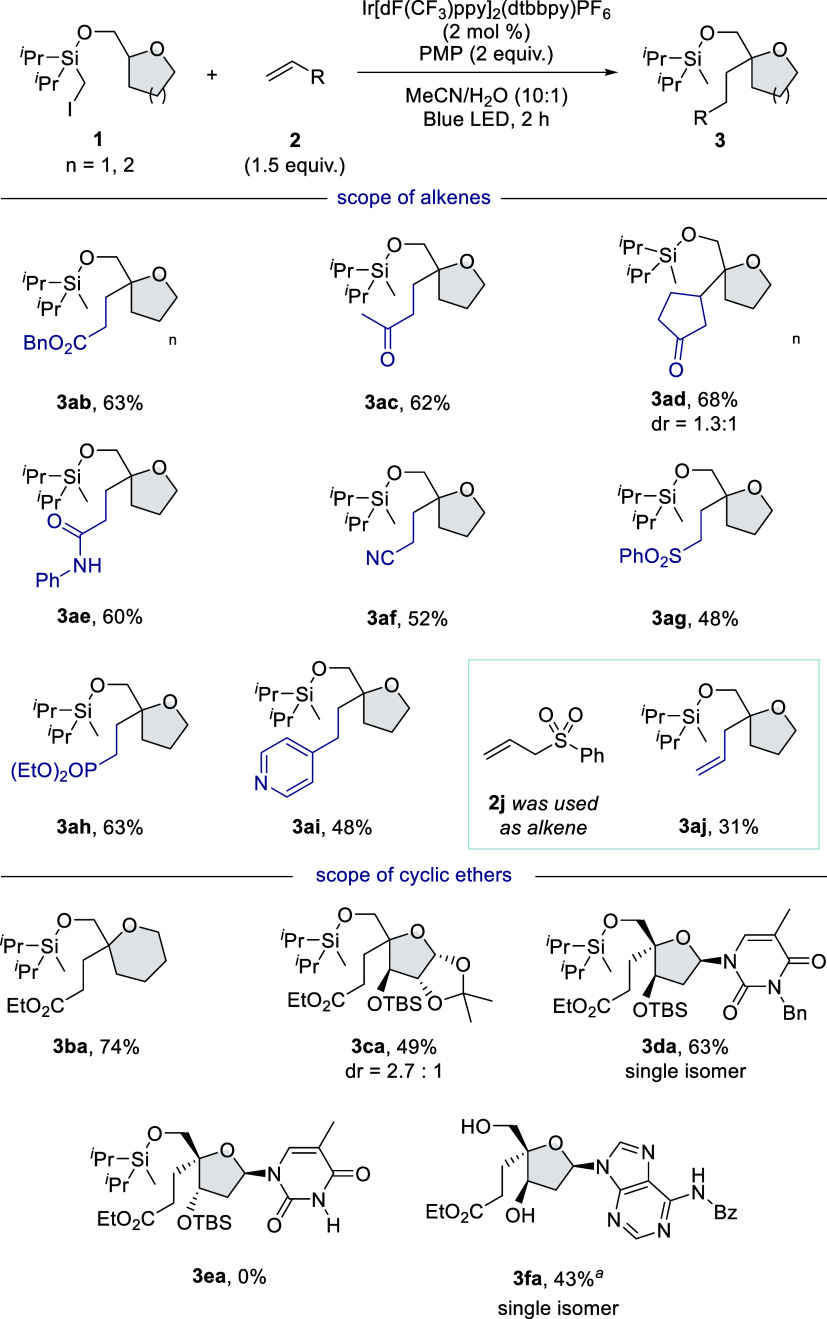
Substrate scope. Reaction was carried out with **1** (0.1 mmol), **2** (0.15 mmol), Ir[dF(CF_3_)ppy]_2_(dtbbpy)PF_6_ (0.002 mmol), and PMP (0.2 mmol) in MeCN (10 mL) and water (1 mL) under blue LED irradiation for 2 h. Isolated yield based on **1**. ^*a*^Isolated as desilylated product. Isolated yield in 2 steps including 4′-alkylation and desilylation.

Subsequently, we evaluated the scope of tetrahydrofurfuryl alcohol derivatives (Figure 1, bottom). Our protocol was applicable to tetrahydropyran-2-methanol (**3ba**). The reaction with a substrate prepared from α-d-xylofuranose proceeded well to produce alkylation product **3ca** with moderate diastereoselectivity. Encouraged by this result, 3′-epimerized thymidine substrate was subjected to the optimal reaction conditions, and we found that the reaction proceeded well to afford a single isomer in good yield (**3da**). The stereochemistry was determined by NOESY (nuclear overhauser effect spectroscopy). In contrast, our protocol was not applicable to 4′-alkylation of thymidine with natural stereoconfiguration (**3ea**). We could not analyze the reaction due to the complexity of the crude. Along with 3′-epimerized thymidine, 4′-alkylated 3-epimerized deoxyadenosine was obtained as a single stereoisomer by this protocol (**3fa**). These results indicated that 3′ stereochemistry would significantly affect 1,5-HAT and the Giese addition step. Also, the stereochemistry of the 4′-position was retained under the reaction conditions.

To gain mechanistic information on this C–H alkylation reaction, several experiments were conducted (Figure 2). When the reaction of **1c** and **2a** was performed with deuterium oxide instead of water, deuterium incorporation at the α-carbonyl position of the product was observed (Figure 2A). Next, we found that the reaction was significantly inhibited by TEMPO (2,2,6,6-tetramethylpiperidine 1-oxyl radical) (Figure 2B). These results were consistent with the proposed reaction pathway shown in Scheme 1C. When the HAT auxiliary was introduced at the 3′-position, 4′-C–H was also cleavable via the 1,5-HAT process. Then, the reaction with **1g** bearing an iodomethylsilyl directing group on the 3′-*O* position was conducted under optimal reaction conditions, and we found that the desired 4′-alkylated product **3ga** was obtained albeit in low yield (Figure 2C). Encouraged by this result, we reevaluated the thymidine derivatives bearing natural stereoconfiguration and found that the reaction of **1h** afforded the desired product **3ha** (5%) with the formation of **3h-1** (15%), **3ha-1** (28%), and **3ha-2** (25%) (Figure 2D). This result indicated that 1,6-HAT and Giese addition of the α-silylmethyl radical compete with 1,5-HAT on thymidine substrates.

**Figure 2:**
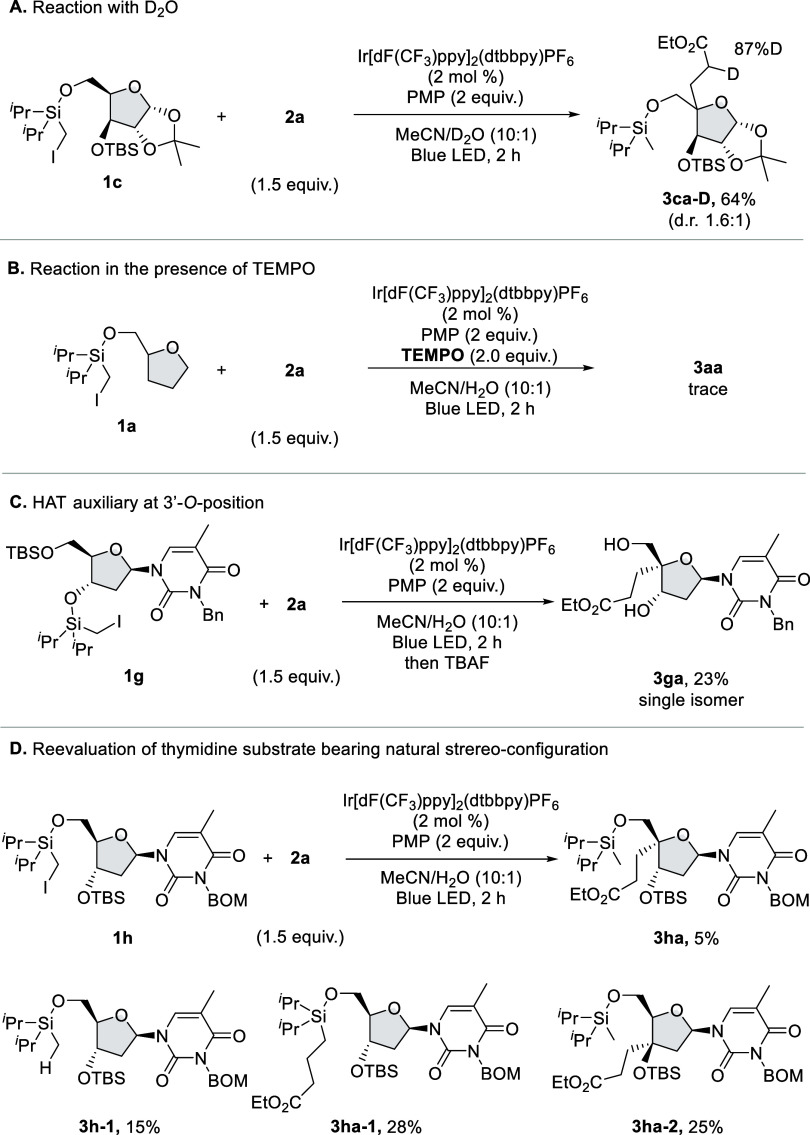
Mechanistic studies.

We performed density functional theory (DFT) calculations to understand the regioselectivity on HAT observed in **1d** and contrasting results induced by 3′-stereochemistry observed in **1d** and **1e** or **1h** (Figure 3).^17^ On the basis of recent reports by Gevorgyan’s group,^16^ an alcohol-tethered silylmethyl radical has the potential to reach C(sp^3^)–H bonds at the β and γ position of alcohol via the 1,5-HAT and 1,6-HAT manner, respectively. In the reaction of **1d**, only the 1,5-HAT product was obtained (Figure 3A). Thus, HAT and Giese addition steps on the reaction of **1d** were examined, and then 1,5-HAT was found to be kinetically favored over 1,6-HAT (Δ*G*^‡^ = 13.1 kcal/mol for 1,5-HAT[**TS1–1**] vs 20.7 kcal/mol for 1,6-HAT[**TS2–1**]). Subsequently, the facial selectivity on Giese addition of 4′-carbon-centered radical [**Int1–2**] to methyl acrylate was explored. Although 4′-epimerized conformational isomer **Int1–2′** was more stable than **Int1–2**, the radical addition preferentially proceeds on the opposite side of the nucleobase (Δ*G*^‡^ = 19.1 kcal/mol for [**TS1–3**] vs 22.5 kcal/mol for [**TS1–3′**]). These results were consistent with the experimental results. As shown as **3aa-1** in Table 1, Giese addition of the α-silylmethyl radical to methyl acrylate was also considered as one of the competing pathways and calculated. The energy barrier was found to be higher than that of 1,5-HAT (Δ*G*^‡^ = 17.0 kcal/mol for [**TS1–5**] vs 13.1 kcal/mol for [**TS1–1**]). Subsequently, an energy diagram of the reaction of **1e** was explored to understand why this reaction did not proceed well (Figure 3B). In the reaction of **1e** or **1h**, both 1,5-HAT and 1,6-HAT processes have a slightly higher energy barrier than 1,5-HAT on the reaction of **1d** (Δ*G*^‡^ = 17.5 kcal/mol for [**TS3–1**] vs 16.2 kcal/mol for [**TS4–1**]), and the Giese addition of α-silylmethyl radical to methyl acrylate has a comparable activation barrier (Δ*G*^‡^ = 17.5 kcal/mol for [**TS3–5**]). These results provided a reasonable explanation for the product distribution observed in Figure 2D.

**Figure 3:**
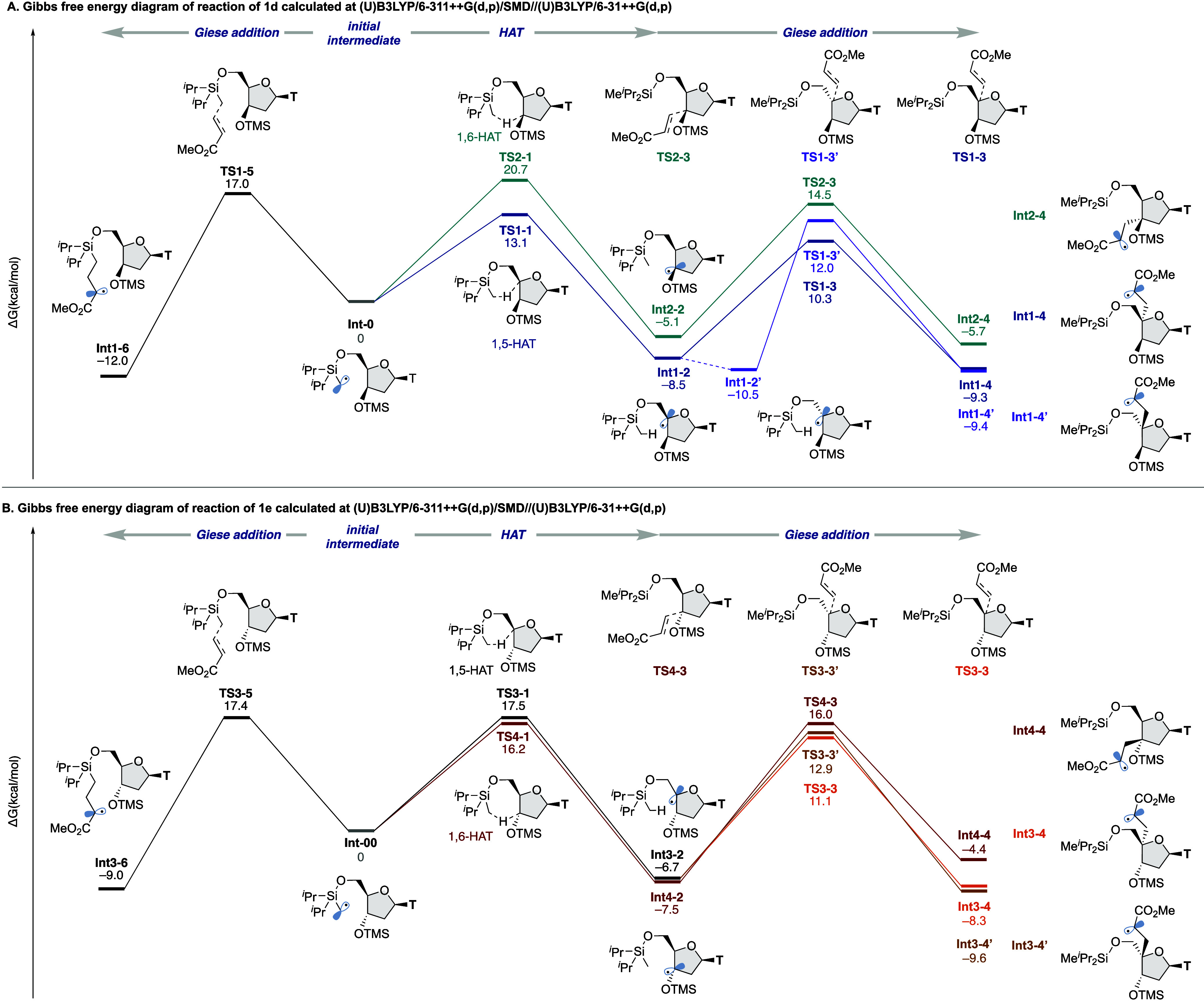
Computational studies.

In summary, we developed a photoredox-catalyzed protocol for site-selective intermolecular C(sp^3^)–H alkylation of tetrahydrofurfuryl alcohol derivatives using a photoredox catalyst and radical-based directing group. This protocol was extended to 4′-carbofunctionalization of 3′-epimerized nucleosides. DFT calculations indicated that the stereochemistry of the 3′-position affects the efficiency of the 1,5-HAT process. This report exhibits the synthetic potentials for stereocontrolled intermolecular functionalization with carbon-centered radicals on nucleoside scaffolds.

## Data Availability

The data underlying this study are available in the published article and its Supporting Information.

## Supporting Information Available

Experimental details and characterization data for all new compounds (PDF)

## References

[ref1] Yoshida Y.; Honma M.; Kimura Y.; Abe H. Structure, Synthesis and Inhibition Mechanism of Nucleoside Analogues as HIV-1 Reverse Transcriptase Inhibitors (NRTIs). ChemMedChem. 2021, 16, 743–766. 10.1002/cmdc.202000695. 33230979

[ref2] Wan W. B.; Seth P. P. The Medicinal Chemistry of Therapeutic Oligonucleotides. J. Med. Chem. 2016, 59, 9645–9667. 10.1021/acs.jmedchem.6b00551. 27434100

[ref3] aNawrat C. C.; Whittaker A. M.; Huffman M. A.; McLaughlin M.; Cohen R. D.; Andreani T.; Ding B.; Li H.; Weisel M.; Tschaen D. M. Nine-Step Stereoselective Synthesis of Islatravir from Deoxyribose. Org. Lett. 2020, 22, 2167–2172. 10.1021/acs.orglett.0c00239. 32108487

[ref4] aObika S. Development of Bridged Nucleic Acid Analogues for Antigene Technology. Chem. Pharma. Bull. 2004, 52, 1399–1404. 10.1248/cpb.52.1399. 15577233

[ref5] Capaldo L.; Ravelli D.; Fagnoni M. Direct Photocatalyzed Hydrogen Atom Transfer (HAT) for Aliphatic C–H Bonds Elaboration. Chem. Rev. 2022, 122, 1875–1924. 10.1021/acs.chemrev.1c00263. 34355884 PMC8796199

[ref6] aFrihed T. G.; Bols M.; Pedersen C. M. C–H Functionalization on Carbohydrates. Eur. J. Org. Chem. 2016, 2016, 2740–2756. 10.1002/ejoc.201600121.

[ref7] Ito Y.; Mizuno K.; Sumise S.; Kimura A.; Noguchi N.; Fuchi Y.; Hari Y. Generation of 4′-Carbon Radicals via 1,5-Hydrogen Atom Transfer for the Synthesis of Bridged Nucleosides. Org. Lett. 2022, 24, 7696–7700. 10.1021/acs.orglett.2c03285. 36214750

[ref8] Brunckova J.; Crich D.; Yao Q. Intramolecular Hydrogen Atom Abstraction in Carbohydrates and Nucleosides: Inversion of an α- to β-Mannopyranoside and Generation of Thymidine C-4′ Radicals. Tetrahedron Lett. 1994, 35, 6619–6622. 10.1016/S0040-4039(00)73450-2.

[ref9] Nakajima K.; Miyake Y.; Nishibayashi Y. Synthetic Utilization of α-Aminoalkyl Radicals and Related Species in Visible Light Photoredox Catalysis. Acc. Chem. Res. 2016, 49, 1946–1956. 10.1021/acs.accounts.6b00251. 27505299

[ref10] Juliá F.; Constantin T.; Leonori D. Applications of Halogen-Atom Transfer (XAT) for the Generation of Carbon Radicals in Synthetic Photochemistry and Photocatalysis. Chem. Rev. 2022, 122, 2292–2352. 10.1021/acs.chemrev.1c00558. 34882396

[ref11] Speckmeier E.; Fischer T. G.; Zeitler K. A Toolbox Approach To Construct Broadly Applicable Metal-Free Catalysts for Photoredox Chemistry: Deliberate Tuning of Redox Potentials and Importance of Halogens in Donor–Acceptor Cyanoarenes. J. Am. Chem. Soc. 2018, 140, 15353–15365. 10.1021/jacs.8b08933. 30277767

[ref12] Connell T. U.; Fraser C. L.; Czyz M. L.; Smith Z. M.; Hayne D. J.; Doeven E. H.; Agugiaro J.; Wilson D. J. D.; Adcock J. L.; Scully A. D.; Gómez D. E.; Barnett N. W.; Polyzos A.; Francis P. S. The Tandem Photoredox Catalysis Mechanism of [Ir(Ppy)2(Dtb-Bpy)]+ Enabling Access to Energy Demanding Organic Substrates. J. Am. Chem. Soc. 2019, 141, 17646–17658. 10.1021/jacs.9b07370. 31545022

[ref13] Constantin T.; Zanini M.; Regni A.; Sheikh N. S.; Juliá F.; Leonori D. Aminoalkyl Radicals as Halogen-Atom Transfer Agents for Activation of Alkyl and Aryl Halides. Science 2020, 367, 1021–1026. 10.1126/science.aba2419. 32108109

[ref14] Prier C. K.; Rankic D. A.; MacMillan D. W. C. Visible Light Photoredox Catalysis with Transition Metal Complexes: Applications in Organic Synthesis. Chem. Rev. 2013, 113, 5322–5363. 10.1021/cr300503r. 23509883 PMC4028850

[ref15] Sun X.; Zheng K. Electrochemical Halogen-Atom Transfer Alkylation via α-Aminoalkyl Radical Activation of Alkyl Iodides. Nat. Commun. 2023, 10.1038/s41467-023-42566-y. PMC10603137 37884528

[ref16] aKurandina D.; Yadagiri D.; Rivas M.; Kavun A.; Chuentragool P.; Hayama K.; Gevorgyan V. Transition-Metal- and Light-Free Directed Amination of Remote Unactivated C(Sp3)–H Bonds of Alcohols. J. Am. Chem. Soc. 2019, 141, 8104–8109. 10.1021/jacs.9b04189. 31046256 PMC6873700

[ref17] aAbazi S.; Rapado L. P.; Renaud P. Diastereoselective Radical Mediated Alkylation of a Chiral Glycolic Acid Derivative. Org. Biomol. Chem. 2011, 9, 5773–5777. 10.1039/c1ob05230f. 21720649

