# Supplementary material for: Photoredox-Catalyzed Site-Selective Intermolecular C(sp3)–H Alkylation of Tetrahydrofurfuryl Alcohol Derivatives
Source: Org Lett. 2025 Jan 14;27(3):795–801. doi: 10.1021/acs.orglett.4c04439 (PMC11773563; doi:10.1021/acs.orglett.4c04439)
Supplement: Supplementary file 1 — ol4c04439_si_001.pdf [file ol4c04439_si_001.pdf]

Photoredox-Catalyzed Site-Selective Intermolecular C(sp^3)–H Alkylation of Tetrahydrofurfuryl Alcohol Derivatives

Reiji Abe,[1] Kazunori Nagao,[1]* Tomohiro Seki,[2] Dai Hata,[2] Yusuke Sasaki,[2] Hirohisa Ohmiya[1]*
[1]Institute for Chemical Research, Kyoto University, Gokasho, Uji, Kyoto 611-0011, Japan
[2]Research, Takeda Pharmaceutical Company Limited, Fujisawa, Kanagawa, Japan.

*E-mail: Kazunori Nagao: nagao.kazunori.4j@kyoto-u.ac.jp
Hirohisa Ohmiya: ohmiya@scl.kyoto-u.ac.jp

Table of Contents

- Instrumentation and Chemicals S2
- Synthesis and Characterization of Directing Group Tethered Alcohols S3–S8
- General Procedure for C4’ Alkylation by Photoredox Catalysis S8–S9
- Characterization Data for Alkylated Products S9–S15
- Mehanistic Experiments S16–S17
- Directing group at 3’-O-position S18–S19
- (*E*)-Oxime Imidate as a Directing Group S20
- Reevaluation of Non-Epimerized Substrate S21–23
- Computational Studies S24–S81
- References S82
- NMR Spectra S83–S160

■ Instrumentation and Chemicals ■

NMR spectra were recorded on a Bruker AVANCE NEO 400N spectrometer, operating at 400 MHz for ^{1}H NMR, 100.6 MHz for ^{13}C NMR, 376.5 MHz for ^{19}F NMR, 128.4 MHz for ^{11}B NMR. Chemical shift values for ^{1}H and ^{13}C are referenced to Me_4Si and the residual solvent resonances, respectively. Structural assignments were made with additional information from NOESY experiments. Chemical shifts were reported in d ppm. Mass spectra were obtained with Bruker timsTOF mass spectrometer (ESI and APCI) and Bruker Impact HD mass spectrometer (ESI). TLC analyses were performed on commercial glass plates bearing 0.25-mm layer of Merck Silica gel 60F254. Silica gel (Wakosil® 60, 64~210 μm) was used for column chromatography. Biotage Selekt was used for purification. Melting points were measured on a Stanford Research Systems MPA100. IR spectra were measured with a Thermo Scientific iD7 ATR Accessory for the Thermo Scientific Nicolet iS5 FT-IR Spectrometer.Kessil A160W Tuna Blue (highest blue and intensity setting) was used as a light source. TEKNOS MG9 was used as a fan.

All reactions were carried out under nitrogen atmosphere. Materials were obtained from commercial suppliers or prepared according to standard procedures unless otherwise noted. $Ir(ppy)_3$, $Ir(ppy)_2(dtbbpy)PF_6$ and PMP were purchased from Aldrich Chemical Co. $Ir[dF(CF_3)ppy]_2(dtbbpy)PF_6$, 4CzIPN was prepared by reported procedure.[1,2] $Et^{i}Pr_2N$, TBAF (1.0 M in THF) and Bn_3N were purchased from Tokyo Chemical Industry Co. Triethylamine was purchased from Nacalai Tesque Inc. THF, toluene, acetone, DMF, DMSO and MeCN were purchased from FUJIFILM Wako Pure Chemical Co., stored under nitrogen, and used as received. Alkenes **2a**–**d**, **2f**–**2i** were purchased from Tokyo Chemical Industry Co., stored under nitrogen, and used after filtration through a short pad of alumina. Alkene **2e** was purchased from Aldrich Chemical Co., stored under nitrogen. Alkene **2j** was synthesized according to the reported procedure.[3]

■ Synthesis and Characterization of Directing Group Tethered Alcohols■

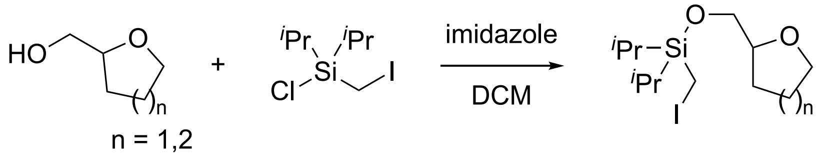

Supplementary Fig. 1. Synthesis of DG tethered fulfuryl alcohol derivatives.

Synthesis of **1a** and **1b**. To a stirred mixture of furfuryl alcohol (1.0 equiv), imidazole (3.0 equiv), and DCM (0.2 M), (chloro)diisopropyliodomethylsilane[4] (1.2 equiv.) were added at rt under nitrogen atmosphere. After stirring at room temperature for 2 h, solvent was removed in vacuo. The obtained reaction mixture was purified by column chromatography.

(Iodomethyl)diisopropyl[(tetrahydrofuran-2-yl)methoxy]silane (1a)

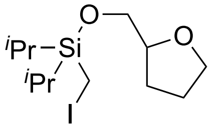

The product **1a** was purified by flash chromatography (2.0 mmol scale, 641 mg, 70% yield, Biotage Selekt, 100:0–95:5, hexane/EtOAc). Colorless oil. **IR** (neat) 2943, 2865, 1462, 1378, 1140, 1084, 986, 919, 881, 728 cm^{-1}. **^{1}H NMR** (400 MHz, $CDCl_3$) δ 4.00 (m, 1H), 3.84 (m, 1H), 3.79–3.69 (m, 3H), 2.09 (s, 2H), 1.98–1.70 (m, 4H), 1.28–1.19 (m, 2H), 1.09–1.06 (m, 12H). **^{13}C NMR** (100 MHz, $CDCl_3$) δ 79.3, 68.4, 66.3, 27.7, 25.8, 17.6, 17.6, 17.4, 12.2, 12.1. **HRMS–ESI** (m/z):$[M+H]^+$ calcd for $C_{12}H_{26}IO_2Si$; 357.0741, found 357.0745.

(Iodomethyl)diisopropyl[(tetrahydro-2*H*-pyran-2-yl)methoxy]silane (1b)

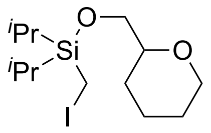

The product **1b** was purified by flash chromatography (1.0 mmol scale, 332 mg, 90% yield, Biotage Selekt, 97:3–95:5, hexane/EtOAc). Colorless oil. **IR** (neat) 2938, 2865, 1462, 1126, 1095, 1081, 1048, 881, 728, 374 cm^{-1}. **^{1}H NMR** (400 MHz, $CDCl_3$) δ 3.97 (dddd, J = 11.2, 6.0, 3.6, 1.6 Hz, 1H), 3.76 (dd, J = 10.4, 5.6 Hz, 1H), 3.60 (dd, J = 10.4, 5.6 Hz, 1H), 3.46–3.36 (m, 2H), 2.10 (s, 2H), 1.84 (m, 1H), 1.68 (m, 1H), 1.59–1.43 (m, 3H), 1.31–1.18 (m, 3H), 1.09–1.06 (m, 12H). **^{13}C NMR** (100 MHz, $CDCl_3$) δ 78.3, 68.3, 67.4, 28.3, 26.1, 23.1, 17.6 (2C), 17.4, 12.2, 12.1. **HRMS–ESI** (m/z):$[M+H]^+$ calcd for $C_{13}H_{28}IO_2Si$; 371.0898, found 371.0918.

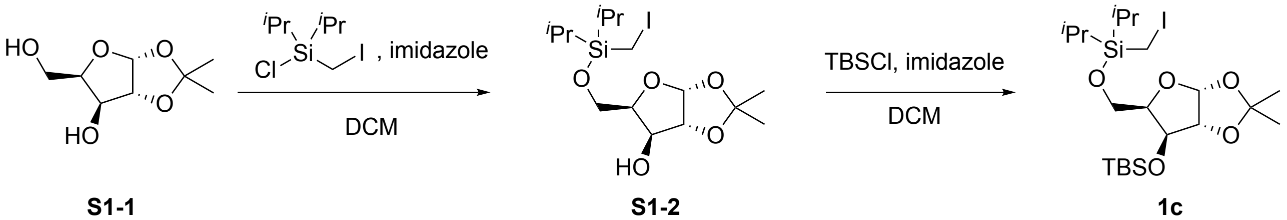

Supplementary Fig. 2. Synthesis of α-D-xylofuranose derivative **1c**

Synthesis of 1c. To a solution of (chloro)diisopropyliodomethylsilane (320 mg, 1.1 mmol) and imidazole (204 mg, 3 mmol) in DCM (5 mL), α-xylofuranose **S1-1** (190 mg, 1mmol) were added. The reaction mixture was stirred overnight at room temperature under nitrogen atmosphere. After the solvent was removed under reduced pressure, purification by flash column chromatography on silica gel (Biotage Selekt, 100:0–80:20, hexane/AcOEt) gave the C5' tethered alcohol **S1-2** as colorless oil (369 mg, 0.83 mmol, 83%).

To a solution of **S1-2** (288 mg, 0.65 mmol) and imidazole (133 mg, 1.95 mmol) in DCM (3 mL), TBSCl (108 mg, 0.72 mmol) was added. The reaction mixture was stirred for 5 h at room temperature under nitrogen atmosphere. After the solvent was removed under reduced pressure, purification by flash column chromatography on silica gel (90:10–80:20, hexane/AcOEt) gave the C3' protected alcohol **1c** as colorless oil (274 mg, 0.49 mmol, 76%).

(3*R*,6*S*,6*R*)-5-{[(Iodomethyl)diisopropylsilyl)oxy]methyl}-2,2-dimethyltetrahydrofuro[2,3-d][1,3]dioxol-6-ol (S1-2)

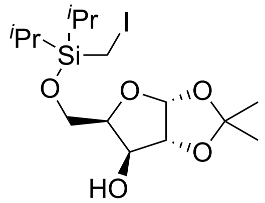

Colorless oil. **IR** (neat) 2935, 2865, 1252, 1164, 1080, 1015, 883, 834, 777, 729 cm^{-1}. **^{1}H NMR** (400 MHz, $CDCl_3$) δ 5.96 (d, *J* = 3.6 Hz, 1H), 4.52 (d, *J* = 3.6 Hz, 1H), 4.36 (d, *J* = 2.4 Hz, 1H), 4.36–4.16 (m, 3H), 3.80 (d, *J* = 2.8 Hz, 1H), 2.11 (s, 2H), 1.49 (s, 3H), 1.32 (s, 3H), 1.31–1.22 (m, 2H), 1.10–1.06 (m, 12H). **^{13}C NMR** (100 MHz, $CDCl_3$) δ 111.5, 104.9, 85.4, 78.3, 62.6, 26.8, 26.1, 17.5, 17.4, 17.3, 17.2, 12.0, 11.9. **HRMS–ESI** (*m/z*): [M+H]$^+$ calcd for $C_{15}H_{30}IO_5Si$; 445.0902, found 445.0906.

***tert*-Butyl{[(3*R*,6*S*,6*R*)-5-{[(iodomethyl)diisopropylsilyl]oxy}methyl]-2,2-dimethyltetrahydrofuro[2,3-d][1,3]dioxol-6-yl)oxy)dimethylsilane (1c)**

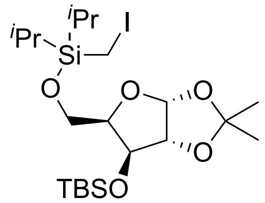

Colorless oil. **IR** (neat) 2935, 2865, 1252, 1164, 1080, 1015, 883, 834, 777, 729 cm^{-1}. **^{1}H NMR** (400 MHz, $CDCl_3$) δ 5.88 (d, *J* = 3.6 Hz, 1H), 4.35 (d, *J* = 3.6 Hz, 1H), 4.25–4.21 (m, 2H), 3.96 (dd, *J* = 10.4, 6.4 Hz, 1H), 3.80 (dd, *J* = 10.4, 5.6 Hz, 1H), 2.09 (s, 2H), 1.50 (s, 3H), 1.31 (s, 3H), 1.31–1.19 (m, 2H), 1.09–1.05 (m, 12H), 0.89 (s, 9H), 0.12 (s, 3H), 0.10 (s, 3H). **^{13}C NMR** (100 MHz, $CDCl_3$) δ 111.7, 104.9, 85.5, 75.1, 61.2, 26.9, 26.4, 25.7, 18.0, 17.6, 17.5, 17.4, 17.3, 12.2, 12.1, –4.7, –5.0. **HRMS–ESI** (*m/z*): [M+H]$^+$ calcd for $C_{21}H_{44}IO_5Si_2$; 559.1767, found 559.1771.

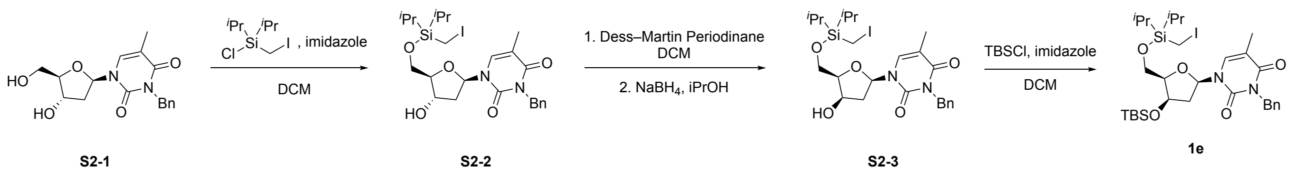

Supplementary Fig. 3. Synthesis of thymidine derivative **1e**

Synthesis of 1e. Thymidine **S2-1** was prepared according to the literature.[5] To a solution of (chloro)diisopropyliodomethylsilane (664 mg, 2.0 mmol) and imidazole (408 mg, 6.0 mmol) in DCM (5 mL), thymidine **S1-1** (665 mg, 2.0 mmol) was added. The reaction mixture was stirred overnight at room temperature under nitrogen atmosphere. After the solvent was removed under reduced pressure, purification by flash column chromatography on silica gel (Biotage Selekt, 70:30–60:40, hexane/AcOEt) gave the C5' tethered thymidine **S2-2** as colorless oil (757 mg, 1.32 mmol, 66%).

To a solution of Dess–Martin periodinane (561 mg, 1.32 mmol) in DCM (3 mL), **S2-2** (379 mg, 0.66 mmol) was added.[5] The reaction mixture was stirred overnight at room temperature under nitrogen atmosphere. After the solvent was removed under reduced pressure, ^{i}PrOH (6 mL) was added, and the reaction mixture was cooled to –60 °C, followed by addition of $NaBH_4$ (50 mg, 1.32 mmol). The reaction mixture was stirred at –60 °C for another 16 h, and 6 mL of acetone was subsequently added. The resulting mixture was allowed to warm to room temperature and poured into EtOAc. The organic phase was wased with aqueous $NaHCO_3$, H_2O and brine, dried over Na_2SO_4, filtered and concentrated under reduced pressure. The crude product was puridied by flash column chromatography on silica gel (98:2, DCM/acetone) and gave the C3' inverted thymidine **S2-3** (301 mg, 0.53 mmol, 80% in 2 steps) as colorless oil.

To a solution of **S2-3** (171 mg, 0.3 mmol) and imidazole (61 mg, 0.9 mmol) in DCM (3 mL), TBSCl (68 mg, 0.45 mmol) was added. The reaction mixture was stirred for 5 h at room temperature under nitrogen atmosphere. After the solvent was removed under reduced pressure, purification by flash column chromatography on silica gel (Biotage Selekt, 100:0–90:10, hexane/AcOEt) gave the C3' protected thymidine **1e** as colorless oil (86 mg, 0.12 mmol, 44%).

3-Benzyl-1-[(2*R*,4*S*,5*R*)-4-hydroxy-5-({[(iodomethyl)diisopropylsilyl]oxy}methyl)tetrahydrofuran-2-yl]-5-methylpyrimidine-2,4(1*H*,3*H*)-dione (S2-2)

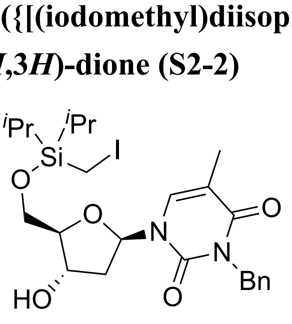

Colorless oil. **IR** (neat) 2944, 1700, 1669, 1663, 1653, 1647, 1635, 1123, 1100, 1003 cm^{-1}. **^{1}H NMR** (400 MHz, $CDCl_3$) δ 7.48 (dd, *J* = 8.0, 1.2 Hz, 2H), 7.39 (d, *J* = 1.2 Hz, 1H), 7.22–7.31 (m, 3H), 6.36 (dd, *J* = 7.2, 6.0 Hz, 1H), 5.12 (d, *J* = 5.2 Hz, 2H), 4.63 (m, 1H), 4.00 (d, 0.8 Hz, 3H), 2.37 (m, 1H), 2.15 (m, 1H), 2.10 (s, 2H), 1.94 (d, 1.2 Hz, 3H), 1.87 (m, 1H), 1.32–1.19 (m, 2H), 1.11–1.07 (m, 12H). **^{13}C NMR** (100 MHz, $CDCl_3$) δ 163.4, 150.9, 136.8, 133.5, 129.0 (2C), 128.3, 127.5,

110.1, 86.6, 85.4, 71.9, 63.7, 44.5, 41.0, 17.6, 17.53, 17.46, 17.43, 13.3, 12.3, 12.2. **HRMS–ESI** (m/z):[M+H]$^+$ calcd for $C_{24}H_{36}IN_2O_5Si$; 587.1433, found 587.1440.

3-Benzyl-1-[(2*R*,4*R*,5*R*)-4-hydroxy-5-({[(iodomethyl)diisopropylsilyl]oxy}methyl)tetrahydrofuran-2-yl]-5-methylpyrimidine-2,4(1*H*,3*H*)-dione (S2-3)

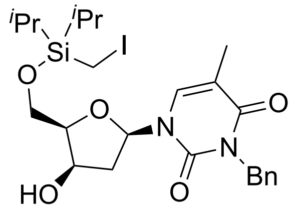

Colorless oil. **IR** (neat) 2943, 1697, 1664, 1631, 1467, 1268, 1072, 768, 731, 699 cm^{-1}. **^{1}H NMR** (400 MHz, $CDCl_3$) δ 7.70 (d, *J* = 1.2 Hz, 1H), 7.48 (dd, *J* = 8.2, 1.2 Hz, 2H), 7.31–7.22 (m, 3H), 6.25 (dd, *J* = 8.4, 3.2 Hz, 1H), 5.12 (s, 2H), 4.55 (m, 1H), 4.18–4.21 (m, 2H), 3.91 (ddd, *J* = 5.6, 4.8, 3.2 Hz, 1H), 3.74 (s, 1H), 2.62 (ddd, *J* = 14.8, 8.4, 5.6 Hz, 1H), 2.11–2.04 (m, 3H), 1.94 (s, 3H), 1.30–1.20 (m, 2H), 1.20–1.06 (m, 12H). **^{13}C NMR** (100 MHz, $CDCl_3$) δ 163.4, 150.9, 136.8, 133.5, 129.0 (2C), 128.3, 127.5, 110.1, 86.6, 85.4, 71.9, 63.7, 44.5, 41.0, 17.5, 17.44, 17.40, 17.35, 13.3, 12.3, 12.2. **HRMS–ESI** (m/z):[M+H]$^+$ calcd for $C_{24}H_{36}IN_2O_5Si$; 587.1433, found 587.1432.

3-Benzyl-1-[(2*R*,4*R*,5*R*)-4-[(*tert*-butyldimethylsilyl)oxy]-5-({[(iodomethyl)diisopropylsilyl]oxy}methyl)tetrahydrofuran-2-yl]-5-methylpyrimidine-2,4(1*H*,3*H*)-dione (1e)

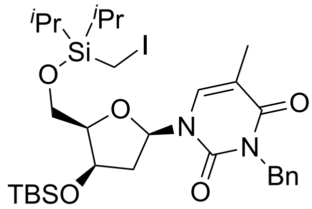

Colorless oil. **IR** (neat) 1700, 1663, 1641, 1436, 1258, 1101, 1077, 837, 778, 698 cm^{-1}. **^{1}H NMR** (400 MHz, $CDCl_3$) δ 7.55 (d, J = 1.2 Hz, 1H), 7.45 (dd, *J* = 8.0, 1.2 Hz, 2H), 7.30–7.23 (m, 3H), 6.18 (dd, *J* = 8.0, 2.0 Hz, 1H), 5.11 (s, 2H), 4.36 (dd, *J* = 4.4, 2.8 Hz, 1H), 4.12–3.99 (m, 3H), 2.56 (ddd, *J* = 12.8, 8.0, 4.4 Hz, 1H), 2.10 (s, 2H), 2.00 (dd, *J* = 12.8, 2.0 Hz, 1H), 1.93 (s, 3H), 1.32–1.19 (m, 2H), 1.10–1.06 (m, 12H), 0.83 (s, 9H), 0.08 (s, 3H), 0.00 (s, 3H). **^{13}C NMR** (100 MHz, $CDCl_3$) δ 163.5, 151.0, 137.0, 134.7, 128.9 (2C), 128.3, 127.4, 109.0, 85.7, 85.7, 70.5, 62.0, 44.3, 42.3, 25.5, 17.9, 17.6, 17.5, 17.4, 17.3, 13.4, 12.2, 12.1, –4.9, –5.2. **HRMS–ESI** (m/z):[M+H]$^+$ calcd for $C_{30}H_{50}IN_2O_5Si_2$; 701.2298, found 701.2305.

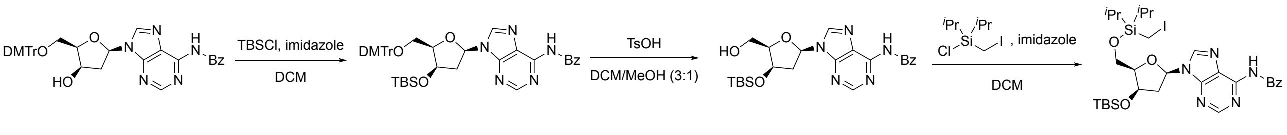

S3-1 **S3-2** **S3-3** **1f**

Supplementary Fig. 4. Synthesis of deoxyadenosine derivative **1f**

Synthesis of 1f. C3'-epimerized deoxyadenosine **S3-1** was prepared according to the literature.[6] To a solution of **S3-1** (498 mg, 0.78 mmol) and imidazole (212 mg, 3.1 mmol) in DCM (4 mL), TBSCl

(235 mg, 1.56 mmol) was added. The reaction mixture was stirred for 24 h at room temperature under nitrogen atmosphere. After the solvent was removed under reduced pressure, purification by flash column chromatography on silica gel (Biotage Selekt, 50:50–30:70, hexane/AcOEt) gave the C3' protected deoxyadenosine **S3-2** as a white amorphous solid (436 mg, 0.57 mmol, 73%).

To the solution of **S3-2** (436 mg, 0.57 mmol) in DCM/MeOH (3:1) (80 mL), TsOH (118 mg, 0.68 mmol) was added. The reaction mixture was stirred for 30 min at 0 °C under nitrogen atmosphere. To the reaction mixture $NaHCO_3$ (72 mg, 0.85 mmol) was added and stirred until the orange solution disappeared. After the solvent was removed under reduced pressure, purification by flash column chromatography on silica gel (Biotage Selekt, 100:0–95:5, AcOEt/MeOH) gave the the C5' deprotected deoxyadenosine **S3-3** (209 mg, 0.46 mmol, 81%) as white amorphous solid.

To a solution of (chloro)diisopropyliodomethylsilane (209 mg, 0.46 mmol) and imidazole (94 mg, 1.4 mmol) in DCM (3 mL), **S3-3** (208 mg, 0.46 mmol) was added. The reaction mixture was stirred overnight at room temperature under nitrogen atmosphere. After the solvent was removed under reduced pressure, purification by flash column chromatography on silica gel (Biotage Selekt, 60:40–50:50, hexane/AcOEt) gave the C5' tethered deoxyadenosine **1f** as a brown amorphous solid (201 mg, 0.28 mmol, 62%).

***N*-[9-((2*R*,4*R*,5*R*)-5-{[bis(4-methoxyphenyl)(phenyl)methoxy]methyl}-4-[(*tert*-butyldimethylsilyl)oxy]tetrahydrofuran-2-yl)-9*H*-purin-6-yl]benzamide (S3-2)**

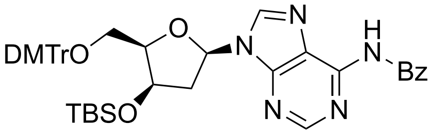

Amorphous solid. **IR** (neat) 1088, 1176, 1216, 1251, 1297, 1452, 1457, 1508, 1581, 1608 cm^{-1}. **^{1}H NMR** (400 MHz, $CDCl_3$) δ 9.19 (s, 1H), 8.80 (s, 1H), 8.26 (s, 1H), 8.03 (d, *J* = 7.4 Hz, 2H), 7.58 (m, 1H), 7.53–7.47 (m, 4H), 7.39–7.34 (m, 4H), 7.29–7.19 (m, 3H), 6.81 (d, *J* = 8.8 Hz, 4H), 6.55 (d, *J* = 6.8 Hz, 1H), 4.41–4.36 (m, 2H), 3.78 (s, 6H), 3.61 (dd, *J* = 10.4, 7.2 Hz, 1H), 3.27 (dd, *J* = 10.4, 3.2 Hz, 1H), 2.69 (m, 1H), 2,41 (d, *J* = 14.4 Hz, 1H), 0.59 (s, 9H), –0.18 (s, 3H), –0.26 (s, 3H). **^{13}C NMR** (100 MHz, $CDCl_3$) δ 164.6, 158.48, 158.46, 152.4, 150.9, 149.2, 144.6, 141.9, 136.0, 135.8, 133.9, 132.6, 130.0, 129.9, 129.1, 128.8, 128.1, 127.8, 126.8, 122.9, 113.14, 113.11, 86.5, 85.5, 84.7, 71.7, 63.6, 55.2, 42.4, 29.6, 25.4, 17.7, –5.1, –5.6. **HRMS–ESI** (m/z):$[M+H]^+$ calcd for $C_{44}H_{50}N_5O_6Si$; 772.3525, found 772.3529.

***N*-(9-{(2*R*,4*R*,5*R*)-4-[(*tert*-butyldimethylsilyl)oxy]-5-(hydroxymethyl)tetrahydrofuran-2-yl}-9*H*-purin-6-yl)benzamide (S3-3)**

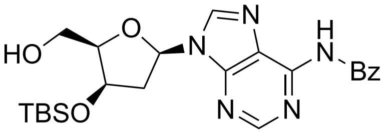

Amorphous solid. **IR** (neat) 1216, 1255, 1295, 1403, 1456, 1516, 1581, 1611, 1700, 2858 cm^{-1}. **^{1}H NMR** (400 MHz, $CDCl_3$) δ 9.30 (s, 1H), 8.78 (s, 1H), 8.52 (s, 1H), 8.04 (d, *J* = 7.2 Hz, 2H), 7.59 (t, *J* = 7.2 Hz, 1H), 7.51 (t, *J* = 7.2 Hz, 2H), 6.48 (dd, *J* = 7.2, 2.0 Hz, 1H), 4.58 (m, 1H), 4.19 (dd, *J* =

9.6, 4.0 Hz, 1H), 4.04 (dd, *J* = 12.0, 5.6 Hz, 1H), 3.95 (dd, *J* = 12.0, 4.0 Hz, 1H), 2.75 (m, 1H), 2.51 (m, 1H), 0.80 (s, 9H), 0.08 (s, 3H), –0.06 (s, 3H). **^{13}C NMR** (100 MHz, $CDCl_3$) δ 164.7, 152.6, 151.0, 149.4, 141.8, 133.8, 132.7, 128.8, 127.9, 122.7, 85.1, 84.0, 72.1, 61.9, 42.3, 25.6, 17.9, –4.9, –5.3. **HRMS–ESI** (m/z):$[M+H]^+$ calcd for $C_{23}H_{32}N_5O_4Si$; 470.2218, found 470.2227.

***N*-{9-[(2*R*,4*R*,5*R*)-4-[(*tert*-butyldimethylsilyl)oxy]-5-({[(iodomethyl)diisopropylsilyl]oxy}methyl)tetrahydrofuran-2-yl]-9*H*-purin-6-yl}benzamide (1f)**

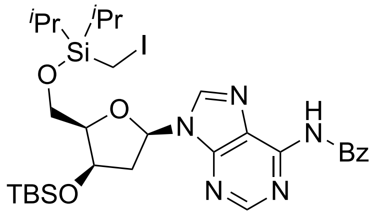

Amorphous solid. **IR** 2947, 1610, 1581, 1456, 1253, 1216, 1093, 1070, 831, 777 cm^{-1}. **^{1}H NMR** (400 MHz, $CDCl_3$) δ 8.98 (s, 1H), 8.81 (s, 1H), 8.45 (s, 1H), 8.03–8.00 (m, 2H), 7.59 (m, 1H) 7.54–7.51 (m, 2H), 6.53 (dd, *J* = 8.0, 1.2 Hz, 1H), 4.54 (dd, *J* = 4.0, 3.2 Hz, 1H), 4.23–4.07 (m, 3H), 2.74 (m, 1H), 2.44 (d, *J* = 14.8 Hz, 1H), 2.10 (s, 2H), 1.57–1.21 (m, 2H), 1.10–1.06 (m, 12H), 0.81 (s, 9H), 0.10 (s, 3H), –0.06 (s, 3H). **^{13}C NMR** (100 MHz, $CDCl_3$) δ 164.61, 152.5, 151.1, 149.2, 142.0, 133.8, 132.6, 128.8, 127.8, 122.7, 86.3, 84.0, 71.0, 62.2, 42.4, 25.6, 18.0, 17.6, 17.5, 17.4, 17.3, 17.2, 12.2, 12.1, –4.8, –5.2. **HRMS–ESI** (m/z):$[M+H]^+$ calcd for $C_{30}H_{47}IN_5O_4Si_2$; 724.2206, found 724.2222.

■ General Procedure for C4' Alkylation by Photoredox Catalysis ■

The reaction in Table 1, entry 1 is representative. In a glovebox, to an oven-dried vial with a stirring bar and $Ir[dF(CF_3)ppy]_2(dtbbpy)PF_6$ (2.2 mg, 0.002 mmol) was added (iodomethyl)diisopropyl[(tetrahydrofuran-2-yl)methoxy]silane **1a** (35.6 mg, 0.1 mmol) in MeCN(1 mL). Then, MeCN (9 mL), degassed H_2O (1 mL), PMP (31 mg, 0.2 mmol), and ethyl acrylate (15 mg, 0.15 mmol) were sequentially added to the vial. After sealing the vial with a cap and removed from the glovebox, the reaction was stirred and irradiated with 45W blue in EvoluChem photoreactor (PhotoRedOx Duo) (Supplementary Fig. 5). After 2 h, the solvent was removed under reduced pressure. The crude product was then purified by flash column chromatography on silica gel (100:0–95:5, hexane/AcOEt) to give the alkylated product **3aa** (22.2 mg, 0.067 mmol, 67% isolated yield) as colorless oil.

1 mmol scale reaction of 3aa. In a glovebox, to an oven-dried vial with a stirring bar and $Ir[dF(CF_3)ppy]_2(dtbbpy)PF_6$ (2.2 mg, 0.002 mmol) was added (iodomethyl)diisopropyl[(tetrahydrofuran-2-yl)methoxy]silane **1a** (356 mg, 1.0 mmol) in MeCN(10 mL). Then, MeCN (40 mL), degassed H_2O (5 mL), PMP (310 mg, 0.2 mmol), and ethyl acrylate (150 mg, 0.15 mmol) were sequentially added to the vial. After sealing the vial with a cap and removed from the glovebox, the reaction was stirred and irradiated with 34W blue LEDs (0.5 cm away) with a cooling fan to keep the temperature around 40 °C (Supplementary Fig. 6). After 2 h, the solvent was removed under reduced pressure. The crude product was then purified by flash column

chromatography on silica gel (100:0–95:5, hexane/AcOEt) to give the alkylated product **3aa** (173.3 mg, 0.52 mmol, 52% isolated yield) as colorless oil.

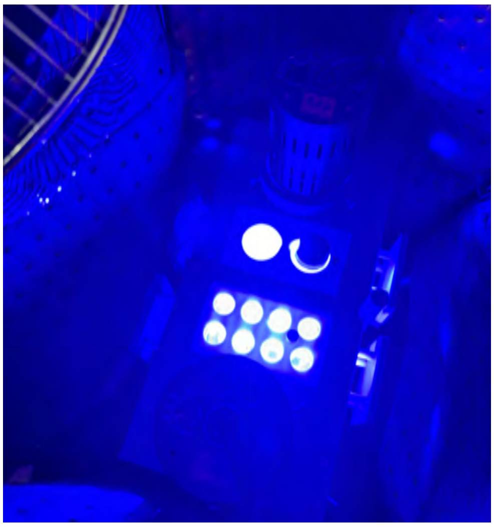

Supplementary Fig. 5. Light set up.

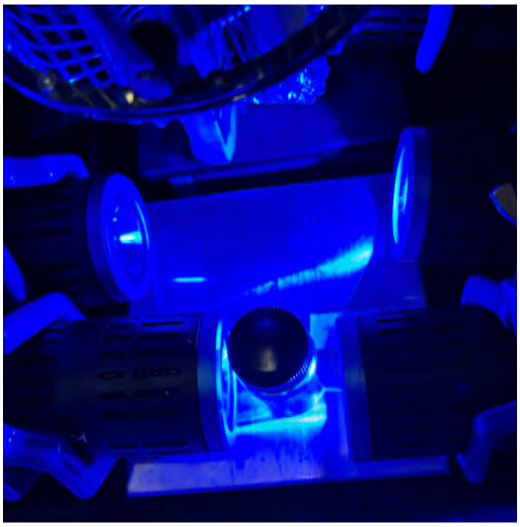

Supplementary Fig. 6 Scale-up reaction.

■ Characterization Data for Alkylated Products ■

Ethyl 3-[2-({[Diisopropyl(methyl)silyl]oxy}methyl)tetrahydrofuran-2-yl]propanoate (3aa)

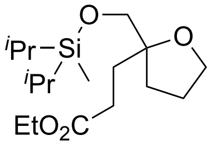

Colorless oil. **IR** (neat) 2941, 2865, 1735, 1251, 1177, 1098, 1042, 881, 820, 779 cm^{-1}. **^{1}H NMR** (400 MHz, $CDCl_3$) δ 8.26 (br, 1H), 7.51 (d, *J* = 7.6 Hz, 2H), 7.30 (t, *J* = 7.6 Hz, 2H), 7.07 (t, *J* = 7.6 Hz, 1H), 3.89 (t, *J* = 6.4 Hz, 2H), 3.50 (d, *J* = 10.0 Hz, 1H), 2.52–2.42 (m, 2H), 2.10–1.89 (m, 5H), 1.66 (m, 1H), 1.01–0.91(m, 14H), 0.02 (s, 3H). **^{13}C NMR** (100 MHz, $CDCl_3$) δ 174.1, 84.2, 68.5, 67.7, 60.2, 32.5, 31.6, 29.2, 29.2, 26.2, 17.5, 17.4, 14.2, 13.0, –8.7. **HRMS–ESI** (m/z):[M+H]$^+$ calcd for $C_{17}H_{35}O_4Si$; 331.2299, found 331.2295.

Diisopropyl(methyl)[(tetrahydrofuran-2-yl)methoxy]silane (1a-1)

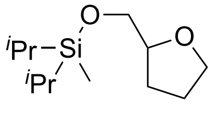

Colorless oil. **IR** (neat) 2941, 2865, 1735, 1463, 1196, 1116, 1085, 1031, 996, 881 cm^{-1}. **^{1}H NMR** (400 MHz, $CDCl_3$) δ 3.95 (m, 1H), 3.85 (m, 1H), 3.74 (m, 1H), 3.65 (dd, *J* = 10.4, 5.2 Hz, 1H), 3.57 (dd, *J* = 10.4, 5.2 Hz, 1H), 1.94–1.79 (m, 3H), 1.70 (m, 1H), 0.92–1.03 (m, 14H), 0.01 (s, 3H). **^{13}C NMR** (100 MHz, $CDCl_3$) δ 79.5, 68.3, 66.0, 27.9, 25.7, 17.4, 17.3, 13.0, 12.9, –8.6. **HRMS–ESI** (m/z):[M+Na]$^+$ calcd for $C_{12}H_{26}O_2SiNa$; 253.1594, found 253.1598.

Ethyl 3-{diisopropyl[(tetrahydrofuran-2-yl)methoxy]silyl}propanoate (3aa-1)

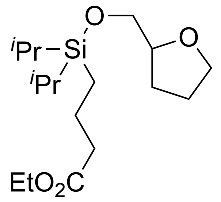

Colorless oil. (containing inseparable impurities) **IR** (neat) 2941, 2865, 1735, 1463, 1196, 1116, 1087, 1072, 996, 881 cm^{-1}. **^{1}H NMR** (400 MHz, $CDCl_3$) δ 4.12 (q, *J* = 7.2 Hz, 2H), 3.96 (m, 1H), 3.84 (m, 1H), 3.75 (m, 1H), 3.67 (dd, *J* = 10.0, 5.2 Hz, 1H), 3.59 (dd, *J* = 10.0, 5.2 Hz, 1H), 2.32 (t, *J* = 7.2 Hz, 2H), 1.92–1.81 (m, 3H), 1.76–1.65 (m, 3H), 1.25 (t, *J* = 7.2 Hz, 3H), 1.03–-0.97 (m, 14H), 0.68–0.64 (m, 2H). **^{13}C NMR** (100 MHz, $CDCl_3$) δ 173.5, 79.4, 68.3, 66.0, 60.1, 38.0, 27.8, 25.7, 19.2, 17.5, 17.4, 14.2, 12.9, 12.4, 10.3. **HRMS–ESI** (m/z):[M+Na]$^+$ calcd for $C_{17}H_{34}O_4SiNa$; 353.2119, found 353.2125.

Benzyl 3-[2-({[Diisopropyl(methyl)silyl]oxy}methyl)tetrahydrofuran-2-yl]propanoate (3ab)

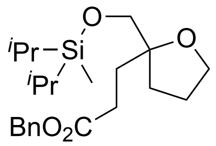

The product **3ab** was purified by flash column chromatography on silica gel (95:5–90:10, hexane/AcOEt) (24.7 mg, 0.063 mmol, 63% isolated yield). Colorless oil. **IR** (neat) 2865, 1737, 1252, 1161, 1103, 881, 821, 781, 752, 698 cm^{-1}. **^{1}H NMR** (400 MHz, $CDCl_3$) δ7.33 (m, 5H), 5.11 (s, 2H), 3.79 (ddd, *J* = 6.4, 6.4, 2.8 Hz, 1H), 3.76 (ddd, *J* = 6.4, 6.4, 2.8 Hz, 1H), 3.47 (d, *J* = 10.0 Hz, 1H), 3.40 (d, *J* = 10.0 Hz, 1H), 2.44 (ddd, *J* = 6.8, 2.8, 2.8 Hz, 1H), 2.42 (ddd, *J* = 6.8, 2.8, 2.8 Hz, 1H), 1.98–1.84 (m, 5H), 1.66 (m, 1H), 1.00–0.91(m, 14H), 0.00 (s, 3H). **^{13}C NMR** (100 MHz, $CDCl_3$) δ 173.9, 136.1, 128.5, 128.2, 128.1, 84.2, 68.5, 67.3, 66.1, 32.5, 31.5, 29.2, 26.1, 17.5, 17.4, 12.9 (2C), –8.7. **HRMS–ESI** (m/z):[M+H]$^+$ calcd for $C_{22}H_{37}O_4Si$; 393.2456, found 393.2451.

Benzyl 3-[5-({[Diisopropyl(methyl)silyl]oxy}methyl)tetrahydrofuran-2-yl]propanoate (3ab-1)

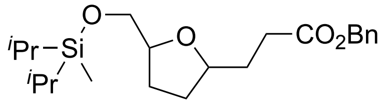

Colorless oil. **IR** (neat) 2943, 2865, 1735, 1457, 1252, 1159, 1100, 1085, 881, 376 cm^{-1}. The ratio (1.5:1) of diastereomers was determined by ^{1}H NMR analysis. **^{1}H NMR** (400 MHz, $CDCl_3$) δ 7.37–7.29 (m, 5H), 5.11 (s, 2H), 4.00 (m, 0.40 × 1H), 3.95 (m, 1H), 3.88 (m, 0.60 × 1H), 3.63 (m, 1H), 3.53 (m, 1H), 2.54–2.37 (m, 2H), 2.03–1.68 (m, 5H), 1.48 (m, 1H), 1.00–0.91 (m, 14H), 0.00 (s, 3H). **^{13}C NMR** (100 MHz, $CDCl_3$) δ 173.4, 136.0, 128.5, 128.1, 79.7, 79.1, 78.6, 78.3, 66.1, 66.0, 31.5, 31.1, 31.0, 30.9, 30.7, 30.7, 28.0, 27.8, 17.3, 17.3, 12.97, 12.94, 12.92, –6.6. (only observed peaks) **HRMS–ESI** (m/z):[M+H]$^+$ calcd for $C_{22}H_{37}O_4Si$; 393.2456, found 393.2462.

4-[2-({[Diisopropyl(methyl)silyl]oxy}methyl)tetrahydrofuran-2-yl]butan-2-one (3ac)

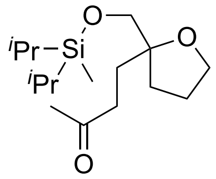

The product **3ac** was purified by flash column chromatography on silica gel (95:5–90:10, hexane/AcOEt) (18.5 mg, 0.062 mmol, 62% isolated yield). Colorless oil. **IR** (neat) 2954, 2865, 1743, 1252, 1100, 1070, 881, 823, 781, 758 cm^{-1}. **^{1}H NMR** (400 MHz, $CDCl_3$) δ 3.81–3.73 (m, 2H), 3.47 (d, J = 9.6 Hz, 1H), 3.40 (d, J = 9.6 Hz, 1H), 2.51–2.45 (m, 2H), 2.14 (s, 3H), 1.97–1.71 (m, 5H), 1.60 (m, 1H), 1.03–0.87 (m, 14H), 0.00 (s, 3H). **^{13}C NMR** (100 MHz, $CDCl_3$) δ 209.2, 84.3, 68.4, 67.4, 38.5, 32.7, 30.5, 29.8, 26.1, 17.4, 17.3, 13.0, 12.9, –8.6. **HRMS–ESI** (m/z):[M+H]$^+$ calcd for $C_{16}H_{33}O_3Si$; 301.2193, found 301.2192.

3-[2-({[Diisopropyl(methyl)silyl]oxy}methyl)tetrahydrofuran-2-yl]cyclopentan-1-one (3ad)

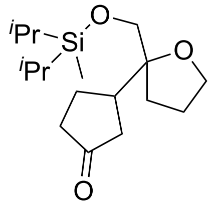

The product **3ad** was purified by flash column chromatography on silica gel (95:5–90:10, hexane/AcOEt) (21.1 mg, 0.068 mmol, 68% isolated yield). Colorless oil. **IR** (neat) 2941, 2865, 1735, 1251, 1177, 1098, 1042, 881, 820, 779 cm^{-1}. The ratio (1.3:1) of diastereomers was determined by ^{1}H NMR analysis. **^{1}H NMR** (400 MHz, $CDCl_3$) δ 3.91–3.76 (m, 2H), 3.55 (d, J = 4.8 Hz, 0.57 × 1H), 3.45–3.39 (m, 0.57 × 1H + 0.43 × 2H), 2.59 (m, 1H), 2.37–1.58 (m, 10H), 0.89–1.03 (m, 14H), 0.02 (s, 0.57 × 3H), 0.01 (s, 0.43 × 3H). **^{13}C NMR** (100 MHz, $CDCl_3$) δ 219.7, 219.2, 85.0, 84.9, 68.9, 68.6, 67.1, 66.2, 41.7, 41.5, 39.9, 39.7, 38.81, 38.76, 30.9, 29.9, 25.9, 25.7, 23.6, 23.3, 17.1 (2C), 17.0 (2C), 12.6 (2C), 12.5 (2C), –9.0(2C). **HRMS–ESI** (m/z):[M+H]$^+$ calcd for $C_{17}H_{33}O_3Si$; 313.2193, found 313.2195.

(*R*)-3-[2-({[Diisopropyl(methyl)silyl]oxy}methyl)tetrahydrofuran-2-yl]-*N*-phenylpropanamide (3ae)

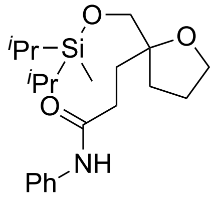

The product **3ae** was purified by flash column chromatography on silica gel (100:0–90:10, hexane/AcOEt) (21.7 mg, 0.060 mmol, 60% isolated yield). Colorless oil. **IR** (neat) 2941, 2864, 1663, 1600, 1545, 1499, 1443, 1252, 1104, 754 cm^{-1}. **^{1}H NMR** (400 MHz, $CDCl_3$) δ 8.26 (br, 1H), 7.51 (d, J = 7.6 Hz, 2H), 7.30 (t, J = 7.6 Hz, 2H), 7.07 (t, J = 7.6 Hz, 1H), 3.89 (t, J = 6.4Hz, 2H), 3.50 (d, J = 10.0 Hz, 1H), 3.46 (d, J = 10.0 Hz, 1H), 2.52–2.42 (m, 2H), 2.10–1.89 (m, 5H), 1.66 (m, 1H), 1.01–

0.91(m, 14H), 0.02 (s, 3H). **^{13}C NMR** (100 MHz, $CDCl_3$) δ 171.8, 138.4, 128.9, 123.8, 119.5, 84.9, 68.6, 67.6, 32.8, 32.6, 31.8, 26.2, 17.4, 17.3, 13.0, 12.9, –8.7. **HRMS–ESI** (m/z):$[M+H]^+$ calcd for $C_{21}H_{36}NO_3Si$; 378.2459, found 378.2460.

3-[2-({[Diisopropyl(methyl)silyl]oxy}methyl)tetrahydrofuran-2-yl]propanenitrile (3af)

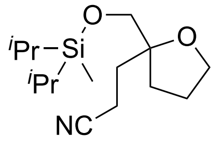

The product **3af** was purified by flash column chromatography on silica gel (95:5–90:10, hexane/AcOEt) (16.0 mg, 0.052 mmol, 52 % isolated yield). Colorless oil. IR (neat) 2943, 2865, 1252, 1104, 1085, 881, 820, 781, 756, 374 cm^{-1}. **^{1}H NMR** (400 MHz, $CDCl_3$) δ 3.83–3.80 (m, 2H), 3.45 (d, J = 10.0 Hz, 1H), 3.38 (d, J = 10.0 Hz, 1H), 2.43–2.39 (m, 2H), 2.06–1.80 (m, 5H), 1.65 (m, 1H), 1.01–0.90 (m, 14H), 0.01 (s, 3H). **^{13}C NMR** (100 MHz, $CDCl_3$) δ 120.4, 83.4, 68.4, 67.1, 32.8, 32.3, 25.9, 17.4, 17.3, 12.93, 12.90, 11.9, –8.7. **HRMS–ESI** (m/z):$[M+H]^+$ calcd for $C_{15}H_{30}NO_2Si$; 284.2040, found 284.2040.

Diisopropyl(methyl)({2-[2-(phenylsulfonyl)ethyl]tetrahydrofuran-2-yl}methoxy)silane (3ag)

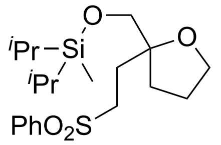

The product **3ag** was purified by flash column chromatography on silica gel (95:5–90:10, hexane/AcOEt) (19.1 mg, 0.048 mmol, 48% isolated yield). Colorless oil. **IR** (neat) 2943, 2864, 1307, 1149, 1101, 1087, 1070, 818, 689, 535 cm^{-1}. **^{1}H NMR** (400 MHz, $CDCl_3$) δ 7.90–7.88 (m, 2H), 7.64 (m, 1H), 7.58–7.53 (m, 2H), 3.78–3.69 (m, 2H), 3.37 (d, J = 9.8 Hz, 1H), 3.32 (d, J = 9.8 Hz, 1H), 3.26–3.13 (m, 2H), 2.00–1.80 (m, 5H), 1.59 (m, 1H), 0.99–0.82 (m, 14H), –0.05 (s, 3H). **^{13}C NMR** (100 MHz, $CDCl_3$) δ 139.0, 133.5, 129.2, 128.1, 128.0, 83.4, 68.3, 67.4, 52.0, 33.1, 29.5, 25.8, 17.4, 17.3, 12.8, -8.7. **HRMS–ESI** (m/z):$[M+H]^+$ calcd for $C_{20}H_{35}O_4SSi$; 399.2020, found 399.2022.

Diethyl {2-[2-({[Diisopropyl(methyl)silyl]oxy}methyl)tetrahydrofuran-2-yl]ethyl}phosphonate (3ah)

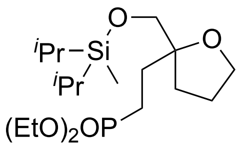

The product **3ah** was purified by flash column chromatography on silica gel (34:66, hexane/AcOEt) (25.1 mg, 0.063 mmol, 63%). **IR** (neat) 2943, 2865, 1251, 1098, 1060, 1031, 962, 881, 820, 784 cm^{-1}. **^{1}H NMR** (400 MHz, $CDCl_3$) δ 4.13–4.01 (m, 4H), 3.80 (t, J = 6.4 Hz, 2H), 3.45 (d, J = 9.6 Hz, 1H), 3.39 (d, J = 9.6 Hz, 1H), 1.97–1.69 (m, 7H), 1.61 (m, 1H), 1.31 (t, J = 7.2 Hz, 6H), 1.00–0.89 (m, 14H), 0.00 (s, 3H). **^{13}C NMR** (100 MHz, $CDCl_3$) δ 84.3, 84.1, 68.4, 67.3, 61.5,

61.4, 61.3, 32.5, 26.1, 17.42, 17.38, 17.33, 16.5, 16.4, 12.9, –8.7. **HRMS–ESI** (m/z):[M+H]$^+$ calcd for $C_{18}H_{40}O_5PSi$; 395.2377, found 395.2387.

4-{2-[2-({[Diisopropyl(methyl)silyl]oxy}methyl)tetrahydrofuran-2-yl]ethyl}pyridine (3ai)

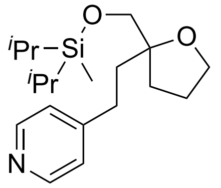

The product **3ai** was purified by flash column chromatography on silica gel (70:30, hexane/AcOEt) (16.2 mg, 0.048 mmol, 48%). **IR** (neat) 2943, 2865, 1601, 1462, 1252, 1104, 1070, 993, 881, 821 cm^{-1}. **^{1}H NMR** (400 MHz, $CDCl_3$) δ 8.46 (d, J = 6.0 Hz, 2H), 7.12 (m, 2H), 3.85 (t, J = 6.4 Hz, 2H), 3.54 (d, J = 9.8 Hz, 1H), 3.45 (d, J = 9.8 Hz, 1H), 2.66 (t, J = 8.8 Hz, 2H), 1.70–2.00 (m, 6H), 1.02–0.92 (m, 14H), 0.02 (s, 3H). **^{13}C NMR** (100 MHz, CD_3Cl) δ 152.0, 149.6, 123.8, 84.5, 68.4, 67.4, 37.5, 32.7, 29.6, 26.1, 17.4 (2C), 17.3, 12.9, –8.6. **HRMS–ESI** (m/z):[M+H]$^+$ calcd for $C_{19}H_{34}NO_2Si$; 336.2353, found 336.2329.

[(2-Allyltetrahydrofuran-2-yl)methoxy]diisopropyl(methyl)silane (3aj)

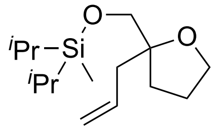

The product **3aj** was purified by flash column chromatography on silica gel (100:0–97:3, hexane/AcOEt) (8.5 mg, 0.031 mmol, 31%). **IR** (neat) 2943, 2865, 1457, 1252, 1103, 1062, 881, 821, 779, 394 cm^{-1}. **^{1}H NMR** (400 MHz, $CDCl_3$) δ 5.83 (m, 1H), 5.09 (m, 1H), 5.05 (m, 1H), 3.81 (t, J = 6.4 Hz, 2H), 3.47 (d, J = 9.6 Hz, 1H), 3.42 (d, J = 9.6 Hz, 1H), 2.35–2.22 (m, 2H), 1.91–1.82 (m, 3H), 1.67 (m, 1H), 1.01–0.92 (m, 14H), 0.00 (s, 3H). **^{13}C NMR** (100 MHz, $CDCl_3$ δ 134.6, 117.4, 84.7, 68.5, 67.9, 41.3, 31.6, 26.2, 17.4, 17.3, 13.02, 13.01, –8.6. **HRMS–ESI** (m/z):[M+Na]$^+$ calcd for $C_{15}H_{30}O_2SiNa$; 29311907, found 293.1910.

Ethyl 3-[2-({[Diisopropyl(methyl)silyl]oxy}methyl)tetrahydro-2*H*-pyran-2-yl]propanoate (3ba)

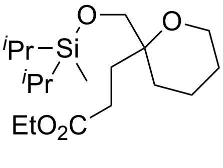

The product **3ba** was purified by flash column chromatography on silica gel (95:5–90:10, hexane/AcOEt) (25.6 mg, 0.074 mmol, 74 % isolated yield). **IR** (neat) 2940, 2865, 1737, 1252, 1180, 1093, 1047, 881, 820, 781 cm^{-1}. **^{1}H NMR** (400 MHz, $CDCl_3$) δ 4.12 (q, J = 7.2 Hz, 2H), 3.62–3.59 (m, 2H), 3.49 (d, J = 9.8 Hz, 1H), 3.47 (d, J = 9.8 Hz, 1H), 2.39–2.25 (m, 2H), 2.11 (m, 1H), 1.80 (m, 1H), 1.67–1.46 (m, 6H), 1.24 (t, J = 7.2 Hz, 3H), 1.02–0.87 (m, 14H), 0.01 (s, 3H). **^{13}C NMR** (100 MHz, $CDCl_3$) δ 174.4, 78.4, 74.3, 66.8, 61.6, 60.2, 31.4, 28.0, 27.0, 25.8, 18.8, 17.4, 17.3, 14.2, 12.9, –8.7. **HRMS–ESI** (m/z):[M+H]$^+$ calcd for $C_{18}H_{37}O_4Si$; 345.2456, found 345.2452.

Ethyl 3-((3*R*,6*R*,6*R*)-6-((*tert*-Butyldimethylsilyl)oxy)-5-(((diisopropyl(methyl)silyl)oxy)methyl)-2,2-dimethyltetrahydrofuro[2,3-d][1,3]dioxol-5-yl)propanoate (3ca)

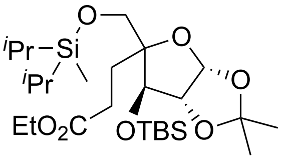

The product **3ca** was purified by flash column chromatography on silica gel (100:0–95:5, hexane/AcOEt) (26.3 mg, 0.049 mmol, 49% isolated yield). **IR** (neat) 2938, 1739, 1253, 1164, 1059, 1067, 1008, 880, 838, 778 cm^{-1}. The ratio (2.7:1) of diastereomers was determined by ^{1}H NMR analysis. **^{1}H NMR** (400 MHz, $CDCl_3$) δ 5.92 (d, *J* = 4.8 Hz, 0.27 × 1H), 5.82 (d, *J* = 4.4 Hz, 0.73 × 1H), 4.56 (dd, *J* = 4.8, 2.8 Hz, 0.27 × 1H), 4.43 (d, *J* = 2.4 Hz, 0.73 × 1H), 4.35 (s, 0.73 × 1H), 4.14–4.08 (m, 2H), 4.02 (d, *J* = 2.8 Hz, 0.27 × 1H), 3.80 (d, *J* = 10.0 Hz, 0.73 × 1H), 3.65 (d, *J* = 10.2 Hz, 0.27 × 1H), 3.52 (m, 1H), 2.52–2.30 (m, 2H), 2.11–1.82 (m, 2H), 1.53 (s, 0.27 × 3H), 1.51 (s, 0.73 × 3H), 1.33 (s, 0.27 × 3H), 1.28 (s, 0.73 × 3H), 1.25–1.21 (m, 3H), 1.02–0.96 (m, 14H), 0.91–0.90 (m, 9H), 0.14–0.13 (m, 3H), 0.10 (s, 3H), 0.02–0.01 (m, 3H). **^{13}C NMR** (100 MHz, $CDCl_3$) δ 174.0, 173.5, 112.6, 111.7, 104.6, 104.5, 90.2, 88.8, 88.5, 81.4, 77.9, 77.2, 64.7, 64.4, 62.2, 60.3, 60.1, 29.3, 28.9, 28.9, 27.5, 27.3, 27.0, 26.7, 25.9, 25.77, 25.75, 25.6, 18.0, 17.9, 17.5, 17.44, 17.36, 17.29, 14.2, 13.1, 13.0, 12.7, -4.5, -4.8, -5.1, -8.5. (only observed peaks) **HRMS–ESI** (m/z):[M+H]$^{+}$ calcd for $C_{26}H_{53}O_7Si_2$; 533.3324, found 533.3329.

Ethyl 3-[(2*R*,3*R*,5*R*)-5-[3-Benzyl-5-methyl-2,4-dioxo-3,4-dihydropyrimidin-1(2*H*)-yl]-3-[(*tert*-butyldimethylsilyl)oxy]-2-({[diisopropyl(methyl)silyl]oxy}methyl)tetrahydrofuran-2-yl]propanoate (3da)

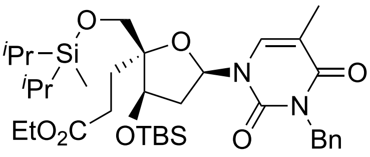

The product **3da** was purified by flash column chromatography on silica gel (90:10–80:20, hexane/AcOEt) (41.7 mg, 0.063 mmol, 63% isolated yield). **IR** (neat) 1735, 1702, 1667, 1644, 1463, 1449, 1253, 1097, 1077, 779 cm^{-1}. The stereochemistry was determined by NOESY. **^{1}H NMR** (400 MHz, $CDCl_3$) δ 7.59 (d, *J* = 1.2 Hz, 1H), 7.45 (dd, *J* = 8.2, 1.2 Hz, 2H), 7.30–7.21 (m, 3H), 6.12 (dd, *J* = 7.6, 2.8 Hz, 1H), 5.10 (dd, *J* = 16.0, 13.6 Hz, 2H), 4.12 (q, *J* = 7.2 Hz, 2H), 4.08 (dd, *J* = 5.2, 2.4 Hz, 1H), 3.90 (d, *J* = 10.4 Hz, 1H), 3.78 (d, *J* = 10.4 Hz, 1H), 2.82 (ddd, *J* = 13.2, 7.6, 5.2 Hz, 1H), 2.44 (m, 1H), 2.33 (m, 1H), 1.97–1.89 (m, 5H), 1.79 (m, 1H), 1.24 (t, *J* = 7.2 Hz, 3H), 1.26–0.92 (m, 14H), 0.84 (s, 9H), 0.06 (s, 3H), 0.05 (s, 3H), 0.00 (s, 3H). **^{13}C NMR** (100 MHz, $CDCl_3$) δ 173.2, 163.5, 150.9, 137.0, 134.6, 129.0, 128.3, 127.4, 109.3, 89.9, 84.9, 74.3, 62.7, 60.6, 44.3, 41.6, 28.8, 28.2, 25.6, 17.9, 17.5, 17.44, 17.36, 17.29, 14.2, 13.3, 13.2, 12.8, –4.9, –5.1, –8.6. **HRMS–ESI** (m/z):[M+H]$^{+}$ calcd for $C_{35}H_{59}N_2O_7Si_2$; 675.3855, found 675.3866.

Ethyl 3-[(2*R*,3*R*,5*R*)-5-(6-benzamido-9*H*-purin-9-yl)-3-hydroxy-2-(hydroxymethyl)tetrahydro furan-2-yl]propanoate (3fa)

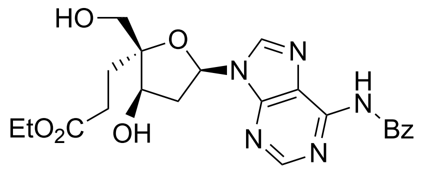

The product **3fa** was purified by flash column chromatography on silica gel (100:0–50:50, hexane/AcOEt) (57.7 mg, including inseparable impurities). To the solution of obtained mixture in THF (1.0 mL), TBAF (1.0 mL, 1.0 M in tetrahydrofuran) was added at room temperature, and the mixture was stirred for 1 h. After removed the solvent under reduced pressure, the crude product was purified by reverse phase column chromatography on Sfär C18 D –Duo 100 Å 30 μm 30 g (100:0–70:30, H_2O/MeCN) to give **3fa** as white amorphous solid (16.4 mg, 0.043 mmol, 43% isolated yield over 2 steps). **IR** 1700, 1613, 1582, 1456, 1298, 1253, 1084, 798, 755, 709 cm^{-1}. The stereochemistry was determined by NOESY. **^{1}H NMR** (400 MHz, $CDCl_3$) δ 9.07 (s, 1H), 8.80 (s, 1H), 8.10 (s, 1H), 8.08–8.02 (m, 2H), 7.63 (m, 1H), 7.56–7.52 (m, 2H), 6.13 (dd, *J* = 7.8, 5.6 Hz, 1H), 5.94 (d, *J* = 10.0 Hz, 1H), 4.48 (d, *J* = 6.8 Hz, 1H), 4.36 (ddd, *J* = 7.2, 6.8, 4.8 Hz, 1H), 4.17–4.11 (m, 2H), 3.90 (d, *J* = 11.6, 1H), 3.71 (dd, *J* = 11.6, 10.0, 1H), 3.06 (ddd, *J* = 14.8, 7.8, 7.2 Hz, 1H), 2.76 (ddd, *J* = 14.8, 5.6, 4.8 Hz, 1H), 2.51–2.35 (m, 2H), 2.04 (m, 1H), 1.90 (m, 1H), 1.25 (t, *J* = 7.2 Hz, 3H). **^{13}C NMR** (100 MHz, $CDCl_3$) δ 173.6, 164.4, 151.8, 150.3, 150.0, 142.9, 133.3, 133.0, 128.9, 127.8, 124.4, 88.7, 84.6, 76.2, 64.0, 60.7, 40.3, 29.2, 28.5, 14.1. **HRMS–ESI** (m/z):$[M+H]^+$ calcd for $C_{22}H_{26}N_5O_6$; 456.1878, found 456.1888.

■ Mechanistic Experiments ■

A. Reaction with D_2O

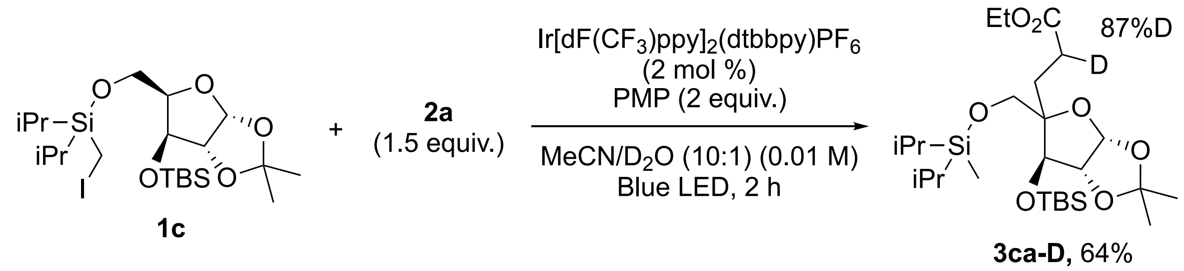

Supplementary Fig. 7 Reaction with D_2O

In a glovebox, to an oven-dried vial with a stirring bar and Ir[dF(CF_3)ppy]$_2$(dtbbpy)PF_6 (2.2 mg, 0.002 mmol) was added C5' tethered alcohol **1c** (27.9 mg, 0.05 mmol) in MeCN(1 mL). Then, MeCN (4 mL), degassed D_2O (0.5 mL), PMP (15.5 mg, 0.1 mmol), and ethyl acrylate (7.5 mg, 0.08 mmol) were added to the vial. After sealing the vial with a cap and removed from the glovebox, the reaction was stirred and irradiated with 45W blue in EvoluChem photoreactor (PhotoRedOx Duo) (Supplementary Fig. 5). After 2 h, the solvent was removed under reduced pressure. The crude product was then purified by flash column chromatography on silica gel (100:0–95:5–90:10, hexane/AcOEt) to give the alkylated product **3ca-D** (32.8 mg, 0.032 mmol, 64% isolated yield) as colorless oil.

Ethyl 3-((3*R*,6*R*,6*R*)-6-[(*tert*-butyldimethylsilyl)oxy]-5-{[(diisopropyl(methyl)silyloxy]methyl}-2,2-dimethyltetrahydrofuro[2,3-d][1,3]dioxol-5-yl)propanoate-2-d (3ca-D)

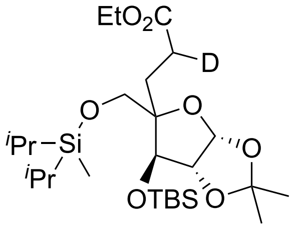

Colorless oil. **IR** (neat) 2940, 2865, 1735, 1253, 1098, 1071, 1006, 880, 838, 778 cm^{-1}. The ration of deuterium incorporation (87%D) was determined by ^{1}H NMR analysis. **^{1}H NMR** (400 MHz, $CDCl_3$) δ 5.92 (d, J = 4.8 Hz, 0.40 × 1H), 5.82 (d, J = 4.4 Hz, 0.60 × 1H), 4.56 (dd, J = 4.8, 2.8 Hz, 0.40 × 1H), 4.43 (d, J = 4.4 Hz, 0.60 × 1H), 4.35 (s, 0.60 × 1H), 4.14–4.08 (m, 2H), 4.02 (d, J = 2.8 Hz, 0.40 × 1H), 3.80 (d, J = 10.0 Hz, 0.60 × 1H), 3.65 (d, J = 10.0 Hz, 0.40 × 1H), 3.52 (m, 1H), 2.52–2.30 (m, 1.13H), 2.11–1.82 (m, 2H), 1.53 (s, 0.40 × 3H), 1.51 (s, 0.60 × 3H), 1.33 (s, 0.40 × 3H), 1.28 (s, 0.60 × 3H), 1.25–1.21 (m, 3H), 1.02–0.96 (m, 14H), 0.91–0.90 (m, 9H), 0.14–0.13 (m, 3H), 0.10 (s, 3H), 0.02–0.01 (m, 3H). **^{13}C NMR** (100 MHz, $CDCl_3$) δ 174.0, 173.5, 112.6, 111.6, 104.6, 104.5, 90.2, 88.8, 88.5, 88.4, 81.3, 77.8, 64.7, 64.4, 60.3, 60.1, 29.2, 27.5, 27.3, 26.9, 26.7, 25.9, 25.77, 25.75, 18.0, 17.9, 17.5, 17.4, 17.3, 17.2, 14.1, 13.1, 12.9, 12.7. (only observed peaks) **HRMS–ESI** (m/z):[M+H]$^+$ calcd for $C_{26}H_{51}DO_7Si_2Na$; 556.3207, found 556.3215.

B. Reaction in the presence of TEMPO

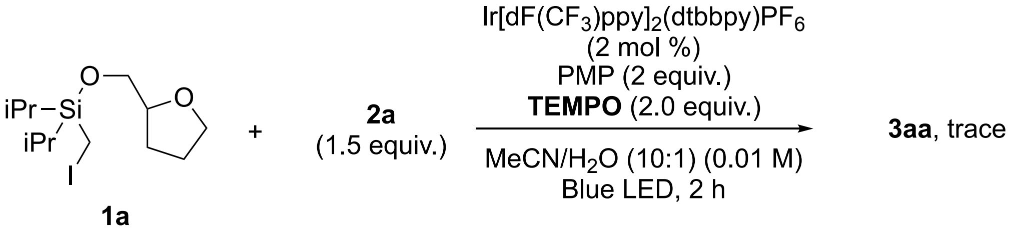

Supplementary Fig. 8 Reaction in the presence of TEMPO

In a glovebox, to an oven-dried vial with a stirring bar, Ir[dF(CF_3)ppy]$_2$(dtbbpy)PF_6 (2.2 mg, 0.002 mmol) and TEMPO (31.3 mg, 0.2 mmol) was added (iodomethyl)diisopropyl[(tetrahydrofuran-2-yl)methoxy]silane **1a** (35.6 mg, 0.1 mmol) in MeCN(1 mL). Then, MeCN (9 mL), degassed H_2O (1 mL), PMP (31 mg, 0.2 mmol) and ethyl acrylate (15 mg, 0.15 mmol) were added to the vial. After sealing the vial with a cap and removed from the glovebox, the reaction was stirred and irradiated with 45W blue in EvoluChem photoreactor (PhotoRedOx Duo) (Supplementary Fig. 5). After 2 h, the solvent was removed under reduced pressure.

■ Directing group at 3'-*O*-position ■

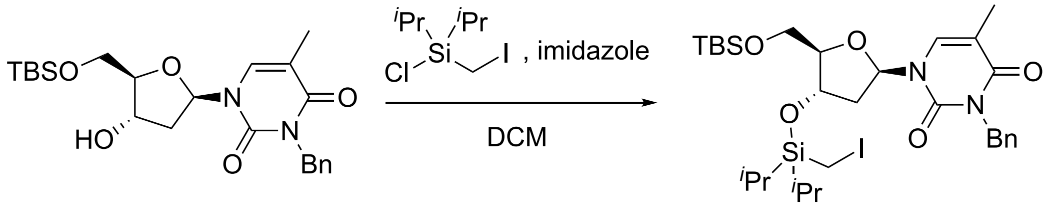

S4-1 **1g**

Supplementary Fig. 9 Synthesis of **1g**

Synhtesis of 1g. To a solution of (chloro)diisopropyliodomethylsilane (280 mg, 1.1 mmol) and imidazole (198 mg, 2.9 mmol) in DCM (5 mL), thymidine **S4-1** (434 mg, 1.0 mmol) was added. The reaction mixture was stirred overnight at room temperature under nitrogen atmosphere. After the solvent was removed under reduced pressure, purification by flash column chromatography on silica gel (Biotage Selekt, 100:0–95:5, hexane/AcOEt) gave the C3' tethered thymidine **1g** as a white amorphous solid (476 mg, 0.68 mmol, 70%).

(*S*)-1-Benzyl-3-[(2*R*,4*S*,5*R*)-5-{|[(*tert*-butyldimethylsilyl)oxy]methyl}-4-{[(iodomethyl)diisopropylsilyl]oxy}tetrahydrofuran-2-yl]-5-methylpyridine-2,6(1*H*,3*H*)-dione (1g)

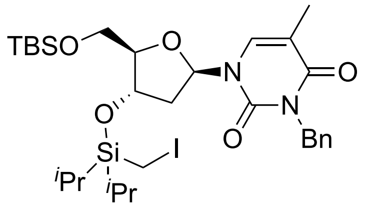

IR (neat) 1667, 1253, 1105, 1067, 1029, 881, 831, 777, 765, 698 cm^{-1}. **^{1}H NMR** (400 MHz, $CDCl_3$) δ 7.48–7.46 (m, 3H), 7.31–7.23 (m, 3H), 6.41 (dd, J = 8.8, 5.6 Hz, 1H), 5.15 (d, J = 13.6 Hz, 1H), 5.09 (d, J = 13.6 Hz, 1H), 4.62 (m, 1H), 4.05 (dd, J = 4.0, 2.4 Hz, 1H), 3.86 (d, J = 2.4 Hz, 2H), 2.32 (ddd, J = 13.2, 5.6, 1.6 Hz, 1H), 2.06 (s, 2H), 2.03 (m, 1H), 1.93 (d, J = 1.2 Hz, 3H), 1.21–1.28 (m, 2H), 1.09–1.05 (m, 12H), 0.09 (s, 9H), 0.11 (s, 6H). **^{13}C NMR** (100 MHz, $CDCl_3$) δ 163.3, 150.9, 136.8, 133.5, 129.0, 128.2, 127.4, 110.1, 87.9, 85.4, 73.6, 63.3, 44.4, 41.7, 25.8, 18.2, 17.5, 17.4, 17.3, 17.2, 13.2, 12.1, –5.3, –5.4. **HRMS–ESI** (m/z):$[M+H]^+$ calcd for $C_{30}H_{50}IN_2O_5Si_2$; 701.2298, found 701.2313.

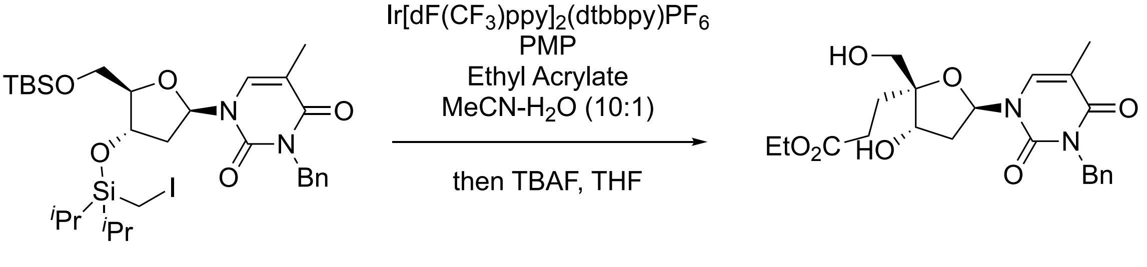

1g **3ga**

Supplementary Fig. 10 Synthesis of **3ga**

In a glovebox, to an oven-dried vial with a stirring bar and Ir[dF(CF_3)ppy]$_2$(dtbbpy)PF_6 (1.1 mg, 0.001 mmol) was added C3' tethered thymidine **1g** (35.0 mg, 0.05 mmol) in MeCN(1 mL). Then, MeCN (4 mL), degassed H_2O (0.5 mL), PMP (18.0 mg, 0.1 mmol), and ethyl acrylate (7.5 mg, 0.08 mmol) were added to the vial. After sealing the vial with a cap and removed from the glovebox, the reaction was stirred and irradiated with 45W blue in EvoluChem photoreactor (PhotoRedOx Duo) (Supplementary Fig. 5). After 4 h, the solvent was removed under reduced pressure. The residue was dissolved in THF (1 mL) and 1 M TBAF in tetrahydrofuran (100 µl, 0.1 mmol) was added and stirred overnight. After removed the solvent, the crude product was purified by flash column chromatography on silica gel (100:0–95:5, hexane/AcOEt) followed by GPC and reverse phase column chromatography on Sfär C18 D –Duo 100 Å 30 µm 30 g (100:0–50:50, H_2O/MeCN) to give the alkylated product **3ga** (5.0 mg, 0.012 mmol, 23% isolated yield) as colorless oil.

Ethyl 3-{(2*R*,3*S*,5*R*)-5-[3-benzyl-5-methyl-2,4-dioxo-3,4-dihydropyrimidin-1(2*H*)-yl]-3-hydroxy-2-(hydroxymethyl)tetrahydrofuran-2-yl}propanoate (3ga)

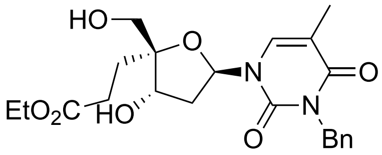

Colorless oil. **IR** (neat) 1700, 1664, 1635, 1470, 1453, 1271, 1197, 1061, 769, 700 cm^{-1}. The stereochemistry was determined by NOESY. **^{1}H NMR** (400 MHz, $CDCl_3$) δ 7.48–7.46 (m, 2H), 7.31–7.22 (m, 4H), 6.10 (dd, *J* = 6.8, 6.8 Hz, 1H), 5.10 (dd, *J* = 16.0, 13.6 Hz, 2H), 4.58 (dd, *J* = 6.8, 4.8 Hz, 1H), 4.15 (q, *J* = 7.2 Hz, 2H), 3.60 (s, 2H), 2.33–2.51 (m, 4H), 2.08–1.95 (m, 5H), 1.25 (t, *J* = 7.2 Hz, 3H). **^{13}C NMR** (100 MHz, $CDCl_3$) δ 174.7, 163.2, 150.9, 136.7, 134.6, 129.2, 128.3, 127.6, 110.5, 87.7, 86.3, 73.2, 65.3, 60.9, 44.4, 39.4, 28.5, 24.8, 14.1, 13.5. **HRMS–ESI** (m/z):$[M+H]^+$ calcd for $C_{22}H_{29}N_2O_7$; 433.1969, found 433.1981.

■ (*E*)-Oxime Imidate as a Directing Group ■

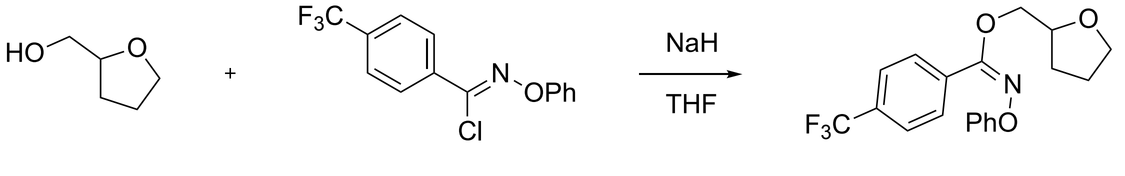

S5-1

Supplementary Fig. 11 Synthesis of **S5-1**

Synthesis of S5-1. To a solution of tetrahydrofurfuryl alcohol (29.0 mg, 0.30 mmol) in THF (1 mL), NaH (13.6 mg, 0.34 mmol) was added at 0°C, and the mixture was stirred for 15 min. After addition of (*Z*)-*N*-phenoxy-4-nitrobenzimidoyl chloride[7] (56.9 mg, 0.19 mmol), the reaction mixture was stirred overnight at room temperature. After removed the solvent in vacuo, the crude residue was purified by flash column chromatography on silica gel (Biotage Selekt, 100:0–95:5, hexane/AcOEt) gave the compound **S5-1** a colorless oil (58.2 mg, 0.16 mmol, 84%).

(Tetrahydrofuran-2-yl)methyl (*E*)-*N*-Phenoxy-4-(trifluoromethyl)benzimidate (S5-1)

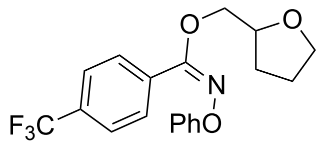

Colorless oil. **IR** (neat) 1315, 1210, 1160, 1121, 1107, 1084, 1065, 1016, 935, 850 cm^{-1}. **^{1}H NMR** (400 MHz, $CDCl_3$) δ 8.03 (d, *J* = 8.4 Hz, 2H), 7.65 (d, *J* = 8.4 Hz, 2H), 7.36–7.32 (m, 2H), 7.28–7.25 (m, 2H), 7.06 (t, *J* = 7.2 Hz, 1H), 4.54 (dd, *J* = 10.8, 3.6 Hz, 1H), 4.45 (dd, *J* = 10.8, 6.8 Hz, 1H), 4.27 (ddd, *J* = 13.6, 6.8, 3.6 Hz, 1H), 3.93–3.83 (m, 2H), 2.07 (m, 1H), 1.99–1.89 (m, 2H), 1.76 (m, 1H). **^{13}C NMR** (100 MHz, $CDCl_3$) δ 159.0, 155.3, 134.5, 132.1 (q, $J_{C\text{-}F}$ = 32.6 Hz), 129.3, 127.7, 125.3 (q, $J_{C\text{-}F}$ = 267.2 Hz), 122.5, 119.8, 114.4, 77.5, 75.0, 68.5, 27.7, 25.9. **^{19}F NMR** (376.5 MHz, $CDCl_3$): δ –62.8. **HRMS–ESI** (m/z):$[M+H]^+$ calcd for $C_{19}H_{19}F_3NO_3$; 366.1312, found 366.1313.

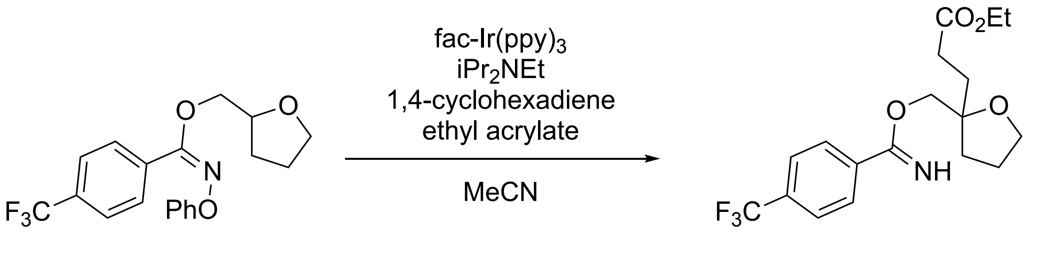

not detected

In a glovebox, to an oven-dried vial with a stirring bar, fac-Ir(ppy)$_3$ (1.3 mg, 0.002 mmol) was added **S5-1** (35.6 mg, 0.1 mmol) in MeCN (1 mL). Then, MeCN (9 mL), ^{i}Pr$_2$NEt (62.5 mg, 0.5 mmol), 1,4-cyclohexadiene (16.3 mg, 0.2 mmol) and ethyl acrylate (15 mg, 0.15 mmol). After sealing the vial with a cap and removed from the glovebox, the reaction was stirred and irradiated with 45W blue in EvoluChem photoreactor (PhotoRedOx Duo) (Supplementary Fig. 5). After 16 h, the solvent was removed under reduced pressure.

■ Reevaluation of Non-Epimerized Substrate ■

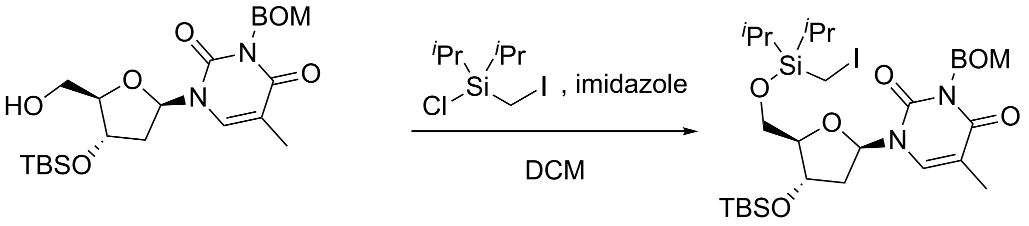

S6-1 **1h**

Supplementary Fig. 12 Synthesis of **1h**

Synthesis of 1h. Thymidine **S6-1** was prepared according to the literature.[8] To a solution of (chloro)diisopropyliodomethylsilane (160 mg, 0.55 mmol) and imidazole (102 mg, 1.5 mmol) in DCM (5 mL), thymidine **S6-1** (238 mg, 0.5 mmol) was added. The reaction mixture was stirred overnight at room temperature under nitrogen atmosphere. After the solvent was removed under reduced pressure, purification by flash column chromatography on silica gel (Biotage Selekt, 100:0–90:10, hexane/AcOEt) gave the non-epimerized thymidine **1h** as a white amorphous solid (239 mg, 0.33 mmol, 66%).

3-{(Benzyloxy)methyl}-1-{(2*R*,4*S*,5*R*)-4-{(*tert*-butyldimethylsilyl)oxy}-5-([{(iodomethyl)diisopropylsilyl}oxy]methyl)tetrahydrofuran-2-yl}-5-methylpyrimidine-2,4(1*H*,3*H*)-dione (1h)

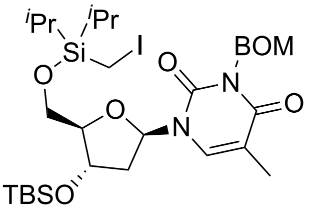

Amorphous solid. **IR** (neat) 696, 731, 772, 833, 881, 1026, 1251, 1463, 1660, 1709 cm^{-1}. **^{1}H NMR** (400 MHz, $CDCl_3$) δ 7.41–7.37 (m, 3H), 7.33–7.29 (m, 2H), 7.25 (m, 1H), 6.35 (dd, *J* = 8.0, 5.6 Hz, 1H), 5.50 (s, 2H), 4.70 (s, 2H), 4.55–4.53 (ddd, *J* = 8.0, 2.8, 2.0 Hz, 1H), 4.00–3.90 (m, 3H), 2.26 (m, 1H), 2.24 (s, 2H), 2.03 (m, 1H), 1.92 (s, 3H), 1.31–1.21 (m, 2H), 1.12–1.08 (m, 12H), 0.90 (s, 9H), 0.09 (s, 3H), 0.09 (s, 3H). **^{13}C NMR** (100 MHz, $CDCl_3$) δ 163.4, 150.8, 137.9, 134.0, 128.1, 127.6, 127.1, 110.0, 87.5, 85.4, 72.1, 72.0, 70.4, 63.3, 41.2, 25.6, 17.9 (2C), 17.58, 17.53, 17.45, 17.42, 13.1, 12.2, 12.1, –4.6, –4.7. **HRMS–ESI** (m/z):[M+H]$^+$ calcd for $C_{31}H_{52}IN_2O_6Si_2$; 731.2403, found 731.2402.

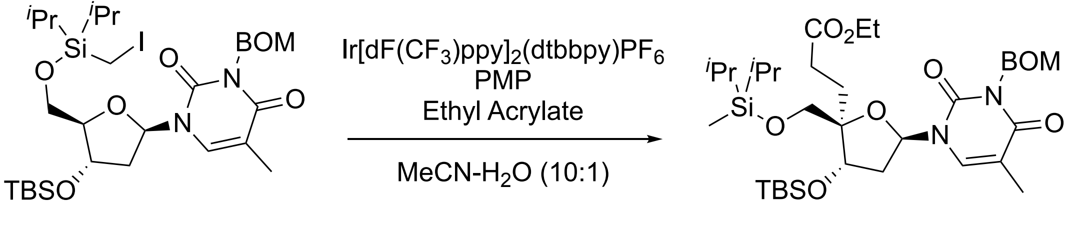

1h **3ha**

In a glovebox, to an oven-dried vial with a stirring bar and Ir[dF(CF_3)ppy]$_2$(dtbbpy)PF_6 (1.13 mg, 0.001 mmol) was added C5' tethered alcohol **1h** (27.9 mg, 0.05 mmol) in MeCN(1 mL). Then, MeCN (4 mL), degassed H_2O (0.5 mL), PMP (15.5 mg, 0.1 mmol), and ethyl acrylate (7.5 mg, 0.08 mmol) were added to the vial. After sealing the vial with a cap and removed from the glovebox, the reaction was stirred and irradiated with 45W blue in EvoluChem photoreactor (PhotoRedOx Duo) (Supplementary Fig. 5). After 2 h, the solvent was removed under reduced pressure. The crude product was then purified by flash column chromatography on silica gel (80:20, hexane/AcOEt) to give the alkylated product **3ha** (1.8 mg, 0.0025 mmol, 5% isolated yield), **3h-1** (4.5 mg, 0.0075 mmol, 15% isolated yield), **3ha-1** (9.7mg, 0.014 mmol, 28% isolated yield), **3ha-2** (8.7 mg, 0.013 mmol, 25% isolated yield) as colorless oil.

3-{(Benzyloxy)methyl}-1-[(2*R*,4*S*,5*R*)-4-{(*tert*-butyldimethylsilyl)oxy}-5-([{diisopropyl(methyl) silyl}oxy]methyl)tetrahydrofuran-2-yl}-5-methylpyrimidine-2,4(1*H*,3*H*)-dione (3h-1)

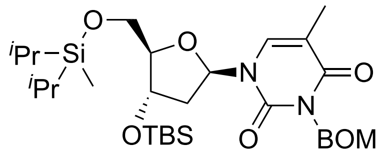

Colorless oil. **IR** (neat) 774, 835, 1028, 1074, 1095, 1253, 1463, 1667, 1712, 2953 cm^{-1}. **^{1}H NMR** (400 MHz, $CDCl_3$) δ 7.46 (d, *J* = 1.2 Hz, 1H), 7.38–7.36 (m, 2H), 7.33–7.29 (m, 2H), 7.24 (m, 1H), 6.34 (dd, *J* = 5.2, 2.4 Hz, 1H), 5.49 (s, 2H), 4.70 (s, 2H), 4.41 (ddd, *J* = 5.6, 2.8, 2.4 Hz, 1H), 3.92 (dd, *J* = 5.2, 2.4 Hz, 1H), 3.88 (dd, *J* = 11.2, 2.4 Hz, 1H), 3.77 (dd, *J* = 11.2, 2.4 Hz, 1H), 2.25 (m, 1H), 1.98 (m, 1H), 1.91 (s, 3H), 1.04–1.01 (m, 14H), 0.89 (s, 9H), 0.07–0.06 (m, 9H). **^{13}C NMR** (100 MHz, $CDCl_3$) δ 163.5, 150.9, 137.9, 134.3, 128.2, 127.6, 127.5, 110.0, 87.3, 85.4, 72.1, 72.0, 70.4, 62.9, 41.4, 25.7, 17.9, 17.4, 17.3, 13.1, 12.9, 12.6, 4.6, –4.8, –8.5. **HRMS–ESI** (m/z):[M+H]$^+$ calcd for $C_{31}H_{53}N_2O_6Si_2$; 605.3437, found 605.3438.

Ethyl 3-((3*S*,5*R*)-5-[3-{(Benzyloxy)methyl}-5-methyl-2,4-dioxo-3,4-dihydropyrimidin-1(2*H*)-yl]-3-{(*tert*-butyldimethylsilyl)oxy}-2-([{diisopropyl(methyl)silyl}oxy]methyl)tetrahydrofuran-2-yl) propanoate (3ha)

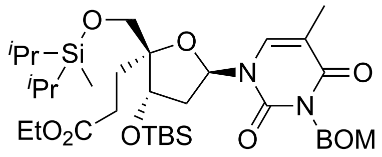

Colorless oil. The sample contains inseparable impurities. **IR** (neat) 1465, 1506, 1559, 1670, 1717, 1735, 2361, 2928, 2955, 3853 cm^{-1}. The stereochemistry was determined by NOESY. **^{1}H NMR** (400 MHz, $CDCl_3$) δ 7.46 (d, *J* = 1.2 Hz, 1H), 7.38–7.37 (m, 2H), 7.33–7.29 (m, 2H), 7.26 (m, 1H), 6.24 (dd, *J* = 7.6, 6.0 Hz, 1H), 5.49 (s, 2H), 4.70 (s, 2H), 4.47 (dd, *J* = 6.4, 3.2 Hz, 1H), 4.14–4.08 (m, 2H), 3.69 (d, *J* = 10.8 Hz, 1H), 3.55 (d, *J* = 10.8 Hz, 1H), 2.51 (m, 1H), 2.43–2.26 (m, 2H), 2.17–1.98 (m, 2H), 1.93–1.92 (m, 3H), 1.72 (m, 1H), 1.23 (t, *J* = 7.2 Hz, 3H), 1.06–1.00 (m, 14H), 0.91–0.89 (m, 9H), 0.08–0.06 (m, 9H). **^{13}C NMR** (100 MHz, $CDCl_3$) δ 173.7, 163.5, 150.9, 137.9, 134.2, 128.2, 127.6, 127.5, 109.9, 88.5, 84.5, 73.3, 72.1, 70.4, 66.4, 60.3, 41.6, 29.1, 27.2, 25.6, 17.9, 17.45, 17.41,

17.3 (2C), 14.1, 13.2, 13.0, 12.6, –4.6, –5.1, –8.5. **HRMS–ESI** (m/z):[M+H]$^+$ calcd for $C_{36}H_{61}N_2O_8Si_2$; 705.3961, found 705.3956.

Ethyl 4-[{((2*R*,3*S*,5*R*)-5-[3-{(benzyloxy)methyl}-5-methyl-2,4-dioxo-3,4-dihydropyrimidin-1(2*H*)-yl]-3-{(*tert*-butyldimethylsilyl)oxy}tetrahydrofuran-2-yl)methoxy}diisopropylsilyl]butanoate (3ha-1)

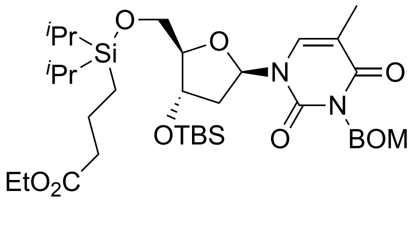

Colorless oil. The sample contains inseparable impurities. **IR** (neat) 774, 835, 1028, 1097, 1196, 1465, 1669, 1713, 1733, 2951 cm^{-1}. **^{1}H NMR** (400 MHz, $CDCl_3$) δ 7.40–7.38 (m, 3H), 7.33–7.30 (m, 2H), 7.26–7.24 (m, 1H), 6.34 (dd, *J* = 7.6, 5.6 Hz, 1H), 5.49 (s, 2H), 4.70 (s, 2H), 4.44 (ddd, *J* = 8.4, 3.2, 2,8 Hz, 1H), 4.11 (q, *J* = 7.2 Hz, 2H), 4.14–3.74 (m, 3H), 2.33 (t, *J* = 7.2 Hz, 2H), 2.25 (m, 1H), 1.98 (m, 1H), 1.91 (d, *J* = 1.2 Hz, 3H), 1.75–1.67 (m, 2H), 1.24 (t, *J* = 7.2 Hz, 3H), 1.05–1.00 (m, 14H), 0.89 (s, 9H), 0.74–0.69 (m, 2H), 0.08 (s, 3H), 0.07 (s, 3H). **^{13}C NMR** (100 MHz, $CDCl_3$) δ 173.1, 163.5, 150.9, 137.9, 134.1, 128.2, 127.6, 127.5, 110.0, 87.7, 85.3, 72.1, 71.9, 70.4, 62.9, 60.2, 41.3, 37.8, 25.7, 19.0, 17.9, 17.6, 17.5, 14.2, 13.1, 12.3, 12.1, 10.2, –4.6, –4.8. **HRMS–ESI** (m/z):[M+Na]$^+$ calcd for $C_{36}H_{60}N_2O_8Si_2Na$; 727.3780, found 727.3771.

Ethyl 3-[(2*R*,3*R*,5*R*)-5-{3-[(Benzyloxy)methyl]-5-methyl-2,4-dioxo-3,4-dihydropyrimidin-1(2*H*)-yl}-3-[(*tert*-butyldimethylsilyl)oxy]-2-({[diisopropyl(methyl)silyl]oxy}methyl)tetrahydrofuran-3-yl]propanoate (3ha-2)

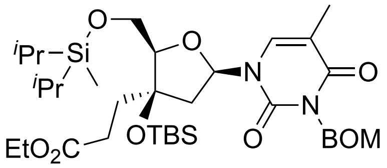

Colorless oil. **IR** (neat) 1095, 1465, 1647, 1653, 1663, 1706, 1733, 2865, 2931, 2951 cm^{-1}. The stereochemistry was determined by NOESY. **^{1}H NMR** (400 MHz, $CDCl_3$) δ 7.53 (m, 1H), 7.38–7.36 (m, 2H), 7.33–7.30 (m, 2H), 7.26 (m, 1H), 5.89 (dd, *J* = 6.8, 5.2 Hz, 1H), 5.48 (d, *J* = 0.8 Hz, 2H), 4.68 (s, 2H), 4.15 (q, *J* = 7.2 Hz, 2H), 3.96–3.88 (m, 2H), 3.80 (dd, *J* = 10.8, 7.2 Hz, 1H), 2.60 (dd, J = 14.0, 6.8 Hz, 1H), 2.45–2.39 (m, 2H), 2.13–2.07 (m, 3H), 1.93 (d, *J* = 0.8 Hz, 3H), 1.27 (t, *J* = 7.2 Hz, 3H), 1.04–0.95 (m, 14H), 0.85 (s, 9H), 0.13 (s, 3H), 0.06 (s, 3H), 0.01 (s, 3H). **^{13}C NMR** (100 MHz, $CDCl_3$) δ 172.5, 163.5, 150.8, 137.9, 134.3, 128.2, 127.1, 127.6, 109.2, 86.5, 85.3, 81.2, 72.1, 70.2, 63.0, 60.7, 46.0, 33.7, 22.7, 29.5, 25.7, 18.3, 17.58, 17.56, 17.37, 17.35, 14.1, 13.2, 12.8, 12.7, –2.2, –2.3. **HRMS–ESI** (m/z):[M+H]$^+$ calcd for $C_{36}H_{61}N_2O_8Si_2$; 705.3961, found 705.3952.

■ Computational Studies ■

All the restricted and unrestricted density functional theory (DFT) calculations were performed with the Gaussian 16 package. The method (U)B3LYP functional with the 6-31++G(d,p) basis set was used for the geometry optimization and normal vibrational mode analysis. The single-point energies were calculated at (U)B3LYP functional with the 6-311++G(d,p) basis set with SMD model (acetonitrile). The IRC calculations were performed for each transitions state to confirm the transition state connecting the reaction pathway between the starting materials and the products.

A. Gibbs free energy diagram of reaction of 1d calculated at (U)B3LYP/6-311++G(d,p)/SMD//(U)B3LYP/6-31++G(d,p)

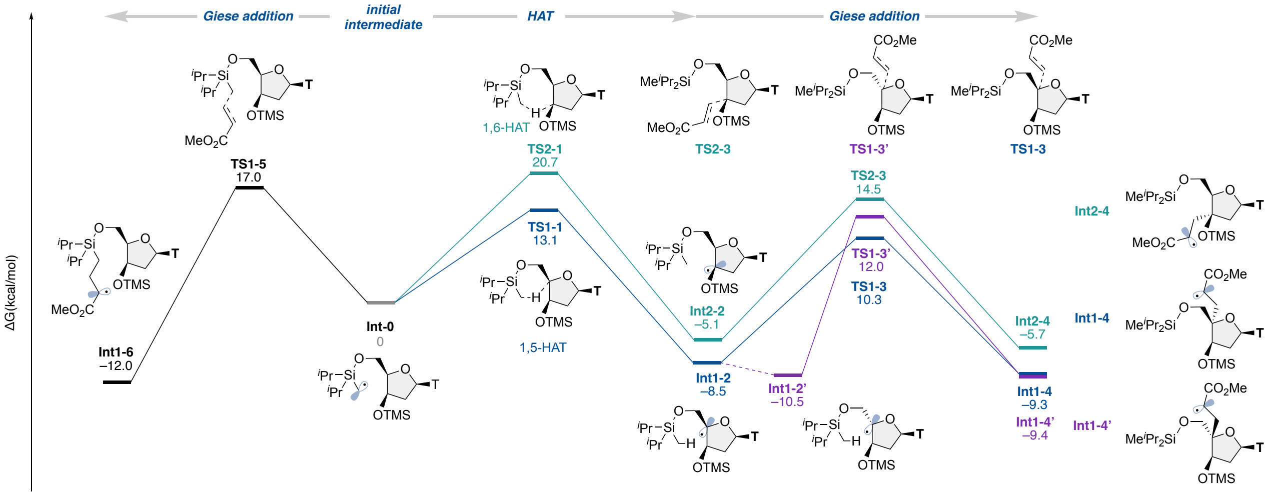

B. Gibbs free energy diagram of reaction of 1e calculated at (U)B3LYP/6-311++G(d,p)/SMD//(U)B3LYP/6-31++G(d,p)

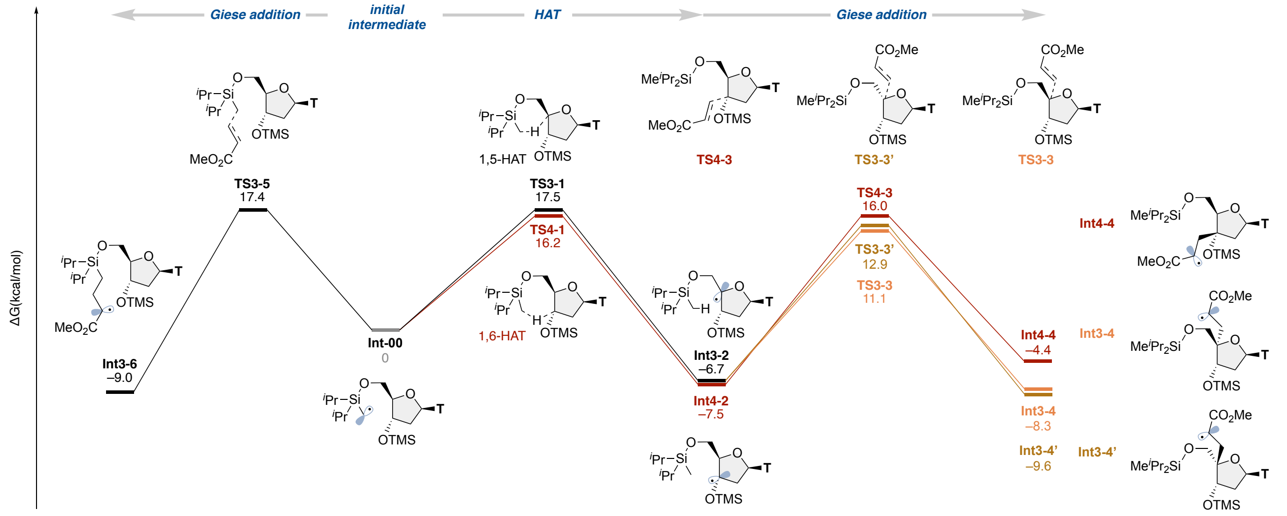

UB3LYP /6-311++G(d,p)(SMD)//UB3LYP/6-31++G (d,p)				
	E (hartree)	G_{corr} (hartree)	Gibbs (hartree)	Gibbs (kcal/mol)
**Int-0**	-1849.58626397	0.476794	-1849.10947	-1160334.684
Methyl acrylate	-306.5712692	0.064759	-306.5065102	-192335.9002
**Int-0**+Methyl acrylate				-1352670.584
**TS1-1**	-1849.564575	0.475941	-1849.088634	-1160321.609
**Int1-2**	-1849.59967	0.476715	-1849.122955	-1160343.146
**TS1-3**	-2156.163613	0.563997	-2155.599616	-1352660.315
**Int1-4**	-2156.199024	0.568155	-2155.630869	-1352679.926
**Int1-2’**	-1849.601661	0.475359	-1849.126302	-1160345.246
**TS1-3’**	-2156.159552	0.562741	-2155.596811	-1352658.555
**Int1-4’**	-2156.197866	0.566941	-2155.630925	-1352679.962
**TS1-5**	-2156.150443	0.56163	-2155.588813	-1352653.536
**Int1-6**	-2156.1968	0.56163	-2155.63517	-1352682.625
**TS2-1**	-1849.5566	0.479018	-1849.077582	-1160314.673
**Int2-2**	-1849.594488	0.475826	-1849.118662	-1160340.452
**TS2-3**	-2156.157074	0.563151	-2155.593923	-1352656.742
**Int2-4**	-2156.193288	0.567022	-2155.626266	-1352677.038
**Int-00**	-1849.587381	0.475005	-1849.112376	-1160336.507
**Int-00**+Methyl acrylate				-1352672.407
**TS3-1**	-1849.559755	0.475199	-1849.084556	-1160319.05
**Int3-2**	-1849.600717	0.477665	-1849.123052	-1160343.207
**TS3-3**	-2156.16261	0.561513	-2155.601097	-1352661.245
**Int3-4**	-2156.197938	0.565669	-2155.632269	-1352680.805
**TS3-3’**	-2156.161789	0.563498	-2155.598291	-1352659.483
**Int3-4’**	-2156.199912	0.565637	-2155.634275	-1352682.064
**TS3-5**	-2156.151169	0.559937	-2155.591232	-1352655.054
**Int3-6**	-2156.197261	0.564073	-2155.633188	-1352681.382
**TS4-1**	-1849.562456	0.475974	-1849.086482	-1160320.259
**Int4-2**	-1849.598988	0.474586	-1849.124402	-1160344.054
**TS4-3**	-2156.156198	0.562738	-2155.59346	-1352656.452
**Int4-4**	-2156.191207	0.565256	-2155.625951	-1352676.841

Int-0

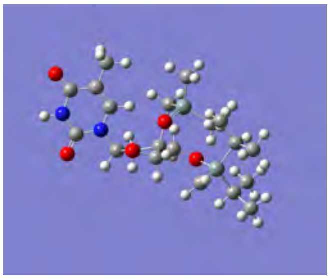

Minimum frequencies (cm $^{-1}$) = 10.5980 15.1936 15.8908

Charge = 0 Multiplicity = 2

C	-0.67821	-0.44012	0.
O	0.39435	-0.83876	0.85234
C	1.59618	-0.16451	0.45226
C	1.12417	1.2188	-0.03377
C	-0.19938	0.84548	-0.72738
H	-0.9184	-1.2361	-0.7062
H	2.0527	-0.6925	-0.4005
H	-0.93295	1.65199	-0.67597
H	-0.02321	0.61603	-1.78294
C	2.57183	-0.19826	1.62085
H	2.62532	-1.228	1.99568
H	2.19843	0.44473	2.42422
O	3.83861	0.2591	1.16409
Si	5.35758	-0.29855	1.6496
C	5.26327	-2.13573	1.94718
H	4.75166	-2.81411	1.26435
H	5.74362	-2.64187	2.78273
C	6.44082	0.08777	0.12552
H	5.88109	-0.38944	-0.69421
C	5.91111	0.60372	3.23899
H	6.1078	1.64191	2.93029
H	1.85089	1.65093	-0.73019
O	0.90151	2.08729	1.07173
Si	1.20524	3.7527	1.09272
C	-0.02282	4.65024	-0.02519
H	0.1471	5.73337	0.00542
H	0.07251	4.3363	-1.07128
H	-1.0573	4.46857	0.28748
C	2.9647	4.0974	0.51174
H	3.20571	5.15949	0.64126
H	3.69127	3.51169	1.08417
H	3.10612	3.86112	-0.549

C	0.96439	4.24536	2.88916
H	1.13272	5.32003	3.02534
H	-0.05317	4.02388	3.22924
H	1.66205	3.7112	3.54313
C	-3.01394	-1.03151	0.47515
C	-1.86344	0.52429	1.94358
C	-2.91403	0.67479	2.78562
H	-0.92119	1.03832	2.09786
C	-4.1424	-0.06409	2.49792
N	-1.8848	-0.28637	0.81908
N	-4.07653	-0.863	1.34466
H	-4.91074	-1.39111	1.11433
O	-3.07895	-1.76106	-0.50702
O	-5.17131	-0.02837	3.16451
C	-2.88079	1.55315	4.0042
H	-3.65707	2.3243	3.9536
H	-3.07792	0.97055	4.91047
H	-1.90732	2.0402	4.10816
C	6.51309	1.59693	-0.18066
H	7.06903	2.13729	0.59548
H	7.03288	1.77551	-1.1313
H	5.51823	2.04678	-0.2548
C	7.84772	-0.54029	0.15148
H	8.35003	-0.39909	-0.81423
H	8.48504	-0.07584	0.91273
H	7.81755	-1.61629	0.35481
C	7.21673	0.0397	3.83808
H	8.04815	0.05285	3.12643
H	7.5252	0.62811	4.71192
H	7.08891	-0.99512	4.17765
C	4.80259	0.64417	4.31117
H	3.89593	1.13646	3.9463
H	4.52451	-0.36396	4.64268
H	5.14191	1.19415	5.1988

TS1-1

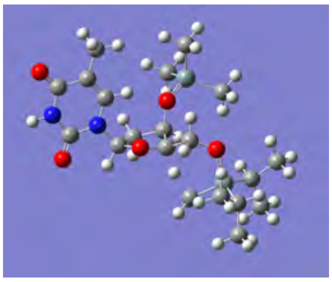

Minimum frequencies (cm $^{-1}$) = –1577.3547

Charge = 0 Multiplicity = 2

C	-0.11544	0.64214	0.
O	-1.00881	0.46206	-1.1107
C	-2.11727	1.3135	-0.98269
C	-1.65434	2.53938	-0.20007
C	-0.60388	1.914	0.73694
H	-0.15119	-0.24155	0.6366
H	-3.07018	0.73954	-0.26061
H	0.20924	2.60682	0.96017
H	-1.06939	1.62001	1.68227
C	-2.84159	1.47175	-2.31083
H	-2.80943	0.50561	-2.83074
H	-2.32431	2.20839	-2.93586
O	-4.17657	1.92566	-2.12686
Si	-5.3316	1.10172	-1.18388
C	-4.33528	0.27531	0.17045
H	-4.41832	0.66003	1.18987
H	-4.20303	-0.80866	0.16647
C	-6.41973	2.51439	-0.51571
H	-5.70496	3.14626	0.03572
C	-6.28041	-0.14493	-2.27722
H	-6.9729	0.4641	-2.87855
H	-2.48567	2.98629	0.35642
O	-1.06156	3.50098	-1.07536
Si	-1.33163	5.17492	-1.02351
C	-0.56949	5.89401	0.54659
H	-0.72132	6.97939	0.58907
H	-1.0199	5.46794	1.45091
H	0.50971	5.7084	0.58954
C	-3.18054	5.53333	-1.06126
H	-3.35503	6.61026	-1.17442
H	-3.66843	5.01958	-1.89612
H	-3.68356	5.22002	-0.13939

C	-0.46863	5.83043	-2.55728
H	-0.57131	6.91964	-2.62682
H	0.60201	5.59878	-2.54172
H	-0.89533	5.39519	-3.46739
C	2.13921	-0.31231	-0.10207
C	1.62073	1.6516	-1.44035
C	2.84729	1.71892	-2.01001
H	0.83561	2.36118	-1.67967
C	3.84351	0.71739	-1.62983
N	1.25324	0.69008	-0.50865
N	3.38728	-0.22122	-0.68954
H	4.05076	-0.93375	-0.40641
O	1.85559	-1.18639	0.7072
O	4.99059	0.65298	-2.0572
C	3.23755	2.76316	-3.01722
H	4.09154	3.35003	-2.66225
H	3.5474	2.29893	-3.9595
H	2.40431	3.4414	-3.21985
C	-7.02094	3.37747	-1.64325
H	-7.75761	2.81301	-2.22818
H	-7.54003	4.25227	-1.23006
H	-6.25171	3.73776	-2.3331
C	-7.50895	2.06924	0.47986
H	-7.99628	2.94101	0.93533
H	-8.29436	1.48886	-0.01784
H	-7.10733	1.4538	1.29285
C	-7.12353	-1.14172	-1.45427
H	-7.82936	-0.64367	-0.78193
H	-7.70699	-1.79355	-2.11748
H	-6.48733	-1.78975	-0.83987
C	-5.36426	-0.89996	-3.26206
H	-4.827	-0.21612	-3.92647
H	-4.62235	-1.51469	-2.7367
H	-5.95234	-1.57929	-3.89301

Int1-2

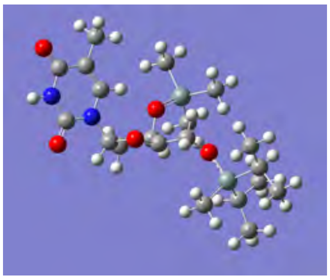

Minimum frequencies (cm $^{-1}$) = 7.7811 10.8510 13.0003

Charge = 0 Multiplicity = 2

C	0.21958	0.55352	0.
O	1.19623	0.31467	1.03912
C	2.25198	1.19028	0.90543
C	1.85573	2.37986	0.09142
C	0.73047	1.78742	-0.77921
H	0.16259	-0.34109	-0.61762
H	4.97232	-0.87138	-0.23305
H	-0.05752	2.51688	-0.97385
H	1.13476	1.45552	-1.73938
C	3.24926	1.14969	2.0156
H	3.33049	0.11384	2.36926
H	2.91057	1.75423	2.8737
O	4.49685	1.68344	1.58378
Si	5.93631	0.8307	1.32608
C	5.55028	-0.90987	0.6971
H	6.47242	-1.46573	0.49387
H	4.97279	-1.49577	1.42061
C	6.80424	1.86415	-0.02025
H	6.01028	2.00107	-0.77167
C	6.89454	0.76121	2.97708
H	7.16015	1.80475	3.20493
H	2.68576	2.75957	-0.51061
O	1.32437	3.4503	0.90859
Si	2.03545	4.95888	1.21225
C	2.92055	5.57612	-0.33717
H	3.28312	6.59852	-0.17522
H	3.79157	4.9628	-0.59294
H	2.25163	5.59795	-1.20513
C	3.24475	4.84344	2.65005
H	3.72412	5.81281	2.83414
H	2.73193	4.54821	3.57291
H	4.03058	4.10734	2.44915

C	0.58909	6.07884	1.65213
H	0.93772	7.08874	1.89882
H	-0.12057	6.16145	0.82168
H	0.0448	5.69668	2.52302
C	-2.01699	-0.35139	0.39762
C	-1.38166	1.74355	1.46208
C	-2.55225	1.88159	2.12811
H	-0.58109	2.47121	1.55318
C	-3.57771	0.85347	1.94993
N	-1.09828	0.68387	0.60871
N	-3.20784	-0.18433	1.07842
H	-3.89309	-0.9179	0.9356
O	-1.8049	-1.31267	-0.32987
O	-4.68083	0.8447	2.48392
C	-2.85294	3.03385	3.04409
H	-3.74186	3.57841	2.70831
H	-3.06711	2.68017	4.05828
H	-2.01066	3.72943	3.08579
C	7.21621	3.26509	0.47493
H	8.02072	3.20622	1.21809
H	7.58904	3.87931	-0.35527
H	6.37816	3.79981	0.93403
C	7.98235	1.15972	-0.72271
H	8.34946	1.76619	-1.56082
H	8.8295	1.00817	-0.04403
H	7.70166	0.18082	-1.12621
C	8.20452	-0.04963	2.89328
H	8.89049	0.33763	2.13282
H	8.73656	-0.02673	3.85324
H	8.01092	-1.10337	2.65832
C	6.0184	0.24867	4.13902
H	5.12188	0.86111	4.27902
H	5.69675	-0.78787	3.97684
H	6.57768	0.26557	5.0836

TS1-3

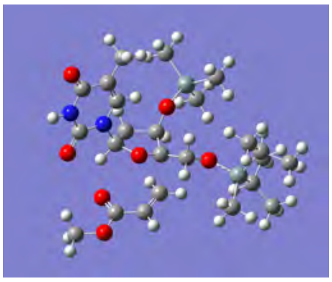

Minimum frequencies (cm $^{-1}$) = -266.6755

Charge = 0 Multiplicity = 2

C	0.51989	0.64195	0.
O	-0.59281	0.801	0.91917
C	-1.78653	0.54617	0.30019
C	-1.54513	-0.29563	-0.93658
C	-0.10231	0.10593	-1.30565
H	0.98441	1.61463	-0.13905
H	-5.21779	2.8408	2.03021
H	0.4534	-0.73426	-1.72536
H	-0.10503	0.921	-2.03308
C	-2.86963	0.25701	1.30508
H	-2.83224	1.03692	2.07699
H	-2.63289	-0.70223	1.79206
O	-4.14025	0.18467	0.68137
Si	-5.64736	0.45008	1.41323
C	-5.5247	1.93908	2.57234
H	-6.49474	2.14969	3.03647
H	-4.80704	1.77859	3.38424
C	-6.76572	0.83406	-0.08281
H	-6.12503	1.46736	-0.71598
C	-6.18718	-1.10875	2.37623
H	-6.18224	-1.92514	1.63802
H	-2.25684	-0.06078	-1.73169
O	-1.62705	-1.68866	-0.60717
Si	-2.44299	-2.9169	-1.443
C	-3.48039	-2.1904	-2.8399
H	-4.03537	-2.99489	-3.33758
H	-4.21344	-1.46321	-2.47527
H	-2.866	-1.70164	-3.6047
C	-3.51961	-3.80834	-0.18539
H	-4.04562	-4.65448	-0.64334
H	-2.91612	-4.20111	0.64081
H	-4.26851	-3.1314	0.23867

C	-1.14599	-4.09267	-2.14196
H	-1.61967	-4.94489	-2.64422
H	-0.50194	-3.59231	-2.87413
H	-0.50276	-4.49313	-1.35025
C	2.69689	0.41747	1.09089
C	1.26559	-1.53981	0.89455
C	2.11995	-2.36251	1.5473
H	0.30537	-1.88752	0.52712
C	3.38896	-1.80938	2.02056
N	1.52476	-0.20007	0.63455
N	3.56426	-0.44616	1.73511
H	4.42581	-0.02479	2.06407
O	2.95167	1.60286	0.93107
O	4.25919	-2.43111	2.61957
C	1.83203	-3.81213	1.81725
H	2.59365	-4.45384	1.36141
H	1.85071	-4.02	2.89237
H	0.8524	-4.09602	1.42324
C	-7.12488	-0.42014	-0.90493
H	-7.78506	-1.09326	-0.34499
H	-7.65228	-0.14421	-1.82717
H	-6.23562	-0.99115	-1.19161
C	-8.02853	1.65421	0.25214
H	-8.56912	1.91814	-0.66586
H	-8.72632	1.09353	0.88454
H	-7.79014	2.58884	0.77061
C	-7.62104	-0.99642	2.93698
H	-8.36489	-0.81883	2.15376
H	-7.90668	-1.9218	3.4536
H	-7.70644	-0.18219	3.66699
C	-5.20521	-1.49631	3.50151
H	-4.194	-1.6832	3.12665
H	-5.13822	-0.7138	4.26716
H	-5.53852	-2.41199	4.00706
C	-2.49455	2.56252	-0.63043
H	-2.10134	2.27418	-1.59987
H	-3.51881	2.28504	-0.41407
C	-1.87914	3.58158	0.044
C	-0.55336	4.04699	-0.36522
O	0.106	3.58721	-1.29315
O	-0.10642	5.05943	0.41795

C	1.21841	5.54105	0.12577
H	1.40022	6.33917	0.84538
H	1.9499	4.73818	0.24696
H	1.26919	5.92487	-0.89629
H	-2.30814	4.02541	0.93642

Int1-4

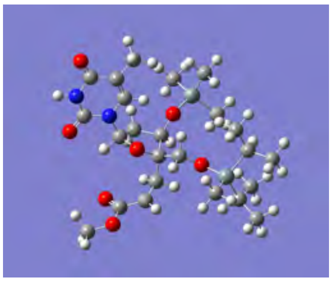

Minimum frequencies (cm $^{-1}$) = 14.3614 16.4371 16.9083

Charge = 0 Multiplicity = 2

C	-0.07685	0.52441	0.
O	1.03202	0.765	-0.87143
C	2.28102	0.53438	-0.19835
C	1.9547	-0.56423	0.85169
C	0.50029	-0.21635	1.23101
H	-0.5461	1.46854	0.27607
H	5.58915	2.2119	-2.85462
H	-0.07054	-1.11123	1.4838
H	0.47164	0.45342	2.09459
C	3.25607	0.12146	-1.30904
H	3.20306	0.88146	-2.09871
H	2.91284	-0.83012	-1.72502
O	4.57205	0.00513	-0.79151
Si	6.02929	0.05563	-1.64989
C	5.8441	1.18835	-3.15303
H	6.78171	1.23762	-3.71797
H	5.06816	0.83967	-3.84322
C	7.24463	0.80125	-0.38364
H	6.67454	1.64576	0.03501
C	6.48355	-1.71823	-2.19177
H	6.55025	-2.29782	-1.25825
H	2.63256	-0.48061	1.70867
O	2.03835	-1.86319	0.27695
Si	2.55203	-3.25683	1.09228
C	4.24999	-2.96964	1.85795
H	4.62945	-3.89982	2.29801

H	4.96956	-2.63151	1.10552
H	4.22478	-2.22095	2.65791
C	2.61038	-4.58066	-0.23851
H	2.93333	-5.54016	0.18209
H	1.62448	-4.7333	-0.69111
H	3.31025	-4.31228	-1.03692
C	1.31656	-3.71678	2.44344
H	1.62843	-4.63886	2.94897
H	1.23677	-2.93784	3.21066
H	0.31529	-3.88824	2.03238
C	-2.34643	0.38616	-0.93762
C	-0.78434	-1.42794	-1.34272
C	-1.6414	-2.1409	-2.11249
H	0.22257	-1.77347	-1.13559
C	-2.97818	-1.59622	-2.34439
N	-1.09793	-0.2138	-0.7517
N	-3.21121	-0.36193	-1.71901
H	-4.12651	0.04991	-1.86184
O	-2.66924	1.46267	-0.45389
O	-3.85914	-2.12534	-3.01448
C	-1.28882	-3.45644	-2.74708
H	-1.96631	-4.24875	-2.41068
H	-1.38759	-3.40323	-3.83661
H	-0.26242	-3.745	-2.50408
C	7.56166	-0.1587	0.78072
H	8.14252	-1.02457	0.44024
H	8.15812	0.34635	1.55163
H	6.6521	-0.53529	1.26023
C	8.53868	1.38214	-0.98924
H	9.13119	1.88781	-0.21591
H	9.17583	0.60152	-1.42002
H	8.3363	2.11598	-1.7768
C	7.85116	-1.81362	-2.89958
H	8.67342	-1.45643	-2.27143
H	8.07547	-2.85391	-3.16888
H	7.86636	-1.23139	-3.82908
C	5.38394	-2.36586	-3.05878
H	4.41628	-2.39022	-2.54774
H	5.24754	-1.82941	-4.00583
H	5.64829	-3.40094	-3.31229
C	2.7634	1.82228	0.54643

H	2.16858	1.95681	1.45455
H	3.80518	1.63931	0.83367
C	2.65848	3.05839	-0.26873
C	1.55764	3.98062	-0.06392
O	0.68975	3.852	0.79508
O	1.58919	5.02225	-0.93555
C	0.51128	5.96766	-0.81355
H	0.68666	6.70877	-1.593
H	-0.45062	5.47038	-0.96097
H	0.52358	6.43611	0.17408
H	3.36219	3.27288	-1.06633

TS1-5

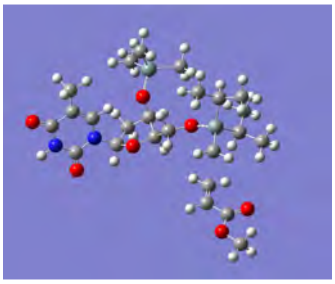

Minimum frequencies (cm $^{-1}$) = -305.9625

Charge = 0 Multiplicity = 2

C	-2.55091	-1.48422	-1.40957
O	-1.39842	-1.39956	-0.5709
C	-0.58641	-0.29329	-0.98752
C	-1.58908	0.76976	-1.47466
C	-2.65186	-0.12101	-2.1457
H	-2.44904	-2.31388	-2.11042
H	0.04008	-0.59194	-1.84368
H	-3.65107	0.31365	-2.08422
H	-2.41209	-0.26628	-3.20363
C	0.32595	0.08648	0.17108
H	0.79627	-0.8302	0.54932
H	-0.27464	0.52331	0.97562
O	1.29131	1.01038	-0.3067
Si	2.82243	1.34975	0.32165
C	3.57046	-0.20602	1.04106
H	4.64138	-0.26131	1.22809
C	3.79574	1.95783	-1.19872
H	3.53402	1.22031	-1.97309
C	2.64002	2.68753	1.68206
H	2.19509	3.55202	1.1648

H	-1.11048	1.4517	-2.18574
O	-2.13317	1.48674	-0.37223
Si	-2.55114	3.12826	-0.37772
C	-4.0613	3.41008	-1.47439
H	-4.35909	4.46553	-1.45636
H	-3.86271	3.14711	-2.52001
H	-4.9195	2.81868	-1.13577
C	-1.10612	4.16204	-1.00655
H	-1.33055	5.23024	-0.89934
H	-0.192	3.94958	-0.44271
H	-0.89482	3.98151	-2.06661
C	-2.94336	3.51205	1.41847
H	-3.23581	4.56172	1.53851
H	-3.77011	2.89415	1.7857
H	-2.0755	3.33033	2.06151
C	-4.41064	-2.99871	-0.88381
C	-4.01795	-1.06566	0.53482
C	-5.0253	-1.35531	1.39292
H	-3.38166	-0.1969	0.6636
C	-5.82607	-2.55262	1.14059
N	-3.70324	-1.83509	-0.5751
N	-5.43718	-3.27553	0.00097
H	-5.96959	-4.11363	-0.20409
O	-4.16637	-3.71151	-1.84967
O	-6.76661	-2.94078	1.82509
C	-5.35797	-0.51729	2.5948
H	-6.38901	-0.15094	2.54456
H	-5.28045	-1.10657	3.51475
H	-4.68344	0.33984	2.67225
C	3.30549	3.33242	-1.69876
H	3.54615	4.12927	-0.9847
H	3.79011	3.59398	-2.64827
H	2.22306	3.34714	-1.8641
C	5.33047	1.9409	-1.0441
H	5.81136	2.20798	-1.99383
H	5.67348	2.66672	-0.29833
H	5.71466	0.95844	-0.7512
C	3.98726	3.13858	2.28138
H	4.66958	3.53804	1.52464
H	3.8317	3.92734	3.0289
H	4.4992	2.31032	2.78583

C	1.66813	2.26585	2.80256
H	0.67042	2.0295	2.41934
H	2.03601	1.38594	3.34423
H	1.55328	3.0728	3.53825
H	3.00345	-0.85497	1.70679
C	3.70813	-1.79077	-0.74561
H	2.64303	-1.71628	-0.92986
H	4.35853	-1.09295	-1.26032
C	4.23734	-2.91535	-0.18958
H	3.6123	-3.70205	0.22055
C	5.69485	-3.07965	-0.07042
O	6.5336	-2.25322	-0.39644
O	6.01253	-4.28605	0.46353
C	7.41744	-4.55401	0.61591
H	7.47717	-5.55221	1.04889
H	7.91967	-4.52333	-0.35429
H	7.87755	-3.81724	1.27927

Int1-6

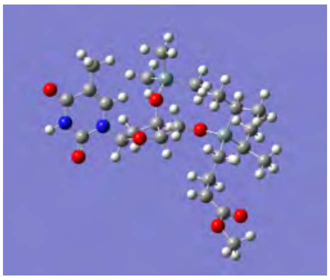

Minimum frequencies (cm $^{-1}$) = 7.1117 10.3472 14.5165

Charge = 0 Multiplicity = 2

C	2.47875	1.55689	-1.2717
O	1.38942	1.37827	-0.36577
C	0.56394	0.29836	-0.82355
C	1.54619	-0.70151	-1.46226
C	2.54721	0.26252	-2.12667
H	2.31536	2.44012	-1.89067
H	-0.11928	0.6587	-1.60963
H	3.55407	-0.15621	-2.17073
H	2.23215	0.49126	-3.14951
C	-0.26948	-0.19484	0.35218
H	-0.70893	0.6798	0.84862
H	0.38212	-0.70556	1.06855
O	-1.26396	-1.08054	-0.14058
Si	-2.8447	-1.28758	0.42239

C	-3.59549	0.40931	0.85358
H	-4.62726	0.24169	1.18943
C	-3.72335	-2.07258	-1.07442
H	-3.38007	-1.45349	-1.91781
C	-2.82922	-2.40169	1.97606
H	-2.39594	-3.35467	1.63536
H	1.0304	-1.33359	-2.1934
O	2.17732	-1.49237	-0.46111
Si	2.60156	-3.12416	-0.62416
C	3.95676	-3.31452	-1.92419
H	4.25599	-4.36554	-2.01767
H	3.62305	-2.98337	-2.9146
H	4.85084	-2.73844	-1.66031
C	1.0933	-4.13761	-1.12208
H	1.34015	-5.20614	-1.13503
H	0.26842	-3.98808	-0.41788
H	0.72906	-3.87705	-2.12223
C	3.22548	-3.61203	1.07886
H	3.5273	-4.66576	1.09657
H	4.09525	-3.01351	1.371
H	2.44952	-3.47329	1.83941
C	4.34802	3.06029	-0.74827
C	4.08589	1.00647	0.5232
C	5.14771	1.24323	1.3304
H	3.47279	0.11725	0.62105
C	5.91112	2.47358	1.1261
N	3.68164	1.86029	-0.49196
N	5.43115	3.28225	0.08288
H	5.93534	4.14543	-0.08629
O	4.02483	3.84725	-1.62971
O	6.89265	2.82168	1.77364
C	5.57768	0.31349	2.42959
H	6.60652	-0.02878	2.27409
H	5.56102	0.82214	3.3994
H	4.92088	-0.5591	2.48249
C	-3.24947	-3.51498	-1.34746
H	-3.5696	-4.2002	-0.553
H	-3.6747	-3.89121	-2.28697
H	-2.15952	-3.5808	-1.42686
C	-5.26396	-1.99344	-1.04351
H	-5.68256	-2.3729	-1.98447

H	-5.68773	-2.60396	-0.23813
H	-5.63254	-0.97061	-0.91459
C	-4.23834	-2.70149	2.52929
H	-4.88631	-3.18009	1.78855
H	-4.17765	-3.37552	3.39357
H	-4.74186	-1.78828	2.8689
C	-1.91931	-1.85862	3.09777
H	-0.88532	-1.72255	2.76586
H	-2.27737	-0.89449	3.47926
H	-1.90022	-2.55183	3.94887
H	-3.06994	0.83965	1.71672
C	-3.60781	1.43903	-0.31799
H	-2.57606	1.64767	-0.62595
H	-4.13472	1.01105	-1.17596
C	-4.27403	2.70971	0.07561
H	-3.7454	3.45411	0.66413
C	-5.6714	2.94931	-0.22588
O	-6.40436	2.17455	-0.83509
O	-6.09882	4.1525	0.24881
C	-7.4746	4.47321	-0.01399
H	-7.6325	5.45782	0.42577
H	-7.66444	4.49705	-1.09037
H	-8.13601	3.73484	0.44712

Int1-2’

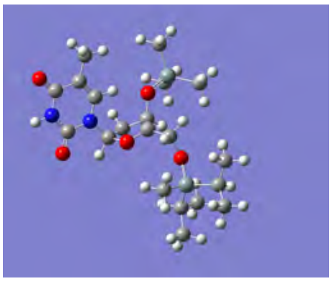

Minimum frequencies (cm $^{-1}$) = 8.0544 11.2123 13.7131

Charge = 0 Multiplicity = 2

C	1.33087	-1.1686	1.14023
O	0.3222	-1.14047	0.11182
C	-0.0193	0.15837	-0.19308
C	0.55118	1.13484	0.79199
C	1.25127	0.21331	1.81625
H	1.08236	-1.99272	1.80473
H	-2.27552	-2.79586	-0.59474
H	2.22895	0.59874	2.10986

H	0.63655	0.12027	2.71579
C	-1.2469	0.33875	-1.0066
H	-1.27844	-0.42677	-1.79048
H	-1.21574	1.32117	-1.49198
O	-2.42688	0.29063	-0.18357
Si	-3.72852	-0.78295	-0.29019
C	-3.16672	-2.3995	-1.09367
H	-3.95345	-3.15927	-1.02782
H	-2.92752	-2.27729	-2.15624
C	-4.21356	-1.06308	1.53183
H	-3.29181	-1.47326	1.97467
C	-5.10762	0.04238	-1.32725
H	-5.54949	0.81556	-0.68027
H	-0.24587	1.71707	1.27362
O	1.48264	2.03273	0.15843
Si	1.41183	3.72217	0.25344
C	1.51784	4.27266	2.05628
H	1.50569	5.36707	2.1272
H	0.67538	3.90009	2.65056
H	2.44259	3.91973	2.52664
C	-0.19688	4.34466	-0.5099
H	-0.25219	5.43868	-0.45614
H	-0.26893	4.05959	-1.56555
H	-1.07935	3.9483	0.0054
C	2.90346	4.30698	-0.72631
H	2.95706	5.40182	-0.74292
H	3.83616	3.93363	-0.28959
H	2.8536	3.95892	-1.76379
C	3.10149	-2.81736	0.79374
C	3.30412	-0.64102	-0.27548
C	4.45766	-0.94689	-0.91556
H	2.8383	0.33309	-0.38645
C	5.03065	-2.27788	-0.71709
N	2.63071	-1.51726	0.56817
N	4.28718	-3.10173	0.14421
H	4.65137	-4.03512	0.30015
O	2.53359	-3.6313	1.51032
O	6.0651	-2.69406	-1.22583
C	5.17978	0.01111	-1.81999
H	6.19682	0.19823	-1.4591
H	5.27749	-0.40446	-2.82858

H	4.6481	0.96411	-1.88349
C	-4.53036	0.25535	2.26617
H	-5.45146	0.71379	1.88524
H	-4.67919	0.07884	3.33975
H	-3.72335	0.98628	2.15382
C	-5.33373	-2.09805	1.75606
H	-5.46511	-2.29942	2.82719
H	-6.2979	-1.73701	1.37936
H	-5.12313	-3.0562	1.26821
C	-6.23316	-0.93245	-1.73273
H	-6.69885	-1.42109	-0.87073
H	-7.02731	-0.40458	-2.27687
H	-5.85824	-1.72156	-2.39574
C	-4.54137	0.75572	-2.57219
H	-3.7999	1.51518	-2.30491
H	-4.06308	0.04706	-3.26084
H	-5.34183	1.25544	-3.13362

TS1-3’

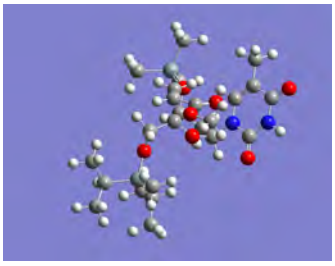

Minimum frequencies (cm $^{-1}$) = -319.6887

Charge = 0 Multiplicity = 2

C	2.47288	1.22061	0.
O	1.76716	0.84258	-1.20354
C	1.44387	1.92921	-1.9801
C	1.75073	3.22306	-1.22436
C	2.14462	2.71118	0.18011
H	2.06359	0.58778	0.78423
H	-0.56801	-1.08597	-1.13143
H	2.9592	3.28915	0.61892
H	1.28096	2.7833	0.84649
C	0.11518	1.77168	-2.67243
H	0.07721	0.78529	-3.14548
H	0.01929	2.53342	-3.45564
O	-0.94569	1.95217	-1.73531
Si	-2.15133	0.84129	-1.30313

C	-1.5421	-0.92035	-1.60407
H	-2.24633	-1.64885	-1.18701
H	-1.43787	-1.14983	-2.67061
C	-2.3984	1.17365	0.55745
H	-1.39712	0.99203	0.98009
C	-3.70344	1.21367	-2.3559
H	-4.13317	2.14035	-1.94594
H	0.83324	3.82099	-1.16714
O	2.79501	3.98961	-1.82931
Si	2.71733	5.66075	-2.10169
C	2.45019	6.56876	-0.4691
H	2.42458	7.65322	-0.63034
H	1.50299	6.2895	0.00687
H	3.25785	6.35898	0.24131
C	1.30102	6.04702	-3.28641
H	1.24451	7.12503	-3.47978
H	1.44121	5.54492	-4.25028
H	0.32905	5.7386	-2.88445
C	4.38083	6.0869	-2.85937
H	4.4349	7.15325	-3.10776
H	5.20159	5.86442	-2.16881
H	4.55285	5.51949	-3.78036
C	4.24708	-0.38434	0.425
C	4.83005	1.70514	-0.66904
C	6.14196	1.3982	-0.78463
H	4.43508	2.63639	-1.0589
C	6.60219	0.11018	-0.26812
N	3.88899	0.87911	-0.06558
N	5.5933	-0.66598	0.32087
H	5.87111	-1.57757	0.66611
O	3.44921	-1.16163	0.9338
O	7.75568	-0.30347	-0.3059
C	7.14546	2.29466	-1.45122
H	7.96914	2.53631	-0.77107
H	7.58974	1.79662	-2.31972
H	6.67901	3.22605	-1.78407
C	-2.76613	2.64201	0.852
H	-3.76659	2.88669	0.47441
H	-2.77477	2.83387	1.9331
H	-2.05865	3.33978	0.39233
C	-3.37516	0.21256	1.26387

H	-3.36826	0.38313	2.34817
H	-4.40633	0.36355	0.92393
H	-3.11987	-0.83974	1.09834
C	-4.78008	0.11199	-2.26009
H	-5.10143	-0.0755	-1.2304
H	-5.67295	0.39071	-2.83478
H	-4.4166	-0.83806	-2.66988
C	-3.34608	1.48755	-3.8314
H	-2.64795	2.32436	-3.93393
H	-2.89025	0.60915	-4.30633
H	-4.2457	1.73294	-4.411
C	2.78587	1.73678	-3.83817
H	2.30375	2.5839	-4.31331
H	3.65046	1.95359	-3.22459
C	2.62315	0.47583	-4.34901
C	3.42644	-0.64918	-3.86164
O	4.32538	-0.58627	-3.03493
O	3.05649	-1.81533	-4.4573
C	3.78705	-2.9837	-4.04758
H	3.65399	-3.16194	-2.97739
H	3.36934	-3.80611	-4.62837
H	4.85261	-2.86423	-4.25986
H	1.86231	0.25194	-5.09018

Int1-4'

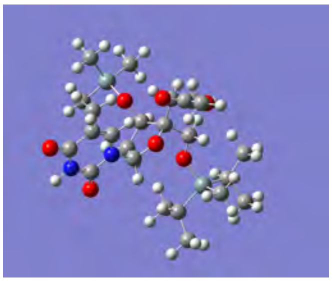

Minimum frequencies (cm $^{-1}$) = 8.2964 12.1378 15.4956

Charge = 0 Multiplicity = 2

C	0.61031	0.18987	0.
O	0.81306	-0.98027	0.76084
C	1.25191	-0.68938	2.10989
C	0.92956	0.81925	2.32666
C	0.97733	1.38277	0.90006
H	1.231	0.15627	-0.89746
H	3.9685	-2.24117	-0.82169
H	0.30002	2.22569	0.76674

H	1.99116	1.70357	0.65987
C	2.76049	-0.98572	2.2128
H	2.91825	-2.02224	1.8908
H	3.05502	-0.90693	3.27002
O	3.52935	-0.09156	1.43375
Si	4.95631	-0.41606	0.57615
C	4.85305	-2.14431	-0.1822
H	5.7341	-2.34815	-0.80089
H	4.80319	-2.9332	0.57643
C	4.94734	0.93457	-0.7674
H	3.90611	0.92497	-1.12527
C	6.43946	-0.28574	1.77296
H	6.41965	0.74828	2.14922
H	1.69932	1.27232	2.9634
O	-0.35823	0.9785	2.90451
Si	-0.83798	2.20917	3.95802
C	-0.68082	3.89681	3.12969
H	-1.01971	4.68941	3.80789
H	0.35508	4.12547	2.85361
H	-1.29073	3.95553	2.22135
C	0.24038	2.16597	5.50881
H	-0.07617	2.94121	6.21703
H	0.16405	1.19967	6.02022
H	1.29834	2.34666	5.28529
C	-2.62706	1.8084	4.35721
H	-3.05338	2.55146	5.04127
H	-3.23817	1.79863	3.44835
H	-2.71676	0.82474	4.83073
C	-1.04687	1.1439	-1.50416
C	-1.78949	-0.6165	-0.02072
C	-3.06958	-0.59816	-0.47529
H	-1.46759	-1.3209	0.735
C	-3.42464	0.35439	-1.52123
N	-0.80052	0.22609	-0.49309
N	-2.35198	1.15466	-1.95576
H	-2.55415	1.82143	-2.69205
O	-0.1787	1.89002	-1.95362
O	-4.53528	0.4942	-2.02619
C	-4.13394	-1.52454	0.04359
H	-4.95499	-0.96025	0.50014
H	-4.57369	-2.10637	-0.77387

H	-3.71988	-2.21272	0.78401
C	5.22472	2.34242	-0.20126
H	6.25934	2.43688	0.15023
H	5.07286	3.10827	-0.97232
H	4.56548	2.5857	0.63916
C	5.84663	0.64338	-1.98628
H	5.70616	1.41154	-2.7572
H	6.91026	0.64584	-1.72161
H	5.62128	-0.32467	-2.44622
C	7.79909	-0.5046	1.07658
H	7.97151	0.20699	0.26279
H	8.62365	-0.38743	1.79172
H	7.87945	-1.51461	0.65627
C	6.3054	-1.22166	2.99194
H	5.39698	-1.02169	3.56939
H	6.28721	-2.27705	2.69266
H	7.15755	-1.09666	3.67281
C	0.47413	-1.61474	3.08734
H	0.81994	-1.3725	4.10158
H	-0.58082	-1.34869	3.02685
C	0.64279	-3.07159	2.83987
C	-0.40214	-3.85578	2.20989
O	-1.49325	-3.4223	1.85004
O	-0.04008	-5.15757	2.06311
C	-1.0166	-6.01277	1.44341
H	-1.2475	-5.66105	0.43466
H	-0.55748	-7.00043	1.41068
H	-1.93625	-6.03401	2.03382
H	1.54853	-3.59487	3.12972

TS2-1

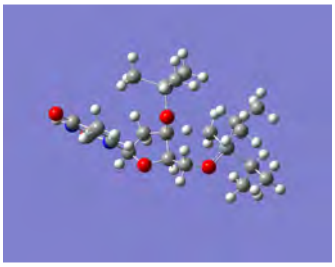

Minimum frequencies (cm $^{-1}$) = -1655.8576

Charge = 0 Multiplicity = 2

C	0.15873	-0.71429	0.
O	1.03009	-0.87295	1.10667

C	2.3471	-0.39925	0.73604
C	2.073	0.72109	-0.26215
C	0.93036	0.11568	-1.07005
H	-0.13966	-1.68852	-0.39415
H	2.86925	-1.21143	0.20872
H	3.26164	0.83304	-0.86119
H	0.29003	0.84049	-1.57053
H	1.32234	-0.56755	-1.83021
C	3.17602	-0.05148	1.96763
H	2.99608	-0.80754	2.73865
H	2.87805	0.9274	2.36171
O	4.5607	-0.08723	1.65333
Si	5.47098	0.82329	0.56101
C	4.62808	0.83239	-1.12786
H	4.74793	1.72863	-1.74145
H	4.73023	-0.06691	-1.74145
C	5.59951	2.63649	1.15157
H	4.63362	3.07916	0.86388
C	7.13527	-0.10278	0.49225
H	7.75381	0.45594	-0.22767
O	1.79516	1.92959	0.37144
Si	1.39659	3.46702	-0.23809
C	-0.40409	3.54383	-0.79116
H	-0.67525	4.58688	-0.99686
H	-0.59676	2.9752	-1.70704
H	-1.08092	3.17733	-0.01228
C	2.51952	3.91728	-1.68274
H	2.44385	3.19415	-2.50238
H	2.22997	4.89661	-2.08282
H	3.57016	3.97887	-1.38203
C	1.66336	4.60426	1.23226
H	1.42849	5.64197	0.96792
H	1.0177	4.32075	2.07065
H	2.70057	4.57248	1.58178
C	-2.19532	-0.20447	-0.36373
C	-1.1831	0.57792	1.69169
C	-2.32583	1.13712	2.16376
H	-0.25602	0.59534	2.24875
C	-3.53394	1.045	1.34717
N	-1.09426	-0.0714	0.47495
N	-3.35384	0.36396	0.13008

H	-4.17022	0.27826	-0.46517
O	-4.63462	1.49981	1.64065
C	-2.41649	1.8329	3.49285
H	-2.74622	2.87037	3.37158
H	-3.15374	1.34493	4.13939
H	-1.44931	1.82965	4.00313
O	-2.13751	-0.77103	-1.4509
C	5.73823	2.80894	2.67719
H	6.69658	2.4261	3.0435
H	5.68712	3.87055	2.95325
H	4.94877	2.28359	3.22373
C	6.7017	3.4163	0.4035
H	6.67539	4.48169	0.66701
H	7.70026	3.04489	0.66244
H	6.59812	3.34667	-0.68577
C	6.96813	-1.5378	-0.04952
H	7.94291	-2.03625	-0.12896
H	6.34348	-2.14001	0.61965
H	6.51046	-1.5571	-1.04452
C	7.8819	-0.12599	1.84219
H	8.82529	-0.68023	1.75032
H	8.12883	0.87884	2.19922
H	7.28361	-0.61861	2.6166

Int2-2

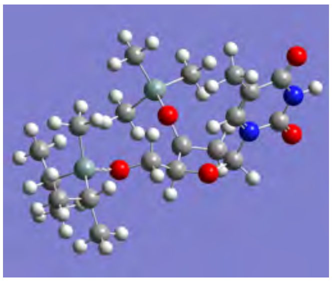

Minimum frequencies (cm $^{-1}$) = 9.5344 11.4538 13.4069

Charge = 0 Multiplicity = 2

C	-0.20231	0.7948	0.
O	1.00236	0.09549	0.22311
C	2.11304	1.02312	0.49289
C	1.43994	2.3389	0.78049
C	0.14321	2.30639	0.00998
H	-0.64608	0.47562	-0.94476
H	2.70434	1.08299	-0.43123
H	4.51491	-0.19036	4.52251

H	-0.65207	2.913	0.44158
H	0.28922	2.63086	-1.02776
C	2.99857	0.41887	1.57924
H	3.06313	-0.66101	1.3868
H	2.54384	0.57451	2.56594
O	4.27572	1.03114	1.51211
Si	5.62991	0.53432	2.39197
C	5.22866	0.59285	4.24111
H	6.12715	0.44842	4.85234
H	4.79153	1.55753	4.52029
C	6.04798	-1.27496	1.93716
H	5.17548	-1.84905	2.29078
C	6.9923	1.78796	1.94435
H	7.94062	1.30839	2.23527
O	1.48461	2.80805	2.05818
Si	1.46476	4.4307	2.59013
C	-0.19982	5.23025	2.2166
H	-0.23864	6.23378	2.65802
H	-0.37484	5.34144	1.14101
H	-1.02985	4.65287	2.63861
C	2.85173	5.36141	1.7276
H	2.6968	5.38977	0.64335
H	2.90584	6.39715	2.08352
H	3.82127	4.88803	1.91398
C	1.74074	4.28268	4.43986
H	1.75732	5.27124	4.91329
H	0.94311	3.69935	4.91266
H	2.69369	3.78919	4.65813
C	-2.51712	0.76477	0.78373
C	-0.84473	-0.22767	2.21882
C	-1.72929	-0.57918	3.1864
H	0.20904	-0.46051	2.29198
C	-3.13919	-0.25456	2.98754
N	-1.19621	0.42921	1.05688
N	-3.41117	0.39762	1.77214
H	-4.38085	0.63313	1.59237
O	-4.05094	-0.50509	3.76952
C	-1.33415	-1.29742	4.4458
H	-1.60614	-0.71344	5.33165
H	-1.85728	-2.25599	4.53051
H	-0.25689	-1.48464	4.46829

O	-2.85749	1.34438	-0.24337
C	6.18943	-1.54378	0.426
H	7.09299	-1.07514	0.0198
H	6.26959	-2.62066	0.22835
H	5.33618	-1.16327	-0.1453
C	7.27666	-1.8069	2.70508
H	7.43553	-2.87276	2.49656
H	8.1929	-1.28186	2.40805
H	7.16931	-1.69982	3.79012
C	6.8742	3.09939	2.74947
H	7.69251	3.78483	2.49362
H	5.93454	3.61829	2.5258
H	6.91295	2.93107	3.83077
C	7.0611	2.09443	0.43349
H	7.87774	2.79645	0.22008
H	7.23306	1.19728	-0.16881
H	6.12864	2.55031	0.08452

TS2-3

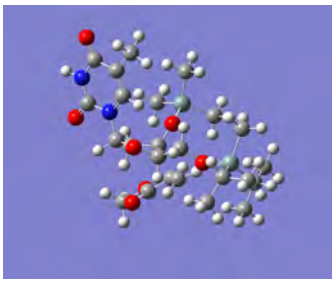

Minimum frequencies (cm $^{-1}$) = -227.3888

Charge = 0 Multiplicity = 2

C	-0.27577	0.11754	0.
O	0.99246	-0.48648	-0.16038
C	2.02197	0.26442	0.56075
C	1.23858	1.09608	1.56203
C	-0.06003	1.39652	0.84373
H	-0.70959	0.33863	-0.97704
H	2.52002	0.91502	-0.16828
H	4.80309	-2.81418	3.02645
H	-0.90143	1.61179	1.49942
H	0.06678	2.24599	0.16573
C	3.04692	-0.72536	1.10012
H	3.26667	-1.44404	0.29857
H	2.62705	-1.27099	1.95415
O	4.20808	-0.00513	1.48315

Si	5.67123	-0.72626	1.93116
C	5.36133	-1.93111	3.35881
H	6.30399	-2.28714	3.79086
H	4.78866	-1.45777	4.16346
C	6.35991	-1.7328	0.45897
H	5.66893	-2.58802	0.37566
C	6.77622	0.71522	2.49988
H	7.7797	0.27968	2.63135
O	1.19933	0.5779	2.81648
Si	0.61445	1.03952	4.35018
C	-0.92692	2.11299	4.23905
H	-1.23607	2.40024	5.25178
H	-0.75377	3.03573	3.67607
H	-1.76899	1.58388	3.78032
C	1.99714	1.94087	5.25418
H	2.23871	2.90401	4.79427
H	1.70362	2.13555	6.29297
H	2.91089	1.33736	5.27377
C	0.24161	-0.59967	5.18642
H	-0.10896	-0.4401	6.21296
H	-0.53528	-1.15459	4.64982
H	1.13531	-1.23143	5.23407
C	-2.55776	-0.54426	0.5756
C	-0.77039	-2.03718	1.2236
C	-1.59683	-2.95388	1.78866
H	0.30075	-2.17995	1.17742
C	-3.03363	-2.69189	1.77832
N	-1.20568	-0.86123	0.64592
N	-3.391	-1.48371	1.15506
H	-4.38172	-1.2717	1.11355
O	-3.89964	-3.42136	2.25089
C	-1.11044	-4.23385	2.40813
H	-1.40582	-4.298	3.46093
H	-1.55062	-5.10264	1.907
H	-0.02127	-4.3101	2.34545
O	-2.97341	0.48523	0.05365
C	6.34271	-1.00134	-0.89774
H	7.07157	-0.18396	-0.92351
H	6.60215	-1.68984	-1.71239
H	5.36177	-0.57162	-1.12555
C	7.75968	-2.31378	0.75265

H	8.08484	-2.97737	-0.05882
H	8.51157	-1.52015	0.84039
H	7.7836	-2.89787	1.67958
C	6.33466	1.2759	3.86868
H	7.00809	2.08005	4.19202
H	5.32445	1.69847	3.81691
H	6.33688	0.51156	4.65291
C	6.88865	1.8542	1.46543
H	7.54145	2.65272	1.84121
H	7.3043	1.51357	0.51236
H	5.90908	2.29813	1.25819
C	2.43469	3.12374	1.61529
H	3.13126	2.63609	2.28705
H	2.59952	2.9918	0.55149
C	1.66488	4.17192	2.03051
C	0.8699	4.9472	1.07195
H	1.59439	4.45958	3.0743
O	0.76655	4.71628	-0.12558
O	0.23411	5.98465	1.67775
C	-0.56849	6.81406	0.81935
H	-1.36087	6.22581	0.34962
H	-0.99165	7.58008	1.4689
H	0.04858	7.2678	0.03972

Int2-4

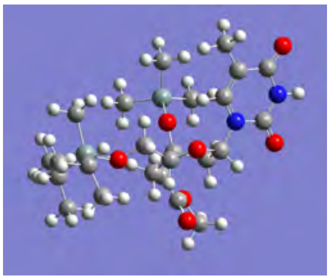

Minimum frequencies (cm $^{-1}$) = 8.5071 13.0718 17.5447

Charge = 0 Multiplicity = 2

C	0.36618	0.53345	0.
O	1.56256	-0.21779	0.04165
C	2.63721	0.57714	0.58149
C	1.9435	1.6095	1.51224
C	0.6584	1.87337	0.70137
H	0.05116	0.68629	-1.03507
H	3.12641	1.11707	-0.24068
H	5.38364	-2.28512	3.33594

H	-0.17368	2.21288	1.31809
H	0.84648	2.63756	-0.05804
C	3.64668	-0.37882	1.20318
H	3.83067	-1.18504	0.47968
H	3.22225	-0.81577	2.1145
O	4.83808	0.34037	1.48996
Si	6.28973	-0.3753	1.98005
C	5.98403	-1.39256	3.54749
H	6.9257	-1.73298	3.99389
H	5.45393	-0.80589	4.3054
C	6.8982	-1.57001	0.61737
H	6.15479	-2.38424	0.62725
C	7.46065	1.08234	2.33937
H	8.46218	0.63398	2.43874
O	1.64251	0.94495	2.7295
Si	1.10225	1.30026	4.28324
C	-0.45912	2.36084	4.27086
H	-0.79394	2.53583	5.30081
H	-0.31207	3.34113	3.80581
H	-1.27747	1.85826	3.7433
C	2.46082	2.12939	5.30191
H	2.72013	3.13689	4.96235
H	2.13754	2.21252	6.34701
H	3.37626	1.52721	5.28806
C	0.73057	-0.38467	5.02928
H	0.39798	-0.28278	6.06912
H	-0.0591	-0.90067	4.47382
H	1.62035	-1.02409	5.02578
C	-2.01715	0.15039	0.34234
C	-0.47889	-1.40012	1.38067
C	-1.4567	-2.17954	1.9092
H	0.57145	-1.62272	1.50896
C	-2.848	-1.80482	1.67217
N	-0.7234	-0.26817	0.62917
N	-3.00468	-0.64503	0.8933
H	-3.95676	-0.35679	0.69714
O	-3.83432	-2.40571	2.08687
C	-1.18056	-3.41704	2.71622
H	-1.616	-3.33918	3.7184
H	-1.63275	-4.29766	2.24743
H	-0.105	-3.58814	2.8149

O	-2.26006	1.14526	-0.33396
C	6.90933	-0.98157	-0.80726
H	7.69322	-0.2249	-0.92277
H	7.1077	-1.76572	-1.54939
H	5.95688	-0.50904	-1.06903
C	8.26317	-2.20432	0.9599
H	8.53813	-2.96341	0.2164
H	9.06324	-1.45402	0.96443
H	8.26173	-2.69444	1.93992
C	7.13314	1.77723	3.67837
H	7.84642	2.58744	3.87737
H	6.13182	2.22373	3.66009
H	7.17495	1.0878	4.52801
C	7.51966	2.12017	1.19929
H	8.22242	2.92596	1.44813
H	7.84552	1.68263	0.25092
H	6.53834	2.57643	1.03103
C	2.83465	2.87738	1.70356
H	3.63652	2.59964	2.39519
H	3.28865	3.10364	0.73445
C	2.13323	4.10033	2.17581
C	1.73202	5.14088	1.24554
H	1.91111	4.26196	3.22425
O	1.90567	5.10555	0.03101
O	1.12715	6.18105	1.87864
C	0.68776	7.25765	1.03124
H	-0.0472	6.89784	0.30666
H	0.23995	7.99012	1.70222
H	1.53524	7.69216	0.49493

Int00

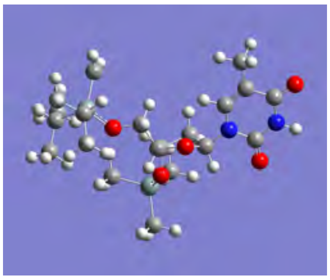

Minimum frequencies (cm $^{-1}$) = 8.9905 10.5200 12.8293

Charge = 0 Multiplicity = 2

C	0.14919	0.13562	0.
O	1.14426	-0.67583	-0.62036

C	2.46146	-0.21323	-0.24685
C	2.25505	0.9585	0.73952
C	0.82838	0.70657	1.24913
H	-0.18289	0.93034	-0.67015
H	2.97726	0.15559	-1.13898
H	3.00445	0.9215	1.53907
H	0.82984	-0.04276	2.04985
H	0.3433	1.6125	1.61622
C	3.22271	-1.40588	0.33106
H	3.12734	-2.24362	-0.37333
H	2.76685	-1.71873	1.2833
O	4.57872	-1.03754	0.52673
Si	5.81271	-2.11937	0.9342
C	5.34431	-3.024	2.49872
H	5.37716	-4.10849	2.59674
H	5.02383	-2.51433	3.40692
C	6.01775	-3.43776	-0.43186
H	5.14158	-4.09448	-0.30666
C	7.33224	-1.01042	1.22991
H	8.16592	-1.70253	1.42534
O	2.3282	2.18163	0.02179
Si	2.88302	3.67041	0.59355
C	4.6521	3.49489	1.22615
H	5.04159	4.46791	1.5494
H	4.71997	2.81738	2.08501
H	5.31643	3.11076	0.44443
C	1.777	4.30151	1.98838
H	2.11363	5.28841	2.32874
H	0.73666	4.40209	1.65902
H	1.79274	3.63573	2.85943
C	2.79496	4.79887	-0.90335
H	1.76981	4.86389	-1.28367
H	3.12744	5.81348	-0.65481
H	3.42936	4.42435	-1.71393
C	-2.28864	-0.14108	-0.00113
C	-0.89935	-1.97437	0.76625
C	-1.94039	-2.7926	1.05236
H	0.12817	-2.29178	0.8944
C	-3.2939	-2.29593	0.80502
N	-1.03415	-0.69494	0.2561
N	-3.34158	-0.99336	0.28299

H	-4.26439	-0.6133	0.10407
O	-4.3308	-2.91622	1.01383
O	-2.45666	0.99447	-0.42723
C	-1.77904	-4.18507	1.59235
H	-2.2919	-4.29341	2.55396
H	-2.22562	-4.92073	0.91492
H	-0.72258	-4.43273	1.72903
C	7.70708	-0.14593	0.00852
H	6.88616	0.52382	-0.26941
H	8.58215	0.47734	0.2346
H	7.95537	-0.74982	-0.86975
C	7.15811	-0.12715	2.48338
H	6.32491	0.57385	2.35995
H	6.96984	-0.72013	3.38465
H	8.0623	0.46837	2.66373
C	7.27194	-4.30893	-0.20537
H	7.30519	-5.13855	-0.92305
H	8.19155	-3.72711	-0.3405
H	7.30237	-4.74559	0.79959
C	5.9762	-2.89304	-1.87327
H	6.85389	-2.2777	-2.09831
H	5.96563	-3.71794	-2.59724
H	5.09034	-2.2772	-2.05901

TS3-1

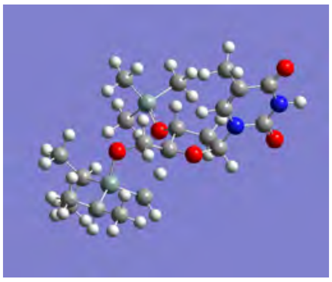

Minimum frequencies (cm $^{-1}$) = -1625.6041

Charge = 0 Multiplicity = 2

C	-0.08658	-0.62771	0.
O	0.87538	-1.42942	0.67698
C	1.99221	-0.65637	1.07668
C	1.51305	0.79286	1.12498
C	0.46714	0.80487	-0.00339
H	-0.24815	-1.01635	-1.00593
H	2.96151	-0.74408	0.15615
H	-0.3169	1.55386	0.11756

H	0.98361	0.99931	-0.94801
C	2.67308	-1.30904	2.27602
H	2.50925	-2.39297	2.20242
H	2.20584	-0.96763	3.21215
O	4.05304	-0.99994	2.36458
Si	5.16411	-1.24657	1.09787
C	4.18246	-0.94962	-0.47001
H	4.33233	-0.00231	-0.99147
H	3.98829	-1.76936	-1.16444
C	6.48164	0.10481	1.34184
H	5.96395	1.01451	1.00017
C	5.85071	-3.0278	1.15224
H	6.55892	-3.07752	0.3092
C	-2.51615	-0.42917	-0.04867
C	-1.49636	-1.17586	2.01696
C	-2.67238	-1.28214	2.68403
H	-0.55251	-1.43641	2.47789
C	-3.90568	-0.95016	1.97175
N	-1.39226	-0.76503	0.70075
N	-3.70948	-0.553	0.63772
H	-4.54201	-0.31454	0.11009
O	-2.44964	-0.0487	-1.21245
O	-5.03719	-0.99783	2.44099
C	-2.77605	-1.73997	4.11127
H	-3.2553	-0.97618	4.73285
H	-3.39535	-2.63994	4.18859
H	-1.78825	-1.95958	4.526
H	1.02347	0.96493	2.10104
O	2.55581	1.72165	0.92648
Si	2.72389	3.19012	1.7523
C	2.91817	2.86452	3.59943
H	3.1254	3.79843	4.13582
H	2.01209	2.43336	4.04091
H	3.746	2.17346	3.79102
C	1.21017	4.27641	1.44827
H	0.29149	3.82096	1.83642
H	1.32525	5.24659	1.94672
H	1.06445	4.46742	0.37912
C	4.26871	3.96324	1.01912
H	4.16527	4.09606	-0.06329
H	4.46108	4.94765	1.4615

H	5.14876	3.33697	1.199
C	7.70695	-0.09977	0.4278
H	8.38575	0.76075	0.4869
H	8.28326	-0.98569	0.72047
H	7.42385	-0.22155	-0.62437
C	6.90392	0.34304	2.80482
H	7.4708	-0.50352	3.20733
H	7.54933	1.22834	2.88048
H	6.03746	0.49804	3.45467
C	4.76625	-4.0978	0.90393
H	4.0555	-4.1444	1.73759
H	4.19475	-3.91612	-0.01248
H	5.21878	-5.09403	0.81511
C	6.63108	-3.35203	2.44251
H	7.00221	-4.38525	2.42167
H	7.49736	-2.69754	2.57985
H	5.9949	-3.25014	3.32952

Int3-2

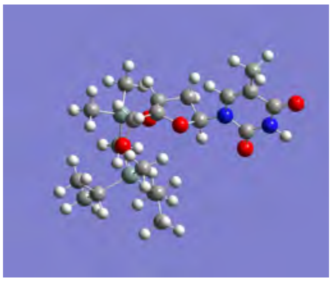

Minimum frequencies (cm $^{-1}$) = 13.5544 16.9295 19.0233

Charge = 0 Multiplicity = 2

C	-0.80018	-0.00904	0.
O	0.00489	-0.90302	0.79979
C	0.93057	-0.17191	1.51643
C	0.88795	1.27529	1.12249
C	-0.54974	1.38763	0.58715
H	-0.4822	-0.08001	-1.04001
H	2.34791	-4.23843	2.61568
H	-1.24711	1.58846	1.40754
H	-0.65128	2.17112	-0.16546
C	2.00765	-0.94664	2.18306
H	1.55882	-1.76751	2.75906
H	2.52083	-0.28551	2.8899
O	3.00979	-1.46347	1.29536
Si	3.05211	-2.97861	0.54545

C	2.04922	-4.22445	1.56177
H	0.9761	-4.00763	1.51589
H	2.19324	-5.23846	1.17012
C	2.27226	-2.88075	-1.19415
H	1.19385	-2.80618	-0.98523
C	4.8929	-3.47967	0.53854
H	4.94146	-4.42049	-0.03275
C	-2.83369	-0.71636	-1.17315
C	-2.80248	-0.70689	1.25782
C	-4.0731	-1.15367	1.38785
H	-2.17508	-0.52325	2.12247
C	-4.8391	-1.41378	0.16673
N	-2.1811	-0.47354	0.03984
N	-4.13214	-1.16803	-1.02165
H	-4.6311	-1.34476	-1.88662
O	-2.31988	-0.53637	-2.26828
O	-5.99603	-1.81426	0.12529
C	-4.73455	-1.41002	2.71169
H	-5.64008	-0.80362	2.81867
H	-5.04652	-2.4564	2.79536
H	-4.05844	-1.17955	3.5397
H	1.05614	1.93326	1.98481
O	1.81202	1.58541	0.06867
Si	3.17099	2.5838	0.1975
C	4.31443	1.97253	1.56709
H	5.2579	2.53192	1.55887
H	3.87175	2.10325	2.56192
H	4.54467	0.90995	1.43584
C	2.61779	4.35098	0.57685
H	2.07721	4.41848	1.52856
H	3.48342	5.02081	0.64874
H	1.96095	4.7389	-0.20993
C	3.99275	2.47956	-1.48645
H	3.30825	2.80301	-2.27813
H	4.88336	3.11752	-1.52934
H	4.30083	1.45271	-1.70881
C	2.49541	-4.17135	-2.01059
H	1.93258	-4.13771	-2.95238
H	3.55263	-4.30367	-2.27152
H	2.17213	-5.06978	-1.47181
C	2.65847	-1.63452	-2.01455

H	3.71094	-1.66215	-2.31968
H	2.0595	-1.57811	-2.93315
H	2.49899	-0.70962	-1.45256
C	5.41001	-3.7724	1.96254
H	5.35584	-2.87708	2.59337
H	4.84032	-4.56564	2.45836
H	6.46052	-4.09082	1.93656
C	5.81101	-2.45689	-0.16105
H	6.85636	-2.79231	-0.13345
H	5.54355	-2.30689	-1.21155
H	5.7651	-1.48144	0.33623

TS3-3

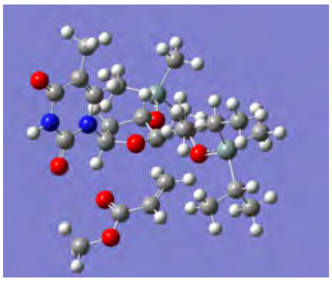

Minimum frequencies (cm $^{-1}$) = -310.5906

Charge = 0 Multiplicity = 2

C	1.72845	-0.39552	0.63164
O	0.31478	-0.45283	0.30864
C	-0.12365	0.76459	-0.14493
C	1.06032	1.48481	-0.77215
C	2.24117	0.93997	0.0583
H	1.84356	-0.45128	1.71073
H	-4.80945	1.85625	-1.32791
H	3.15963	0.81547	-0.51954
H	2.43336	1.62414	0.8879
C	-1.42207	0.70689	-0.89653
H	-1.26668	0.06856	-1.78816
H	-1.64405	1.72179	-1.25581
O	-2.47813	0.20797	-0.10242
Si	-3.93466	-0.41135	-0.69622
C	-4.74667	0.87987	-1.82042
H	-4.18477	1.01179	-2.75278
H	-5.76607	0.58454	-2.09563
C	-3.58146	-1.96392	-1.75411
H	-3.09526	-1.56269	-2.65925
C	-4.99772	-0.71457	0.85121

H	-5.90469	-1.22506	0.48915
C	2.84521	-2.54395	1.02083
C	2.38988	-1.84336	-1.25896
C	2.90479	-2.95811	-1.82691
H	1.9525	-1.05884	-1.86577
C	3.45761	-3.98608	-0.94178
N	2.35984	-1.6005	0.10562
N	3.37692	-3.67379	0.42357
H	3.7422	-4.36804	1.06615
O	2.82235	-2.3925	2.23322
O	3.95773	-5.04304	-1.30733
C	2.93242	-3.20366	-3.30826
H	3.95919	-3.34754	-3.66074
H	2.38342	-4.11661	-3.56185
H	2.48898	-2.36605	-3.85417
H	1.14369	1.13731	-1.82179
O	0.91757	2.88663	-0.76093
Si	1.65213	3.99821	-1.80302
C	1.15468	3.62033	-3.58544
H	1.59139	4.35904	-4.26837
H	1.50073	2.63369	-3.91503
H	0.06669	3.65493	-3.71169
C	3.52954	3.92429	-1.63648
H	3.9378	2.96397	-1.97191
H	3.9988	4.70676	-2.2452
H	3.8423	4.07811	-0.59785
C	0.98181	5.65783	-1.24136
H	1.24915	5.85403	-0.19747
H	1.38655	6.47353	-1.85164
H	-0.11034	5.68895	-1.31888
C	-4.87739	-2.66448	-2.21525
H	-4.65209	-3.48633	-2.90708
H	-5.4201	-3.098	-1.36655
H	-5.56086	-1.98154	-2.73256
C	-2.60302	-2.97363	-1.12133
H	-3.05705	-3.49132	-0.26948
H	-2.31755	-3.74392	-1.84995
H	-1.68776	-2.49461	-0.75966
C	-5.44329	0.60939	1.50893
H	-4.58128	1.18034	1.87281
H	-6.00284	1.25079	0.81978

H	-6.09148	0.41141	2.37227
C	-4.32329	-1.63012	1.89378
H	-4.97324	-1.76027	2.76882
H	-4.10702	-2.62629	1.49632
H	-3.37748	-1.20259	2.24308
C	-0.7018	2.05003	1.70208
H	-1.67824	2.19905	1.25776
H	0.07467	2.7627	1.45086
C	-0.60133	1.29389	2.83954
C	0.64879	1.21057	3.59511
H	-1.43051	0.69087	3.19349
O	1.72345	1.705	3.26588
O	0.49761	0.48853	4.7348
C	1.68387	0.30337	5.52614
H	1.36293	-0.26432	6.39948
H	2.43422	-0.25578	4.96125
H	2.10096	1.26837	5.82516

Int3-4

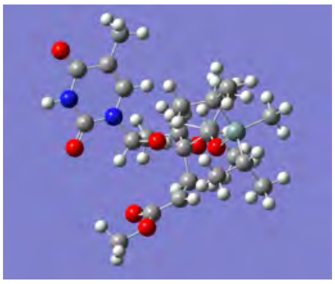

Minimum frequencies (cm $^{-1}$) = 6.6020 12.4523 14.7464

Charge = 0 Multiplicity = 2

C	1.80302	-0.45547	0.92377
O	0.43706	-0.41567	0.48416
C	-0.02172	0.94176	0.36316
C	1.25852	1.70055	-0.085
C	2.37616	0.95668	0.67223
H	1.85442	-0.73658	1.97533
H	-4.16852	2.28452	-2.0345
H	3.32048	0.92637	0.12362
H	2.55746	1.45743	1.62669
C	-1.08173	0.9916	-0.73793
H	-0.67816	0.45553	-1.60988
H	-1.21758	2.04703	-1.01802
O	-2.31664	0.43921	-0.3232
Si	-3.53705	-0.05997	-1.38352

C	-3.93937	1.35887	-2.57358
H	-3.10207	1.56623	-3.25042
H	-4.8079	1.11784	-3.19784
C	-2.90923	-1.53713	-2.42033
H	-2.20232	-1.08173	-3.13392
C	-5.04489	-0.43161	-0.28486
H	-5.8431	-0.7158	-0.98969
C	2.99453	-2.6123	0.91493
C	2.52784	-1.50906	-1.19216
C	3.09497	-2.47131	-1.9571
H	2.05367	-0.64494	-1.64226
C	3.68407	-3.62664	-1.27832
N	2.47738	-1.53275	0.19105
N	3.57725	-3.58637	0.11907
H	3.96605	-4.3754	0.62337
O	2.95895	-2.70068	2.13316
O	4.23229	-4.57323	-1.83218
C	3.14068	-2.42559	-3.45773
H	4.17424	-2.45752	-3.81846
H	2.6316	-3.29339	-3.89034
H	2.66539	-1.5167	-3.83793
H	1.37258	1.54481	-1.17006
O	1.17686	3.07904	0.19643
Si	1.99595	4.33973	-0.57556
C	1.65929	4.27436	-2.43246
H	2.14902	5.11558	-2.93764
H	2.04129	3.35632	-2.894
H	0.58664	4.3383	-2.64731
C	3.8518	4.21434	-0.2614
H	4.28692	3.31275	-0.70756
H	4.37356	5.07609	-0.69514
H	4.07302	4.19779	0.81156
C	1.28005	5.89241	0.19755
H	1.44381	5.90083	1.28058
H	1.74633	6.79234	-0.21996
H	0.20101	5.95936	0.02159
C	-4.0351	-2.18289	-3.25634
H	-3.63138	-2.95562	-3.92309
H	-4.78041	-2.66862	-2.61504
H	-4.56152	-1.45439	-3.88387
C	-2.12814	-2.60148	-1.62328

H	-2.78354	-3.15541	-0.94279
H	-1.67585	-3.33584	-2.30286
H	-1.3283	-2.16306	-1.01894
C	-5.5244	0.82658	0.47051
H	-4.76524	1.17691	1.17891
H	-5.75546	1.6564	-0.20598
H	-6.43381	0.60859	1.04509
C	-4.84761	-1.60693	0.69411
H	-5.75181	-1.76029	1.29745
H	-4.63782	-2.54768	0.17617
H	-4.01998	-1.42098	1.38721
C	-0.5523	1.49305	1.72743
H	-1.17421	2.36767	1.50842
H	0.29753	1.83531	2.32264
C	-1.31945	0.4977	2.52096
C	-0.68162	-0.21154	3.61081
H	-2.33846	0.24425	2.25915
O	0.48032	-0.04087	3.97608
O	-1.51532	-1.10682	4.20422
C	-0.94134	-1.87335	5.27712
H	-1.73444	-2.54177	5.61201
H	-0.07917	-2.44309	4.9213
H	-0.62309	-1.2157	6.09051

TS3-5

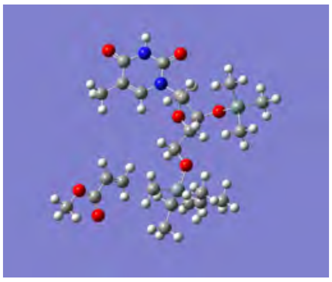

Minimum frequencies (cm $^{-1}$) = -308.9554

Charge = 0 Multiplicity = 2

C	3.10659	0.49859	-0.44032
O	1.99761	0.29384	-1.31403
C	1.23117	-0.8471	-0.86993
C	1.86627	-1.32083	0.45879
C	2.65508	-0.08011	0.90413
H	3.99871	-0.00906	-0.81132
H	1.31553	-1.64833	-1.61096
H	1.08624	-1.60695	1.17439

H	1.99838	0.63379	1.416
H	3.4909	-0.32322	1.56213
C	-0.22807	-0.40561	-0.75661
H	-0.50579	0.09352	-1.6954
H	-0.33082	0.32925	0.05794
O	-1.0354	-1.541	-0.50491
Si	-2.71828	-1.61766	-0.62358
C	-3.48201	-0.42507	0.60547
C	-3.27538	-1.13965	-2.38699
H	-3.02612	-0.07047	-2.47356
C	-3.15016	-3.40558	-0.10811
H	-4.2243	-3.51805	-0.32332
O	2.7373	-2.40862	0.18829
Si	3.1199	-3.69227	1.21782
C	1.54056	-4.57567	1.75214
H	1.78052	-5.44267	2.37956
H	0.87934	-3.92599	2.33704
H	0.9746	-4.93542	0.88604
C	4.03925	-3.07029	2.74577
H	4.32742	-3.91144	3.3881
H	4.95506	-2.53469	2.47136
H	3.42229	-2.39661	3.35212
C	4.21473	-4.80431	0.17569
H	5.10862	-4.26794	-0.16058
H	4.54236	-5.68267	0.74388
H	3.68154	-5.15678	-0.71381
C	4.77933	2.2916	-0.46137
C	2.42866	2.8801	-0.35996
C	2.6486	4.21609	-0.3211
H	1.42806	2.46589	-0.37012
C	4.03353	4.68661	-0.357
N	3.43198	1.9291	-0.43027
N	4.98779	3.65947	-0.43387
H	5.95956	3.94814	-0.45508
O	4.38907	5.85949	-0.32701
O	5.6997	1.48507	-0.49779
C	1.54995	5.2387	-0.2604
H	1.64984	5.86757	0.63063
H	1.59396	5.9107	-1.12407
H	0.56732	4.7589	-0.24204
C	-2.38649	-4.47723	-0.91307

H	-1.30436	-4.37258	-0.78101
H	-2.66767	-5.48218	-0.57181
H	-2.59615	-4.42609	-1.98531
C	-2.95298	-3.63144	1.40591
H	-1.90169	-3.50803	1.69039
H	-3.54734	-2.93735	2.00928
H	-3.24889	-4.65104	1.6844
C	-4.80417	-1.26416	-2.56401
H	-5.11163	-0.89421	-3.55028
H	-5.12959	-2.30922	-2.49639
H	-5.363	-0.69312	-1.81445
C	-2.52818	-1.86668	-3.52312
H	-2.80314	-2.92547	-3.57338
H	-2.78081	-1.42238	-4.49433
H	-1.44051	-1.81516	-3.40728
H	-4.56409	-0.3611	0.71214
H	-2.96415	-0.20852	1.53919
C	-3.42904	1.81938	-0.19026
H	-2.37298	1.75663	-0.42376
H	-4.13294	1.51571	-0.95656
C	-3.85965	2.58309	0.85256
H	-3.17075	3.01899	1.56906
C	-5.29672	2.79431	1.08626
O	-6.20348	2.30192	0.43233
O	-5.50787	3.61751	2.14434
C	-6.8842	3.89932	2.45425
H	-6.85698	4.56697	3.31501
H	-7.37533	4.38275	1.60605
H	-7.41779	2.97706	2.69753

Int3-6

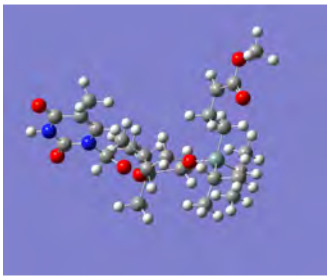

Minimum frequencies (cm $^{-1}$) = 5.7138 7.9751 12.6194

Charge = 0 Multiplicity = 2

C	-3.21084	0.41751	0.39728
O	-2.09751	0.28996	1.27917

C	-1.25221	-0.79864	0.84658
C	-1.85311	-1.33029	-0.47553
C	-2.71832	-0.14824	-0.93837
H	-4.07345	-0.13792	0.76967
H	-1.27867	-1.59464	1.59747
H	-1.05463	-1.57505	-1.18619
H	-2.10677	0.6	-1.45713
H	-3.53471	-0.45193	-1.59556
C	0.17214	-0.25676	0.72348
H	0.41448	0.28059	1.65016
H	0.22141	0.46723	-0.10531
O	1.05838	-1.33681	0.49128
Si	2.73281	-1.35439	0.72353
C	3.57932	-0.23681	-0.5729
C	3.15319	-0.69931	2.46747
H	2.85165	0.36033	2.44664
C	3.24461	-3.15976	0.39742
H	4.29045	-3.23172	0.73562
O	-2.65326	-2.46819	-0.19214
Si	-2.94981	-3.7854	-1.20733
C	-1.31566	-4.57712	-1.7208
H	-1.49779	-5.46357	-2.34043
H	-0.69054	-3.89449	-2.30786
H	-0.73582	-4.89279	-0.8467
C	-3.89488	-3.23962	-2.74869
H	-4.12732	-4.10464	-3.38183
H	-4.84327	-2.7572	-2.48654
H	-3.316	-2.53725	-3.36002
C	-3.98153	-4.94971	-0.15793
H	-4.90975	-4.46571	0.16448
H	-4.24926	-5.85428	-0.7163
H	-3.43459	-5.25638	0.74015
C	-4.98773	2.10664	0.36346
C	-2.67542	2.83602	0.3107
C	-2.97523	4.15585	0.25373
H	-1.65213	2.48348	0.34798
C	-4.38642	4.54164	0.25371
N	-3.6208	1.82665	0.36775
N	-5.27825	3.45896	0.31878
H	-6.26596	3.68814	0.3148
O	-4.81184	5.69042	0.2044

O	-5.85786	1.24545	0.38527
C	-1.93913	5.24259	0.20833
H	-2.05595	5.85702	-0.69069
H	-2.04373	5.91763	1.06439
H	-0.92929	4.82273	0.21693
C	2.41591	-4.18315	1.2015
H	1.35482	-4.12337	0.93719
H	2.75609	-5.20461	0.98731
H	2.49581	-4.03147	2.28185
C	3.21857	-3.51812	-1.10441
H	2.20542	-3.43566	-1.51483
H	3.87584	-2.87652	-1.70027
H	3.54757	-4.55403	-1.25686
C	4.67278	-0.72783	2.74292
H	4.89807	-0.26739	3.7131
H	5.05329	-1.75594	2.77867
H	5.24826	-0.18421	1.98598
C	2.37448	-1.36518	3.62007
H	2.69446	-2.4012	3.77462
H	2.55072	-0.8305	4.56216
H	1.29369	-1.37849	3.44429
H	4.642	-0.51277	-0.6144
H	3.16579	-0.49222	-1.55764
C	3.4755	1.30272	-0.35
H	2.41846	1.59831	-0.34455
H	3.90434	1.5671	0.62072
C	4.19036	2.0588	-1.4137
H	3.7532	2.17137	-2.40185
C	5.52635	2.57964	-1.20037
O	6.16324	2.48076	-0.15524
O	6.01382	3.20554	-2.30774
C	7.33429	3.75862	-2.18033
H	7.55108	4.21803	-3.14451
H	7.36217	4.50547	-1.38251
H	8.05986	2.97212	-1.95644

TS3-3'

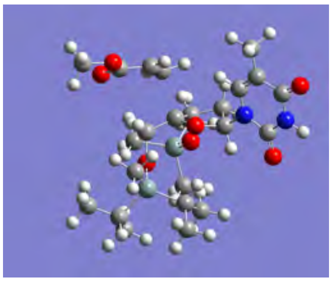

Minimum frequencies (cm $^{-1}$) = -286.2653

Charge = 0 Multiplicity = 2

C	-0.0859	0.46112	0.
O	0.57348	1.68216	0.41141
C	1.48697	2.08208	-0.53732
C	1.55688	1.03275	-1.64238
C	0.17917	0.36118	-1.50599
H	0.3561	-0.37262	0.54501
H	3.04687	4.41521	2.87545
H	-0.58427	0.90619	-2.07079
H	0.1971	-0.67376	-1.85052
C	2.66402	2.82425	0.03844
H	2.28694	3.69737	0.5811
H	3.28319	3.19572	-0.78338
O	3.47663	2.01624	0.87988
Si	3.49953	1.95251	2.57011
C	2.64431	3.48283	3.28565
H	1.56553	3.46744	3.0944
H	2.78323	3.51974	4.37268
C	2.54451	0.4101	3.1607
H	1.49607	0.66558	2.94123
C	5.34413	1.95144	3.05976
H	5.36238	1.76932	4.14631
C	-1.97734	-0.53008	1.19559
C	-2.27947	1.59571	0.05512
C	-3.58035	1.72552	0.40307
H	-1.76816	2.36196	-0.51436
C	-4.1861	0.66281	1.20932
N	-1.482	0.51649	0.4083
N	-3.30846	-0.38312	1.53851
H	-3.69388	-1.13324	2.1018
O	-1.30897	-1.4944	1.54011
O	-5.34843	0.63751	1.59382
C	-4.43219	2.902	0.0211
H	-5.30717	2.57863	-0.55234

H	-4.81253	3.41294	0.91183
H	-3.86618	3.6201	-0.57858
H	1.68874	1.50079	-2.6258
O	2.57512	0.0727	-1.39667
Si	3.962	-0.19974	-2.33049
C	5.04129	1.34341	-2.39586
H	5.97758	1.13143	-2.92654
H	4.55392	2.17244	-2.92234
H	5.29288	1.68341	-1.38599
C	3.44089	-0.68573	-4.08075
H	2.87399	0.10879	-4.58053
H	4.32185	-0.89118	-4.70098
H	2.82048	-1.58907	-4.07534
C	4.83482	-1.61342	-1.46055
H	4.19421	-2.50032	-1.40768
H	5.75361	-1.89195	-1.98987
H	5.10538	-1.33273	-0.43753
C	2.65388	0.1928	4.68456
H	2.00255	-0.62963	5.00716
H	3.67692	-0.07128	4.97959
H	2.3626	1.08139	5.25657
C	2.86418	-0.88958	2.39553
H	3.85992	-1.27003	2.65079
H	2.14318	-1.67593	2.65347
H	2.83414	-0.74543	1.31112
C	6.00398	3.32322	2.80631
H	5.986	3.5809	1.74053
H	5.5079	4.13215	3.35296
H	7.05584	3.31199	3.12102
C	6.16366	0.8345	2.38205
H	7.21535	0.88175	2.69444
H	5.79284	-0.1645	2.63065
H	6.13958	0.93599	1.29117
C	0.5749	3.82131	-1.73345
H	1.47417	3.9105	-2.33584
H	-0.19043	3.14558	-2.09838
C	0.21782	4.87423	-0.92822
C	1.1431	5.98085	-0.67469
H	-0.74747	4.91734	-0.43433
O	0.57475	6.93073	0.11609
O	2.28303	6.07628	-1.1105

C	1.39231	8.07786	0.4021
H	0.77869	8.72225	1.03143
H	2.30244	7.7794	0.92876
H	1.66856	8.59178	-0.52222

Int3-4'

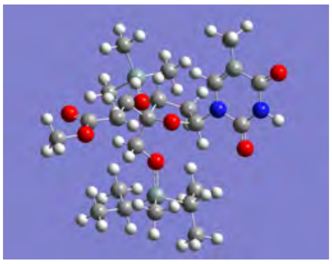

Minimum frequencies (cm $^{-1}$) = 9.7907 13.6240 14.6954

Charge = 0 Multiplicity = 2

C	-1.50406	-0.76415	0.1439
O	-0.99757	0.39638	0.8175
C	0.0495	1.00953	0.04536
C	-0.38855	0.73998	-1.42132
C	-1.09583	-0.62673	-1.33951
H	-1.09927	-1.6673	0.59303
H	2.42972	-0.82493	3.22372
H	-1.95802	-0.69914	-2.0076
H	-0.3834	-1.41007	-1.60028
C	1.41884	0.40646	0.40435
H	1.62953	0.67781	1.44818
H	2.17914	0.87111	-0.23534
O	1.42257	-1.00016	0.23892
Si	2.45212	-2.08173	1.03784
C	2.25201	-1.85782	2.90545
H	1.23798	-2.13191	3.21764
H	2.9552	-2.49086	3.45908
C	1.78135	-3.79279	0.543
H	0.73462	-3.76422	0.88447
C	4.27469	-1.78584	0.53305
H	4.79088	-2.72989	0.77295
C	-3.46904	-1.94417	1.0382
C	-3.76204	0.23713	0.02249
C	-5.09913	0.28409	0.22998
H	-3.23567	1.06319	-0.44151
C	-5.72938	-0.85867	0.89294

N	-2.94997	-0.82334	0.38561
N	-4.83755	-1.88453	1.24147
H	-5.23891	-2.6903	1.7081
O	-2.79655	-2.9034	1.39278
O	-6.92393	-0.96393	1.14725
C	-5.96553	1.44413	-0.16958
H	-6.75177	1.12451	-0.8617
H	-6.47133	1.87189	0.70252
H	-5.37429	2.229	-0.64993
H	-1.12233	1.51785	-1.68446
O	0.70235	0.7903	-2.31129
Si	0.67117	1.239	-3.93975
C	-0.13026	2.93899	-4.12122
H	-0.10292	3.26068	-5.16936
H	-1.18197	2.94183	-3.81205
H	0.39723	3.69487	-3.52869
C	-0.29365	-0.0297	-4.95002
H	-1.34822	-0.07511	-4.65426
H	-0.26527	0.22323	-6.01688
H	0.12997	-1.03358	-4.8347
C	2.47814	1.27649	-4.44412
H	2.94876	0.30012	-4.28628
H	2.58619	1.53473	-5.50402
H	3.03536	2.0162	-3.85922
C	2.48105	-4.94063	1.30086
H	2.01115	-5.90354	1.06351
H	3.53933	-5.02201	1.02228
H	2.43098	-4.8142	2.38771
C	1.76264	-4.07554	-0.97198
H	2.77599	-4.21515	-1.36607
H	1.20516	-4.99673	-1.18553
H	1.29492	-3.26354	-1.53785
C	4.96299	-0.67293	1.35272
H	4.50096	0.30582	1.17463
H	4.92542	-0.86524	2.42985
H	6.02026	-0.58293	1.07113
C	4.4551	-1.52695	-0.97764
H	5.5187	-1.41516	-1.22631
H	4.05672	-2.33991	-1.59162
H	3.94729	-0.6071	-1.28849
C	0.05293	2.51895	0.37027

H	0.79925	3.02481	-0.25032
H	-0.93432	2.90963	0.07587
C	0.28334	2.84984	1.80202
C	1.2591	3.84668	2.19722
H	-0.30657	2.36609	2.57357
O	1.27519	4.0353	3.54497
O	1.995	4.4589	1.42812
C	2.2154	5.00971	4.02732
H	2.08641	5.0264	5.10928
H	3.2365	4.72037	3.76506
H	2.003	5.99259	3.59849

TS4-1

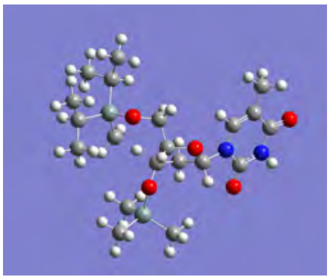

Minimum frequencies (cm $^{-1}$) = -1588.2203

Charge = 0 Multiplicity = 2

C	-0.11544	0.42569	0.
O	0.65148	-0.29167	-0.95138
C	2.07476	-0.10145	-0.69878
C	2.16161	0.84121	0.50996
C	0.81613	0.64925	1.20163
H	-0.45508	1.37986	-0.41285
H	2.52409	0.37955	-1.5724
H	3.14146	0.39709	1.29368
H	0.83195	-0.25457	1.82257
H	0.50194	1.48758	1.82262
C	2.69057	-1.49308	-0.50553
H	2.38167	-2.12124	-1.34903
H	2.2838	-1.95231	0.40954
O	4.10455	-1.43195	-0.48724
Si	5.01729	-1.35561	0.93912
C	4.21285	-0.07532	2.05361
H	3.73274	-0.42438	2.9719
H	4.75174	0.85738	2.23378
C	4.93524	-3.04907	1.82221
H	3.92305	-3.07081	2.25961

C	6.74799	-0.80969	0.37234
H	7.3518	-0.74368	1.29126
O	2.47718	2.13014	0.10682
Si	2.37448	3.65014	0.86121
C	2.75131	3.52056	2.70514
H	2.72217	4.52004	3.15613
H	2.02504	2.90121	3.24228
H	3.74779	3.10478	2.8875
C	0.6571	4.3703	0.58244
H	0.59491	5.37801	1.01167
H	0.44176	4.45576	-0.48882
H	-0.13987	3.76986	1.03421
C	3.67509	4.67651	-0.02247
H	3.48618	4.69879	-1.10127
H	3.67257	5.71119	0.34001
H	4.67859	4.26597	0.1327
C	-2.37255	0.36863	0.90428
C	-1.43617	-1.70465	0.07024
C	-2.53146	-2.44454	0.37488
H	-0.5641	-2.13024	-0.40886
C	-3.67489	-1.77139	0.988
N	-1.33782	-0.34569	0.30911
N	-3.48914	-0.3922	1.19103
H	-4.25801	0.10788	1.62339
O	-4.72875	-2.30492	1.31679
O	-2.29618	1.56687	1.15911
C	-2.63676	-3.91769	0.09877
H	-2.82033	-4.47668	1.02254
H	-3.47888	-4.13065	-0.56833
H	-1.72095	-4.29623	-0.3636
C	7.43517	-1.81502	-0.57498
H	6.84447	-1.96972	-1.4847
H	8.42141	-1.44217	-0.88097
H	7.58765	-2.79188	-0.10541
C	6.71527	0.59349	-0.26978
H	6.12065	0.59086	-1.18977
H	6.28903	1.3498	0.398
H	7.72979	0.92096	-0.53151
C	5.93445	-3.13765	2.99494
H	5.78627	-4.06216	3.56772
H	6.97082	-3.14238	2.63623

H	5.83033	-2.29896	3.69315
C	5.05389	-4.27429	0.89484
H	6.0583	-4.36192	0.4669
H	4.85689	-5.20087	1.44992
H	4.3475	-4.22732	0.05983

Int4-2

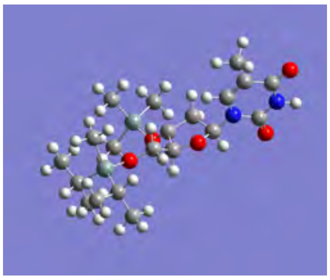

Minimum frequencies (cm $^{-1}$) = 4.9578 11.7325 16.2758

Charge = 0 Multiplicity = 2

C	-0.11544	-0.32468	0.
O	0.87552	-1.34618	0.11641
C	2.17399	-0.72573	0.17638
C	1.90995	0.64927	0.7453
C	0.41766	0.87035	0.81818
H	-0.26678	-0.04519	-1.04541
H	2.60698	-0.63606	-0.83511
H	5.40979	-1.59756	3.78943
H	0.02378	0.83862	1.84449
H	0.11377	1.82486	0.37662
C	3.06368	-1.67226	0.99268
H	2.9123	-2.68757	0.59873
H	2.73776	-1.65976	2.04271
O	4.41449	-1.26664	0.87531
Si	5.74446	-2.17987	1.37389
C	5.60533	-2.51975	3.23163
H	4.79135	-3.22108	3.45019
H	6.52749	-2.95753	3.63139
C	5.73748	-3.8634	0.46768
H	4.83345	-4.36812	0.84669
C	7.25759	-1.08876	0.98915
H	8.12645	-1.76537	1.02374
O	2.775	1.63991	0.41311
Si	3.19786	3.03315	1.29763
C	3.71058	2.51887	3.0335
H	4.04935	3.3862	3.61261

H	2.87642	2.06205	3.57787
H	4.52869	1.79186	3.0029
C	1.74041	4.22724	1.37266
H	2.04684	5.16476	1.8528
H	1.37285	4.47462	0.3705
H	0.90233	3.82363	1.95134
C	4.61845	3.76494	0.31692
H	4.30325	4.01667	-0.70155
H	4.9921	4.68113	0.78878
H	5.45049	3.05682	0.24497
C	-2.51354	-0.70492	-0.35395
C	-1.45958	-1.5539	1.66203
C	-2.59672	-2.08927	2.16618
H	-0.50876	-1.64629	2.17329
C	-3.82656	-1.952	1.38522
N	-1.38776	-0.88113	0.4531
N	-3.66482	-1.26356	0.17174
H	-4.49787	-1.14851	-0.39473
O	-4.92858	-2.37974	1.70975
O	-2.49919	-0.10561	-1.42129
C	-2.66062	-2.82638	3.47341
H	-3.37108	-2.34912	4.15666
H	-3.01041	-3.85362	3.32547
H	-1.67896	-2.85759	3.95439
C	7.20476	-0.45643	-0.41753
H	6.3329	0.19822	-0.5195
H	8.10299	0.1474	-0.60218
H	7.14513	-1.20672	-1.21173
C	7.48197	0.00299	2.05662
H	6.6377	0.70162	2.09116
H	7.61104	-0.41386	3.06093
H	8.38056	0.58997	1.82615
C	6.94166	-4.7461	0.85932
H	6.86114	-5.74075	0.40218
H	7.88718	-4.30976	0.51443
H	7.01828	-4.89091	1.94265
C	5.6009	-3.77325	-1.06492
H	6.50661	-3.36083	-1.52364
H	5.44468	-4.76878	-1.50043
H	4.76081	-3.14093	-1.3706

TS4-3

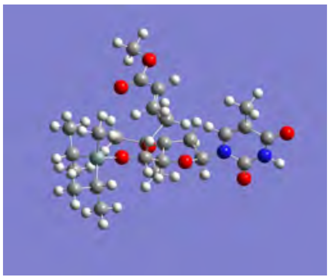

Minimum frequencies (cm $^{-1}$) = -293.2714

Charge = 0 Multiplicity = 2

C	0.41139	1.43761	0.
O	-0.51953	0.50258	-0.54332
C	-1.83111	1.04275	-0.3348
C	-1.65274	2.56234	-0.48214
C	-0.1582	2.83016	-0.3433
H	0.50245	1.30666	1.08143
H	-2.1511	0.86839	0.70848
H	-4.99729	0.76813	-4.0386
H	0.28312	3.21979	-1.26707
H	0.0638	3.54803	0.45025
C	-2.77861	0.25793	-1.24581
H	-2.6813	-0.80177	-0.96926
H	-2.45766	0.35338	-2.29034
O	-4.09865	0.72428	-1.06762
Si	-5.48268	-0.01327	-1.70494
C	-5.28426	-0.18417	-3.57998
H	-4.51994	-0.92636	-3.83929
H	-6.2208	-0.50512	-4.05097
C	-5.64529	-1.77317	-0.9741
H	-4.78831	-2.31964	-1.40184
C	-6.90714	1.16904	-1.27133
H	-7.83139	0.59642	-1.45081
O	-2.55674	3.2947	0.18562
Si	-2.65589	4.75976	1.05792
C	-4.42658	5.31497	0.82444
H	-4.6187	6.24456	1.37359
H	-4.63419	5.48668	-0.23669
H	-5.12643	4.55662	1.19102
C	-1.43499	6.05551	0.43685
H	-1.6859	7.01996	0.89628
H	-0.39888	5.83015	0.70986
H	-1.48014	6.18785	-0.64791

C	-2.28059	4.33789	2.85581
H	-1.25674	3.96932	2.98702
H	-2.39411	5.22525	3.49066
H	-2.96562	3.56998	3.23133
C	2.81057	1.08019	0.33518
C	1.8946	0.95213	-1.91162
C	3.08437	0.69014	-2.50199
H	0.97374	1.00156	-2.47987
C	4.27108	0.60131	-1.64928
N	1.73021	1.14288	-0.54827
N	4.01609	0.80429	-0.28256
H	4.81746	0.75382	0.33675
O	5.41082	0.37273	-2.03636
O	2.71199	1.26202	1.54189
C	3.2503	0.47232	-3.97879
H	3.95072	1.19882	-4.40401
H	3.66656	-0.52006	-4.18182
H	2.2928	0.56281	-4.49915
C	-6.90112	1.60772	0.20805
H	-5.97845	2.14505	0.45003
H	-7.74438	2.28048	0.41198
H	-6.98243	0.7612	0.89702
C	-6.93828	2.40545	-2.19628
H	-6.03248	3.01261	-2.08934
H	-7.03499	2.13224	-3.25239
H	-7.7917	3.04892	-1.94608
C	-6.92284	-2.4923	-1.45727
H	-6.94089	-3.53297	-1.10864
H	-7.82623	-2.00677	-1.06834
H	-7.0018	-2.51366	-2.54993
C	-5.51794	-1.85578	0.55972
H	-6.38516	-1.40911	1.05863
H	-5.46052	-2.90153	0.88906
H	-4.6257	-1.34042	0.93064
C	-2.09398	2.99699	-2.6936
H	-3.13472	2.7719	-2.48783
H	-1.4428	2.16977	-2.95506
C	-1.73718	4.26151	-3.08192
C	-2.68522	5.37416	-3.03978
O	-3.84511	5.32777	-2.64986
O	-2.12019	6.52147	-3.51459

C	-2.98331	7.66832	-3.57187
H	-3.33838	7.93552	-2.57301
H	-2.37395	8.47051	-3.98824
H	-3.84585	7.46823	-4.21292
H	-0.73919	4.48632	-3.44555

Int4-4

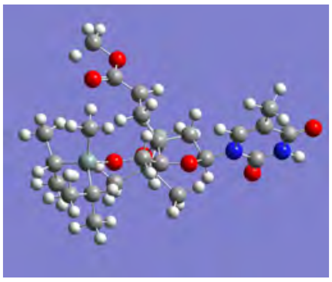

Minimum frequencies (cm $^{-1}$) = 6.7787 10.7294 13.2341

Charge = 0 Multiplicity = 2

C	-0.11343	0.32668	0.
O	-1.06233	-0.69641	-0.24993
C	-2.36322	-0.10093	-0.10608
C	-2.2236	1.40822	-0.5592
C	-0.6835	1.55792	-0.71383
H	0.00404	0.50219	1.0727
H	-2.63885	-0.07131	0.95605
H	-5.81225	-0.8553	-3.41827
H	-0.39967	1.51257	-1.77067
H	-0.29746	2.49146	-0.30269
C	-3.34287	-1.0232	-0.82917
H	-3.15267	-2.04386	-0.46764
H	-3.14625	-1.01761	-1.91089
O	-4.66381	-0.60868	-0.54637
Si	-6.07179	-1.43533	-0.99522
C	-6.01773	-1.77294	-2.85659
H	-5.24445	-2.5072	-3.11227
H	-6.97361	-2.17002	-3.2173
C	-6.11527	-3.11527	-0.08386
H	-5.218	-3.64477	-0.44533
C	-7.49682	-0.26105	-0.53468
H	-8.38582	-0.9043	-0.43247
O	-2.79554	2.21644	0.45392
Si	-2.34573	3.37324	1.58952
C	-3.96491	3.79093	2.44629
H	-3.81486	4.5501	3.22297

H	-4.69883	4.17805	1.73129
H	-4.39778	2.90344	2.92032
C	-1.63162	4.9182	0.7678
H	-1.47879	5.699	1.52328
H	-0.6613	4.74125	0.29049
H	-2.31298	5.32028	0.00997
C	-1.10291	2.68722	2.83298
H	-0.11919	2.47768	2.39879
H	-0.9488	3.41769	3.63719
H	-1.47192	1.76563	3.29809
C	2.31722	0.31534	0.21712
C	1.3066	-0.88476	-1.63511
C	2.48844	-1.2913	-2.15809
H	0.356	-1.1606	-2.07472
C	3.71941	-0.90027	-1.47042
N	1.19505	-0.12144	-0.4843
N	3.51374	-0.12394	-0.31666
H	4.34624	0.17696	0.178
O	4.85671	-1.19224	-1.82135
O	2.25469	1.03074	1.21065
C	2.6016	-2.13403	-3.39647
H	3.19633	-1.62494	-4.16226
H	3.11211	-3.07848	-3.18014
H	1.61441	-2.3574	-3.81041
C	-7.27317	0.45274	0.81581
H	-6.38725	1.09442	0.77619
H	-8.13594	1.08591	1.06063
H	-7.13368	-0.24931	1.64387
C	-7.79698	0.77137	-1.64313
H	-6.94323	1.43372	-1.82553
H	-8.05636	0.29593	-2.59499
H	-8.64422	1.4065	-1.35343
C	-7.33565	-3.97156	-0.48281
H	-7.29155	-4.95969	-0.00673
H	-8.27506	-3.50335	-0.16379
H	-7.3959	-4.13313	-1.56463
C	-5.99913	-3.0105	1.44949
H	-6.89839	-2.56226	1.88757
H	-5.88269	-4.00468	1.90057
H	-5.14317	-2.40161	1.7592
C	-2.97521	1.71599	-1.87303

H	-4.03435	1.48145	-1.74708
H	-2.59385	1.03807	-2.65624
C	-2.82908	3.11923	-2.34049
C	-3.93379	3.817	-2.96831
O	-5.04943	3.34584	-3.165
O	-3.59084	5.08537	-3.33031
C	-4.62709	5.85343	-3.96462
H	-5.48653	5.96241	-3.29788
H	-4.18011	6.8243	-4.17754
H	-4.95317	5.36833	-4.88846
H	-1.88651	3.65022	-2.25086

■ References ■

(1) Monos, T. M.; Sun, A. C.; McAtee, R. C.; Devery, J. J., 3rd; Stephenson, C. R. J. Microwave-Assisted Synthesis of Heteroleptic Ir(III)(+) Polypyridyl Complexes. *J. Org. Chem.* **2016**, *81*, 6988–6994.

(2) Sun, Y.; Zhang, N.; Ren, J.; Huang, H.; Luan, X.; Zuo, Z. Highly Selective 1,4-Diacylation/Cycloisomerization of 1,3-Enynes: De Novo Synthetic Strategy to Polysubstituted Furans. *Org. Lett.* **2023**, *26*, 35–40.

(3) Schrempp, M.; Wagner, R.; Gleich, H.; Gansäuer, A.; Menche, D. Quaternary Carbon Synthesis by Titanocene Catalyzed Radical Allyl Transfer on Epoxides. *Org. Lett.* **2023**, *25*, 8089–8094.

(4) Parasram, M.; Iaroshenko, V. O.; Gevorgyan, V. Endo-Selective Pd-Catalyzed Silyl Methyl Heck Reaction. *J. Am. Chem. Soc.* **2014**, *136*, 17926–17929.

(5) Wu, J.; Bär, R. M.; Guo, L.; Noble, A.; Aggarwal, V. K. Photoinduced Deoxygenative Borylations of Aliphatic Alcohols. *Angew. Chem. Int. Ed.* **2019**, *58*, 18830–18834.

(6) Wang, P.; Amato, N. J.; Wang, Y. Cytotoxic and Mutagenic Properties of C3'-Epimeric Lesions of 2'-Deoxyribonucleosides in Escherichia Coli Cells. *Biochemistry* **2017**, *56*, 3725–3732.

(7) Ito, Y.; Mizuno, K.; Sumise, S.; Kimura, A.; Noguchi, N.; Fuchi, Y.; Hari, Y. Generation of 4'-Carbon Radicals via 1,5-Hydrogen Atom Transfer for the Synthesis of Bridged Nucleosides. *Org. Lett.* **2022**, *24*, 7696–7700.

(8) Schmidtgall, B.; Spork, A. P.; Wachowius, F.; Höbartner, C.; Ducho, C. Synthesis and Properties of DNA Oligonucleotides with a Zwitterionic Backbone Structure. *Chem. Commun.* **2014**, *50*, 13742–13745.

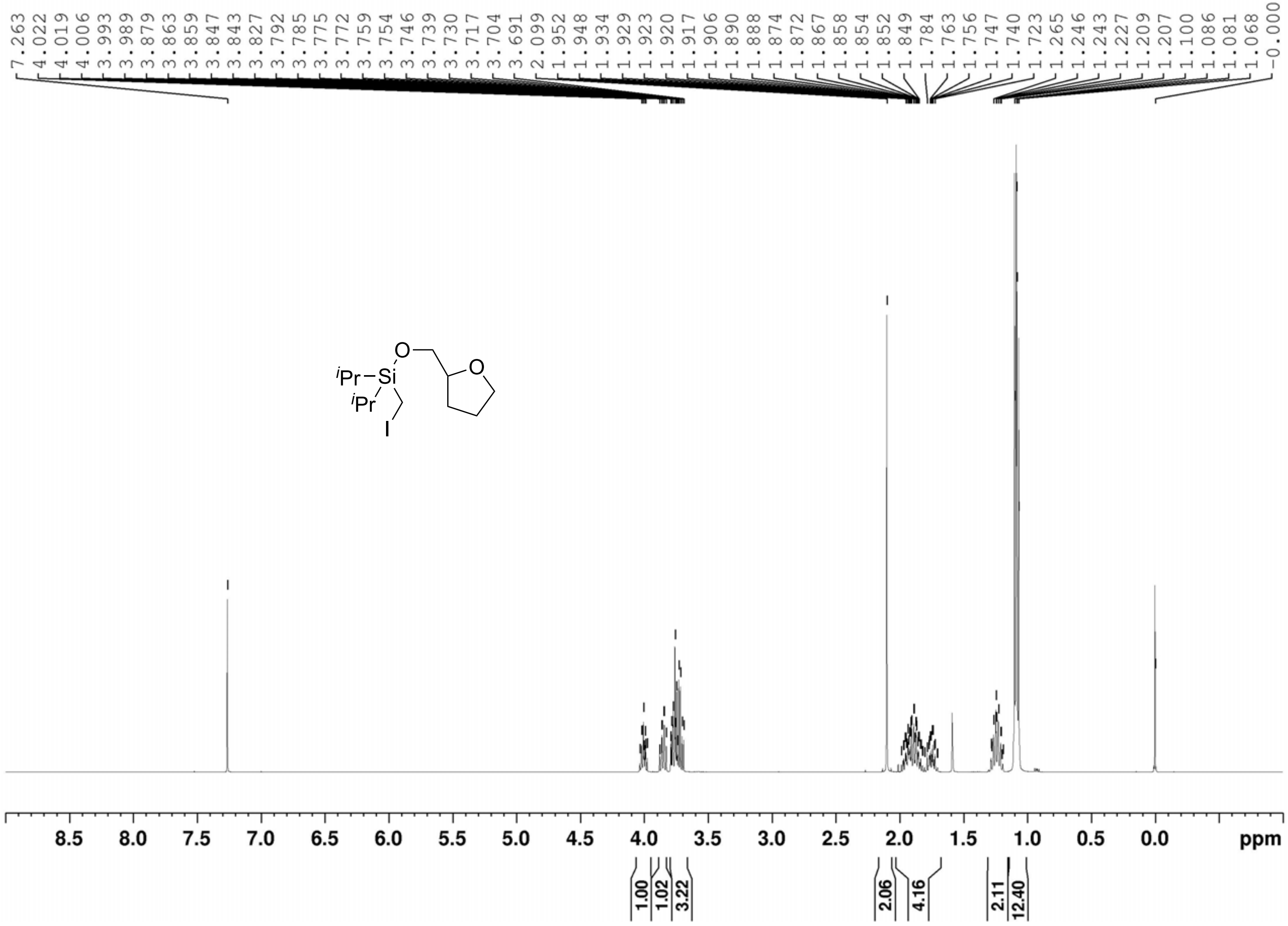

^{1}H NMR (400 MHz, $CDCl_3$) spectrum of **1a**

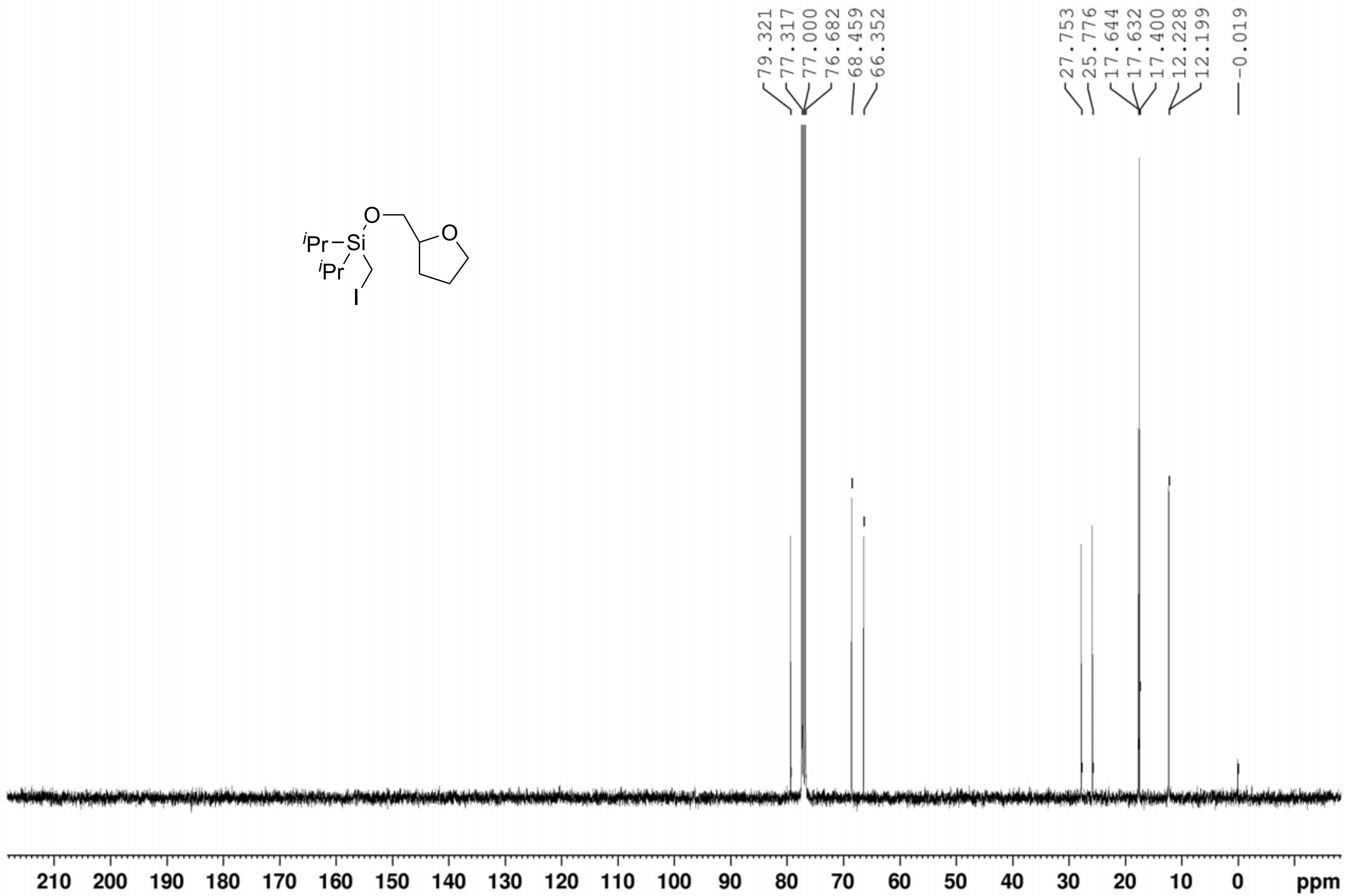

^{13}C NMR (100.6 MHz, $CDCl_3$) spectrum of **1a**

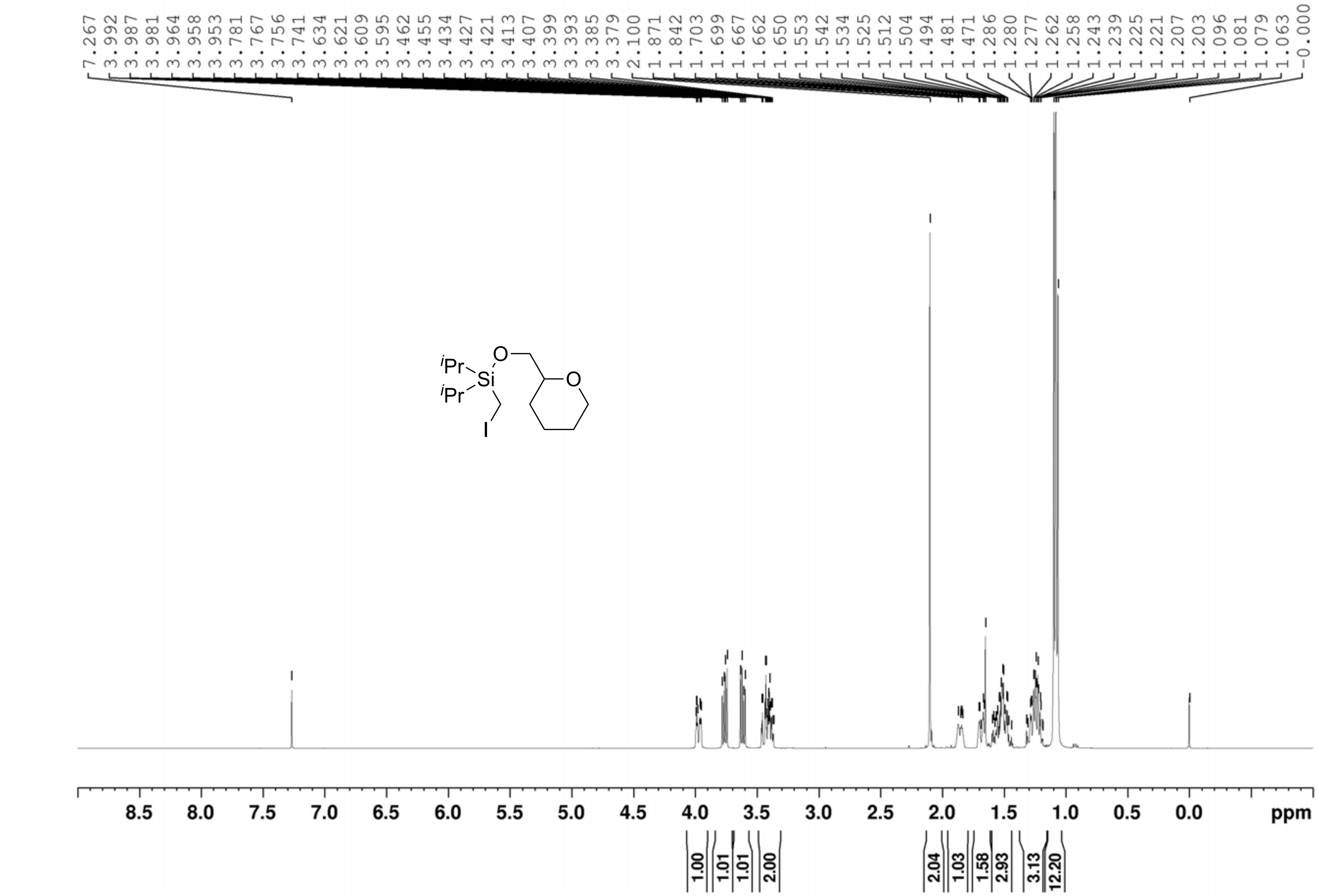

^{1}H NMR (400 MHz, $CDCl_3$) spectrum of **1b**

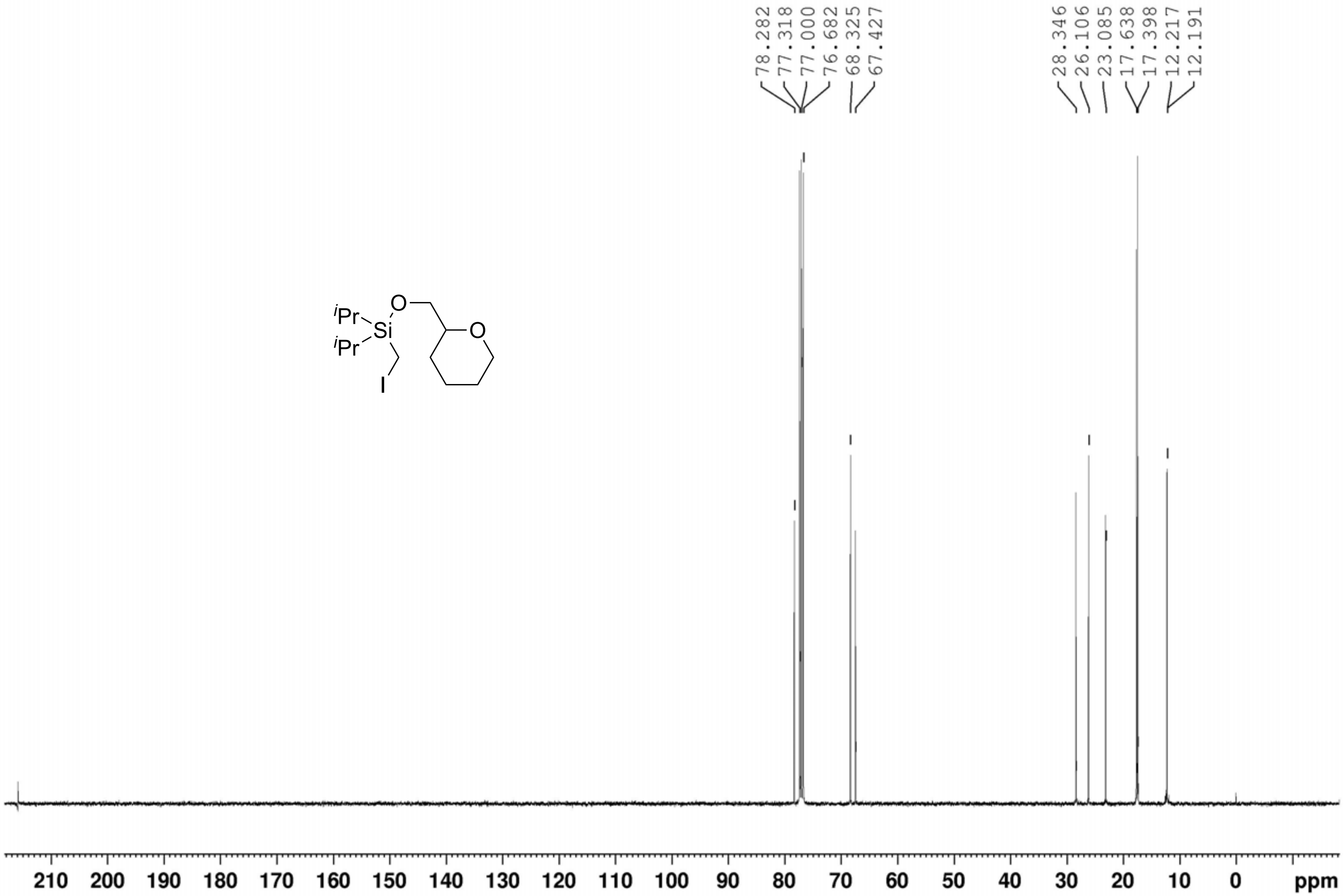

^{13}C NMR (100.6 MHz, CDCl$_3$) spectrum of **1b**

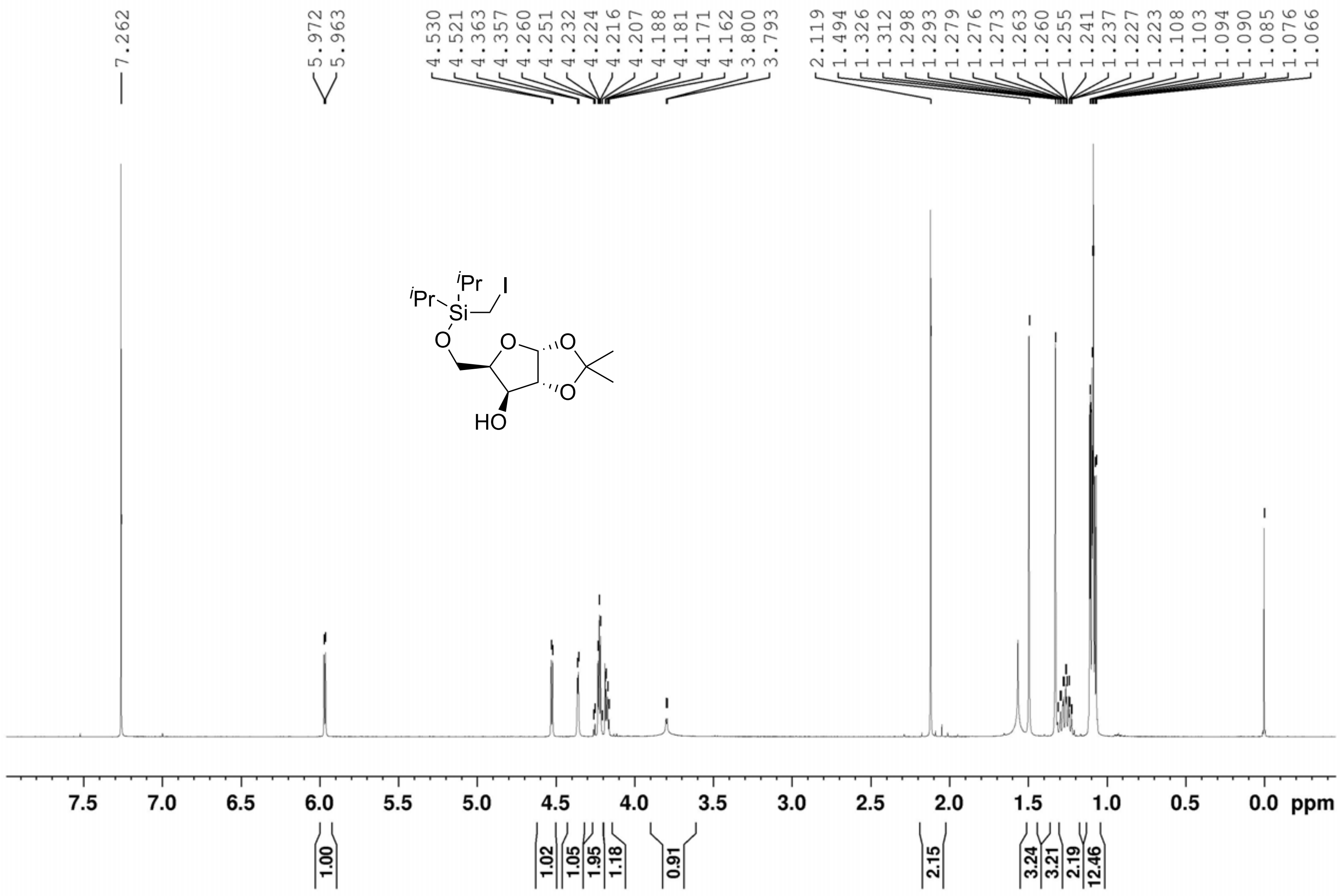

^{1}H NMR (400 MHz, $CDCl_3$) spectrum of **S1-2**

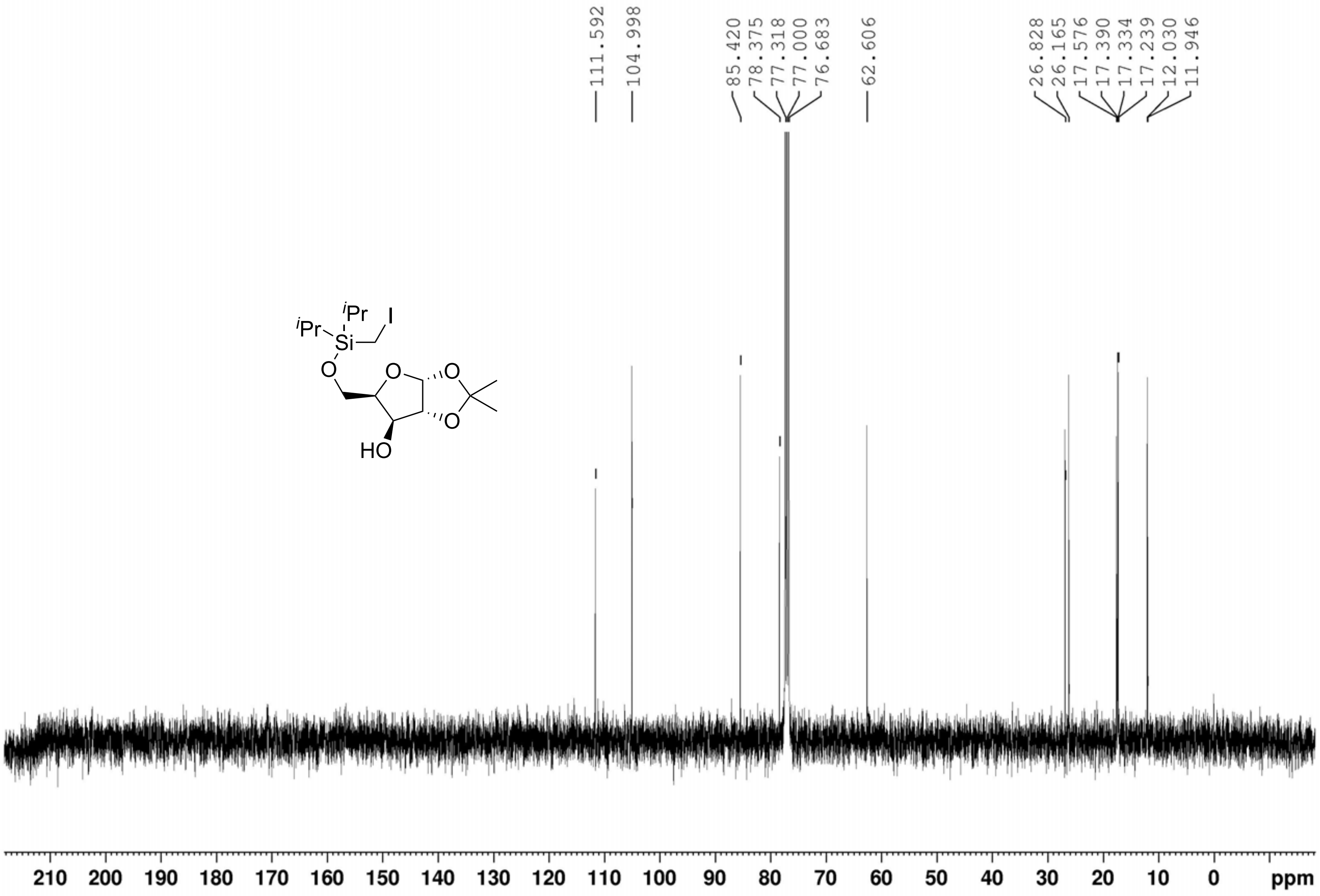

^{13}C NMR (100.6 MHz, $CDCl_3$) spectrum of **S1-2**

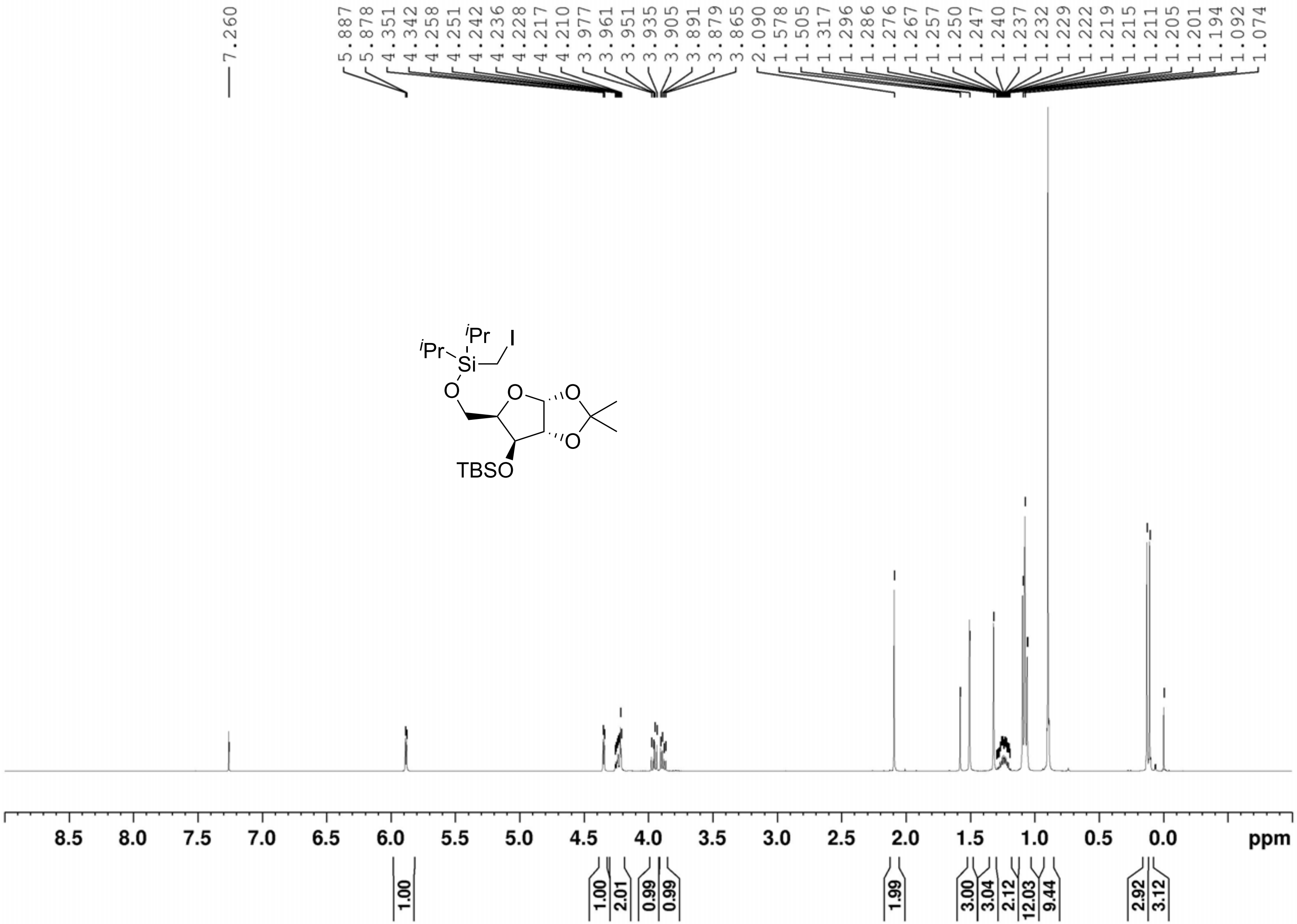

^{1}H NMR (400 MHz, $CDCl_3$) spectrum of **1c**

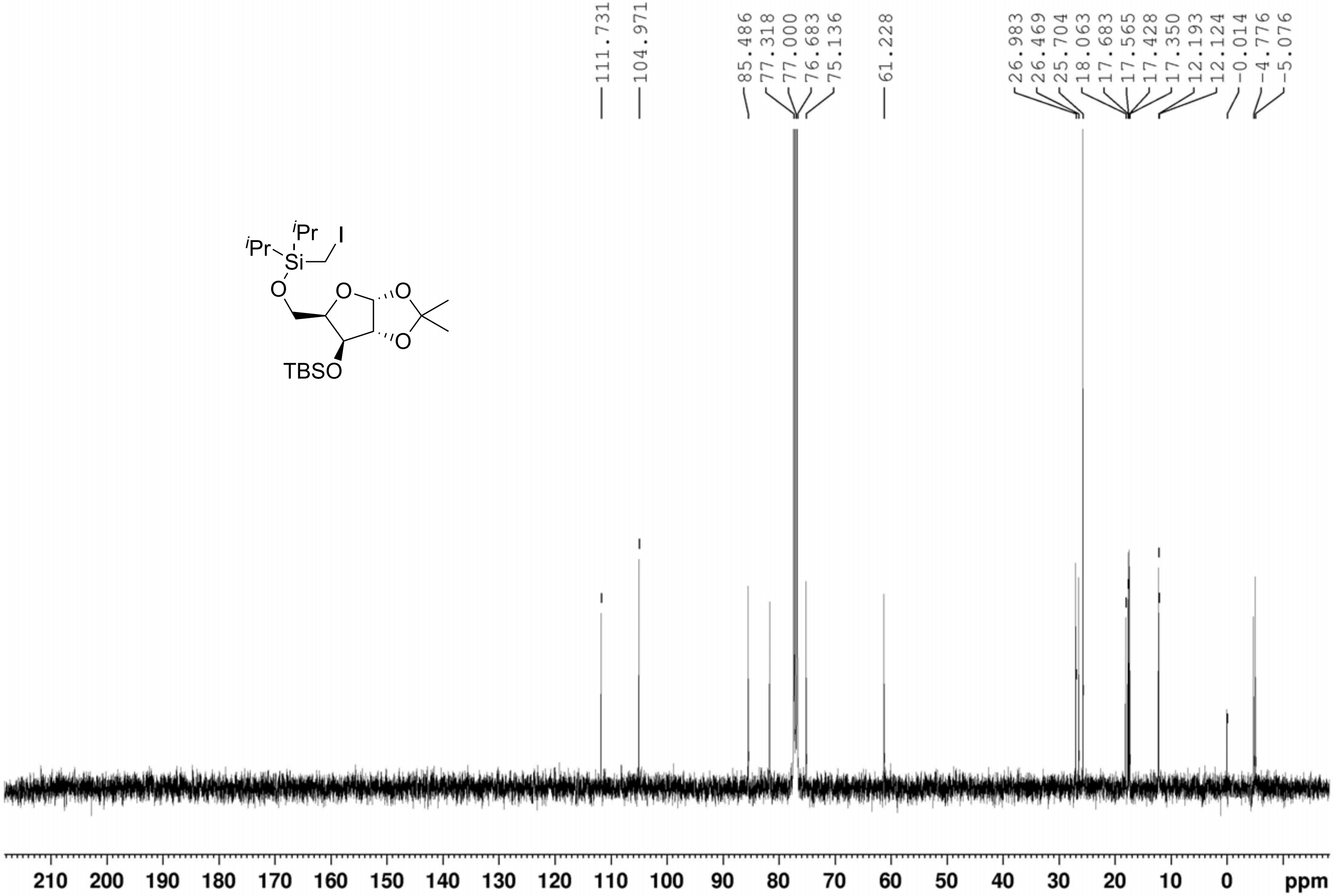

^{13}C NMR (100.6 MHz, $CDCl_3$) spectrum of **1c**

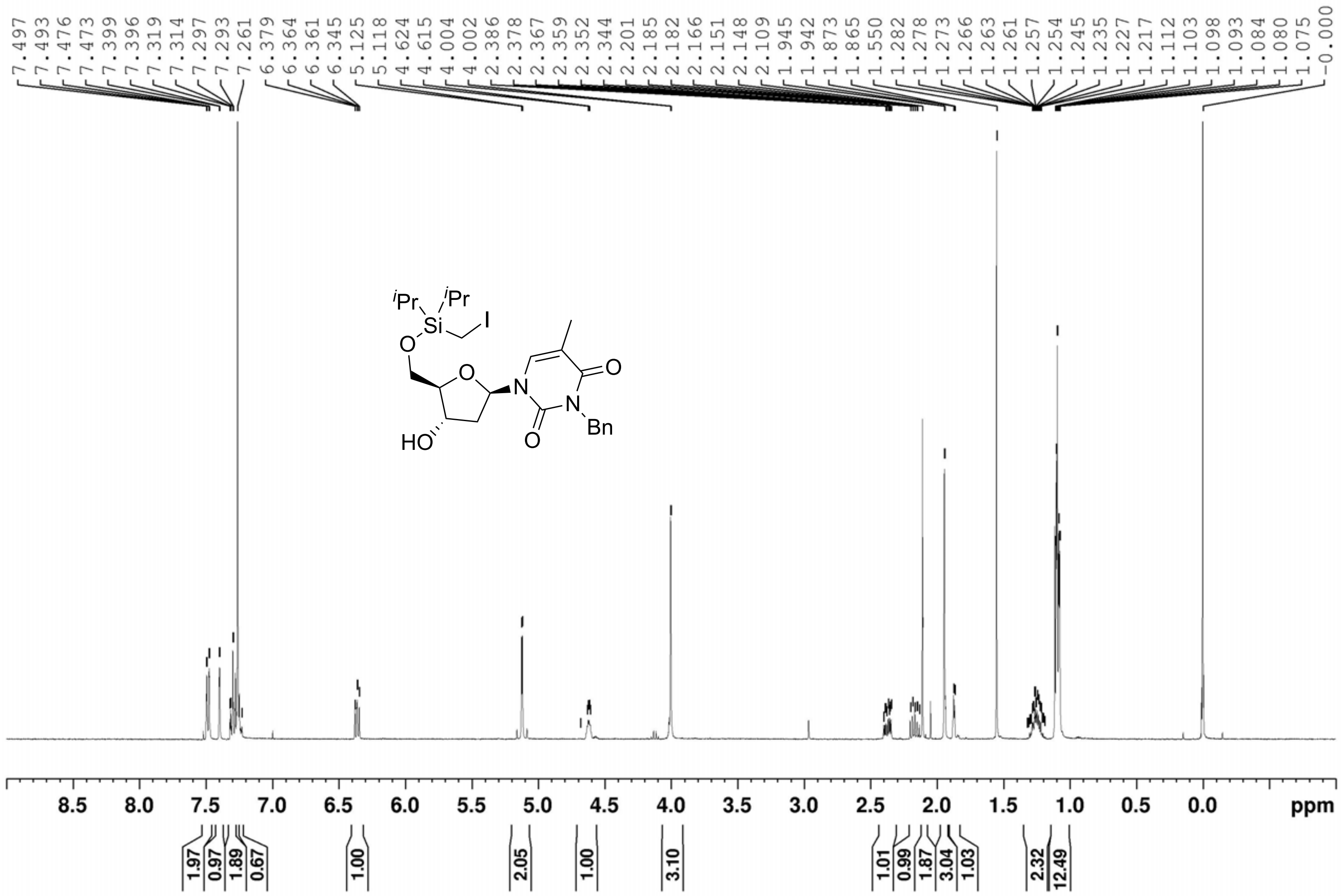

^{1}H NMR (400 MHz, $CDCl_3$) spectrum of **S2-2**

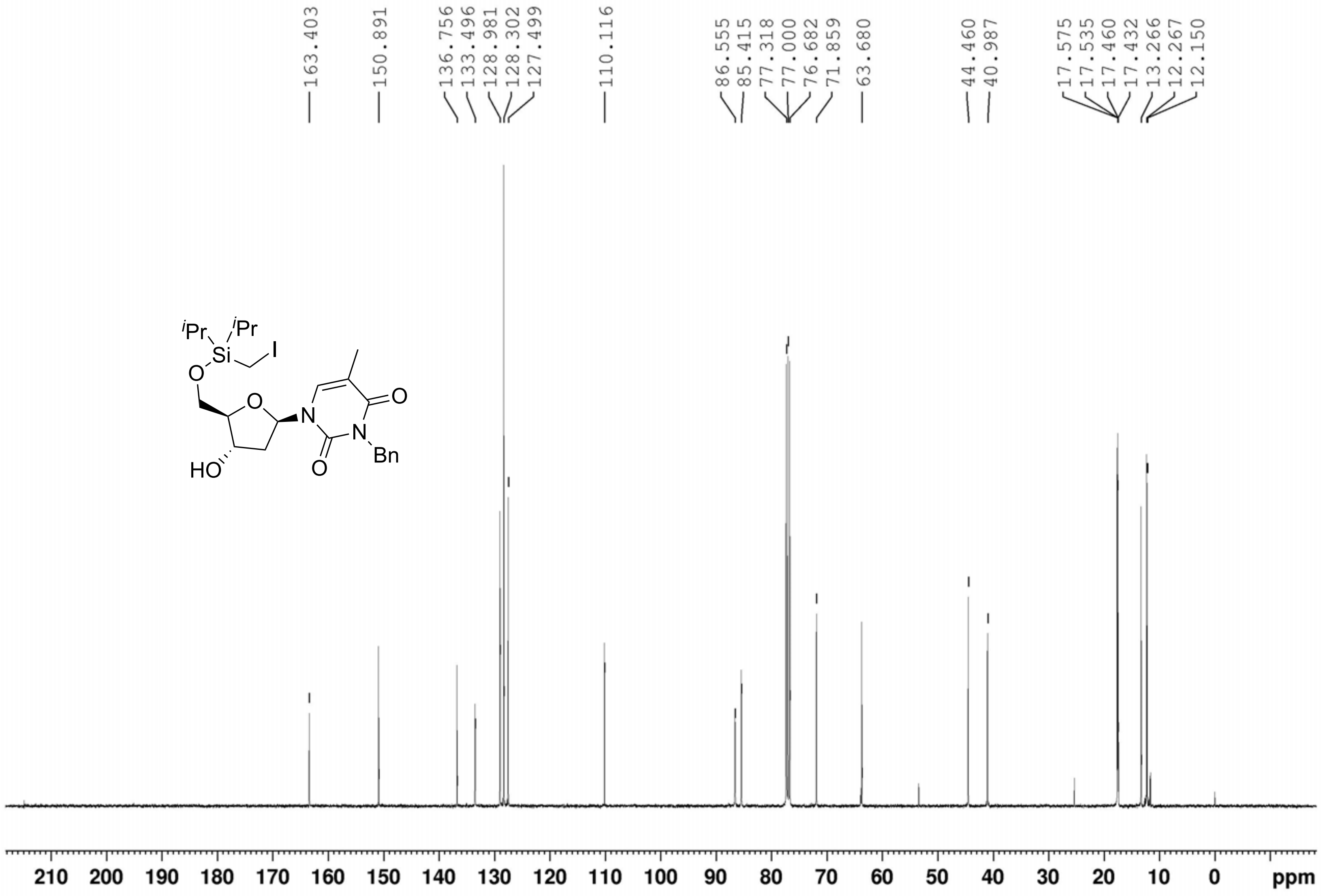

^{13}C NMR (100.6 MHz, $CDCl_3$) spectrum of **S2-2**

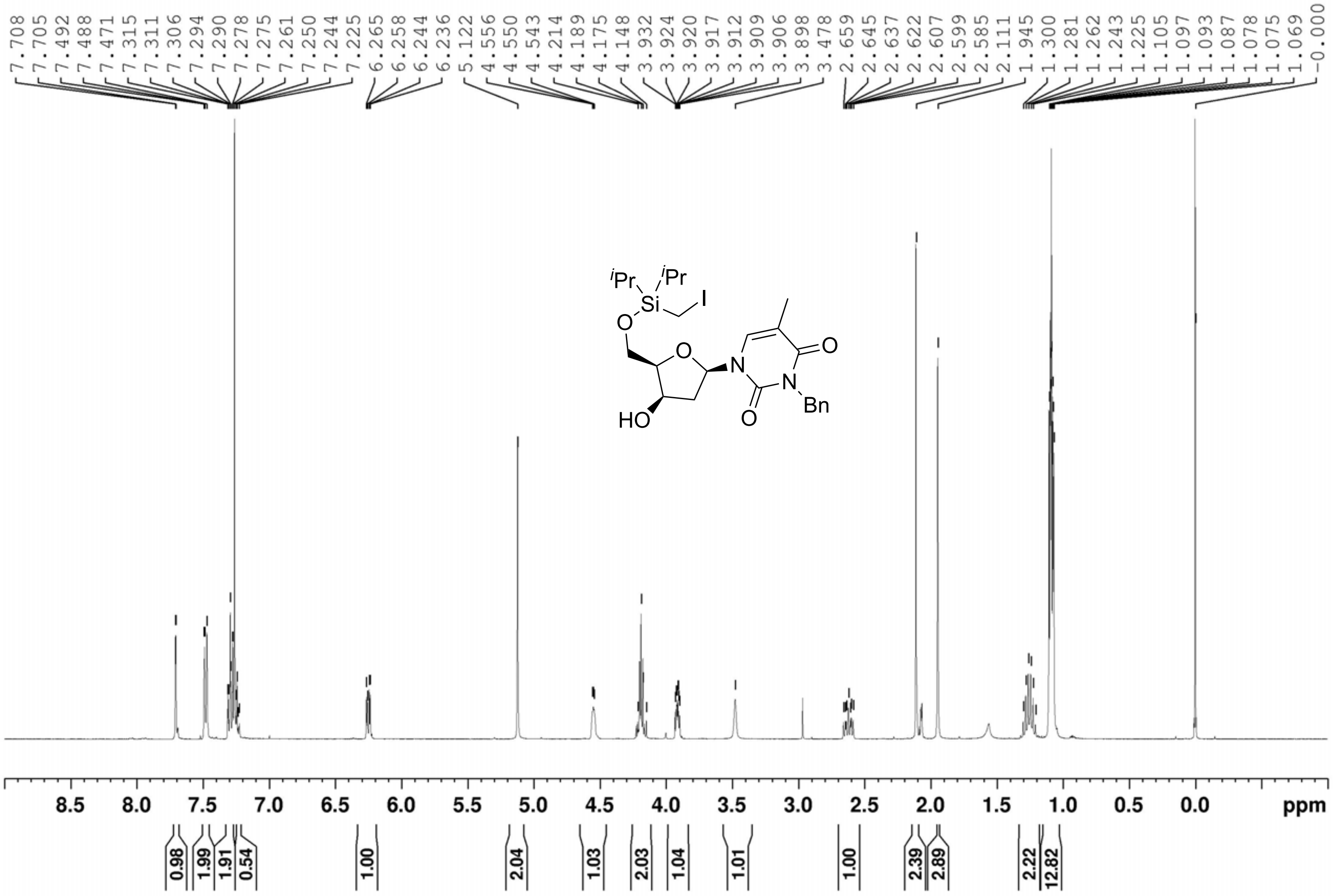

^{1}H NMR (400 MHz, $CDCl_3$) spectrum of **S2-3**

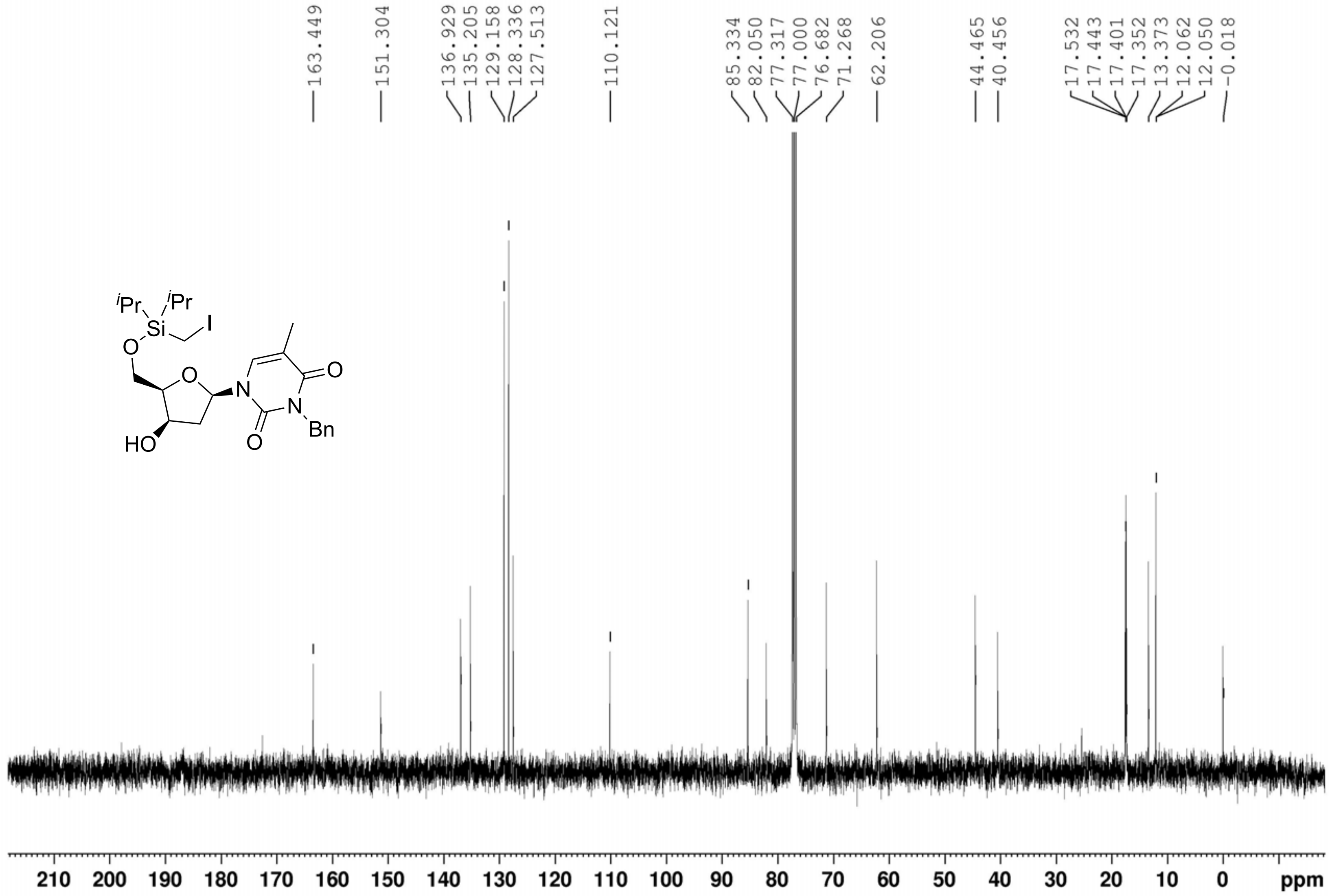

^{13}C NMR (100.6 MHz, $CDCl_3$) spectrum of **S2-3**

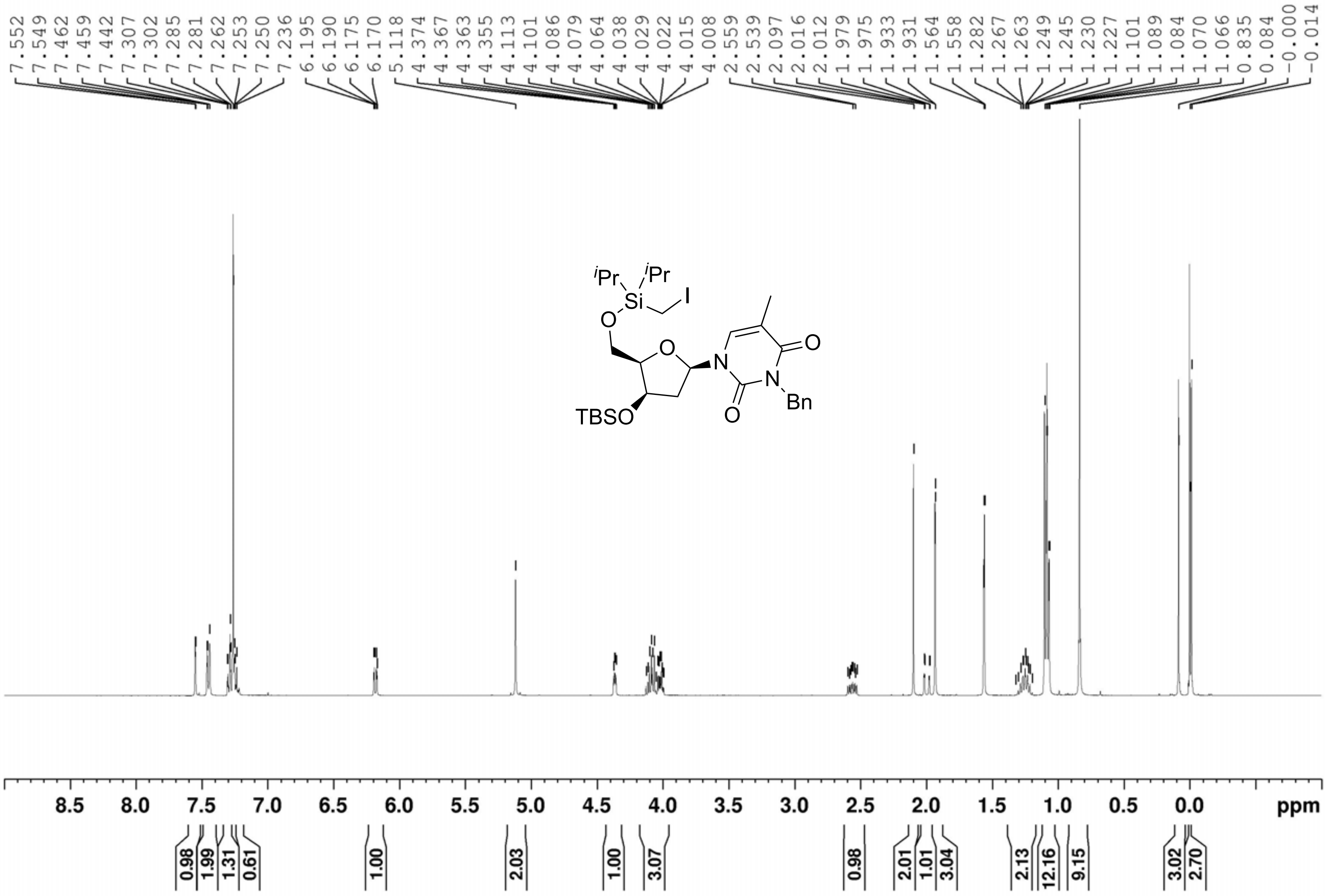

^{1}H NMR (400 MHz, $CDCl_3$) spectrum of **1e**

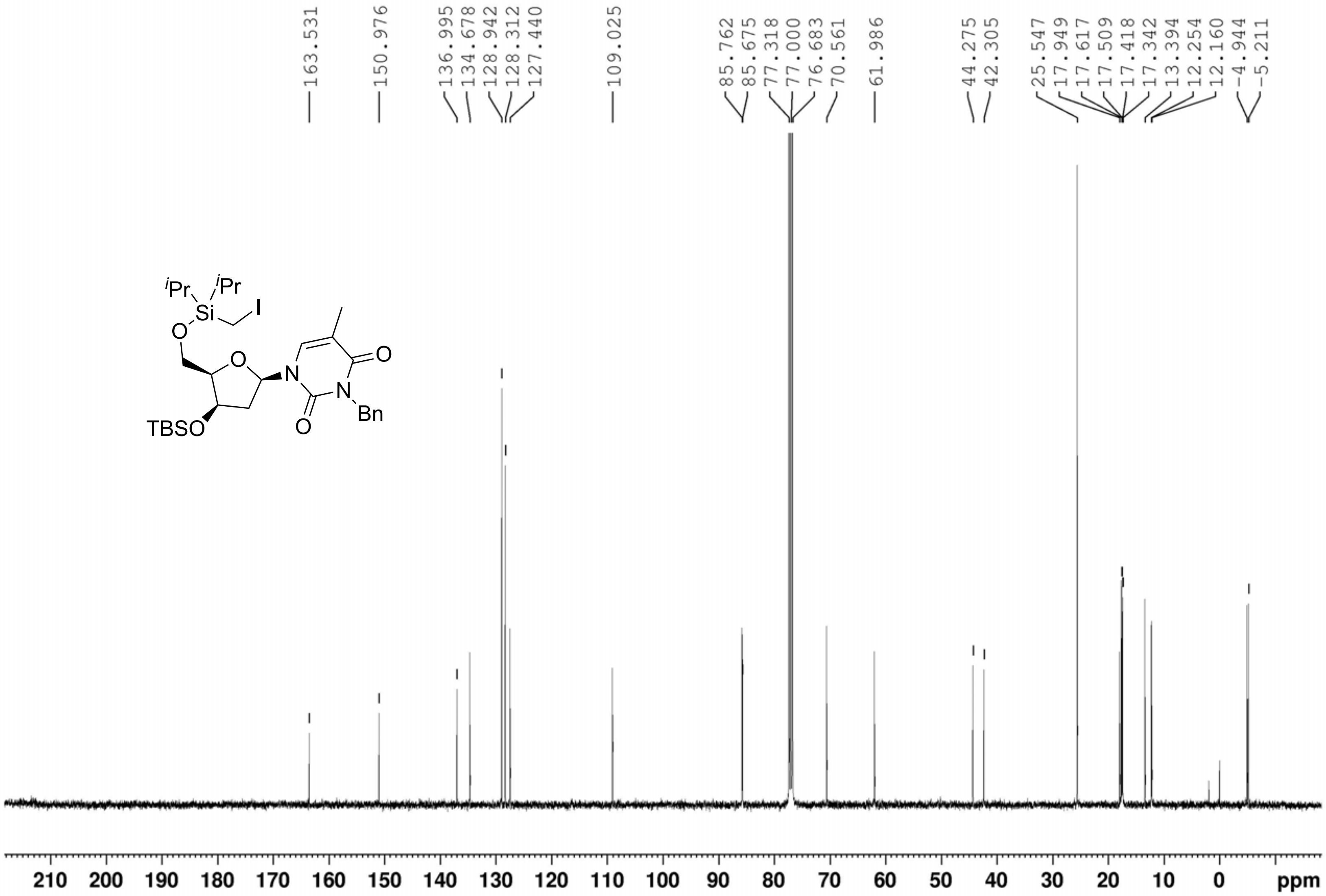

^{13}C NMR (100.6 MHz, $CDCl_3$) spectrum of **1e**

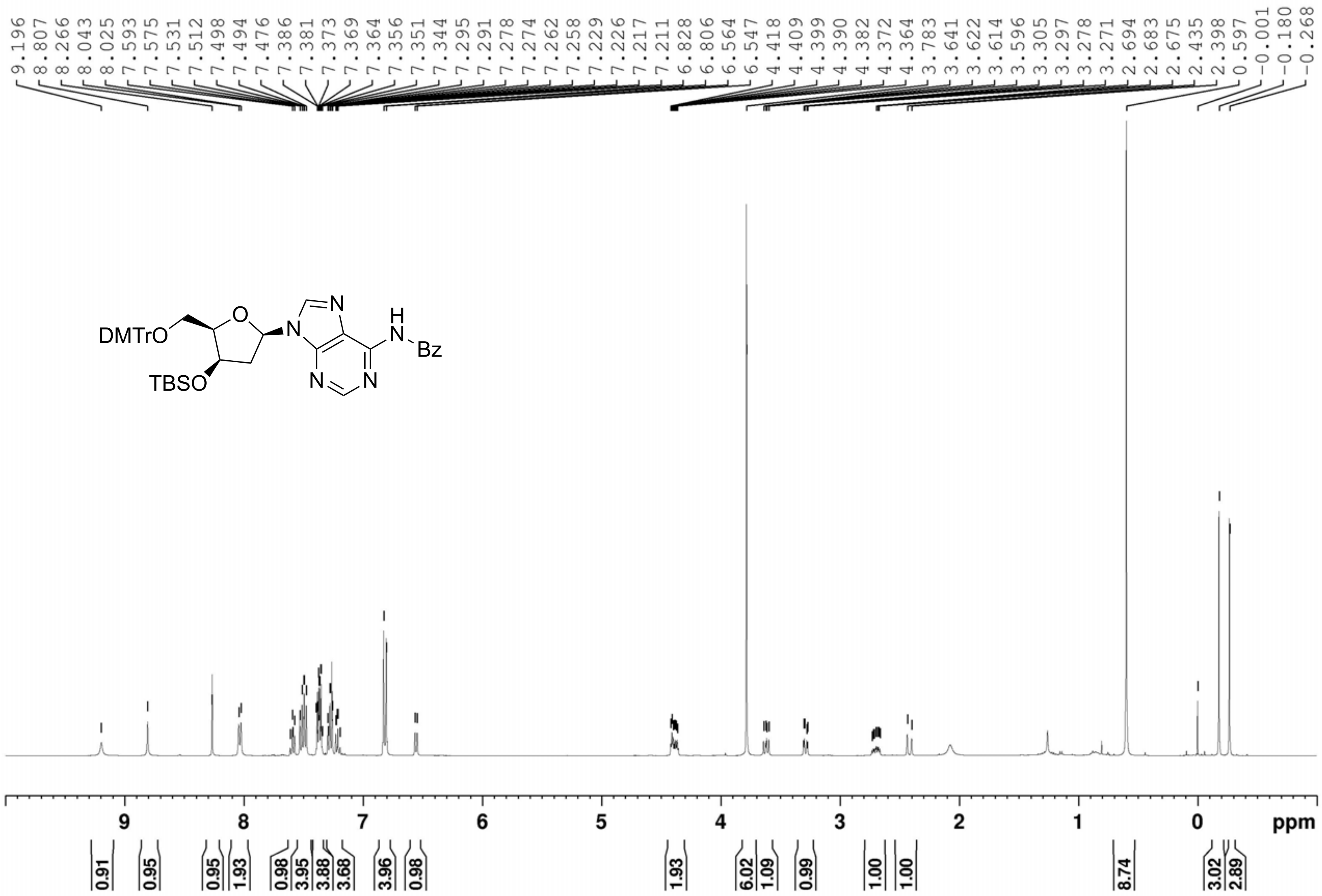

^{1}H NMR (400 MHz, $CDCl_3$) spectrum of **S3-2**

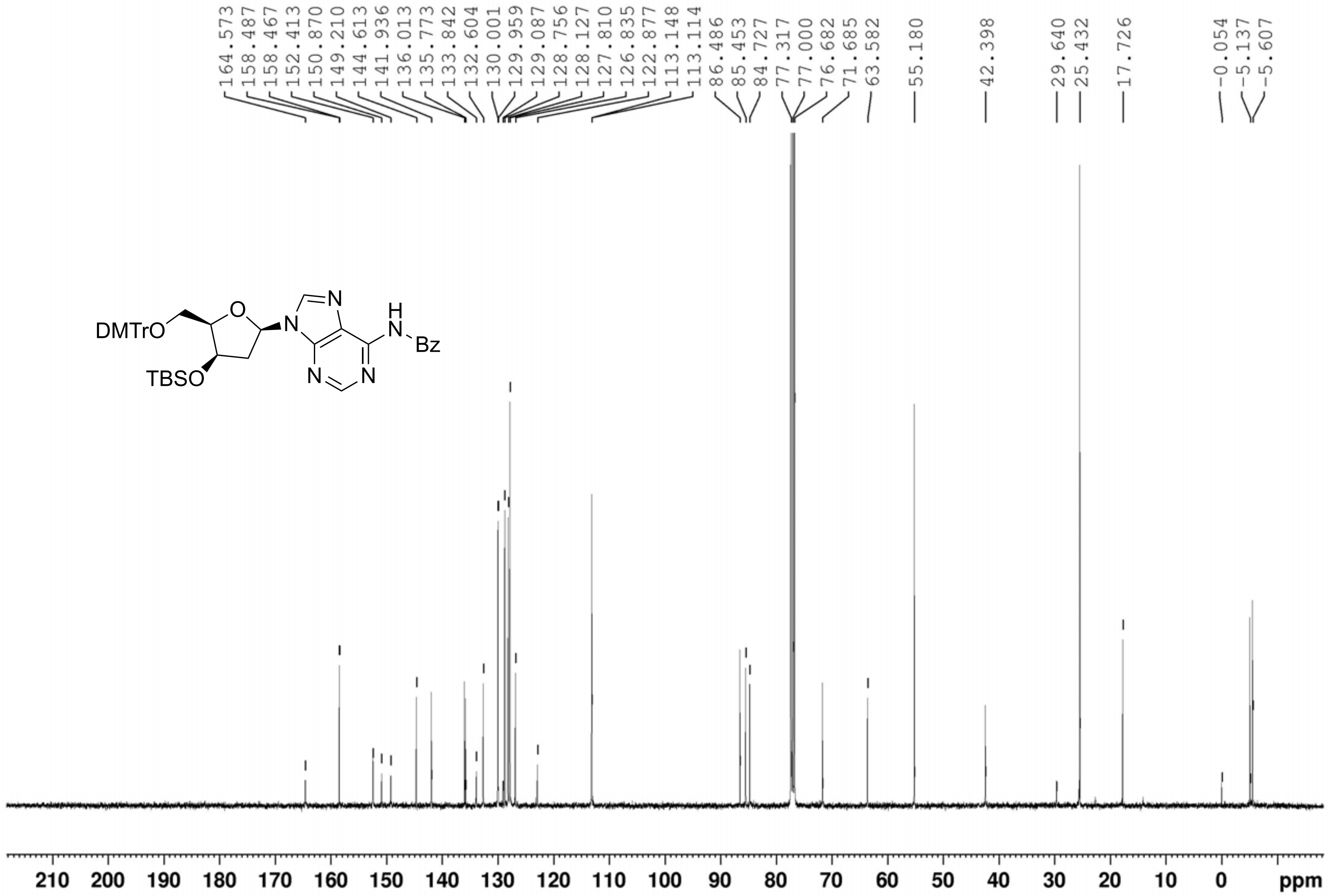

^{13}C NMR (100.6 MHz, $CDCl_3$) spectrum of **S3-2**

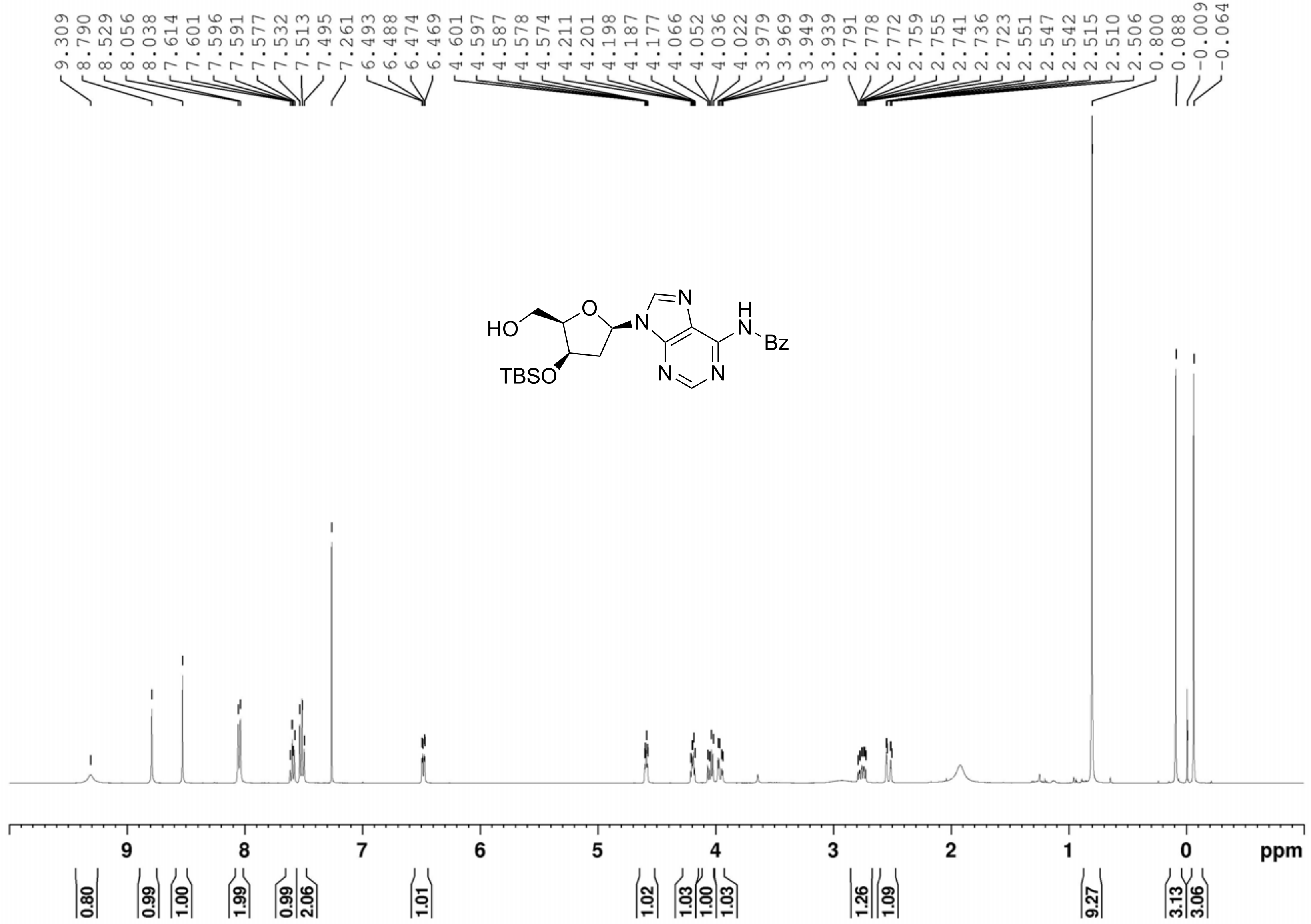

^{1}H NMR (400 MHz, $CDCl_3$) spectrum of **S3-3**

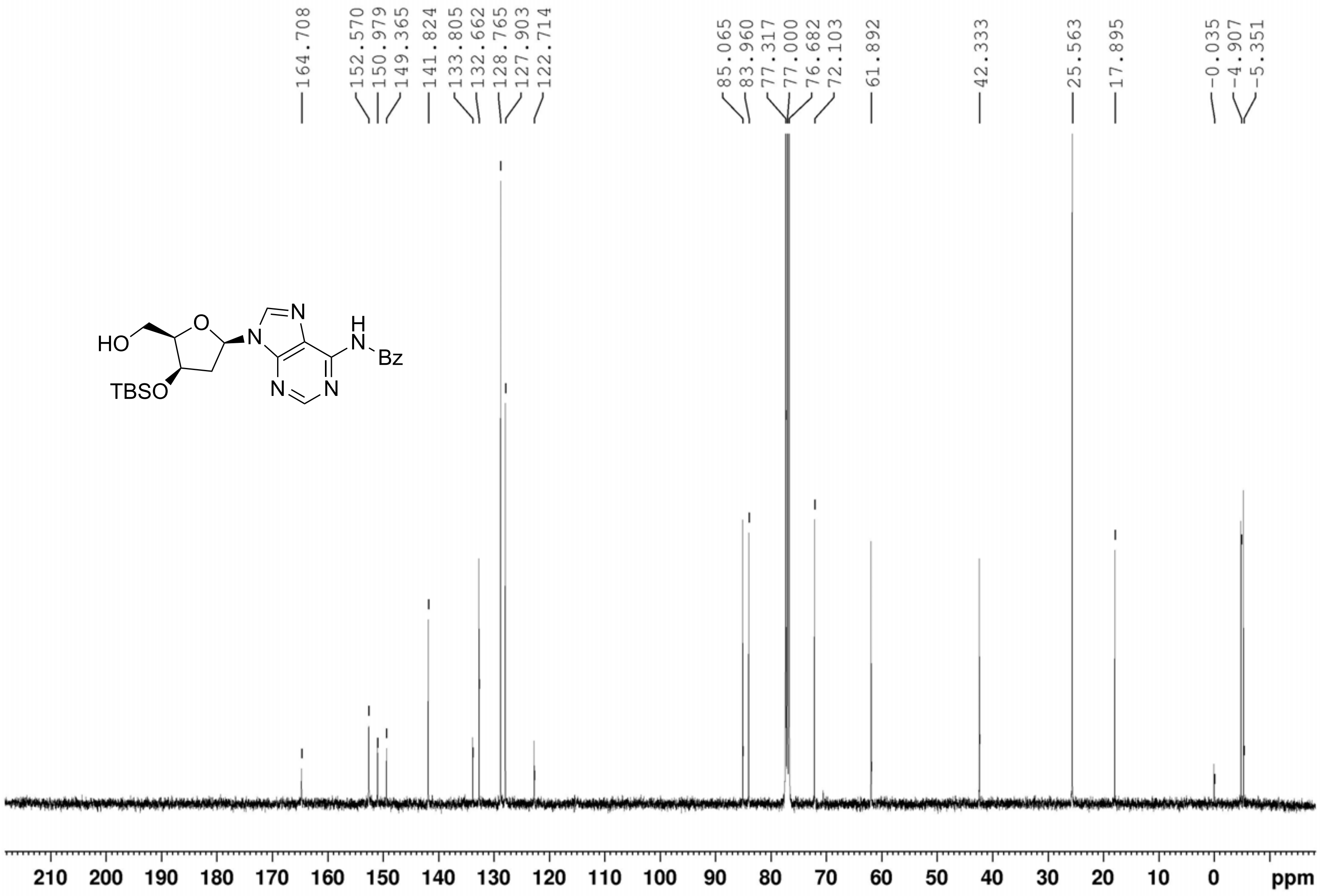

^{13}C NMR (100.6 MHz, $CDCl_3$) spectrum of **S3-3**

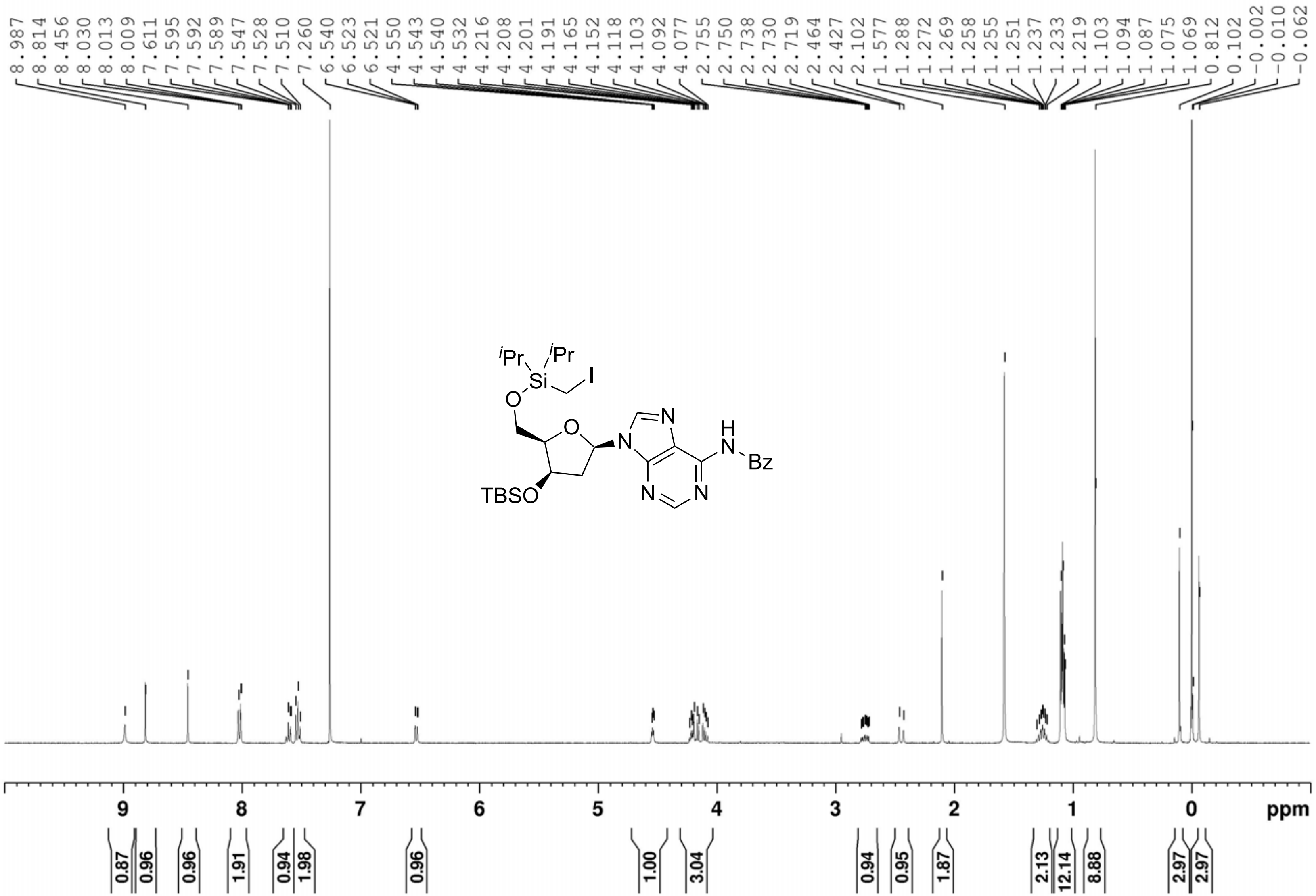

^{1}H NMR (400 MHz, $CDCl_3$) spectrum of **1f**

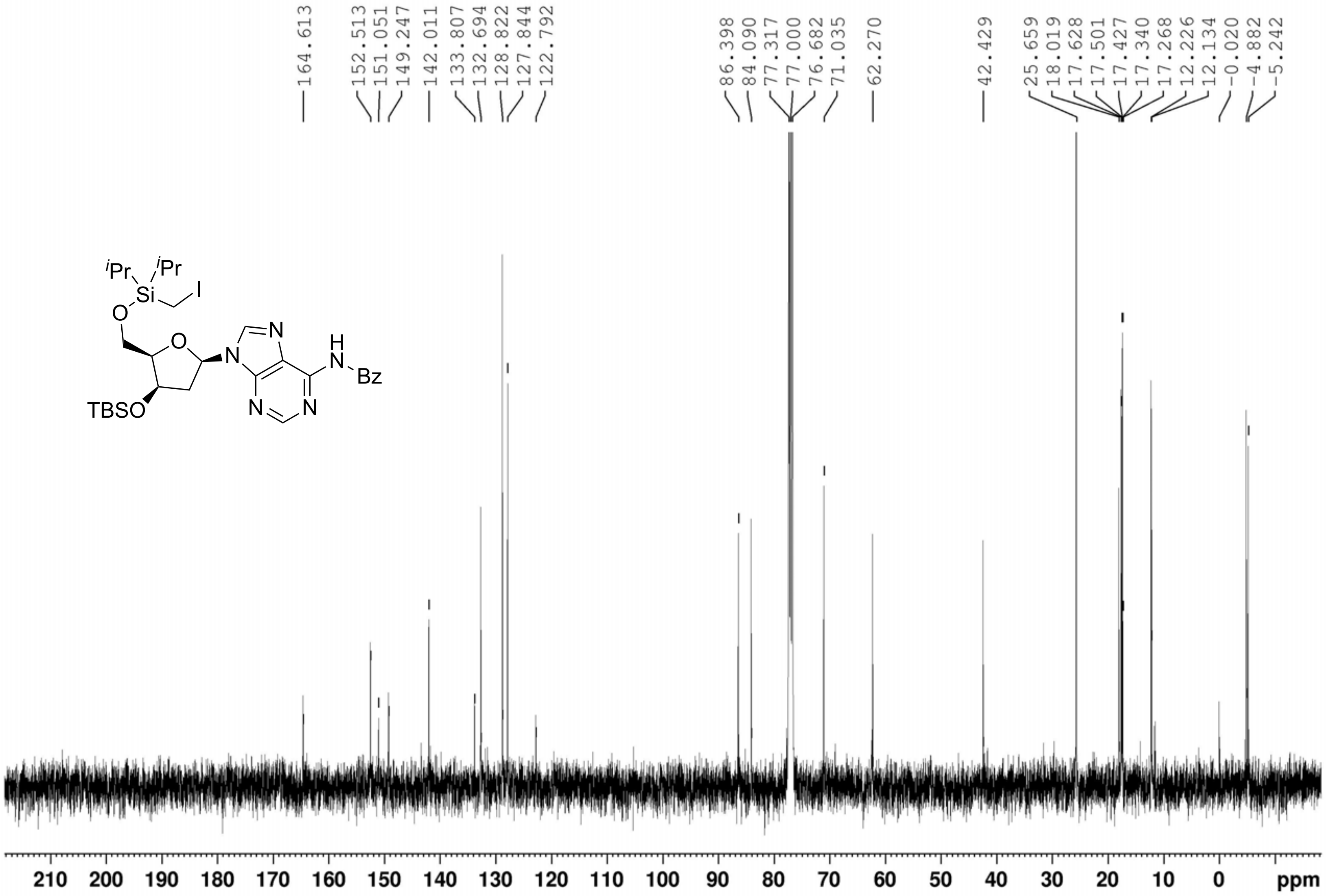

^{13}C NMR (100.6 MHz, $CDCl_3$) spectrum of **1f**

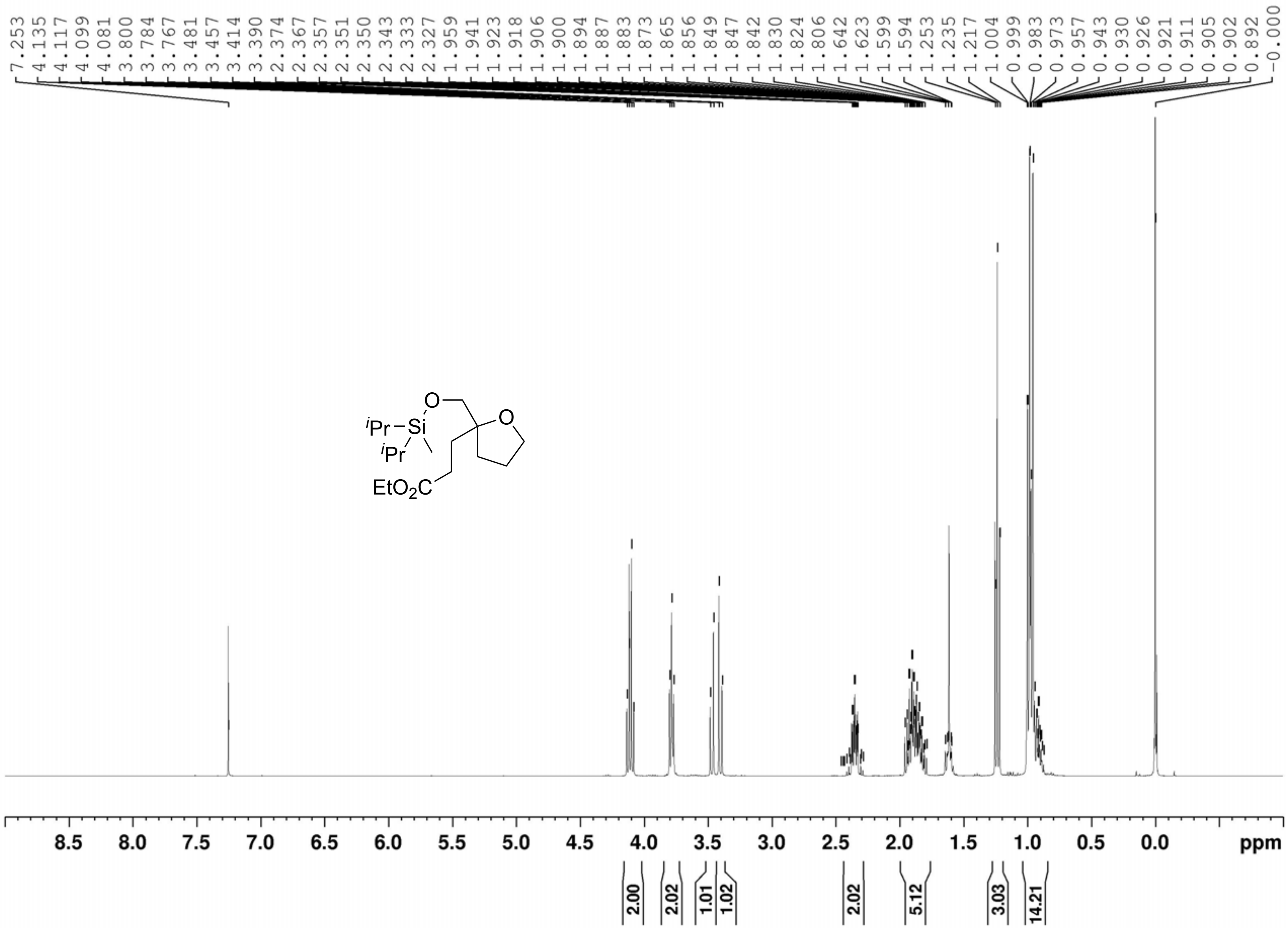

^{1}H NMR (400 MHz, $CDCl_3$) spectrum of **3aa**

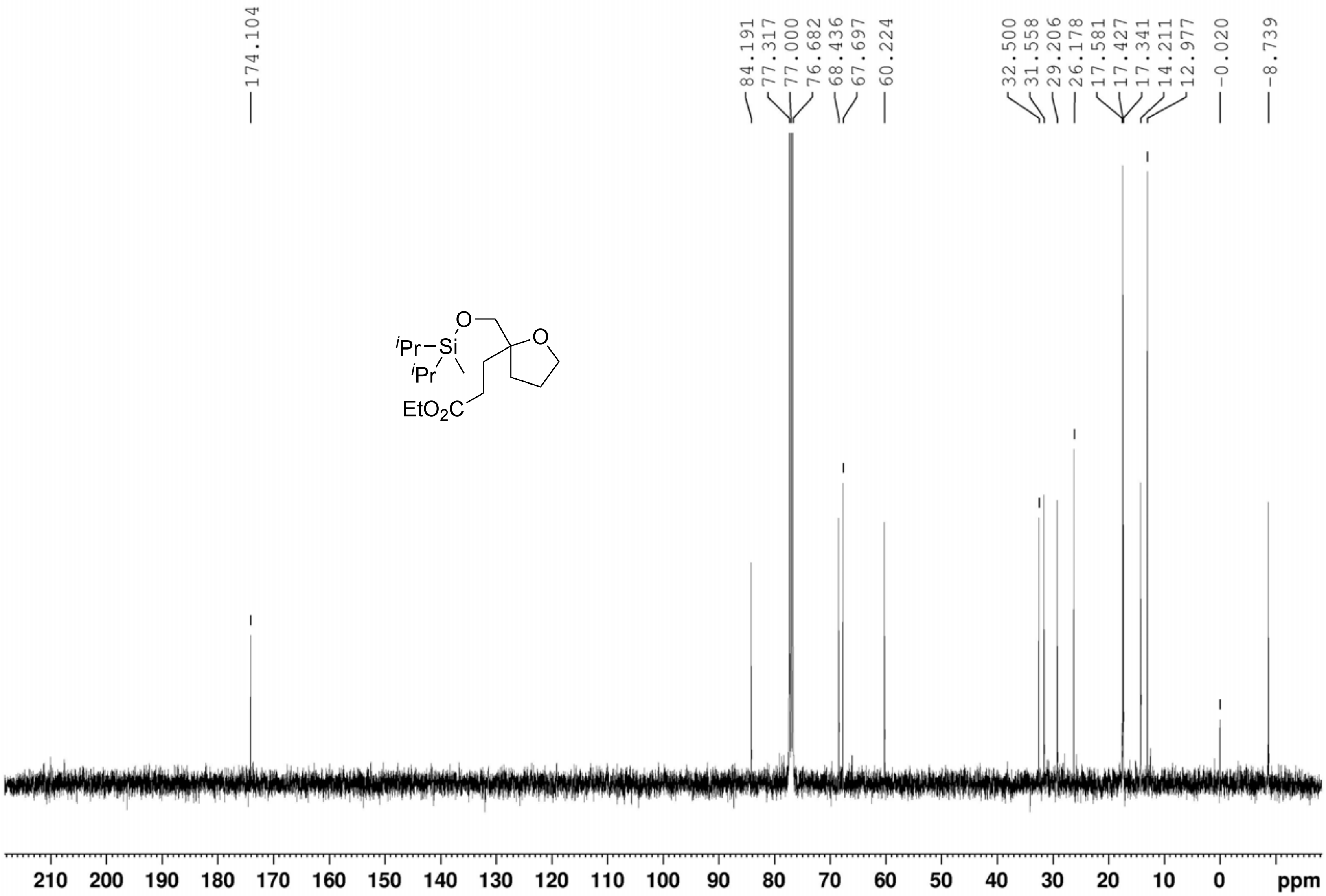

^{13}C NMR (100.6 MHz, $CDCl_3$) spectrum of **3aa**

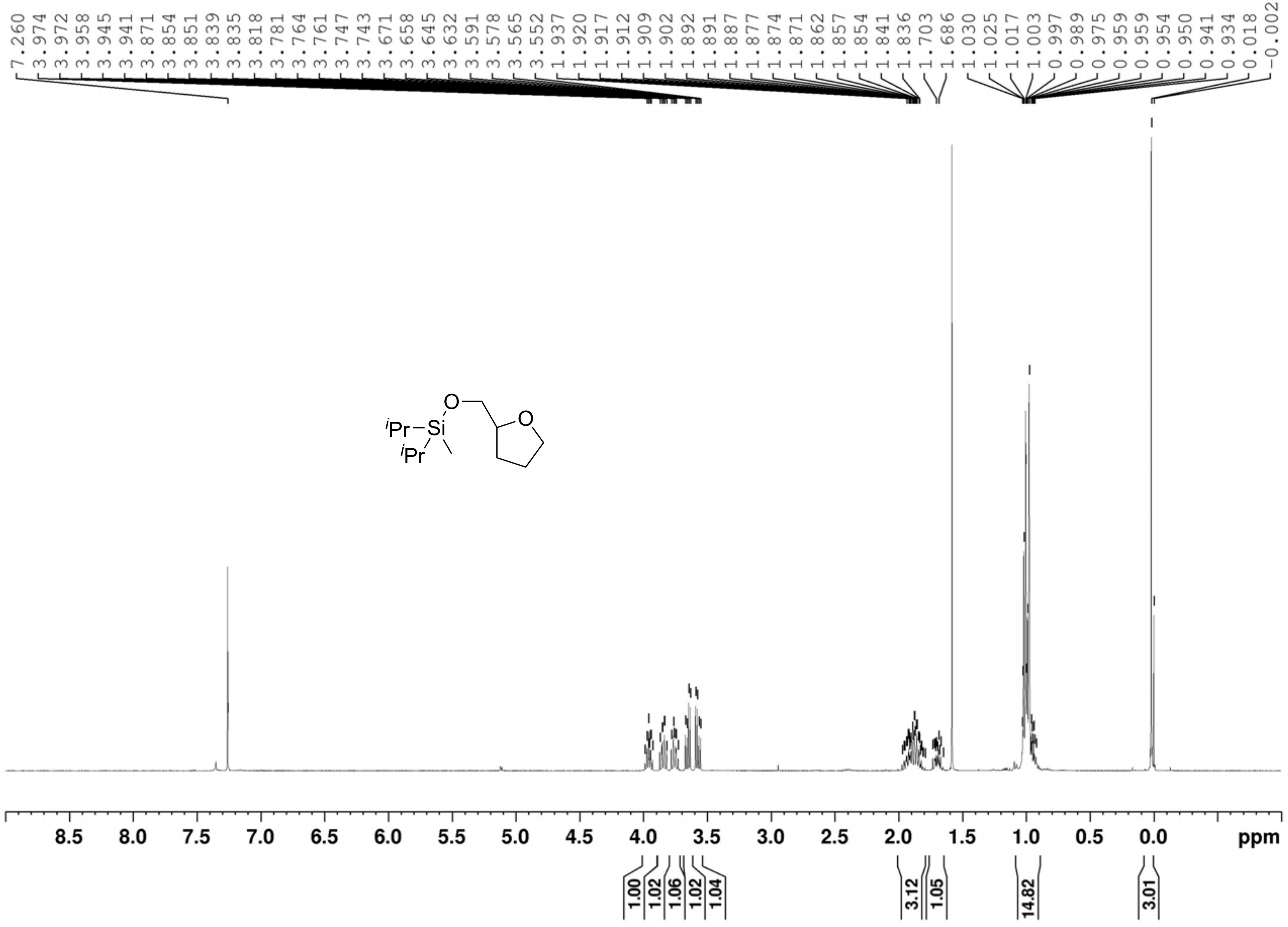

^{1}H NMR (400 MHz, $CDCl_3$) spectrum of **1a-1**

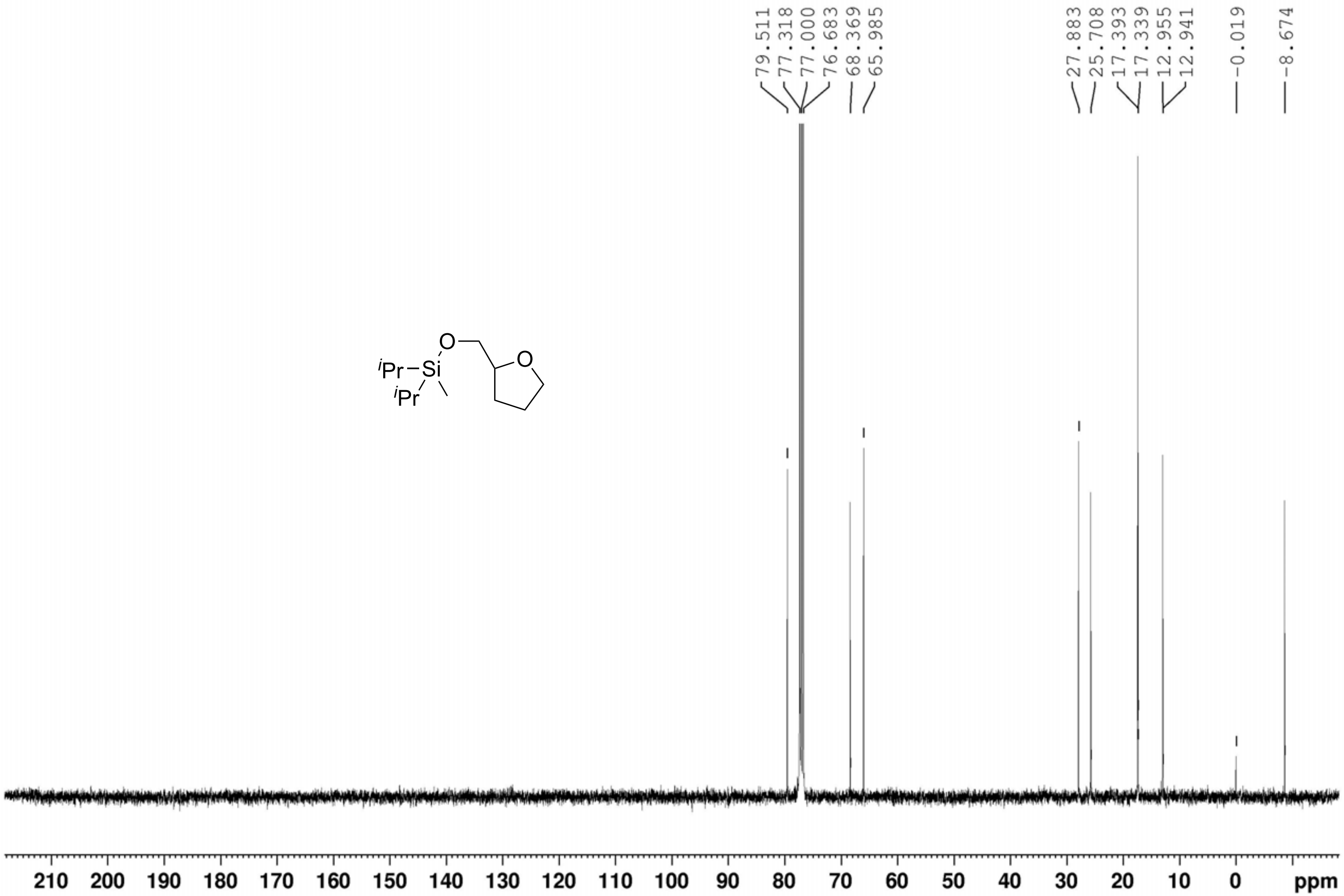

^{13}C NMR (100.6 MHz, $CDCl_3$) spectrum of **1a-1**

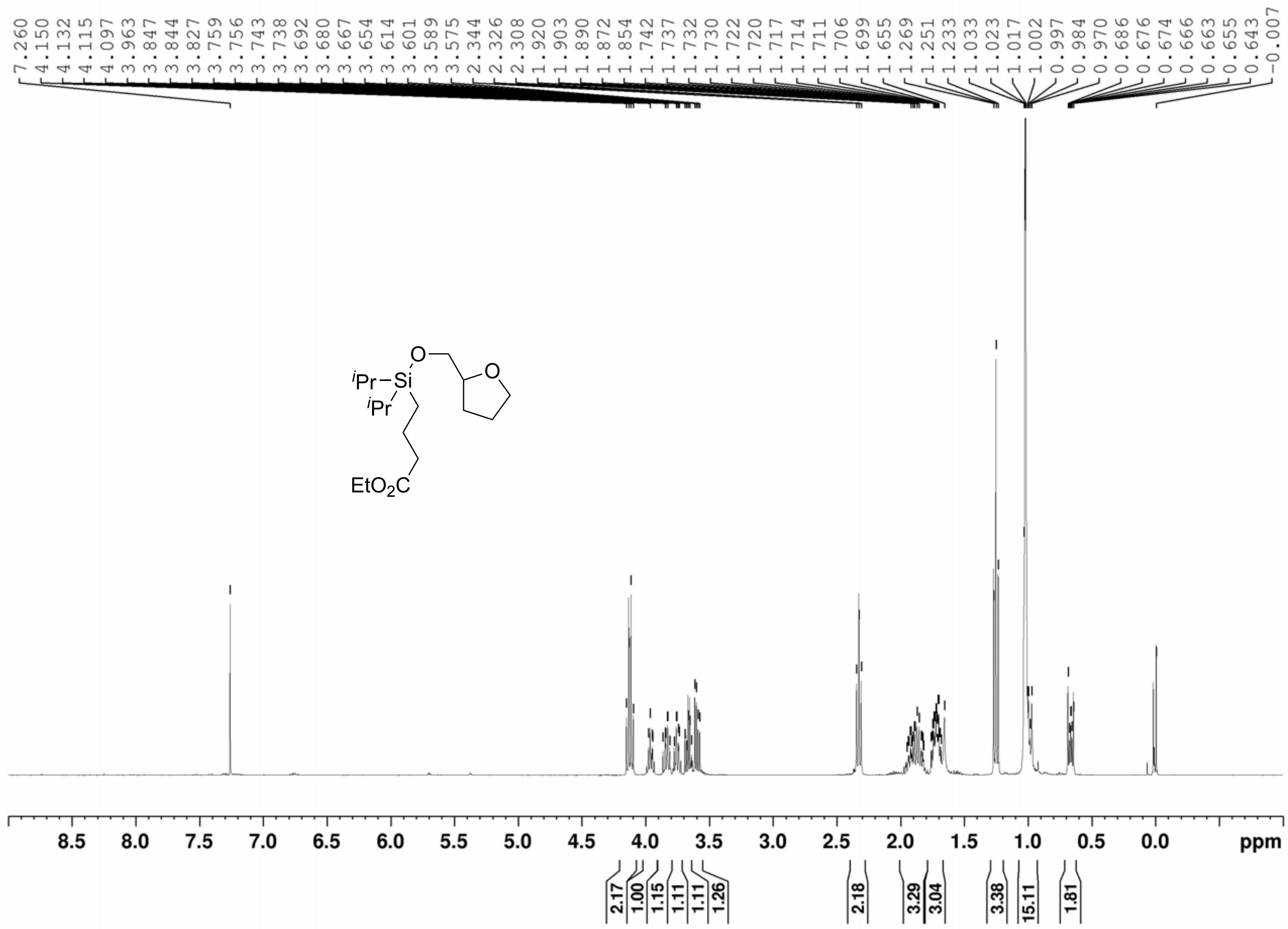

^{1}H NMR (400 MHz, $CDCl_3$) spectrum of **3aa-1**

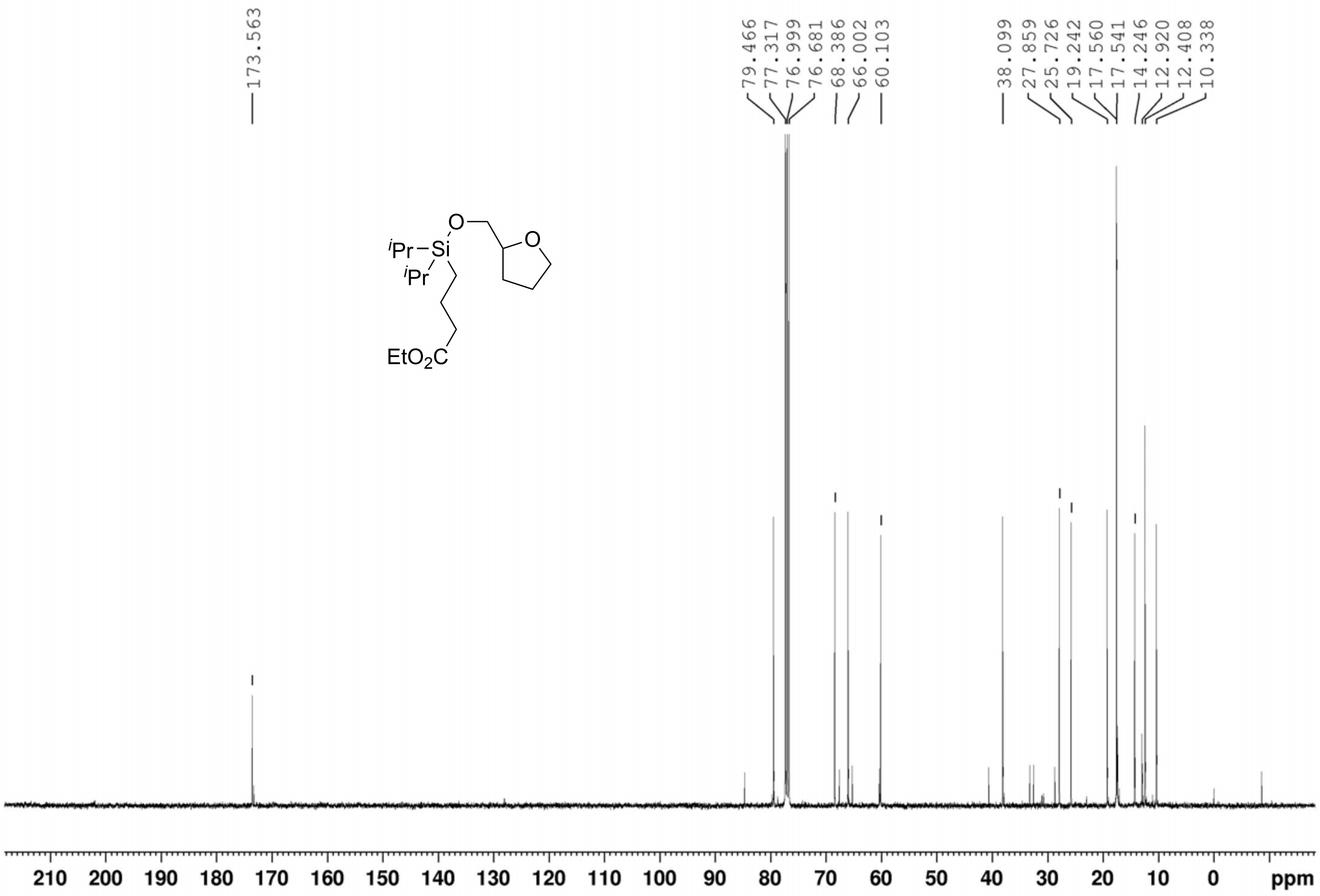

^{13}C NMR (100.6 MHz, $CDCl_3$) spectrum of **3aa-1**

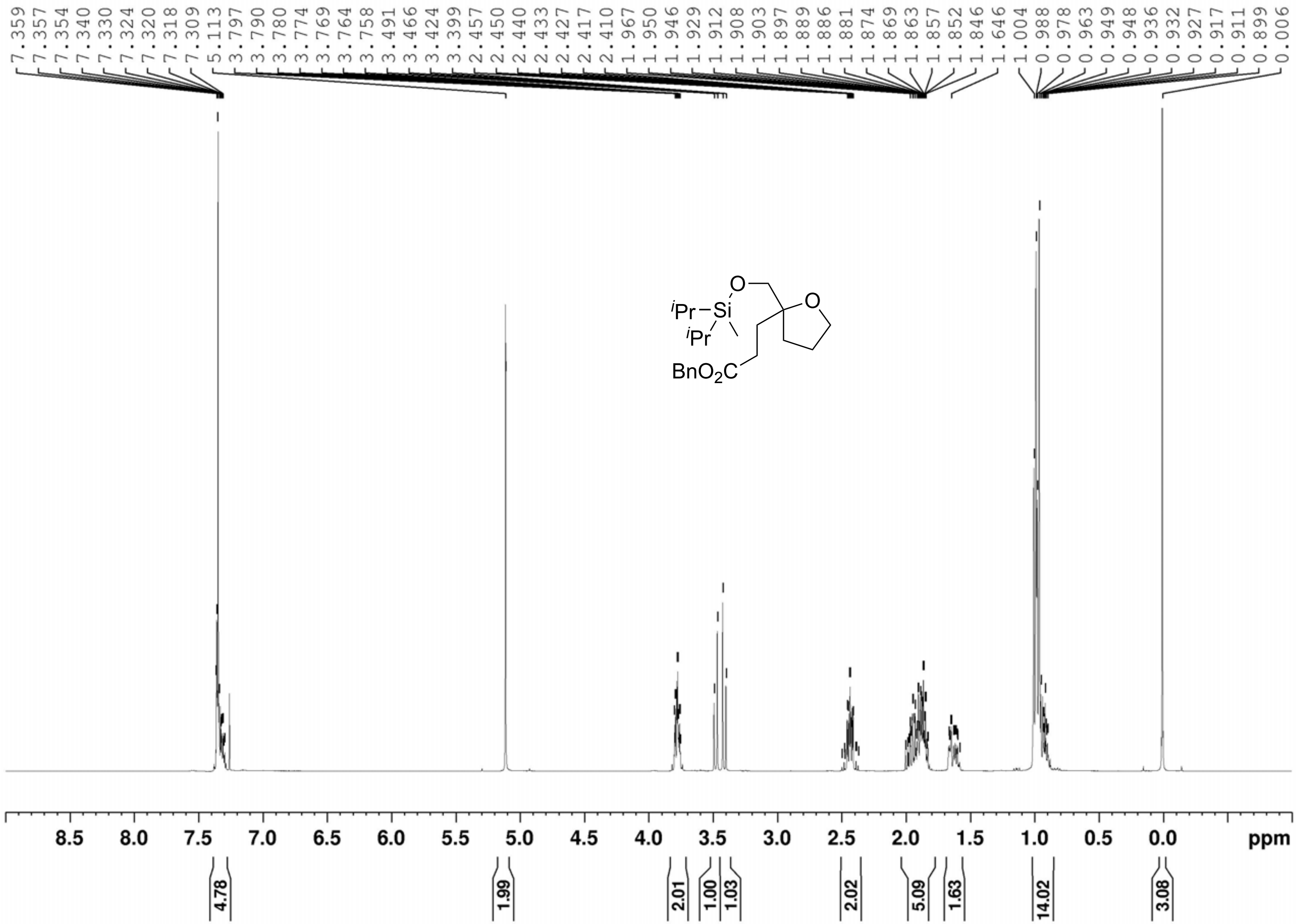

^{1}H NMR (400 MHz, $CDCl_3$) spectrum of **3ab**

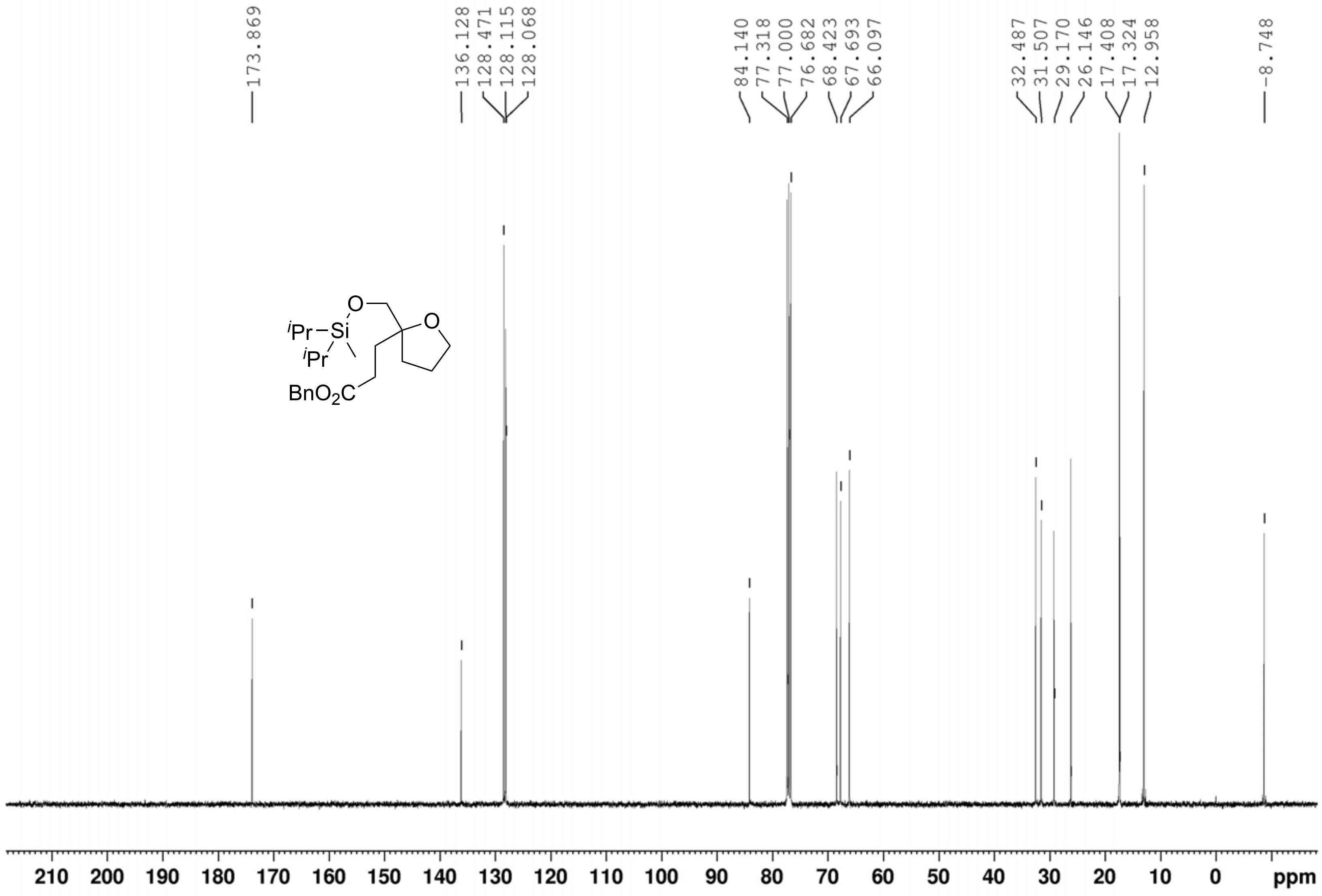

^{13}C NMR (100.6 MHz, $CDCl_3$) spectrum of **3ab**

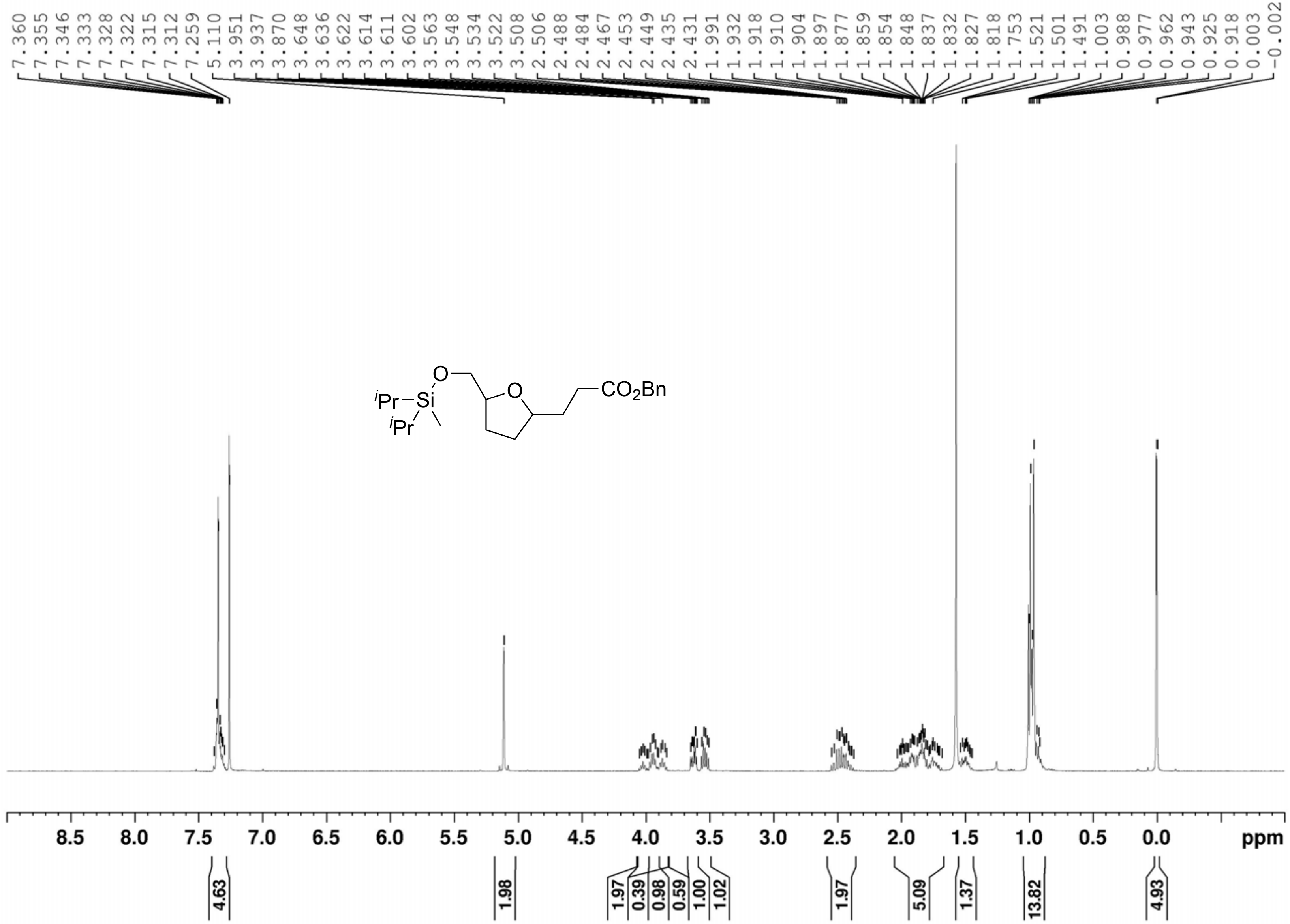

^{1}H NMR (400 MHz, $CDCl_3$) spectrum of **3ab-1**

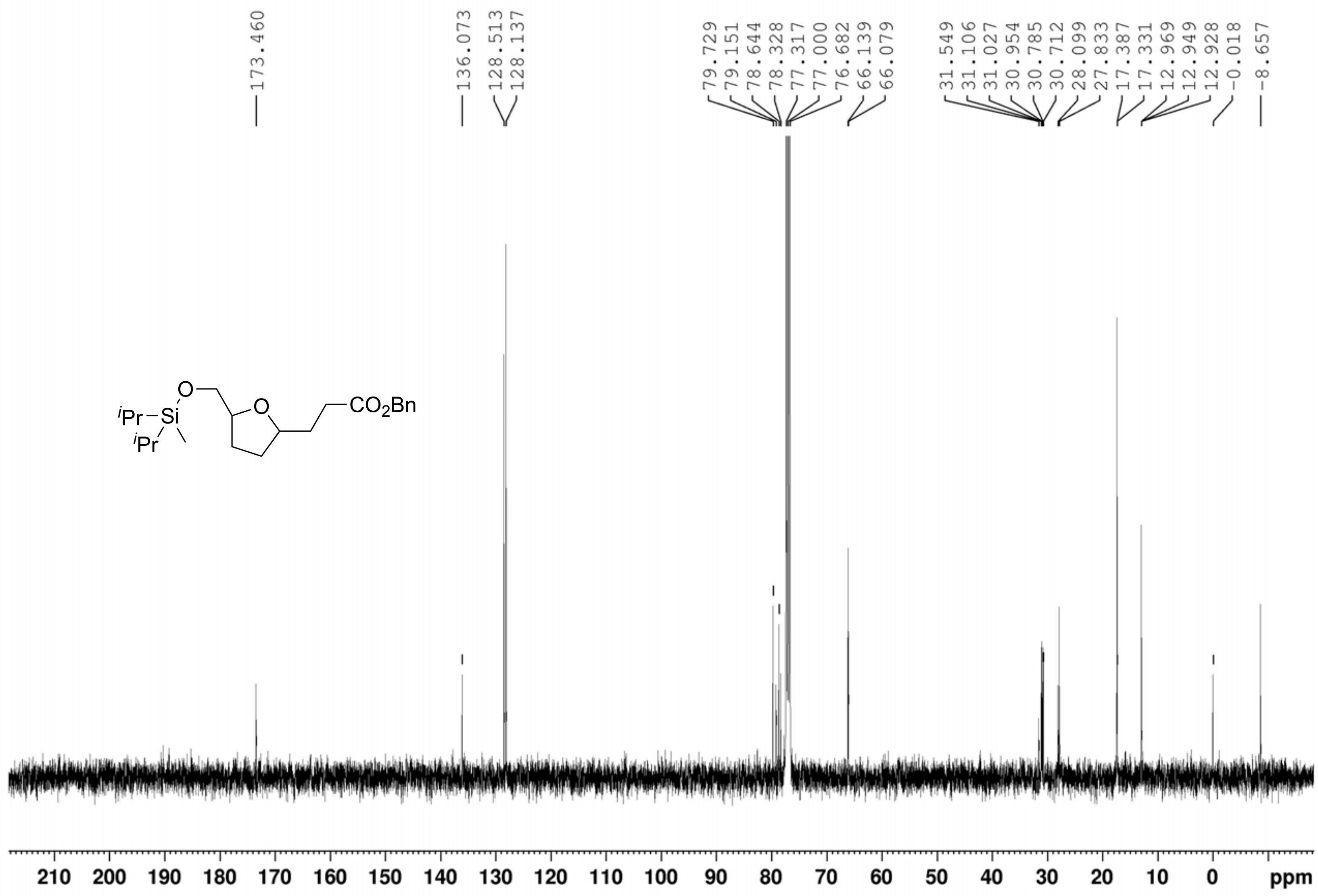

^{13}C NMR (100.6 MHz, $CDCl_3$) spectrum of **3ab-1**

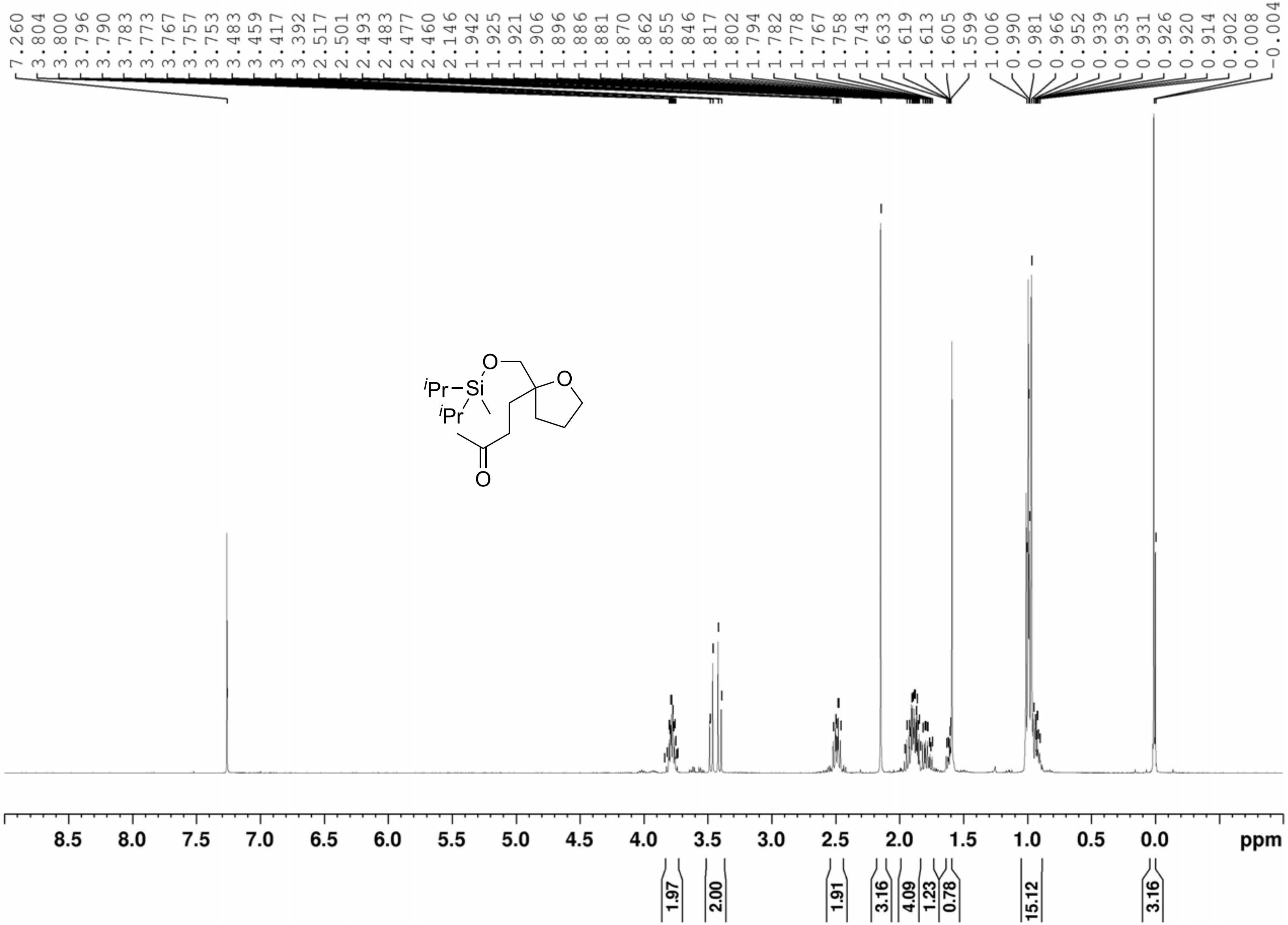

^{1}H NMR (400 MHz, CDCl$_3$) spectrum of **3ac**

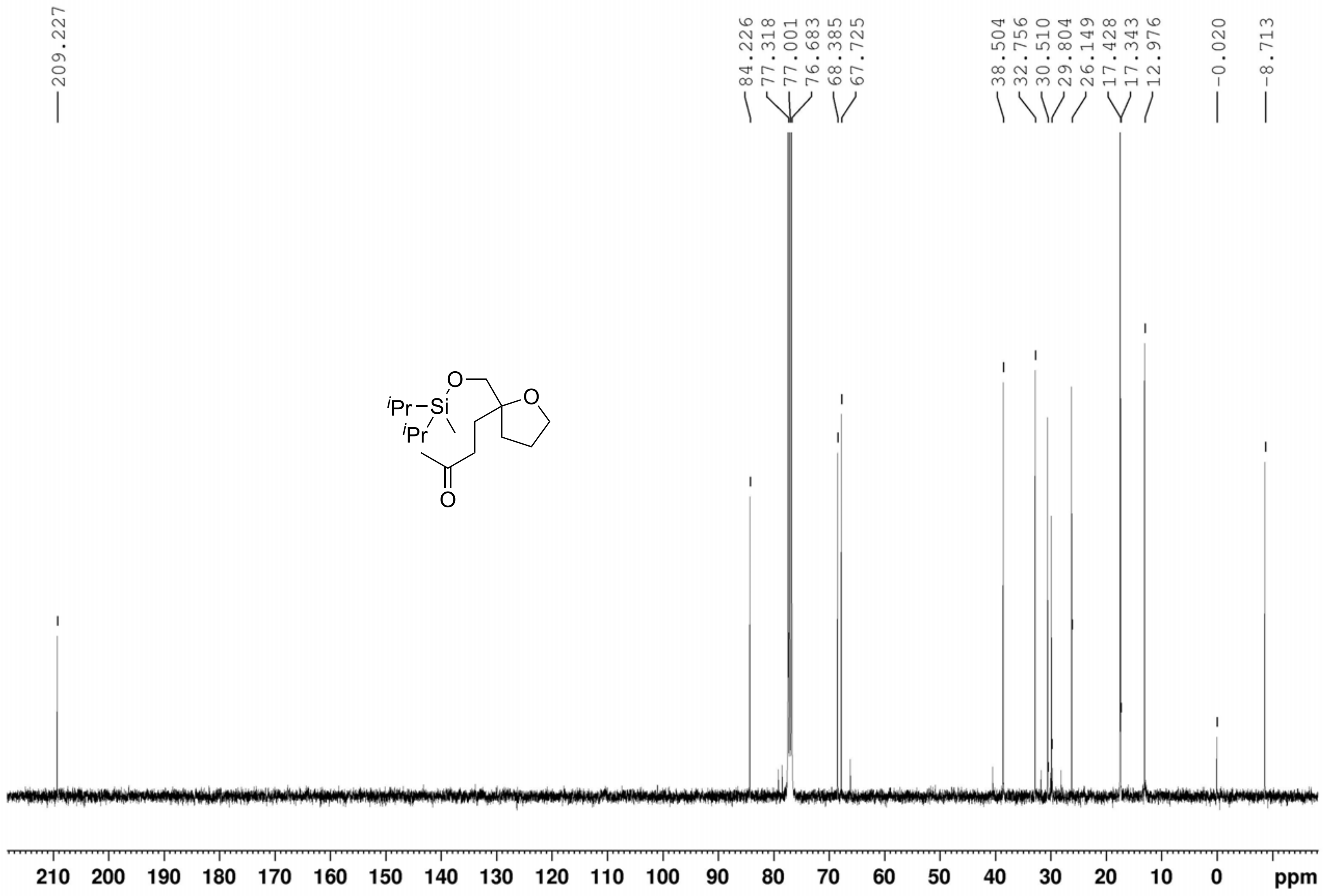

^{13}C NMR (100.6 MHz, $CDCl_3$) spectrum of **3ac**

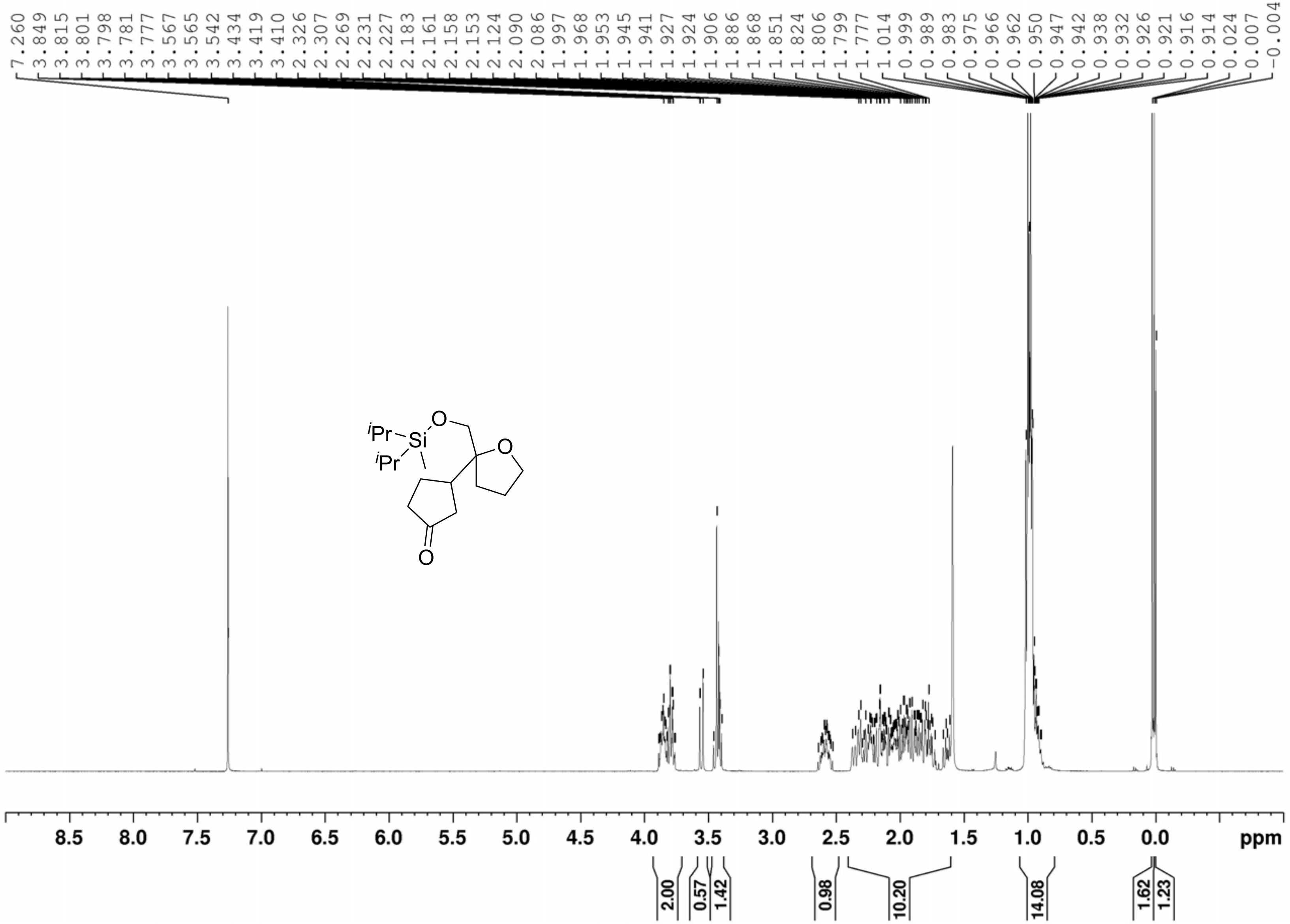

^{1}H NMR (400 MHz, CDCl3) spectrum of **3ad**

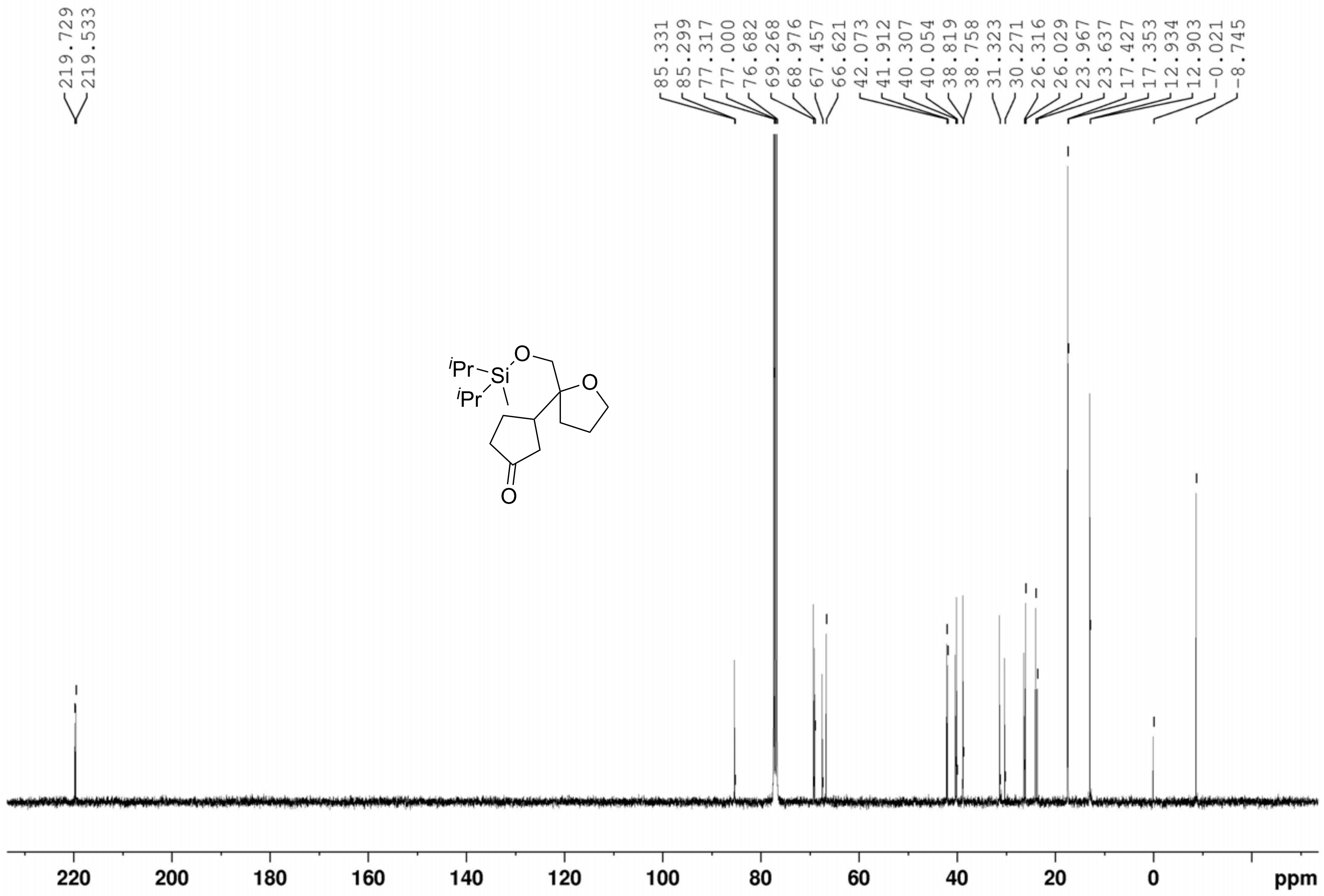

^{13}C NMR (100.6 MHz, $CDCl_3$) spectrum of **3ad**

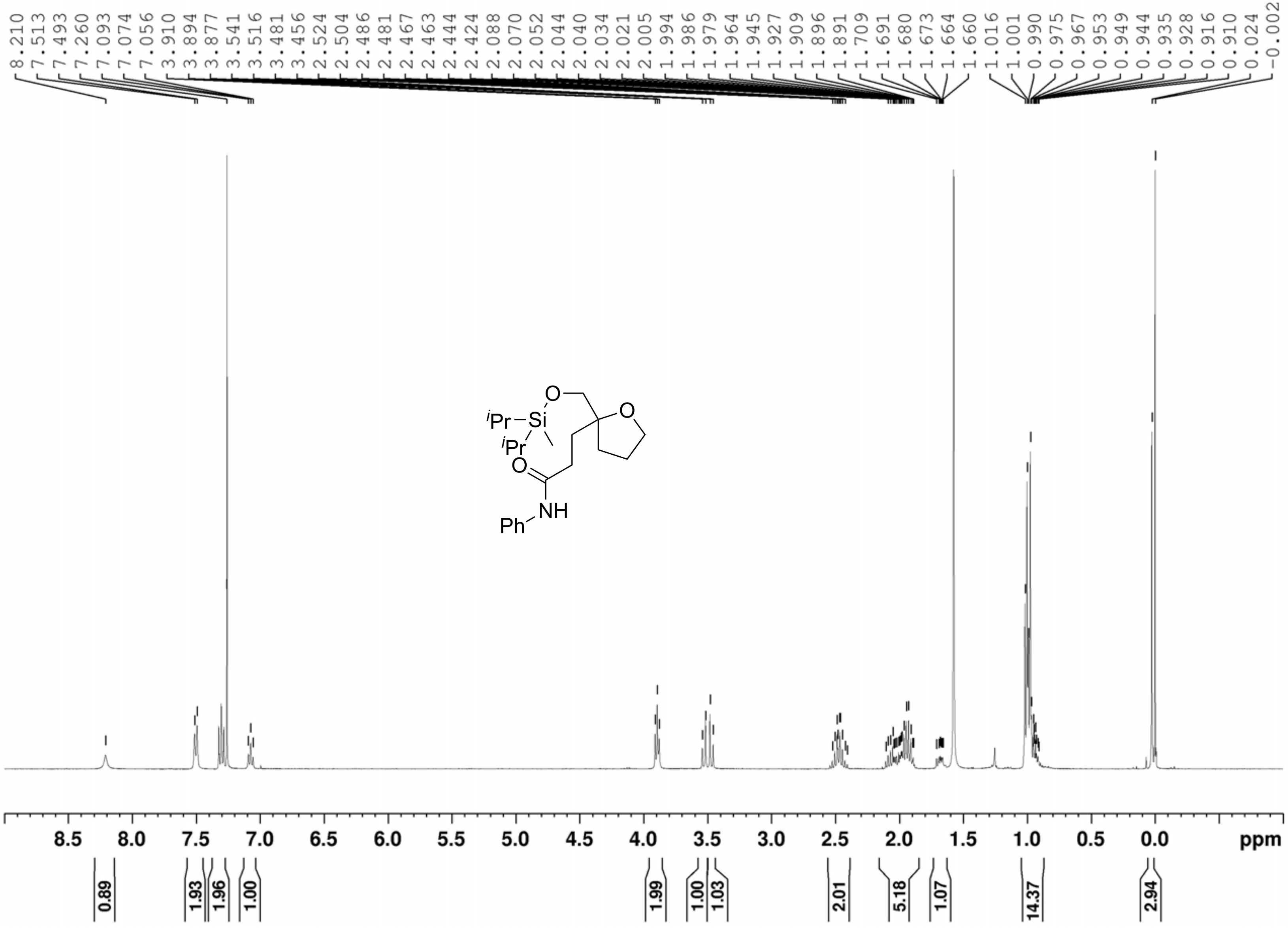

^{1}H NMR (400 MHz, $CDCl_3$) spectrum of **3ae**

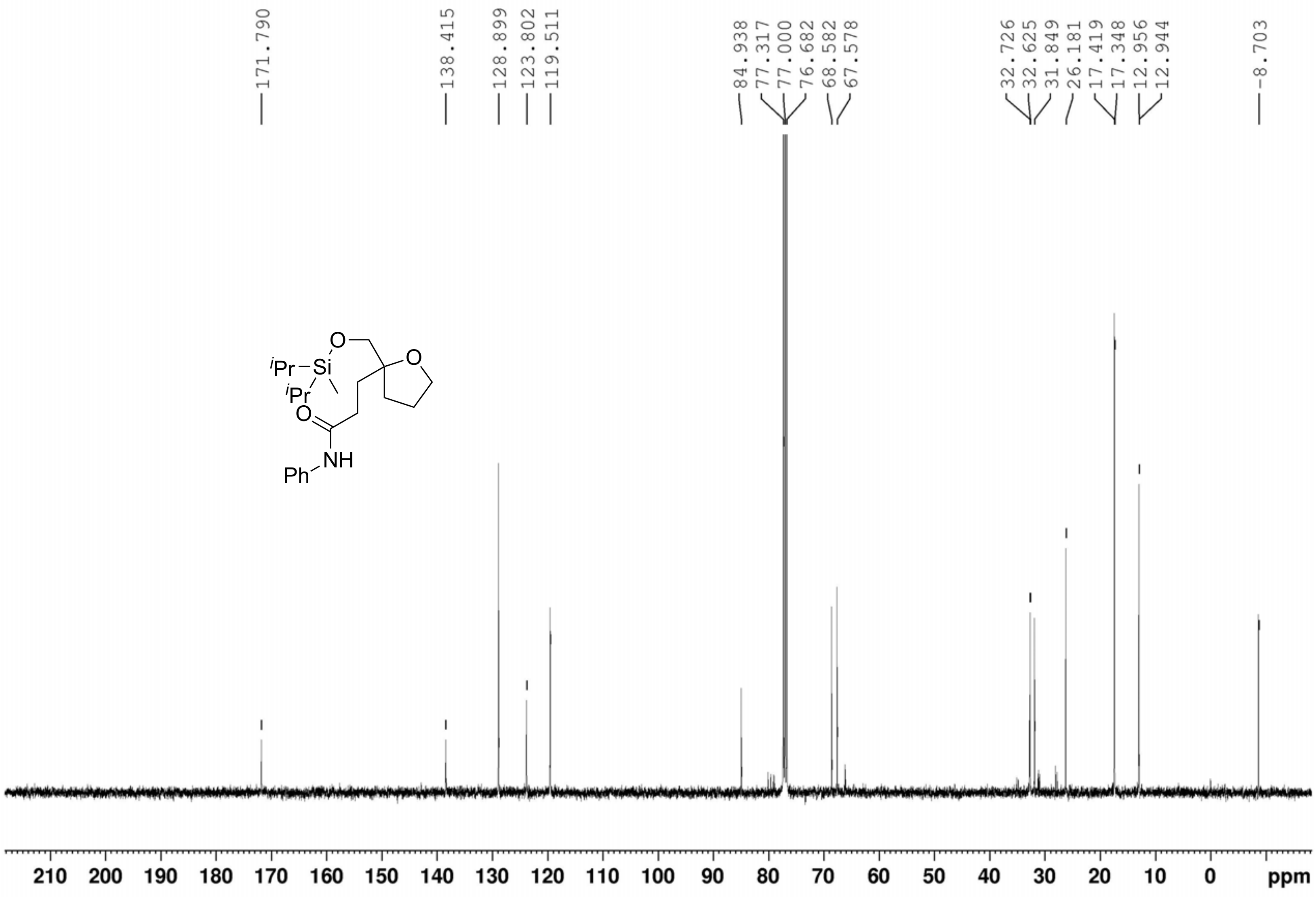

^{13}C NMR (100.6 MHz, $CDCl_3$) spectrum of **3ae**

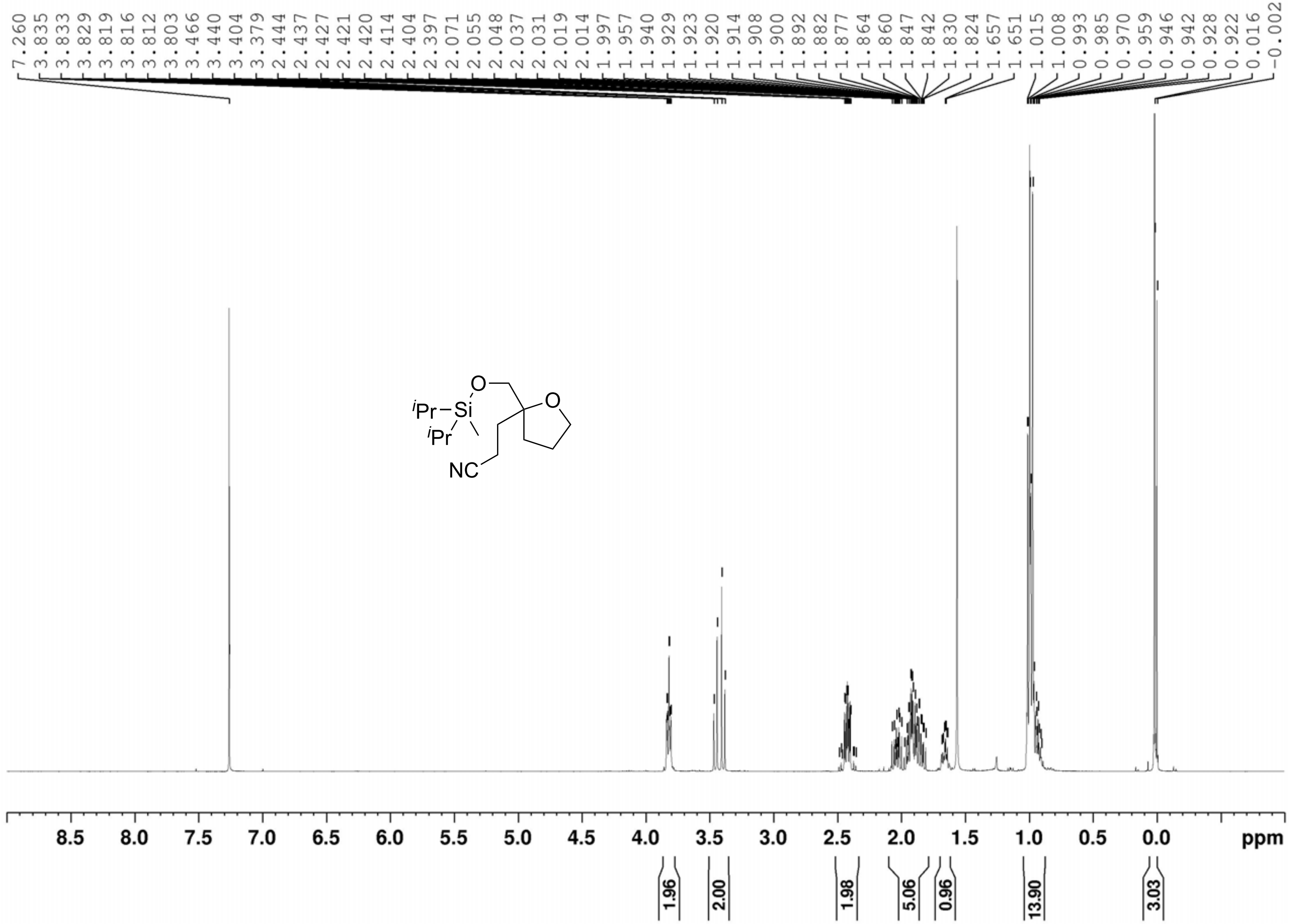

^{1}H NMR (400 MHz, $CDCl_3$) spectrum of **3af**

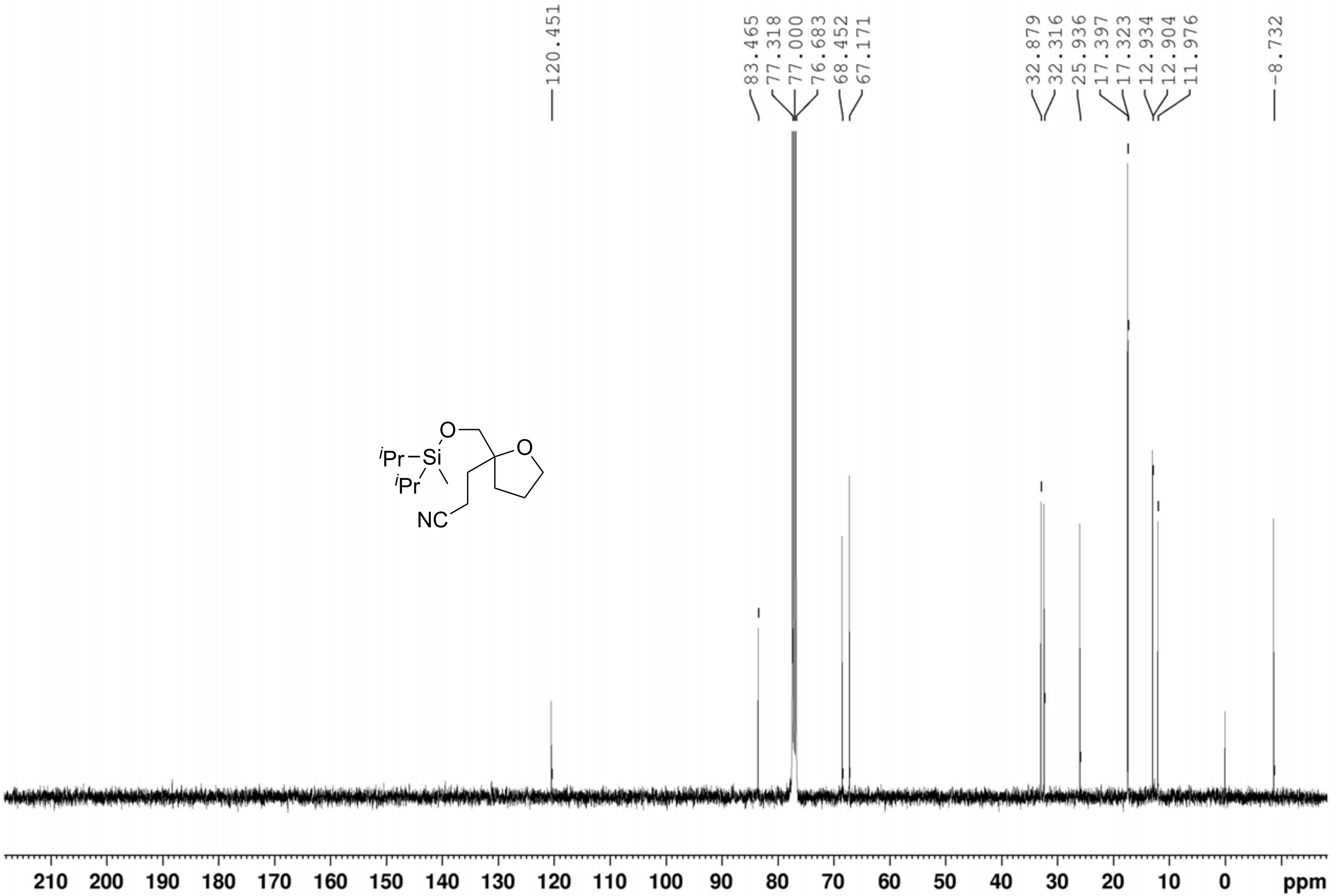

^{13}C NMR (100.6 MHz, $CDCl_3$) spectrum of **3af**

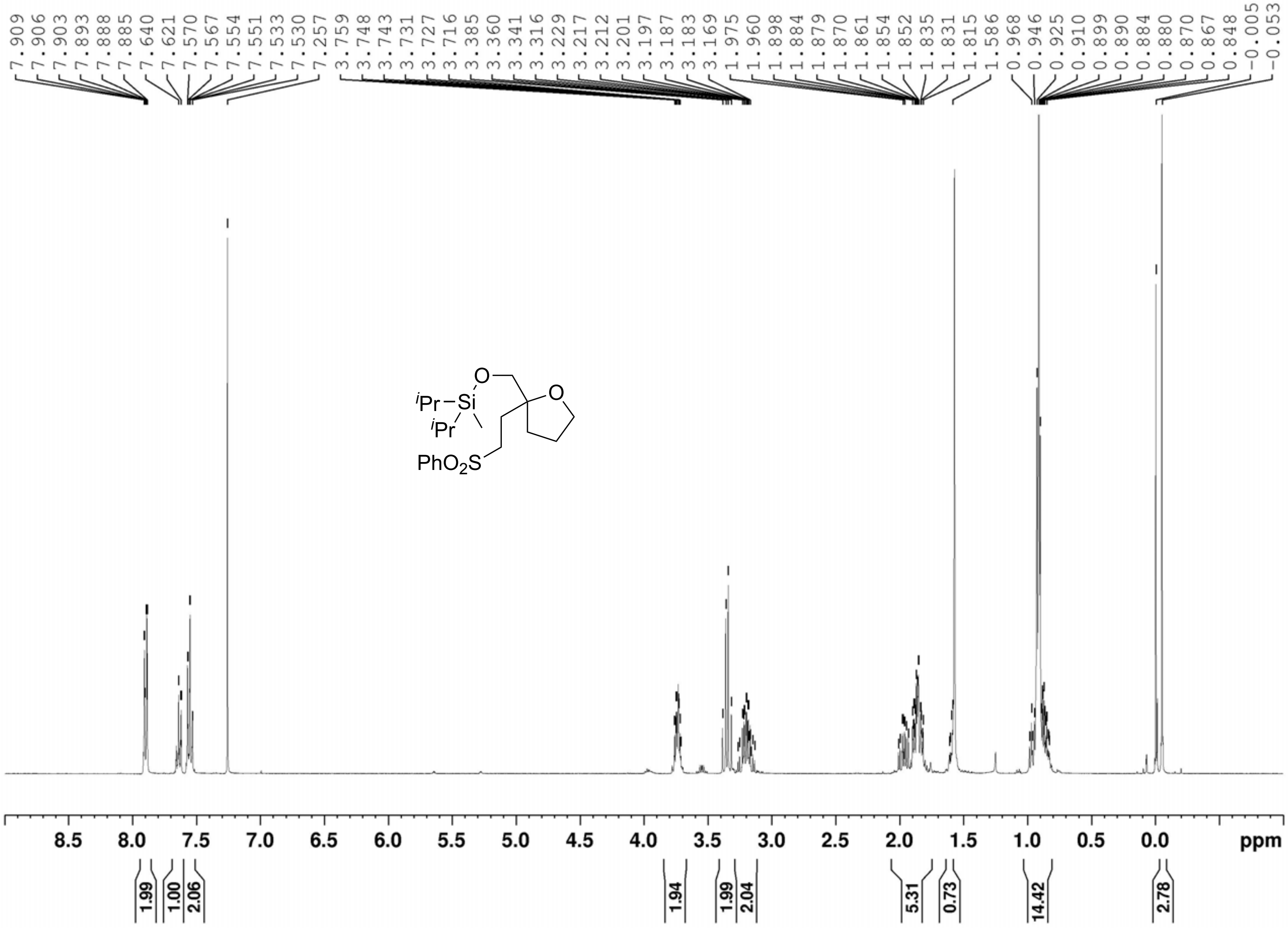

^{1}H NMR (400 MHz, CDCl$_3$) spectrum of **3ag**

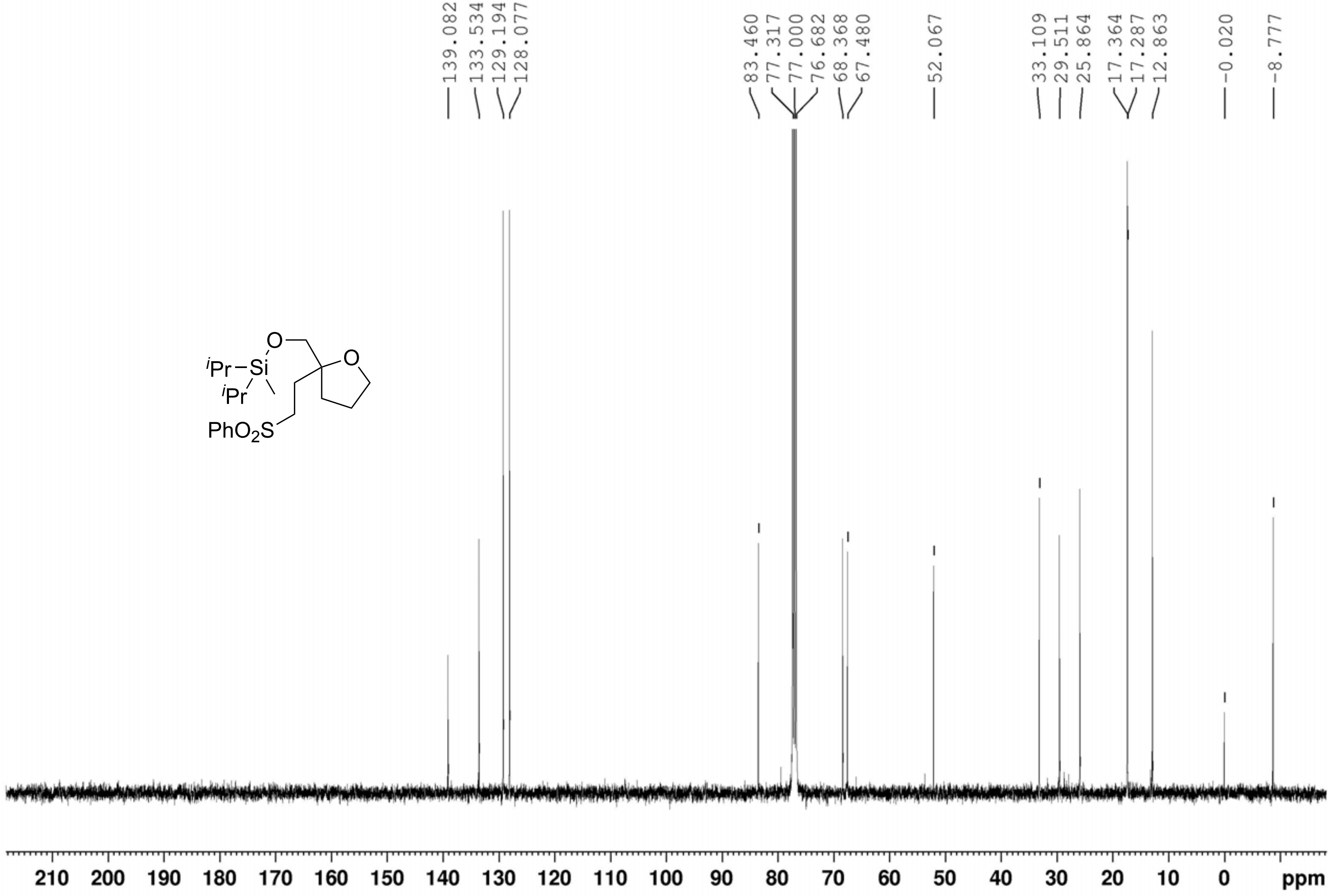

^{13}C NMR (100.6 MHz, $CDCl_3$) spectrum of **3ag**

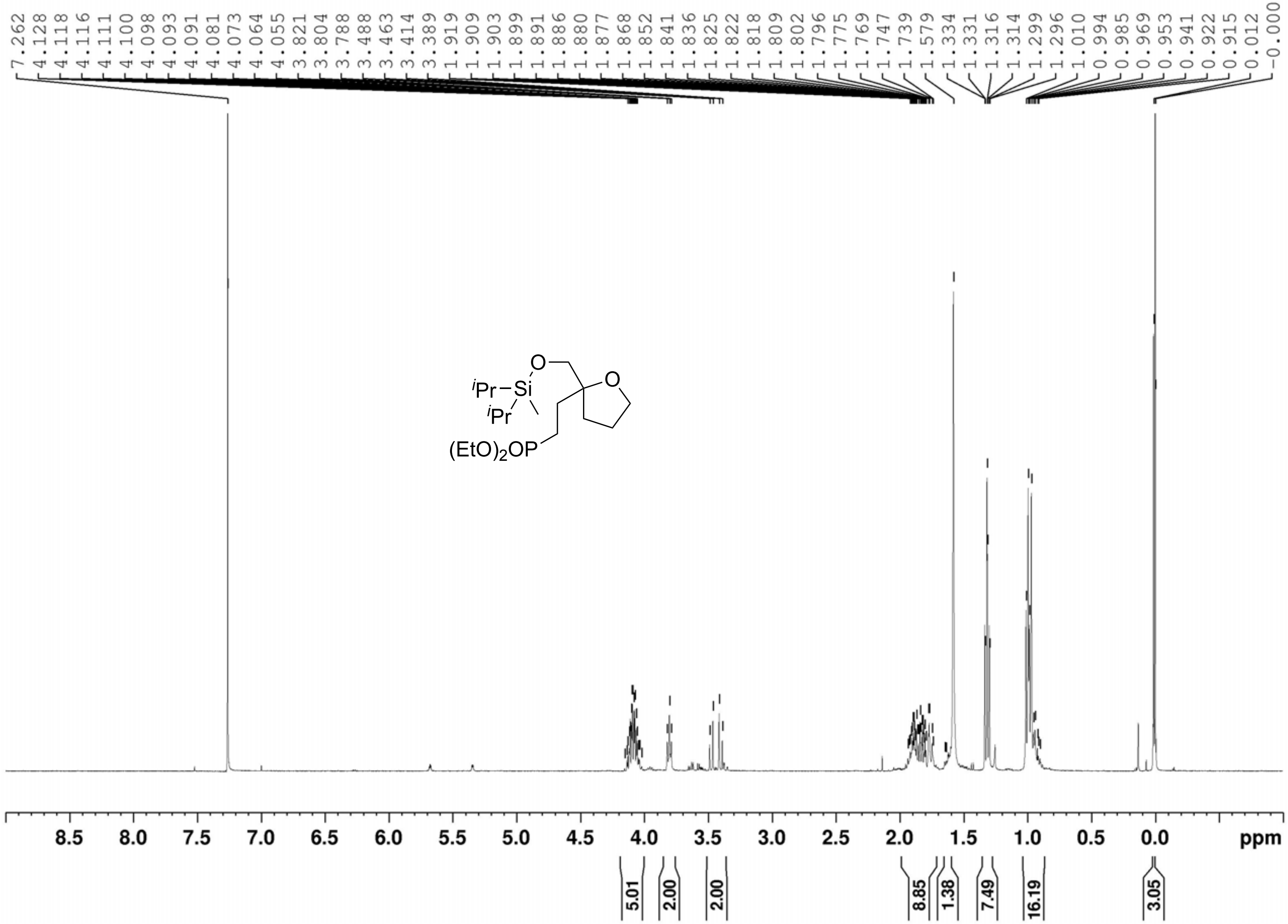

^{1}H NMR (400 MHz, $CDCl_3$) spectrum of **3ah**

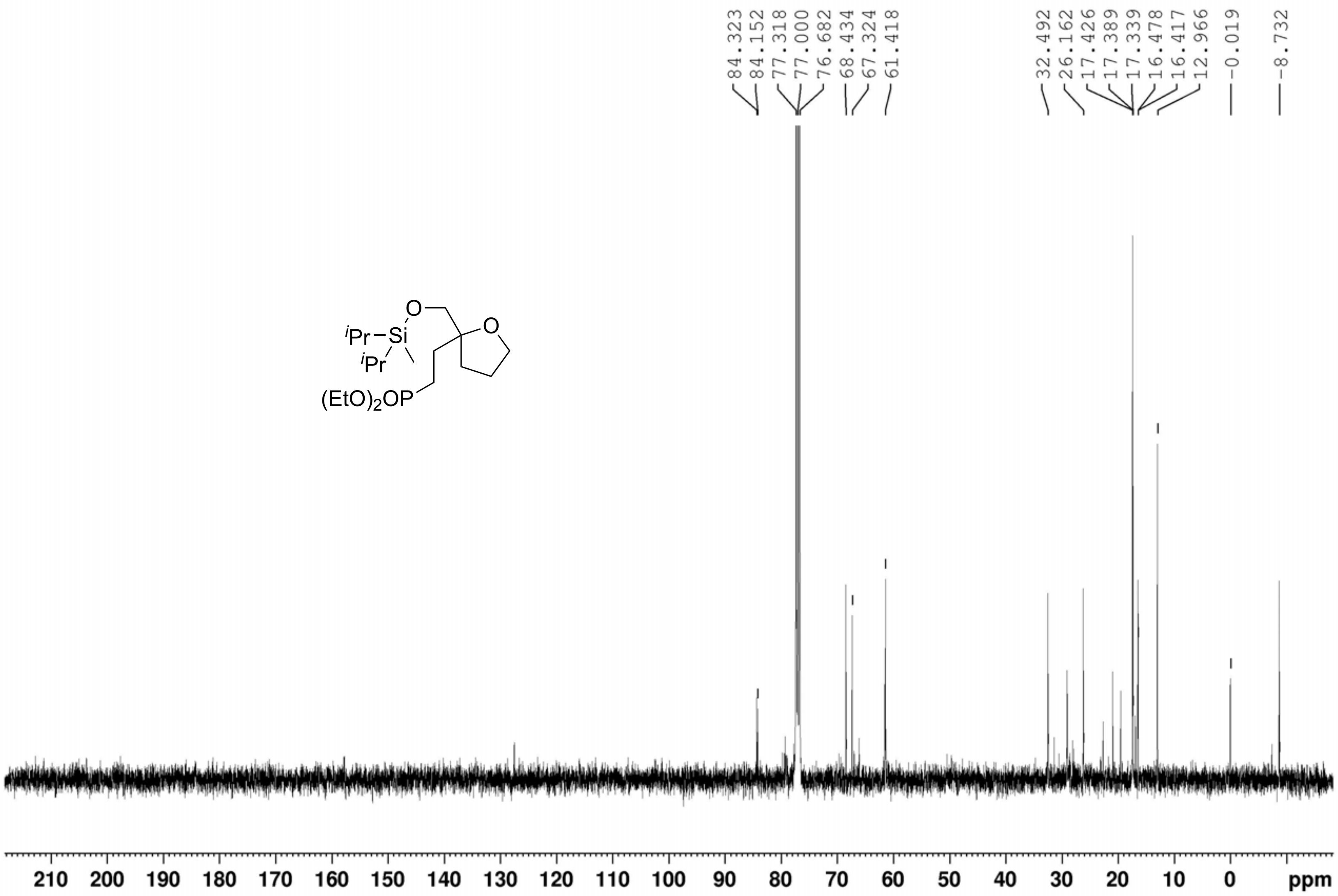

^{13}C NMR (100.6 MHz, $CDCl_3$) spectrum of **3ah**

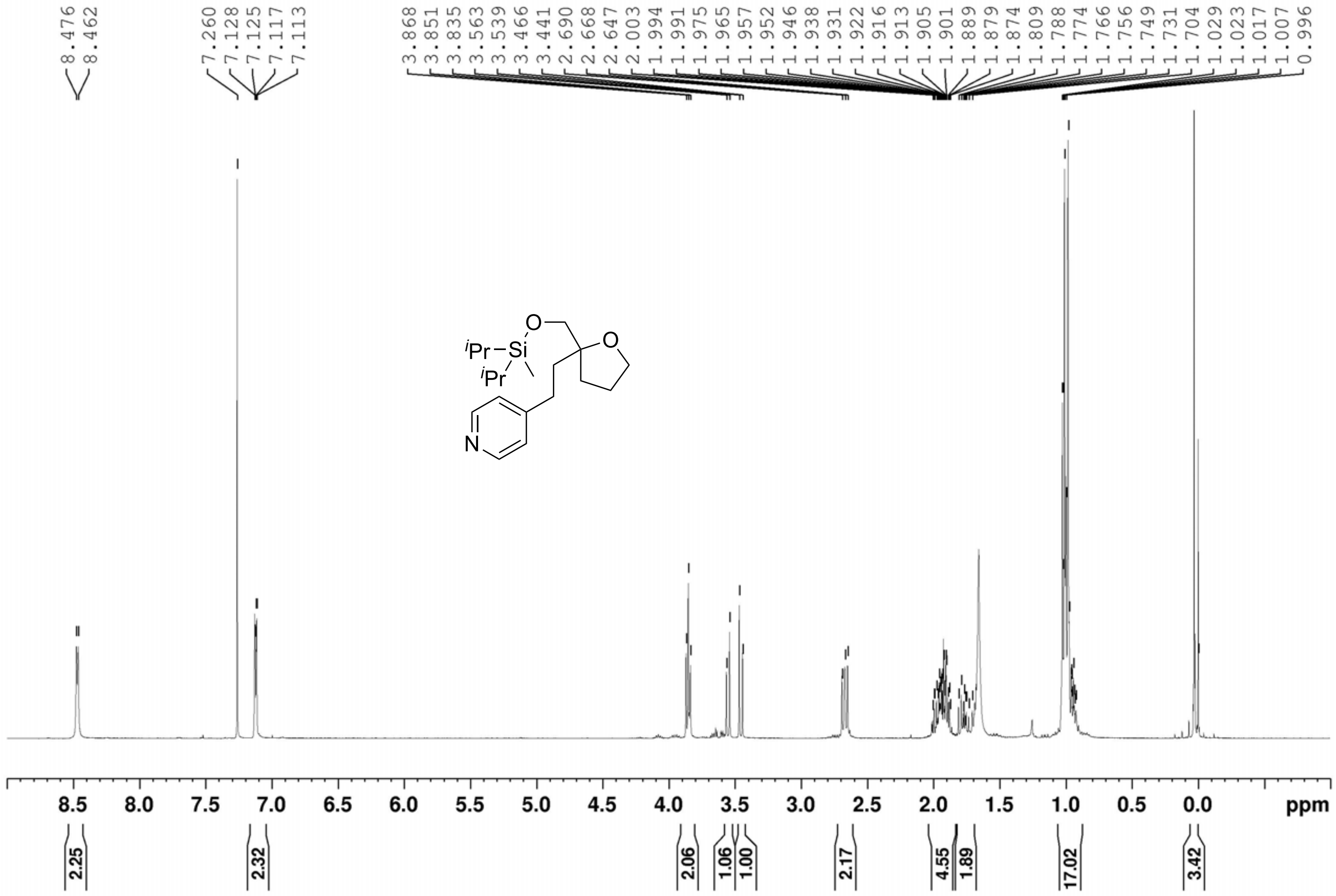

^{1}H NMR (400 MHz, $CDCl_3$) spectrum of **3ai**

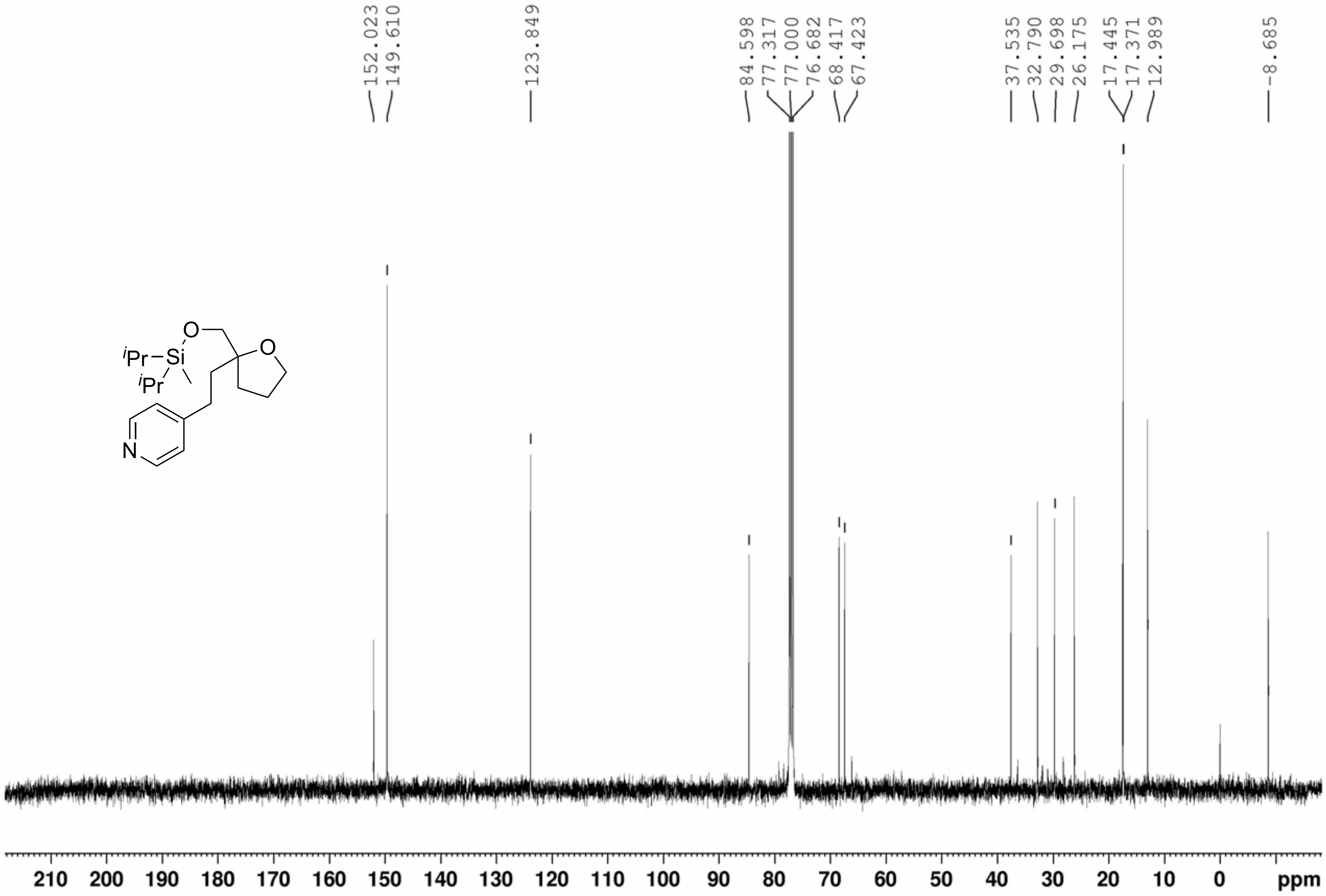

^{13}C NMR (100.6 MHz, $CDCl_3$) spectrum of **3ai**

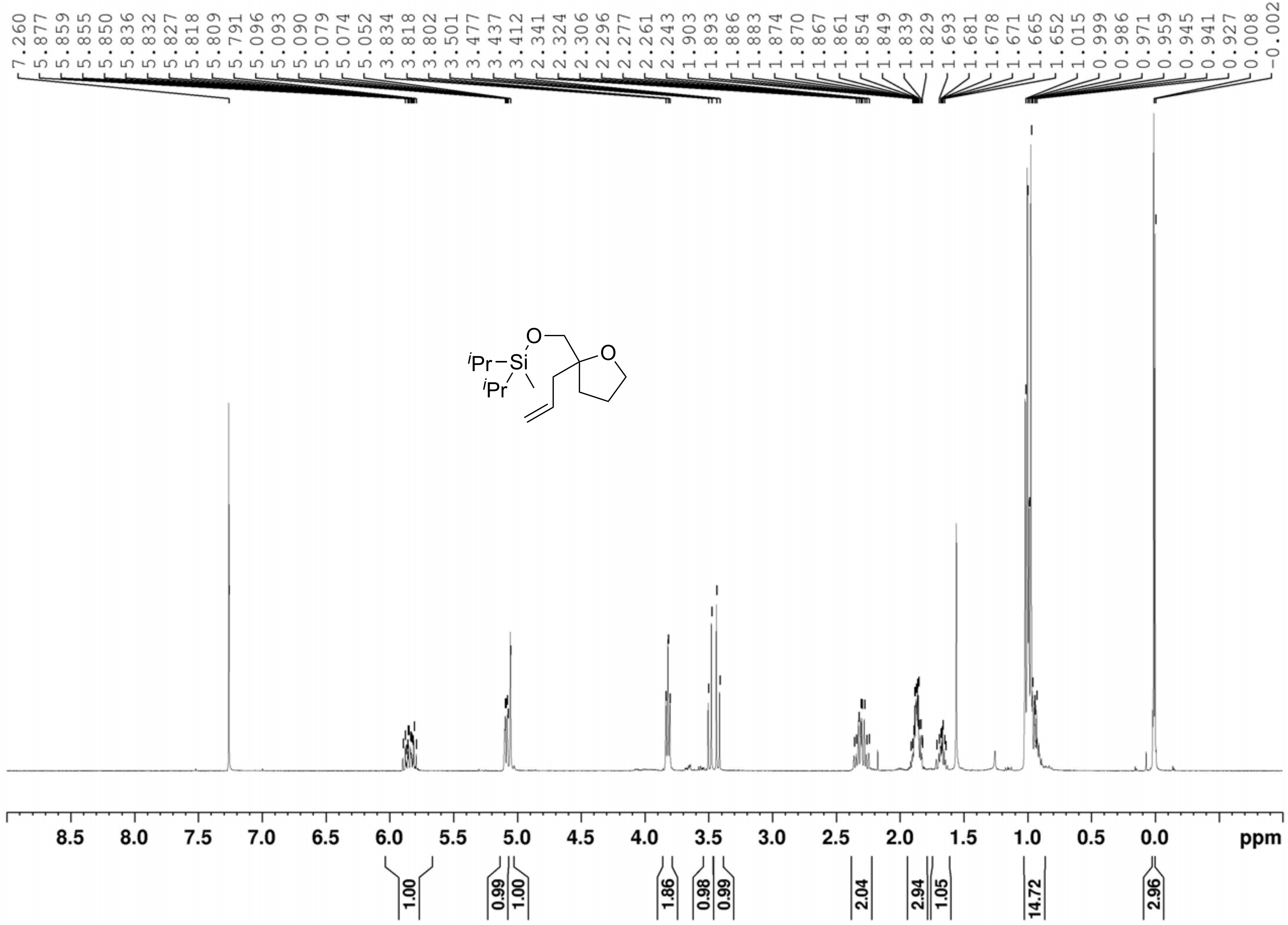

^{1}H NMR (400 MHz, $CDCl_3$) spectrum of **3aj**

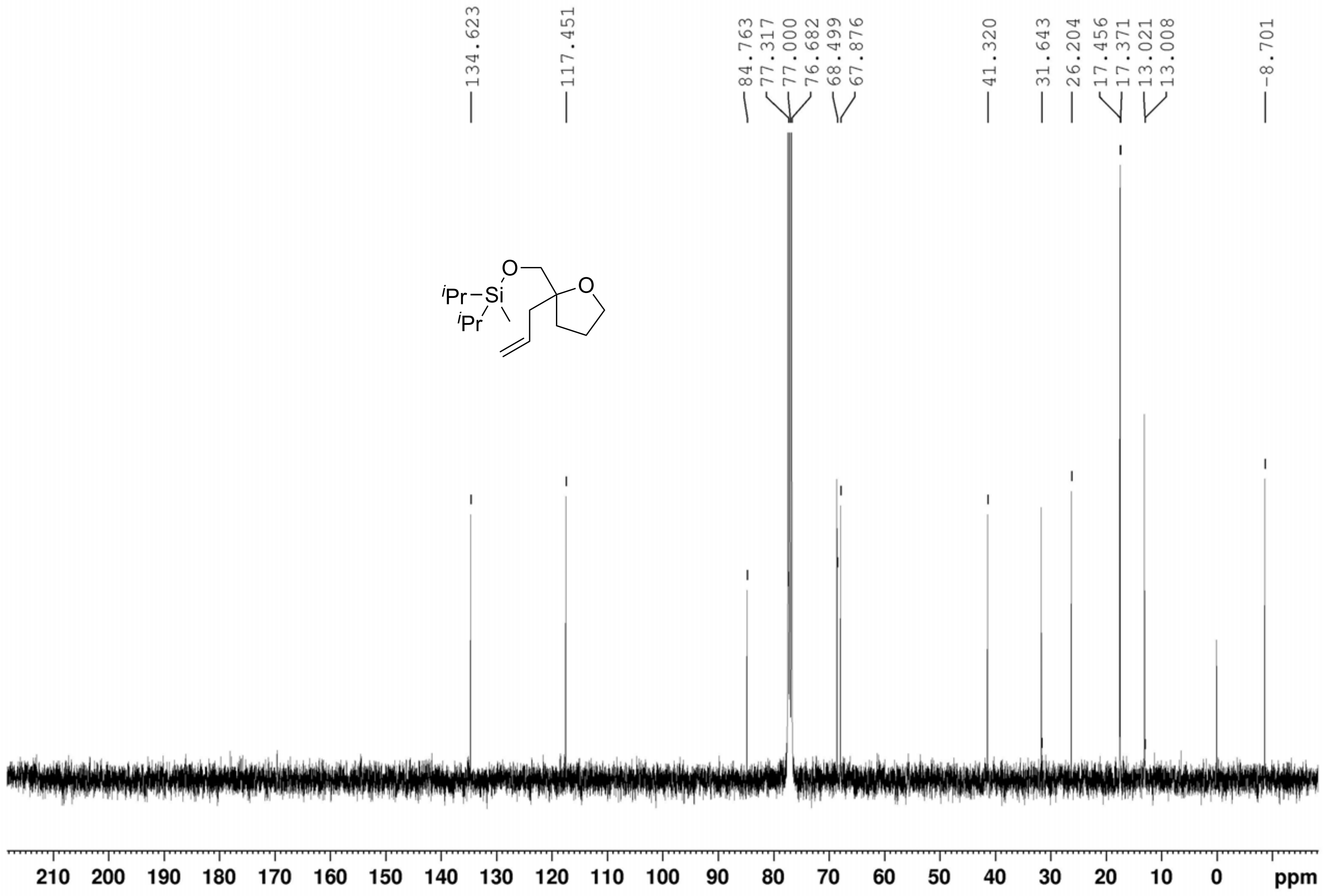

^{13}C NMR (100.6 MHz, $CDCl_3$) spectrum of **3aj**

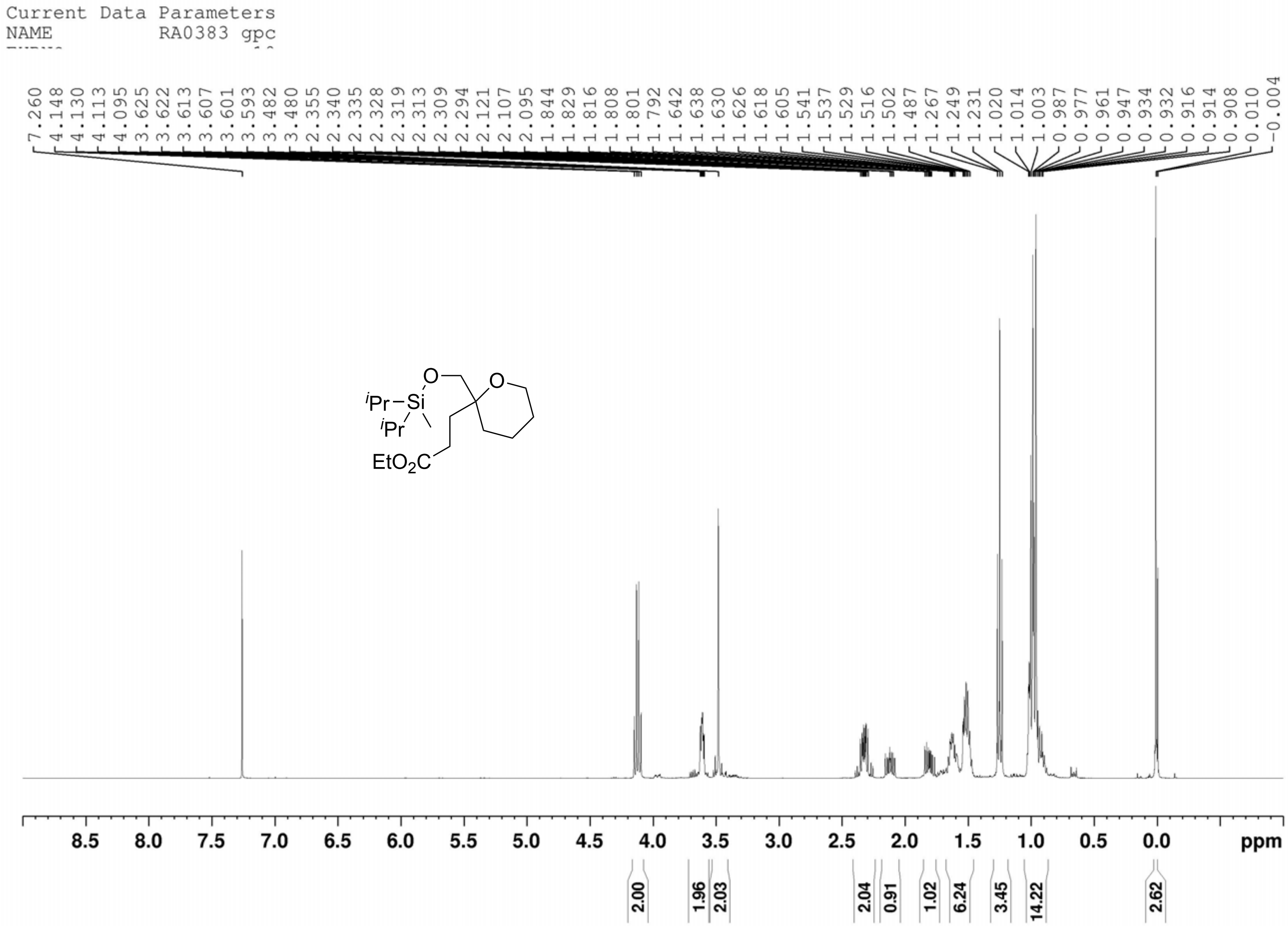

^{1}H NMR (400 MHz, CDCl$_3$) spectrum of **3ba**

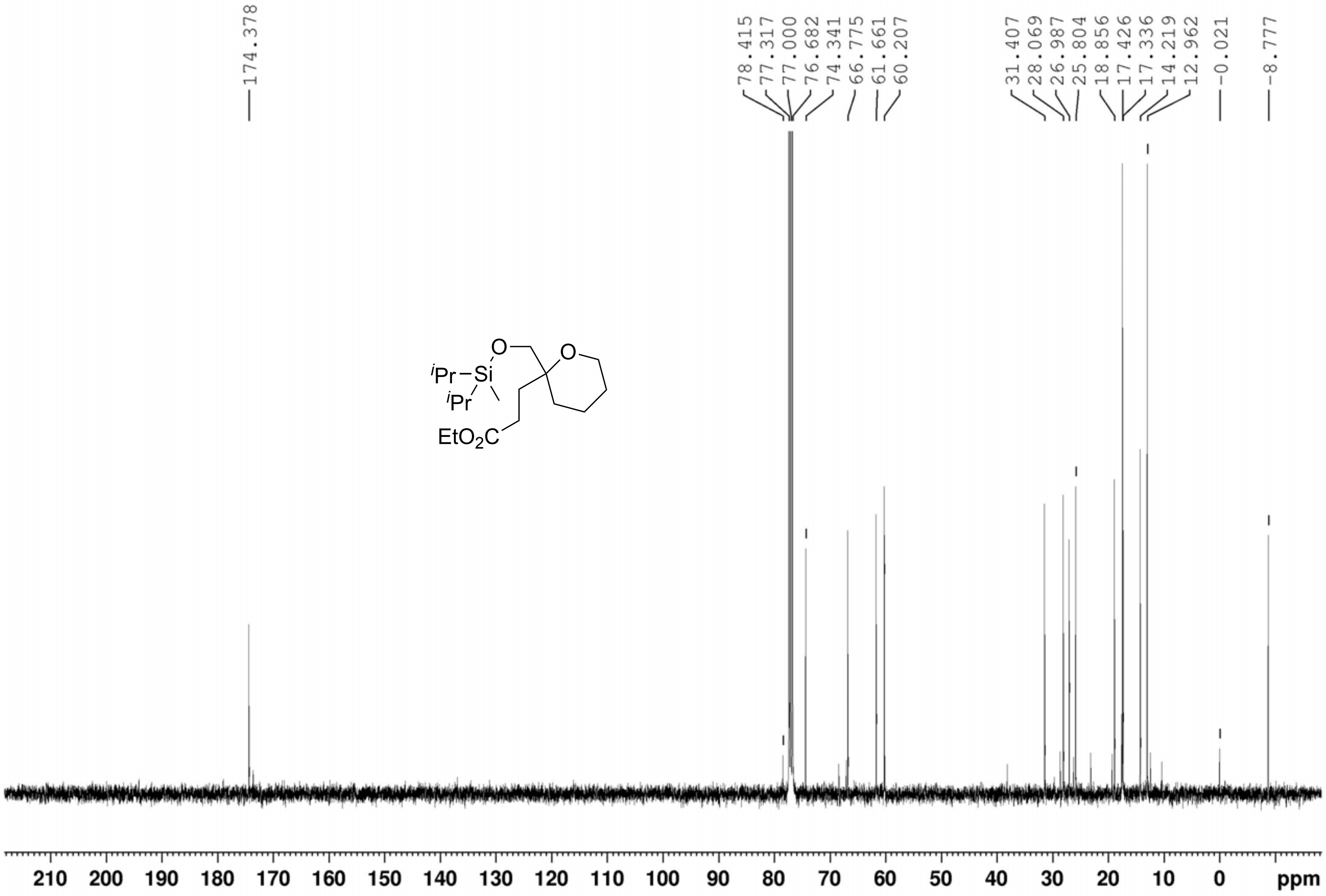

^{13}C NMR (100.6 MHz, $CDCl_3$) spectrum of **3ba**

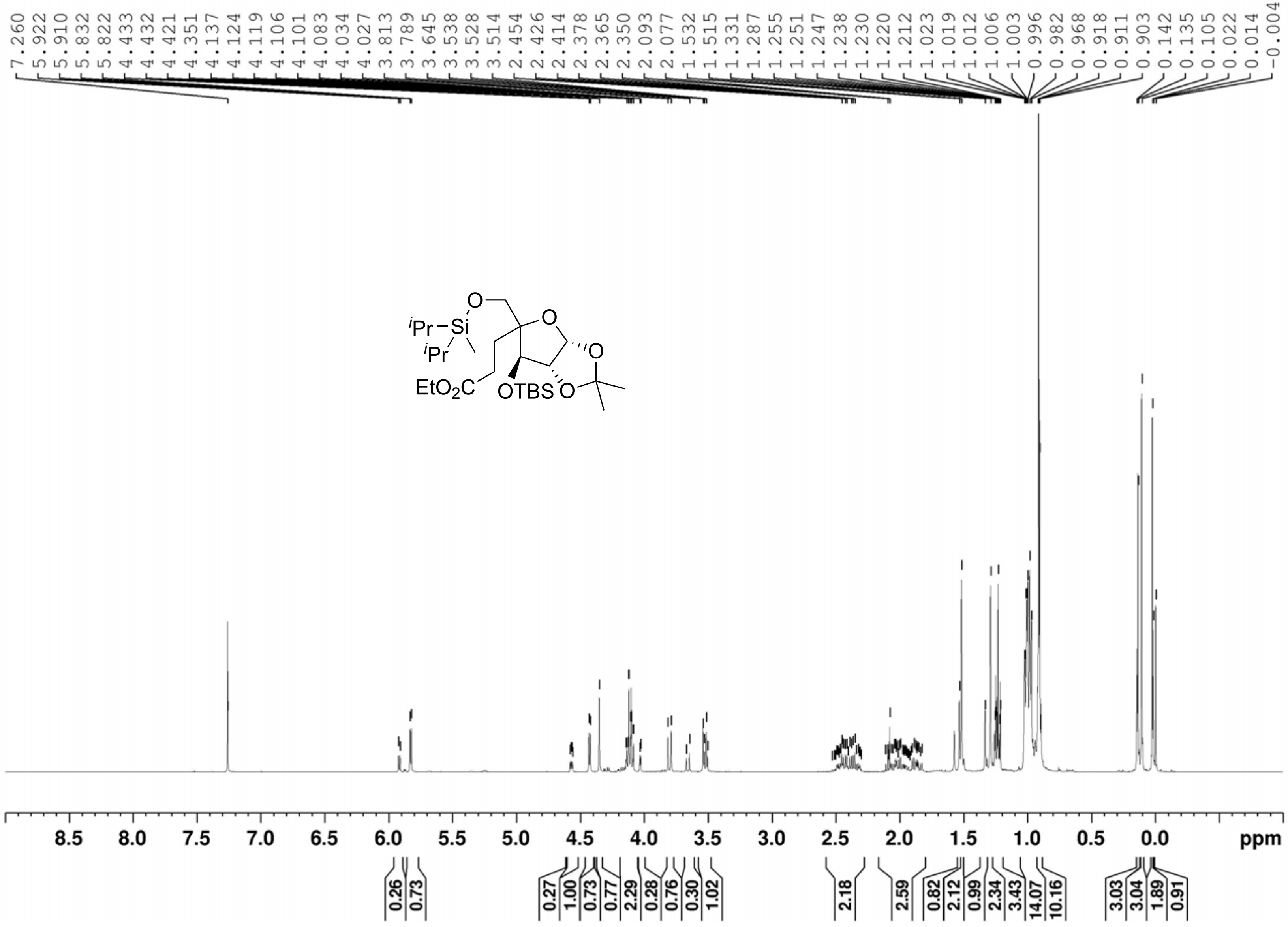

^{1}H NMR (400 MHz, $CDCl_3$) spectrum of **3ca**

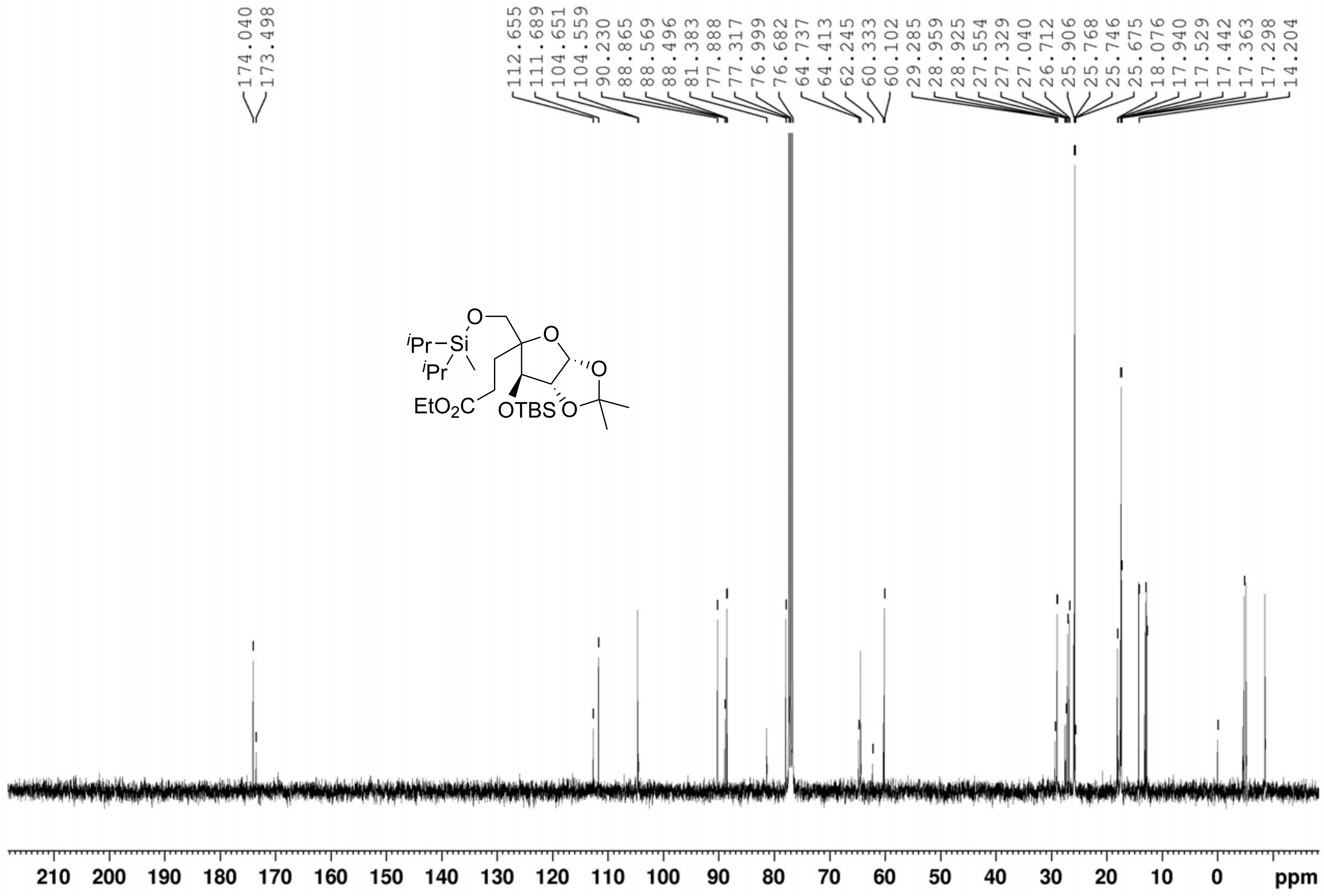

^{13}C NMR (100.6 MHz, $CDCl_3$) spectrum of **3ca**

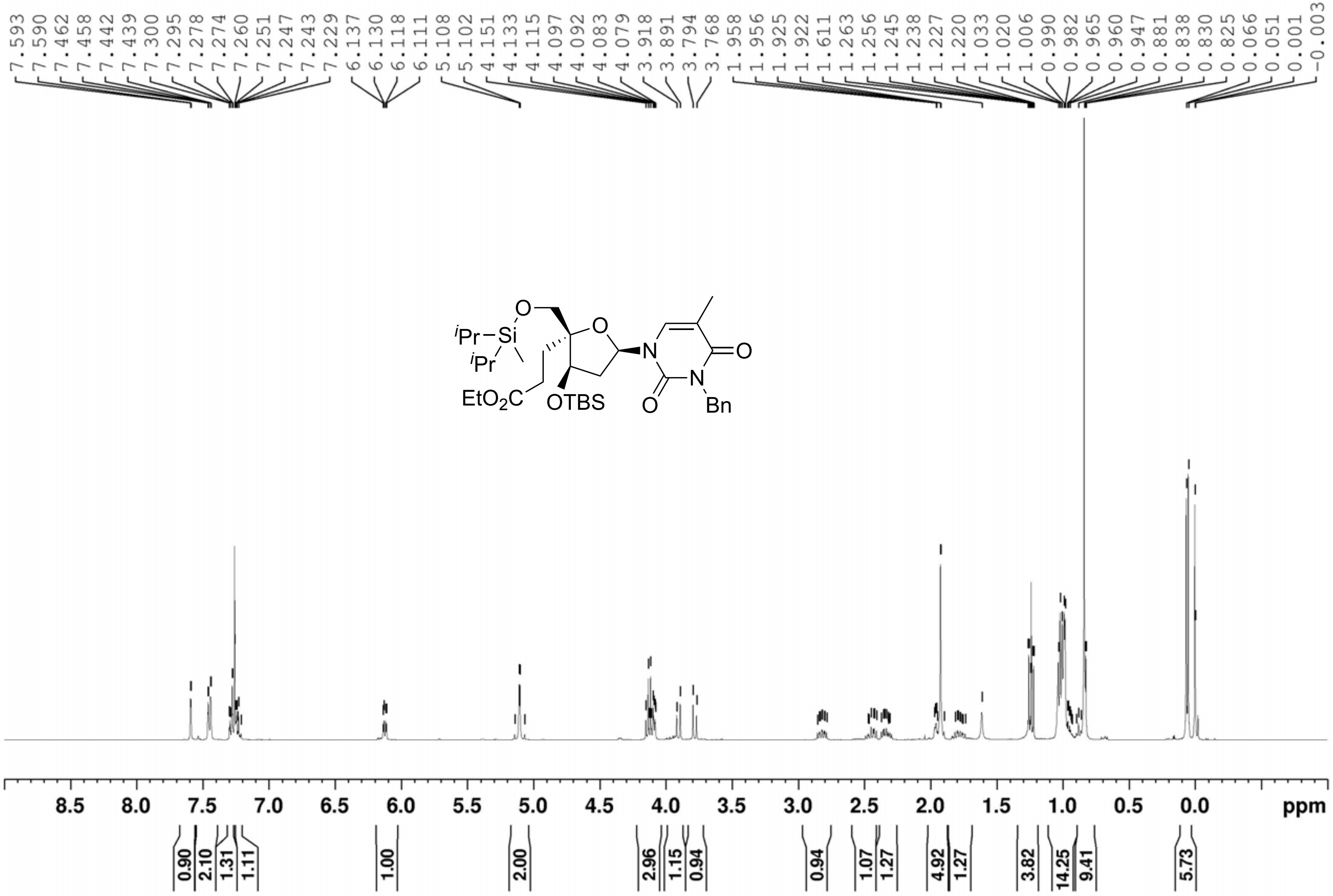

^{1}H NMR (400 MHz, $CDCl_3$) spectrum of **3da**

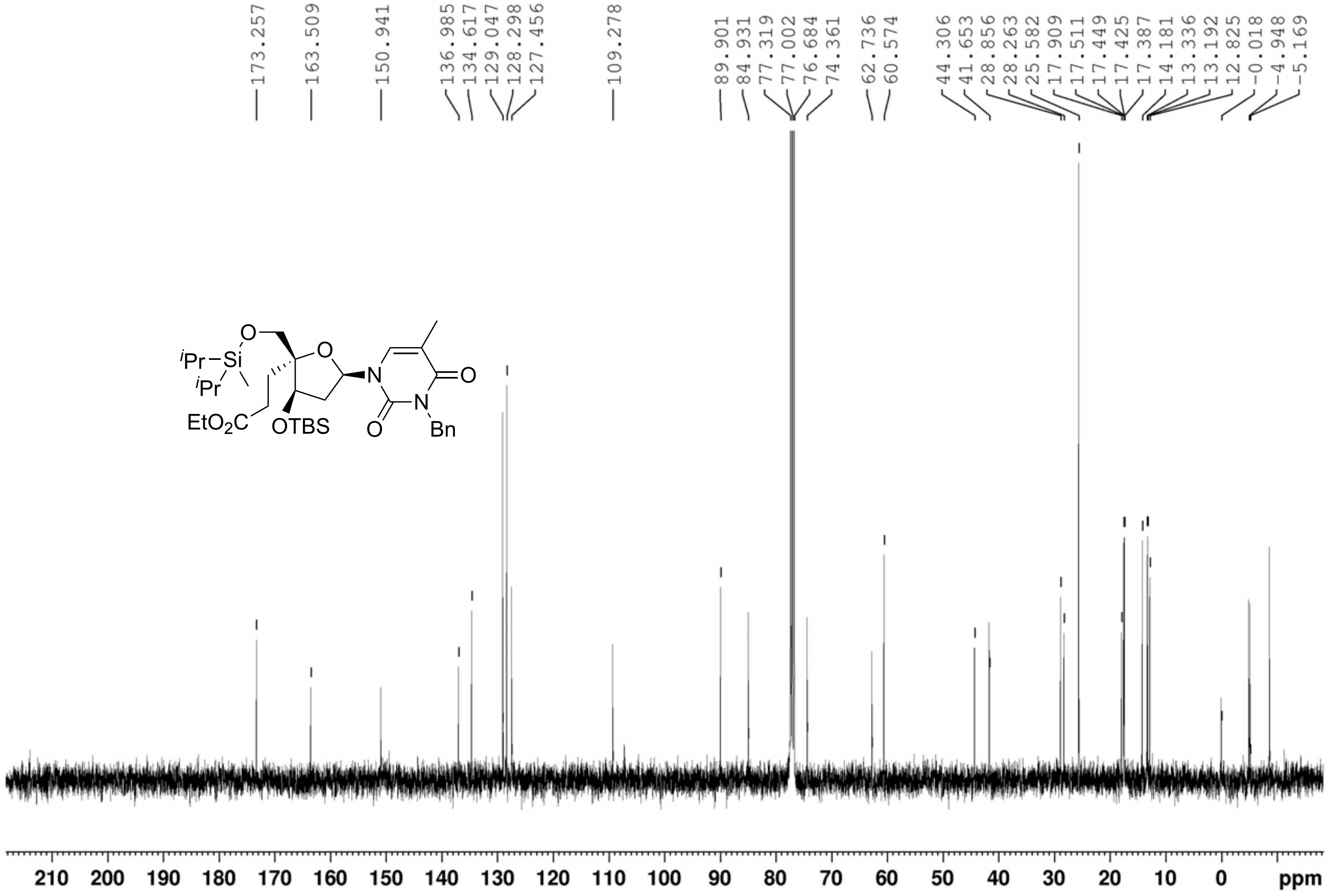

^{13}C NMR (100.6 MHz, $CDCl_3$) spectrum of **3da**

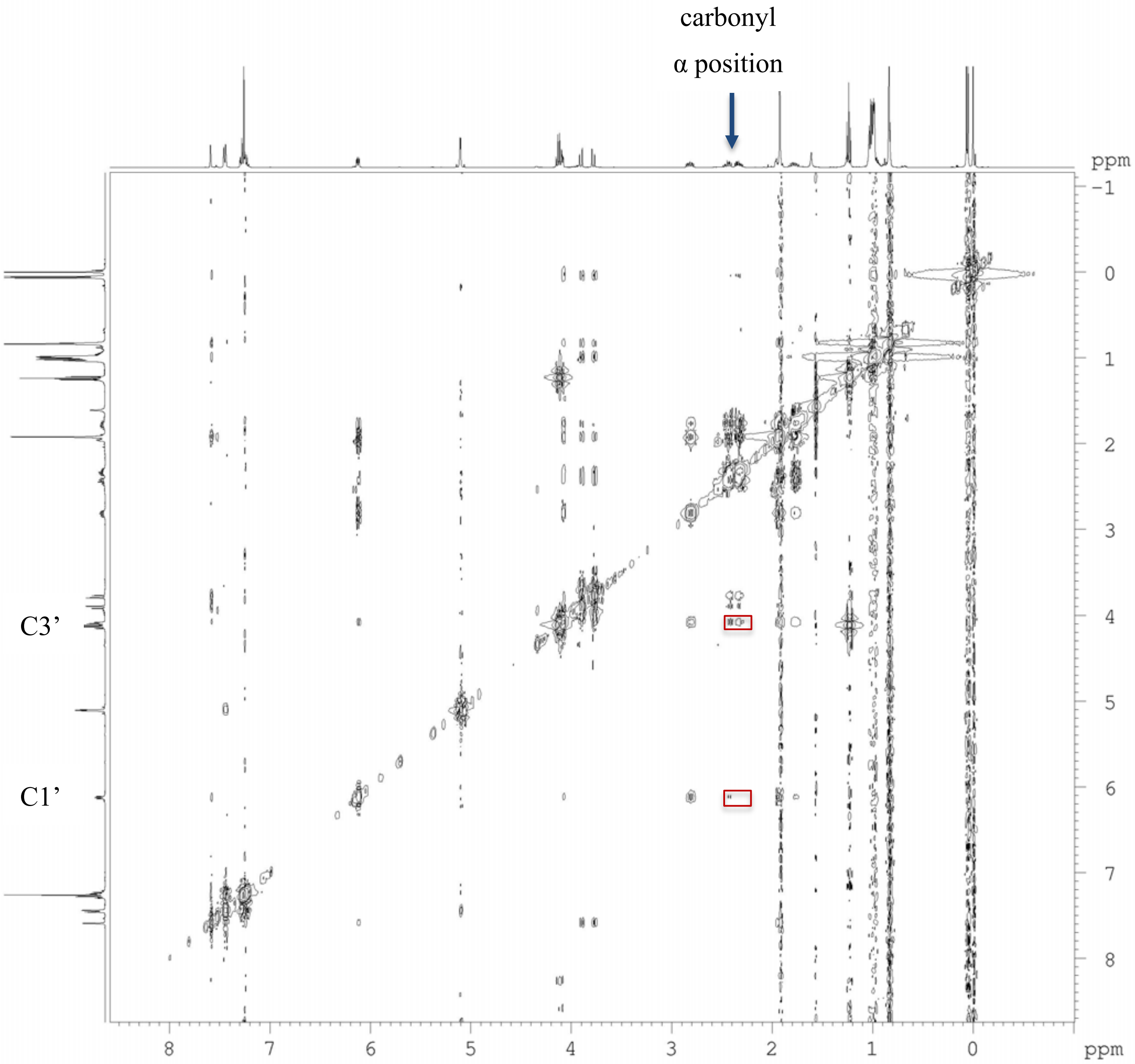

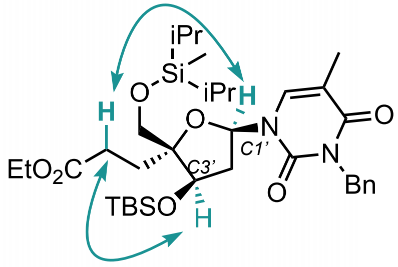

NOESY spectrum of **3da**

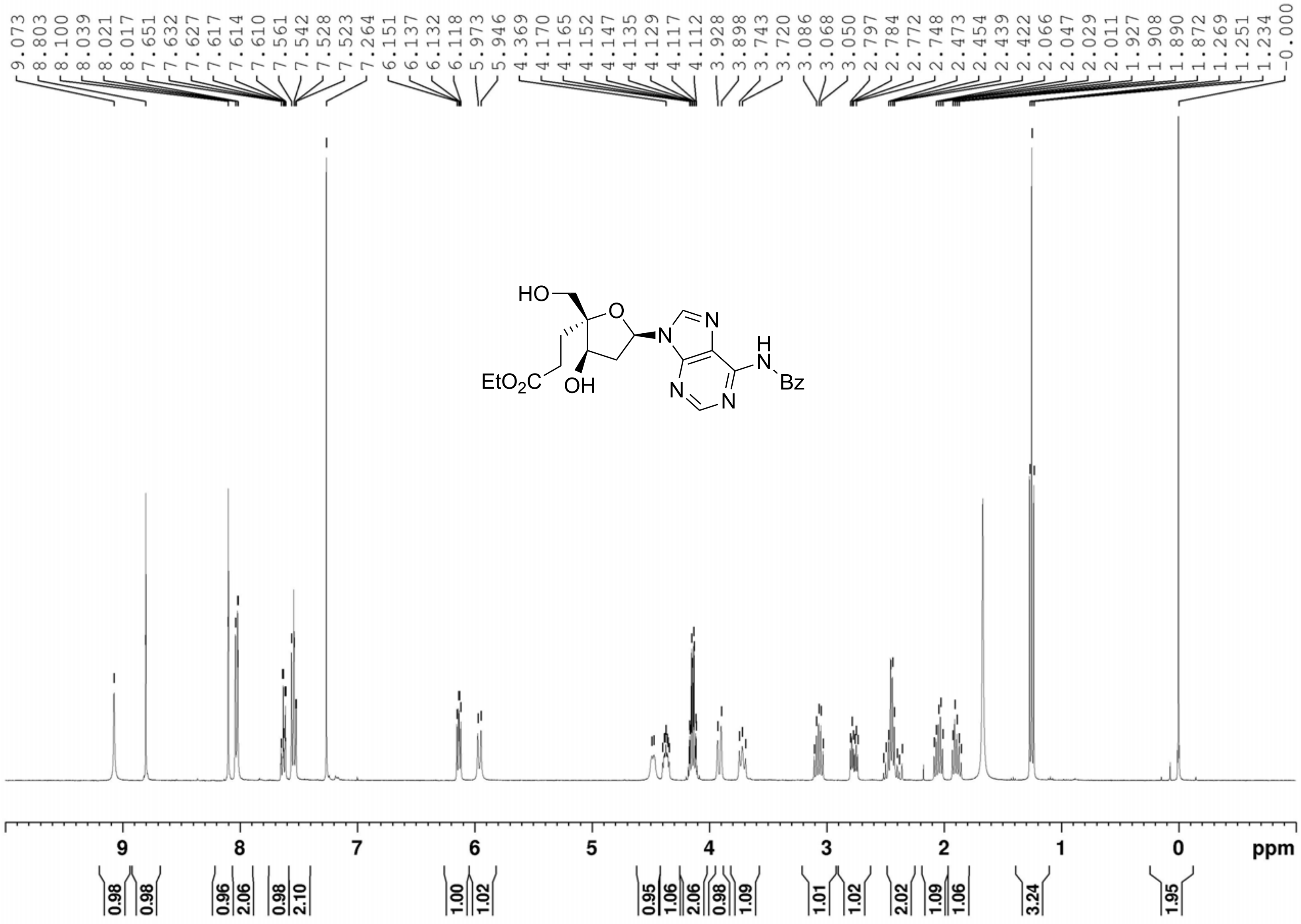

^{1}H NMR (400 MHz, $CDCl_3$) spectrum of **3fa**

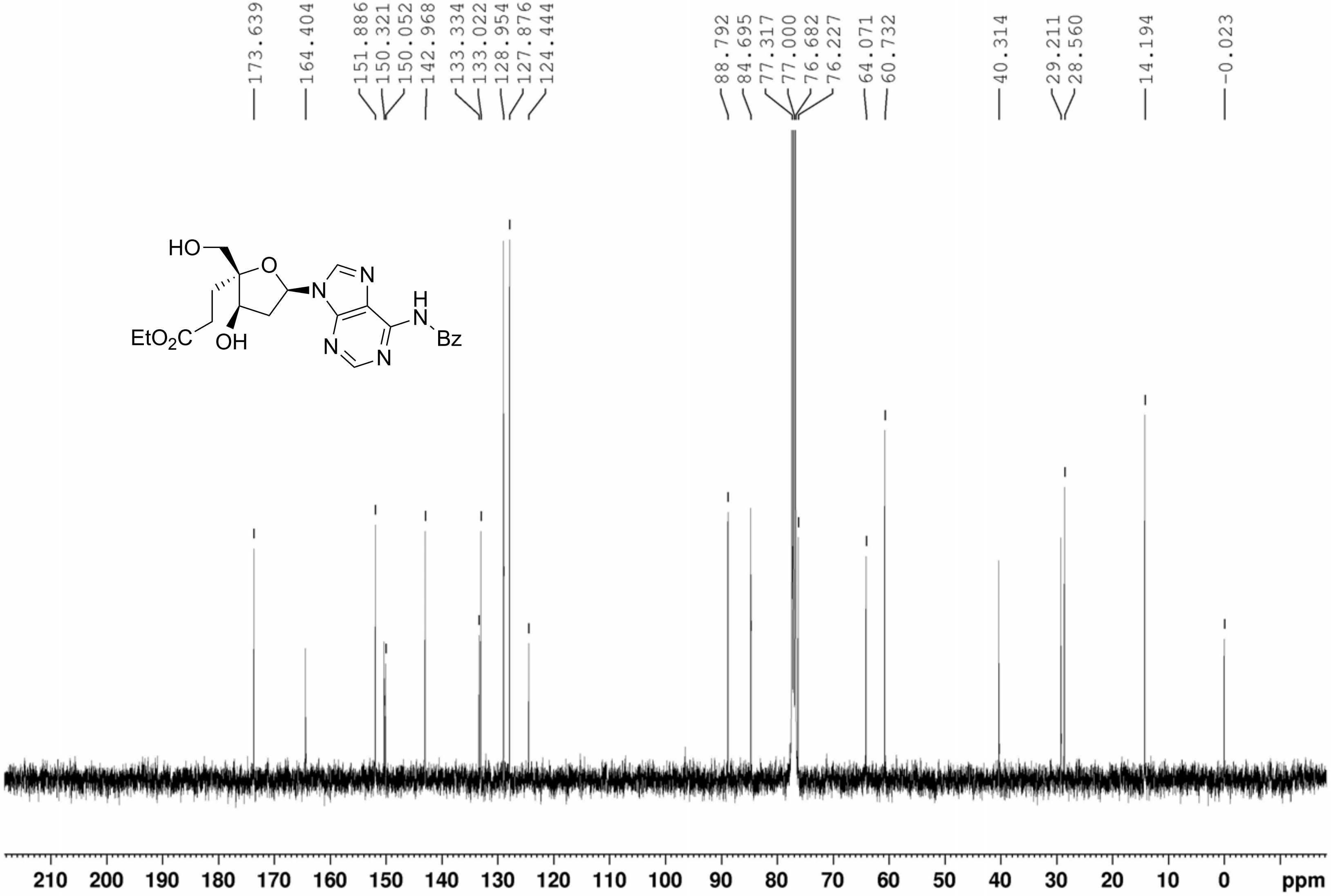

^{13}C NMR (100.6 MHz, $CDCl_3$) spectrum of **3fa**

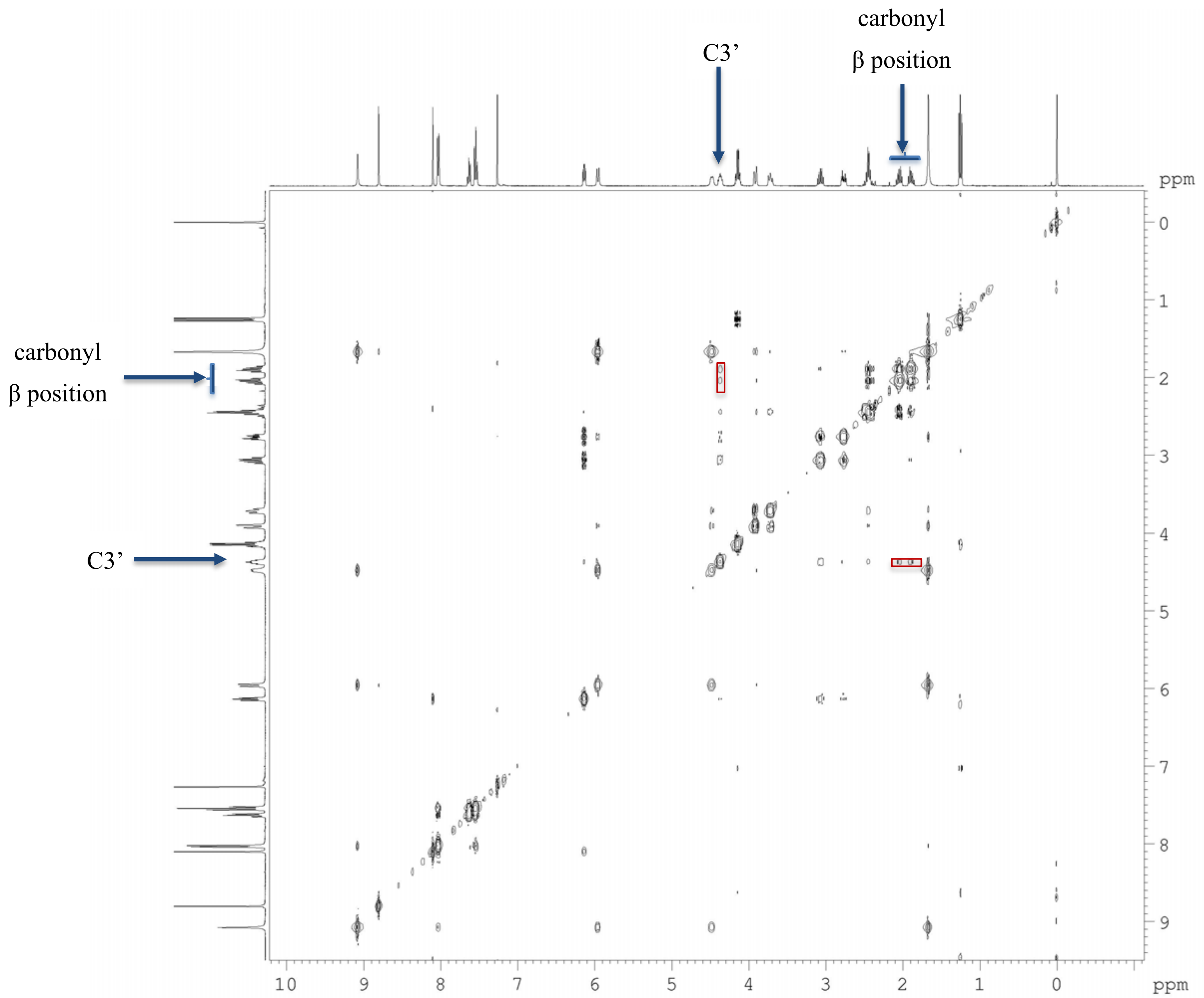

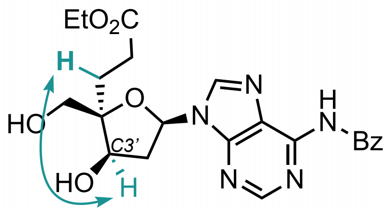

NOESY spectrum of **3fa**

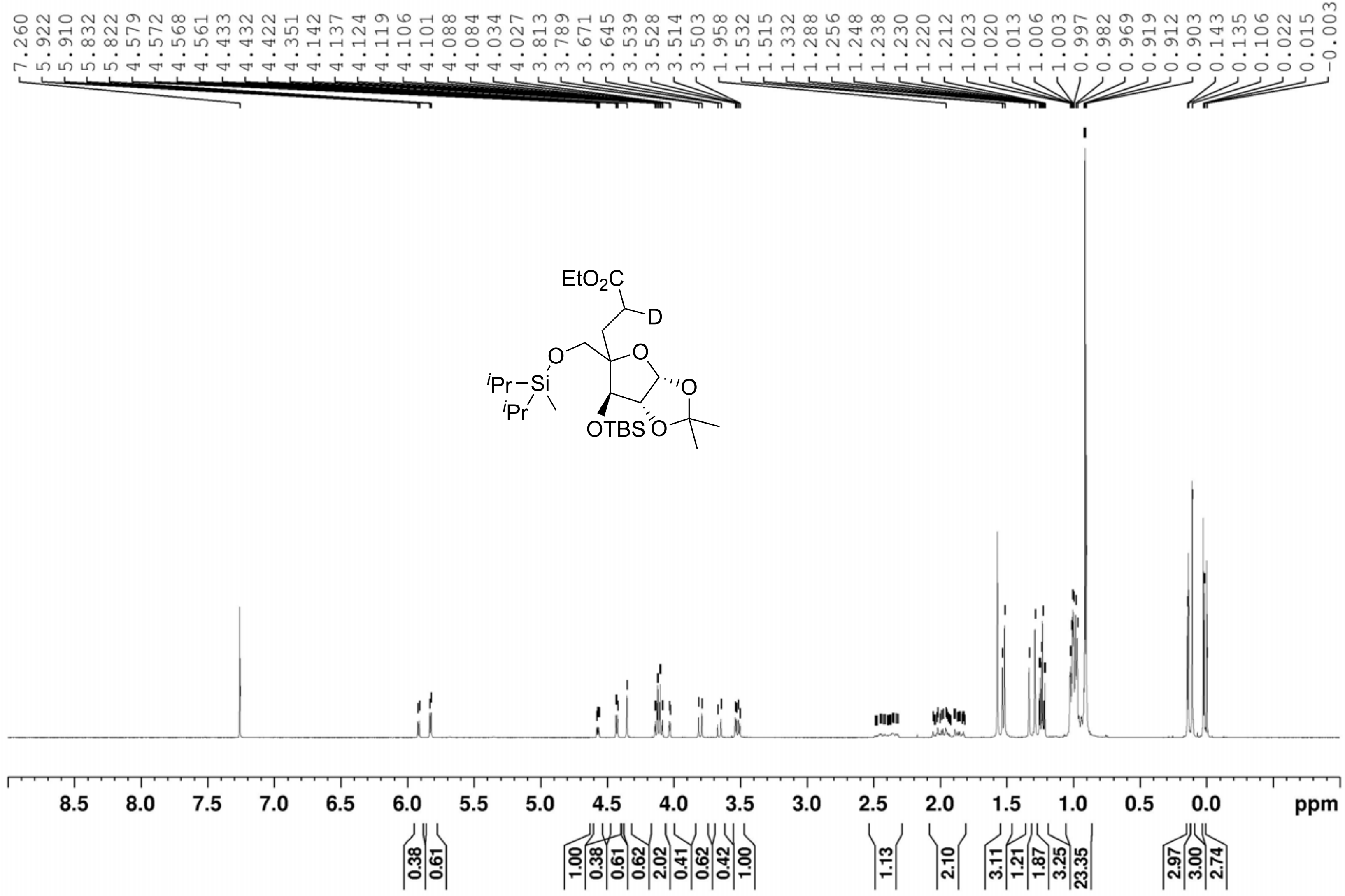

^{1}H NMR (400 MHz, $CDCl_3$) spectrum of **3ca-d**

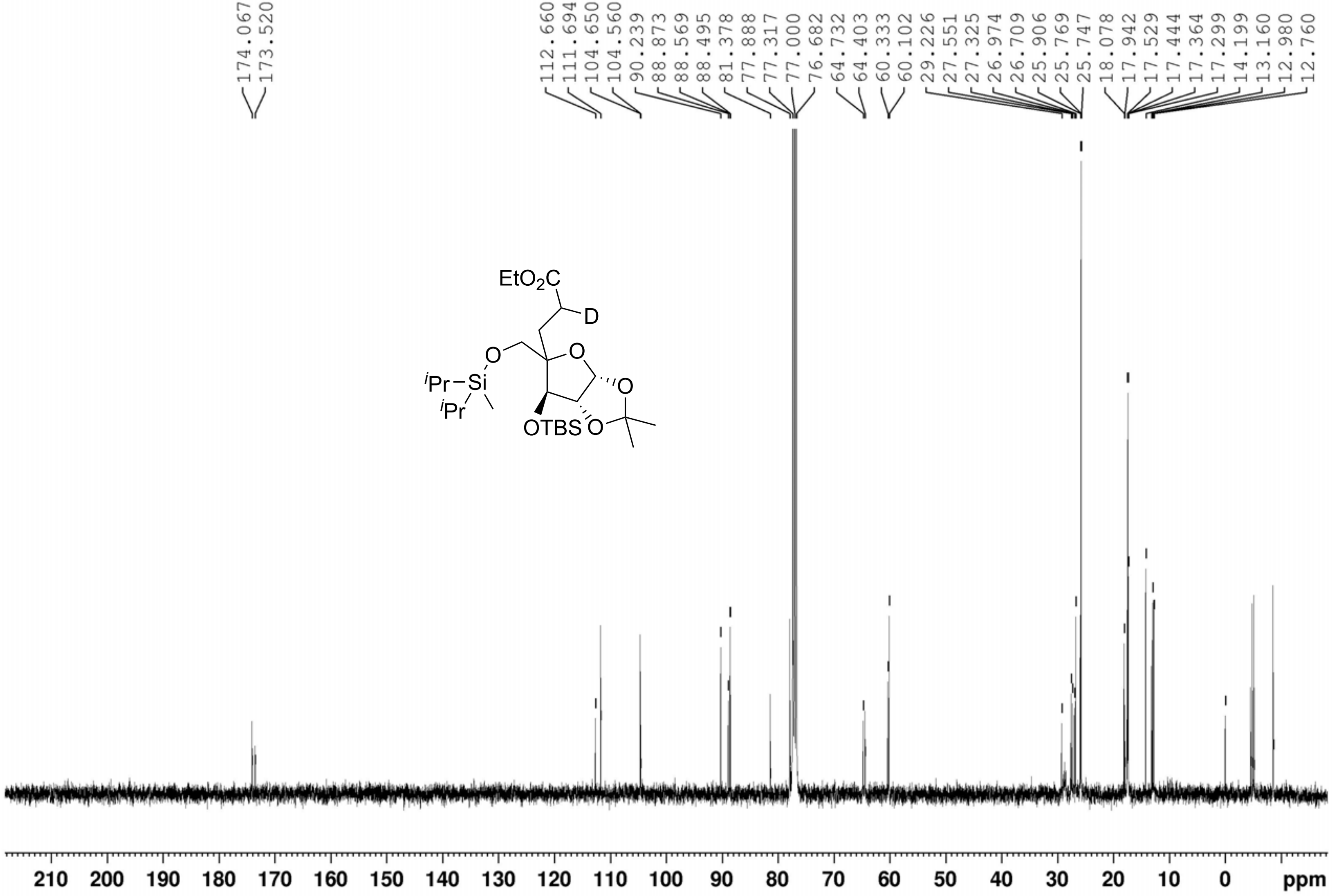

^{13}C NMR (100.6 MHz, $CDCl_3$) spectrum of **3ca-d**

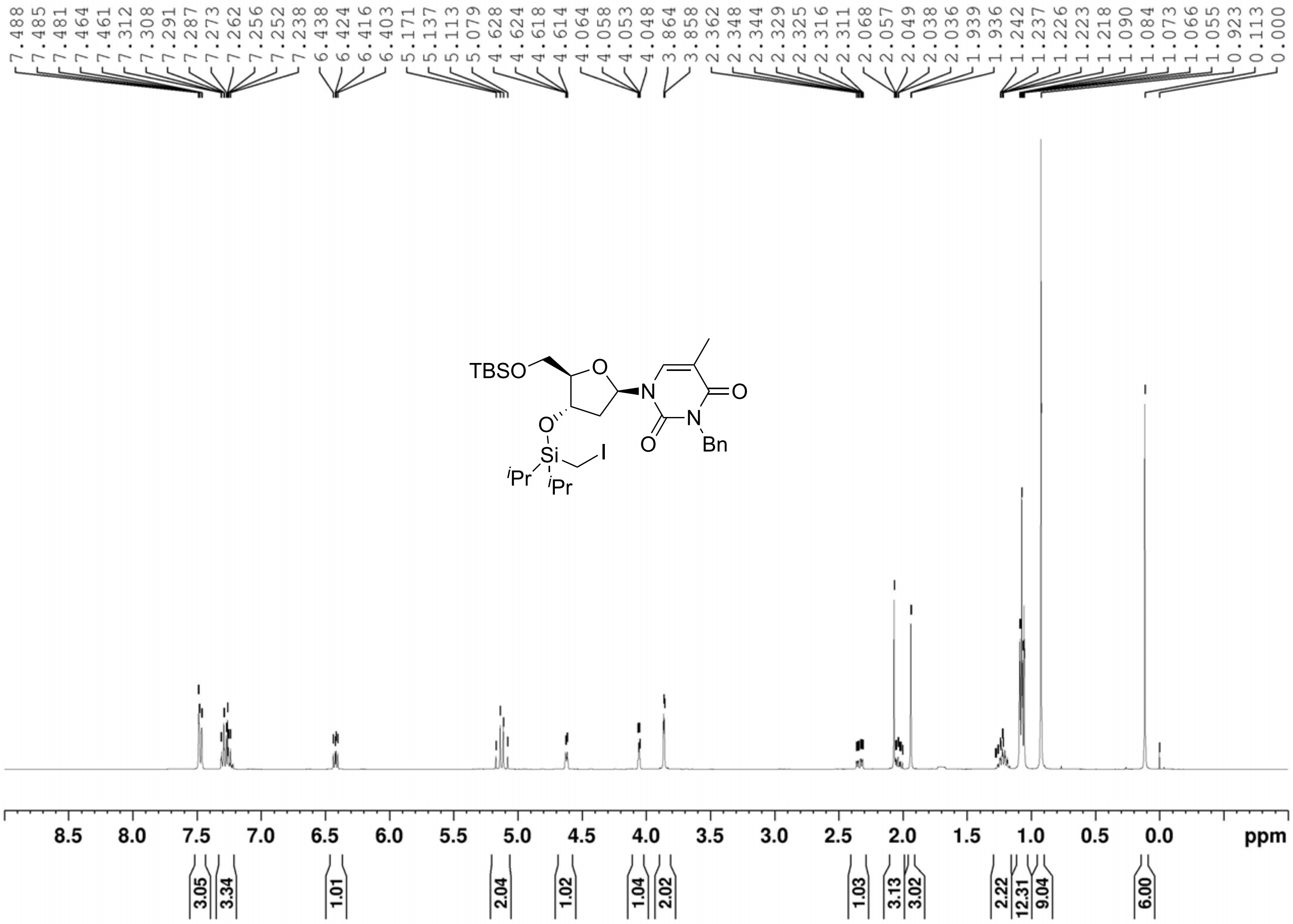

^{1}H NMR (400 MHz, $CDCl_3$) spectrum of **1g**

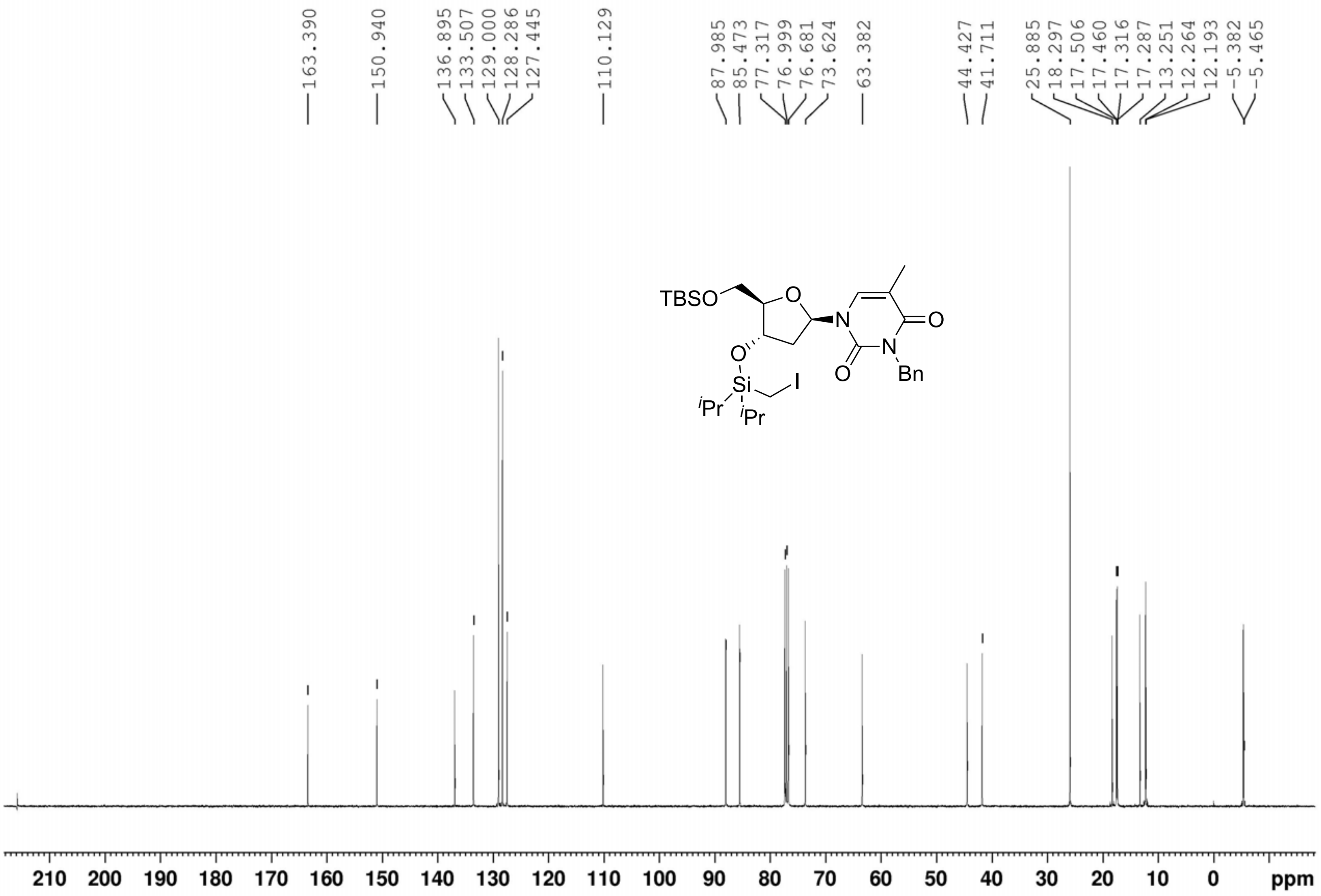

^{13}C NMR (100.6 MHz, $CDCl_3$) spectrum of **1g**

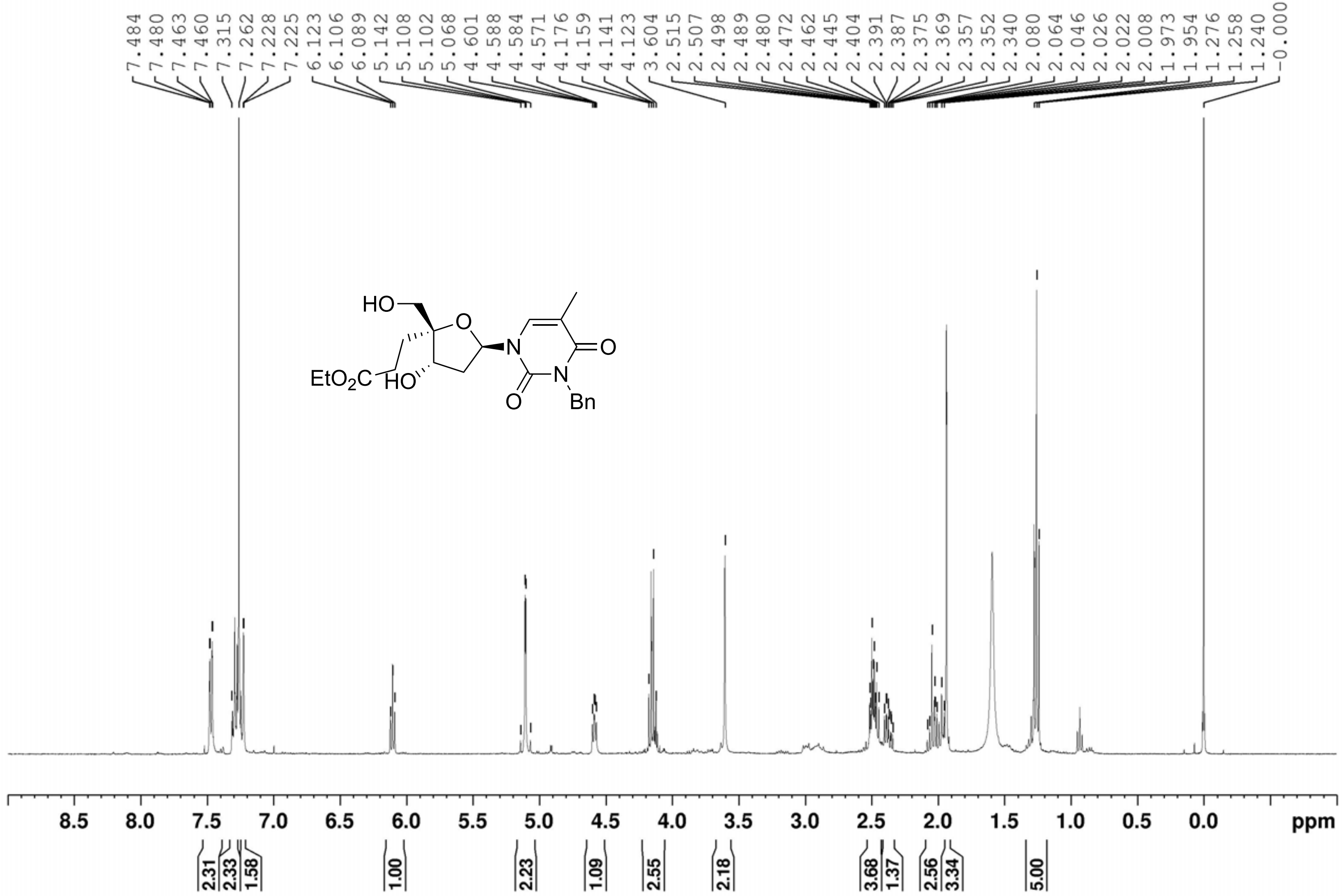

^{1}H NMR (400 MHz, $CDCl_3$) spectrum of **3ga**

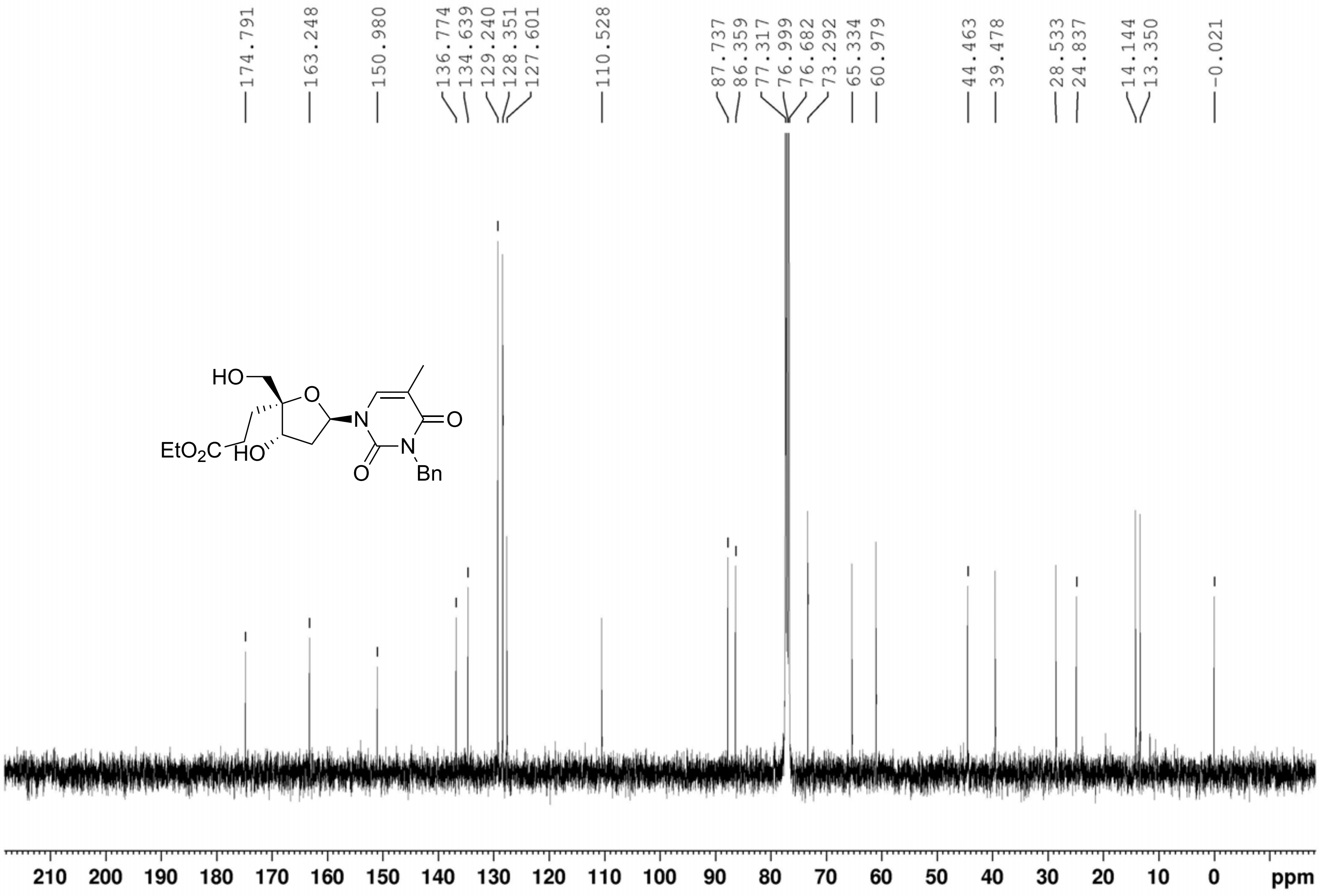

^{13}C NMR (100.6 MHz, $CDCl_3$) spectrum of **3ga**

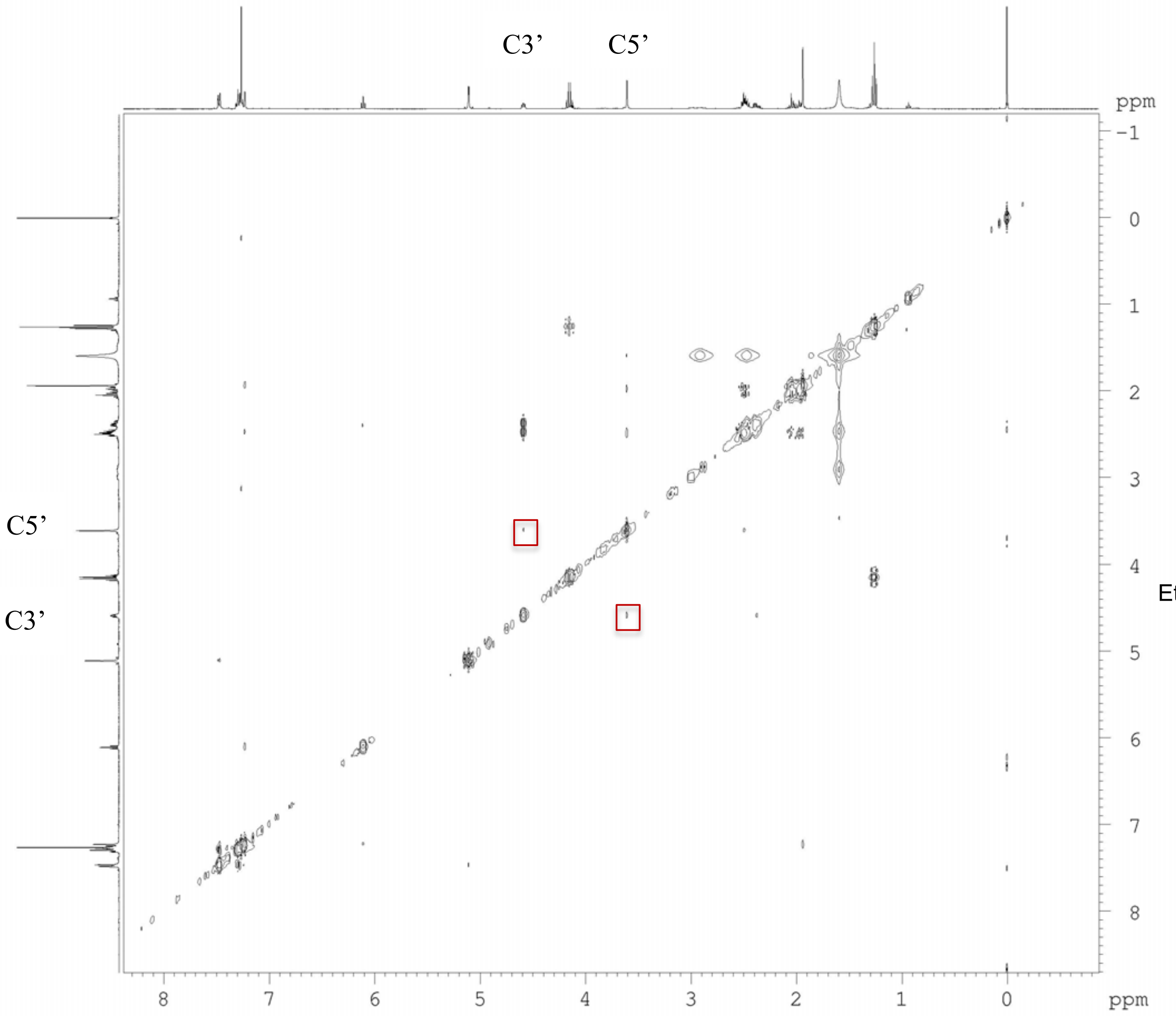

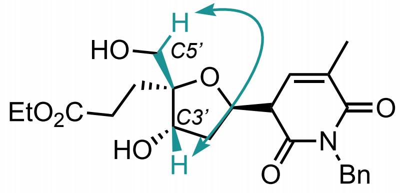

NOESY spectrum of **3ga**

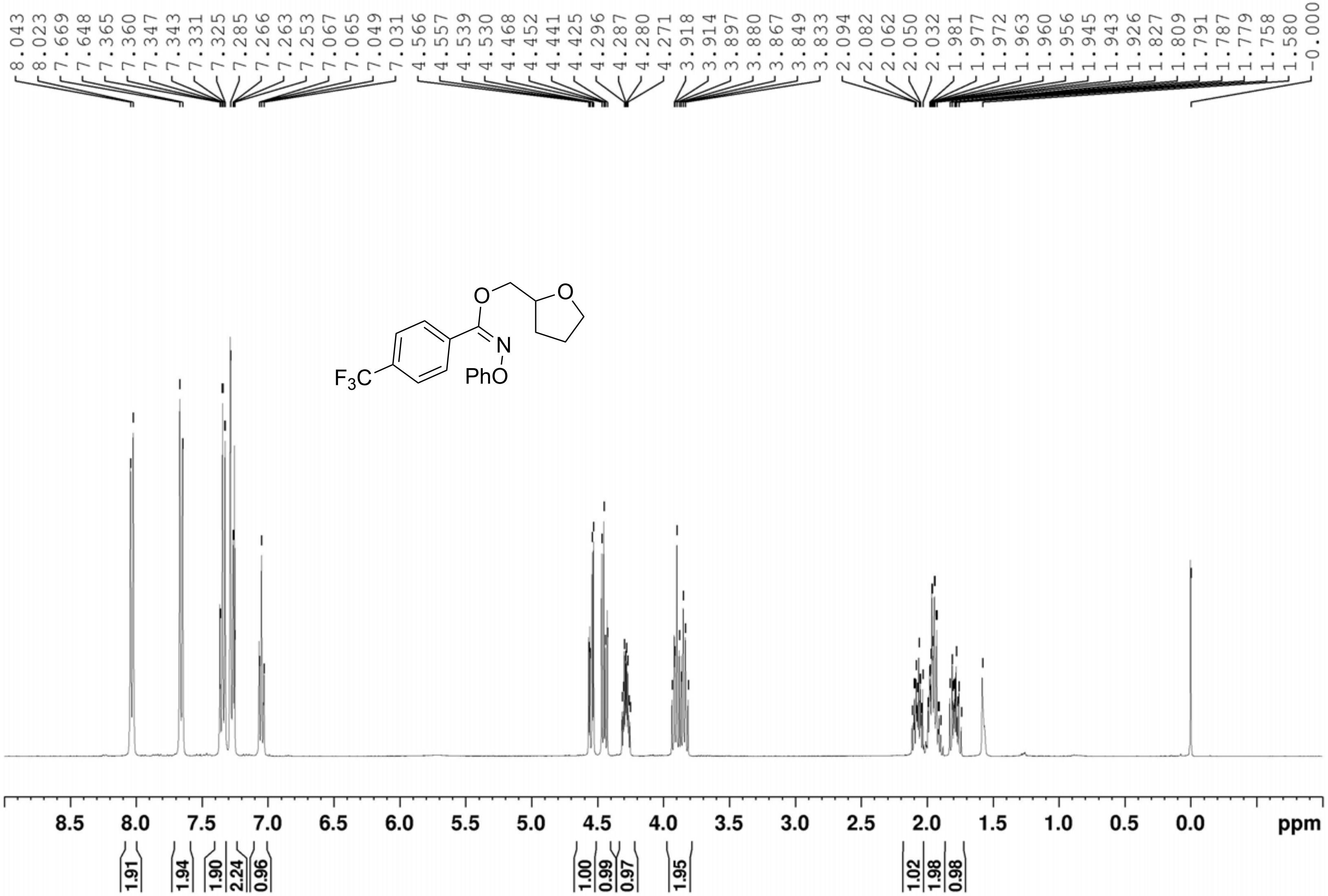

^{1}H NMR (400 MHz, $CDCl_3$) spectrum of **5S-1**

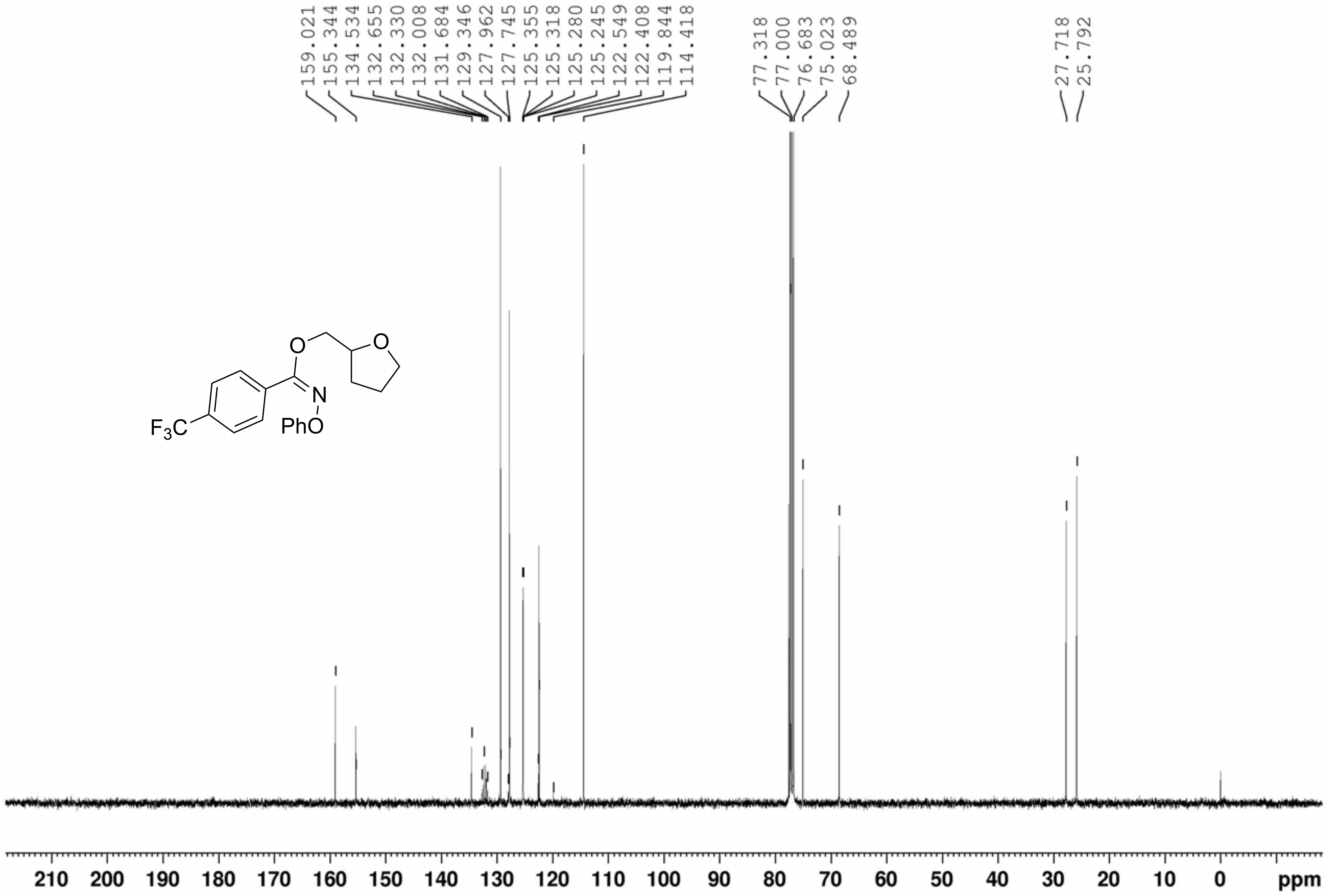

^{13}C NMR (100.6 MHz, $CDCl_3$) spectrum of **5S-1**

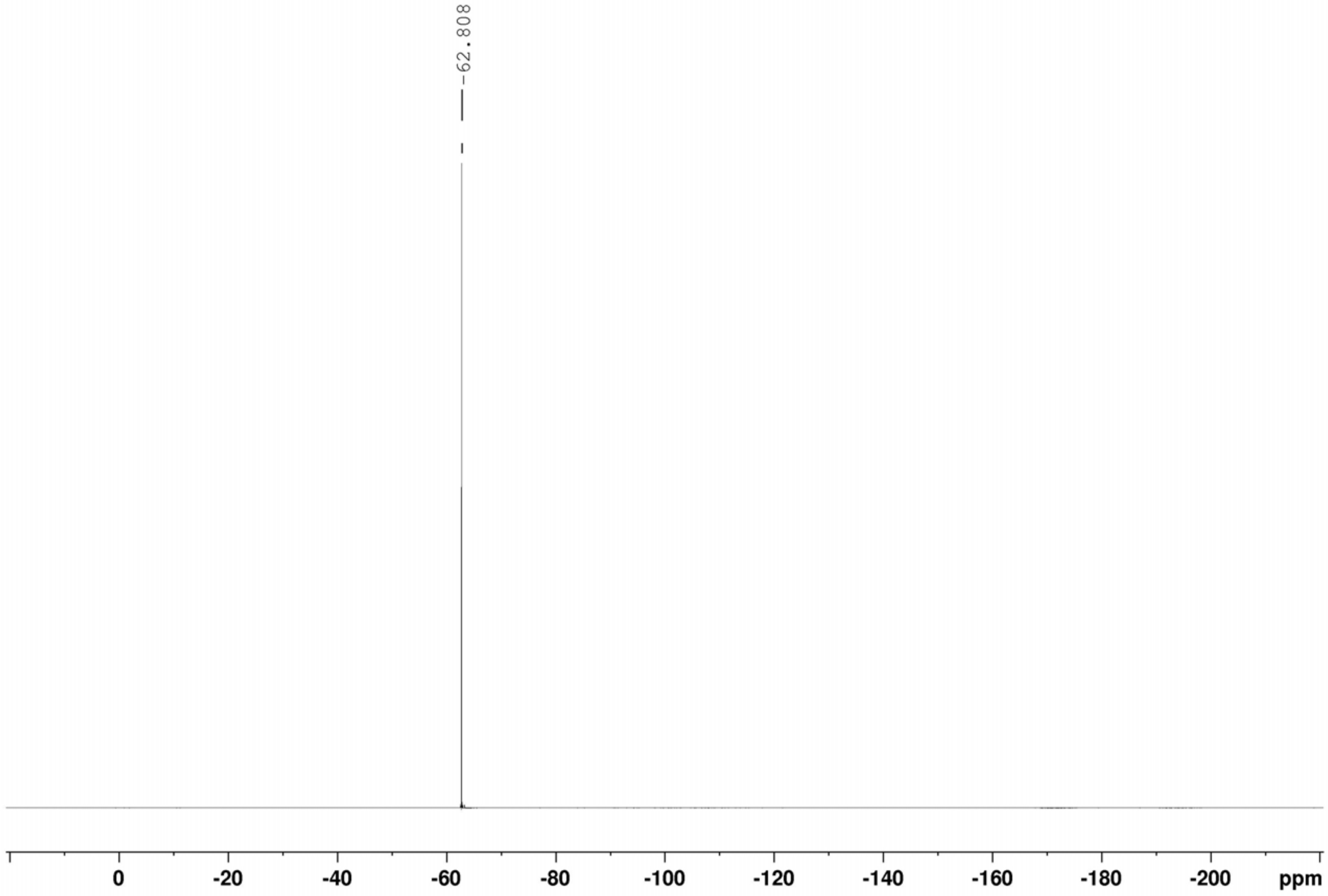

^{19}F NMR (376.5 MHz, $CDCl_3$) spectrum of **5S-1**

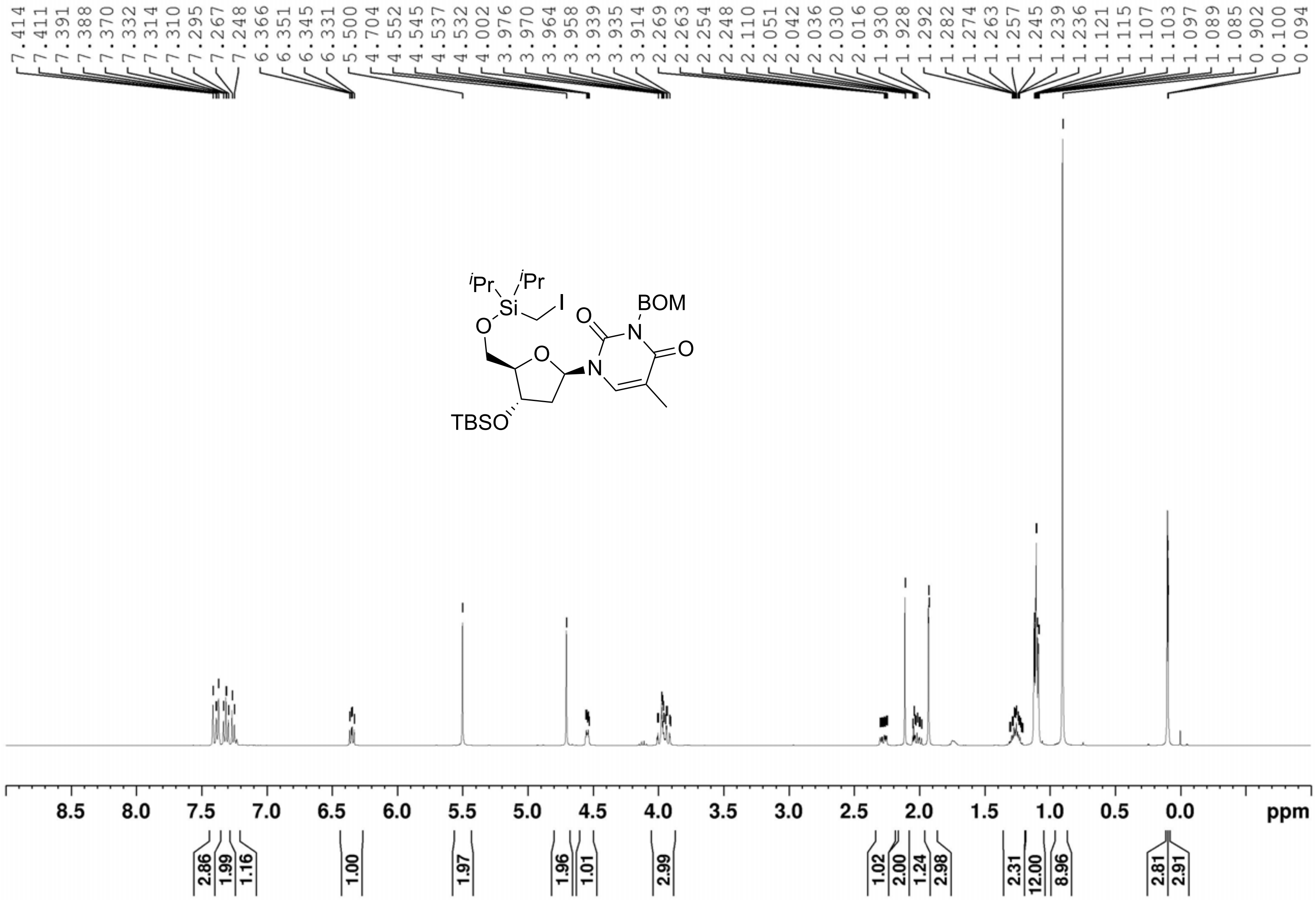

^1H NMR (400 MHz, $CDCl_3$) spectrum of **1h**

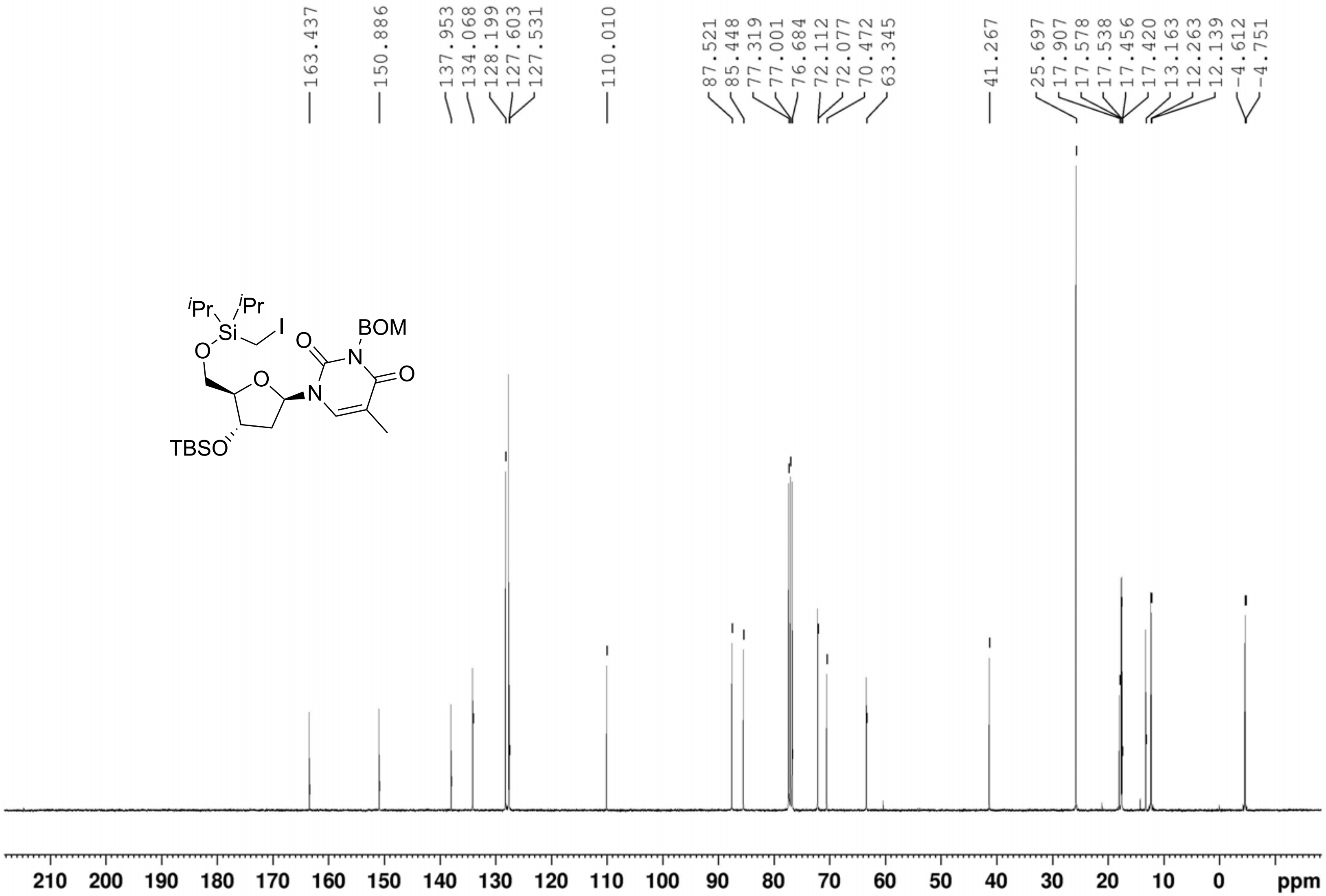

^{13}C NMR (100.6 MHz, $CDCl_3$) spectrum of **1h**

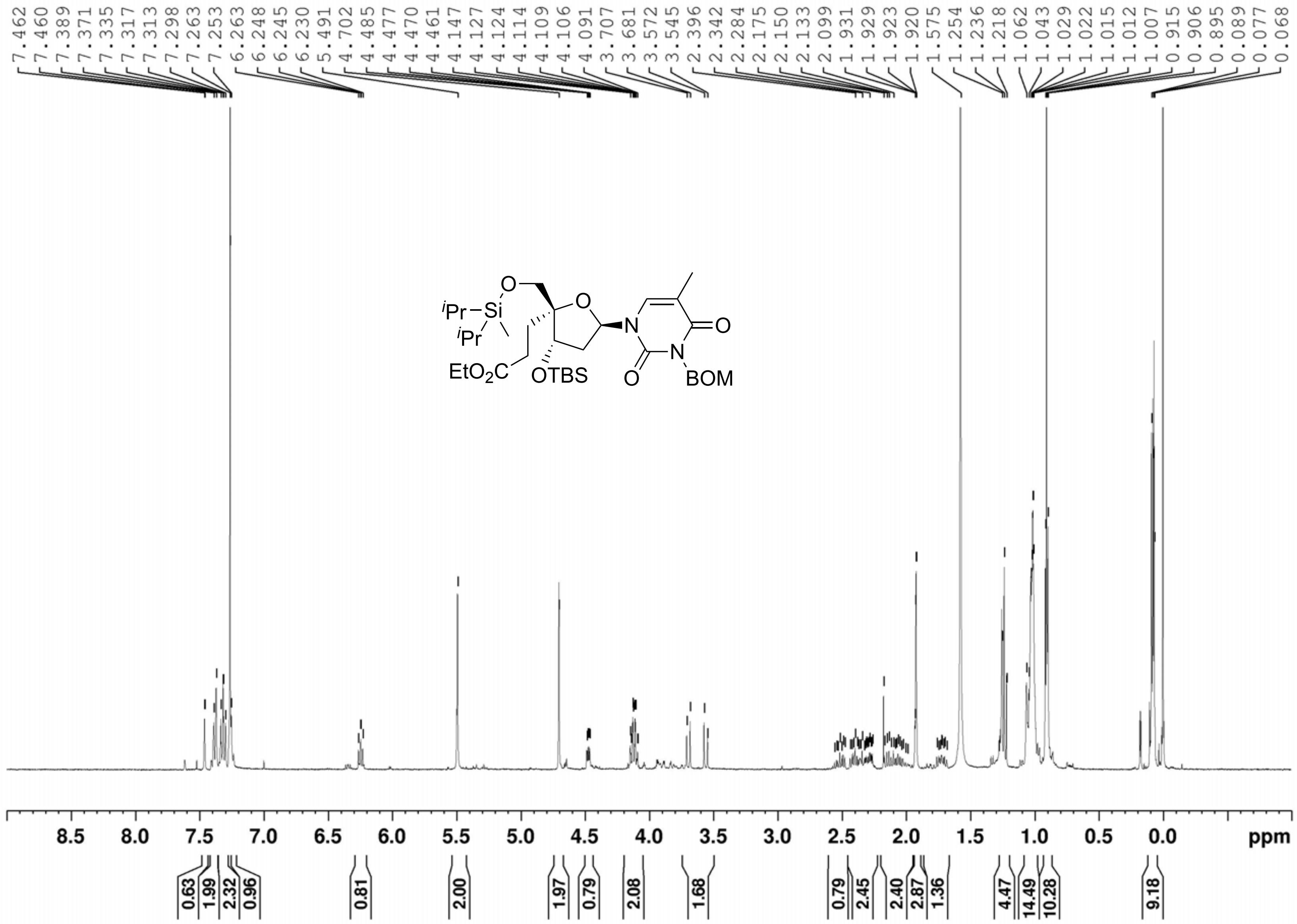

^{1}H NMR (400 MHz, $CDCl_3$) spectrum of **3ha**

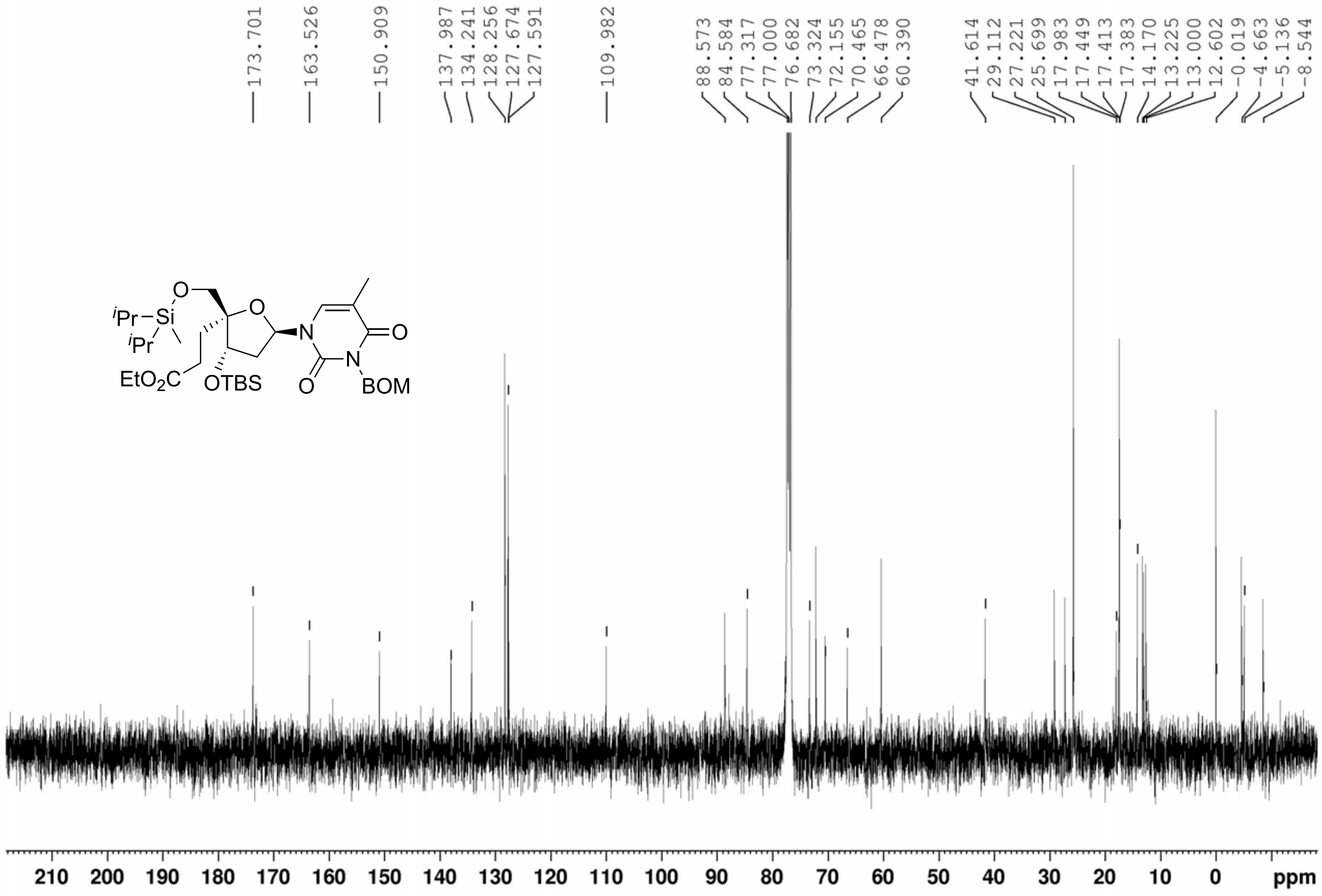

^{13}C NMR (100.6 MHz, $CDCl_3$) spectrum of **3ha**

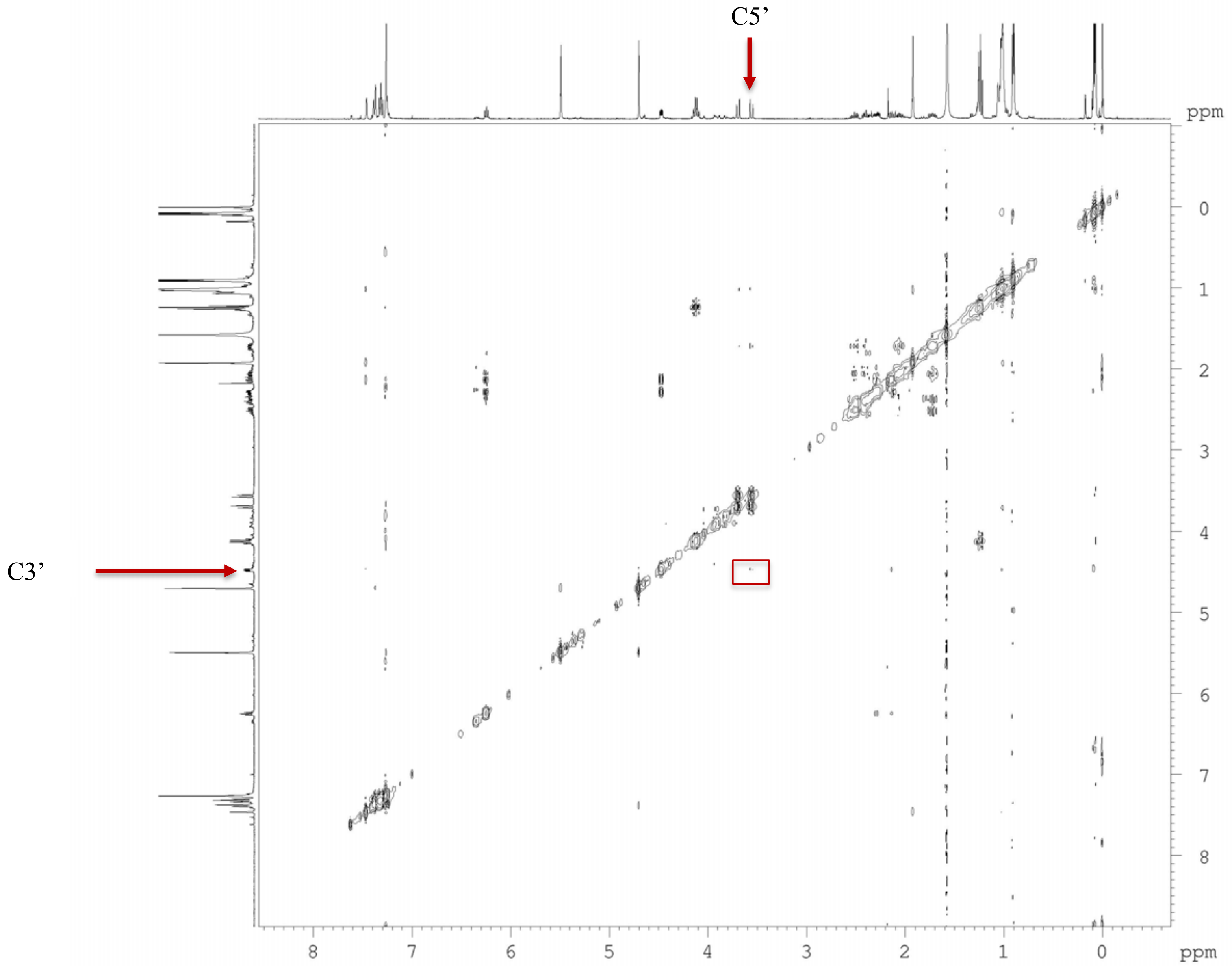

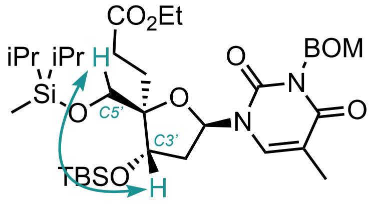

NOESY spectrum of **3ha**

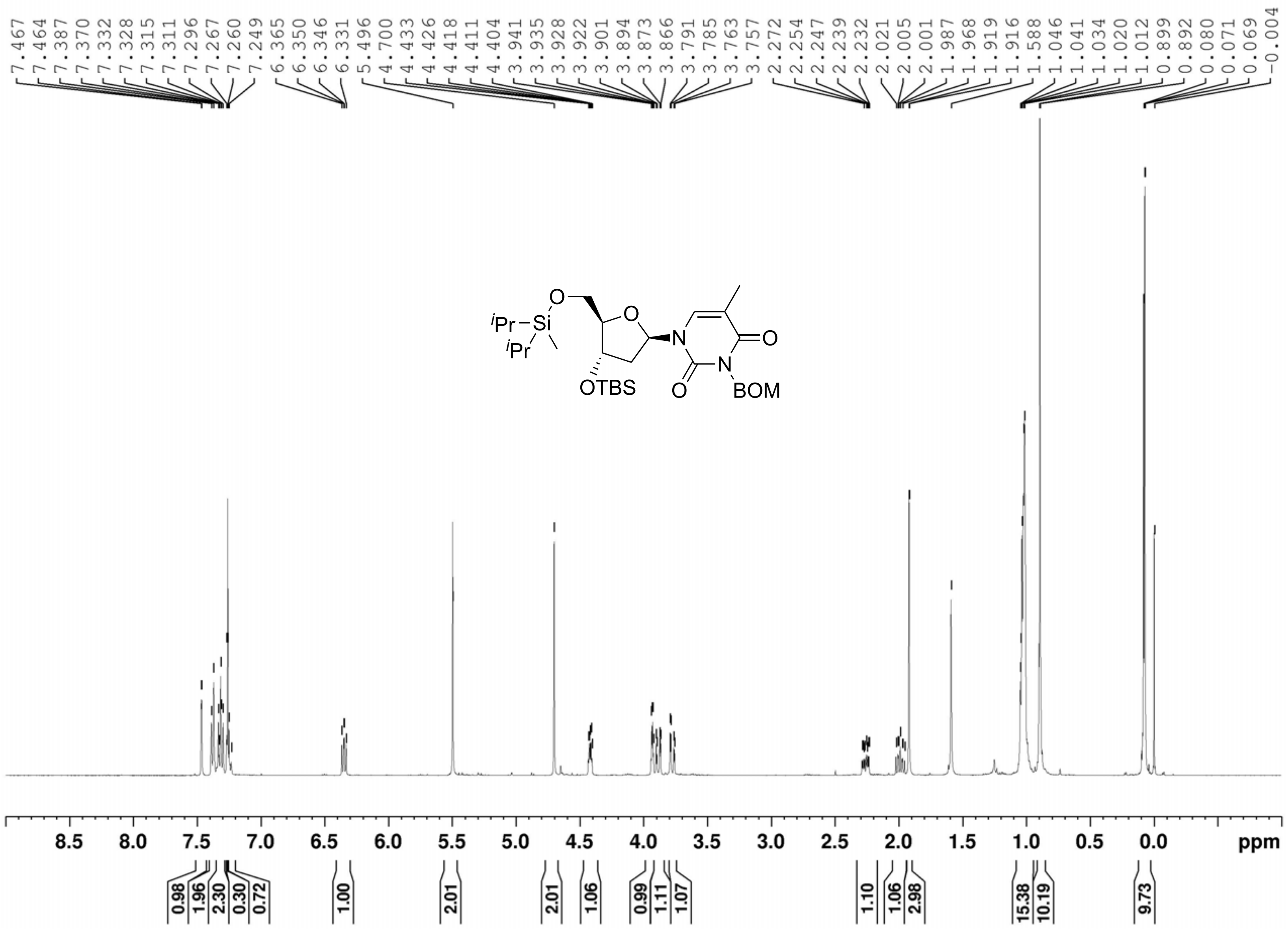

^{1}H NMR (400 MHz, $CDCl_3$) spectrum of **3h-1**

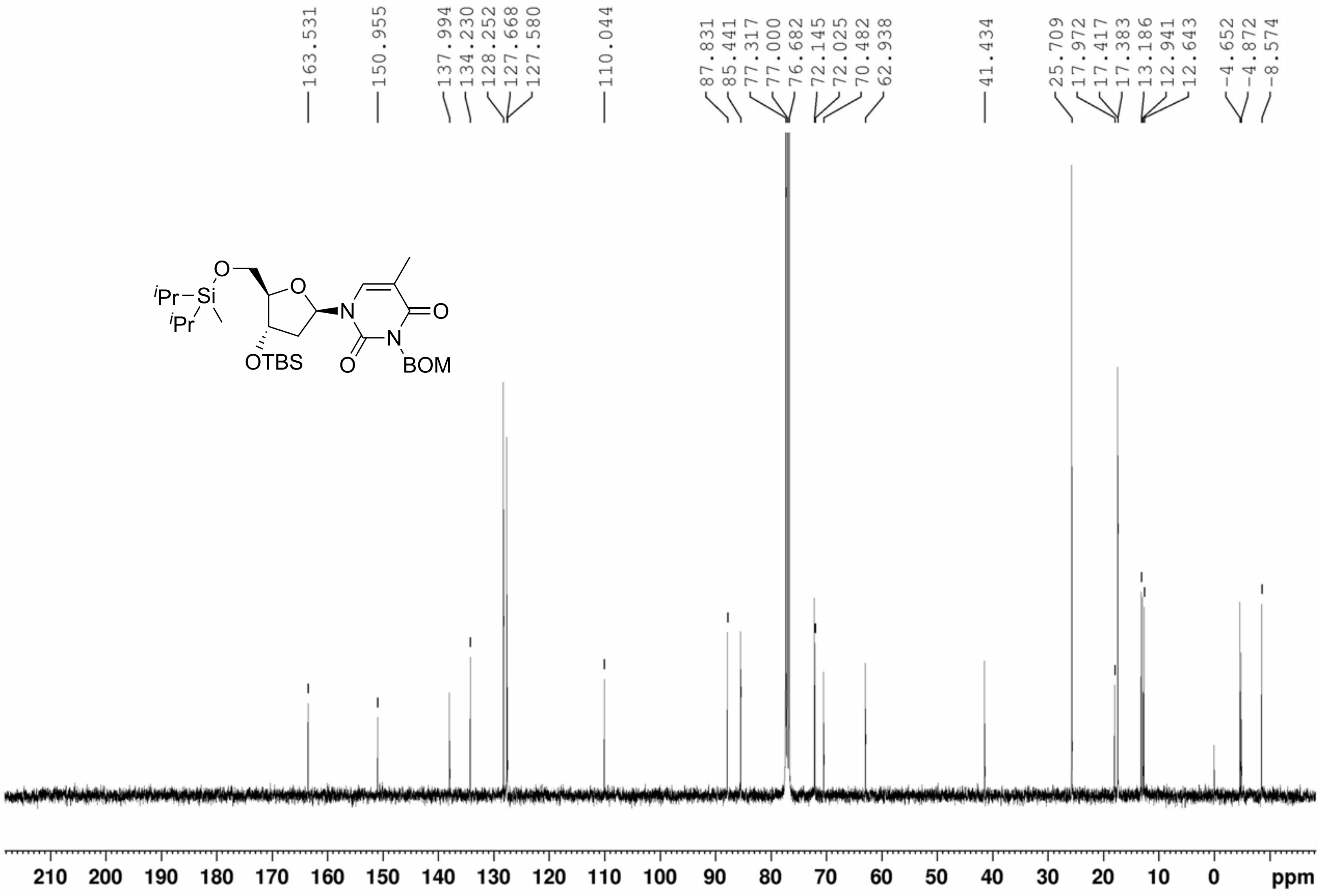

^{13}C NMR (100.6 MHz, $CDCl_3$) spectrum of **3h-1**

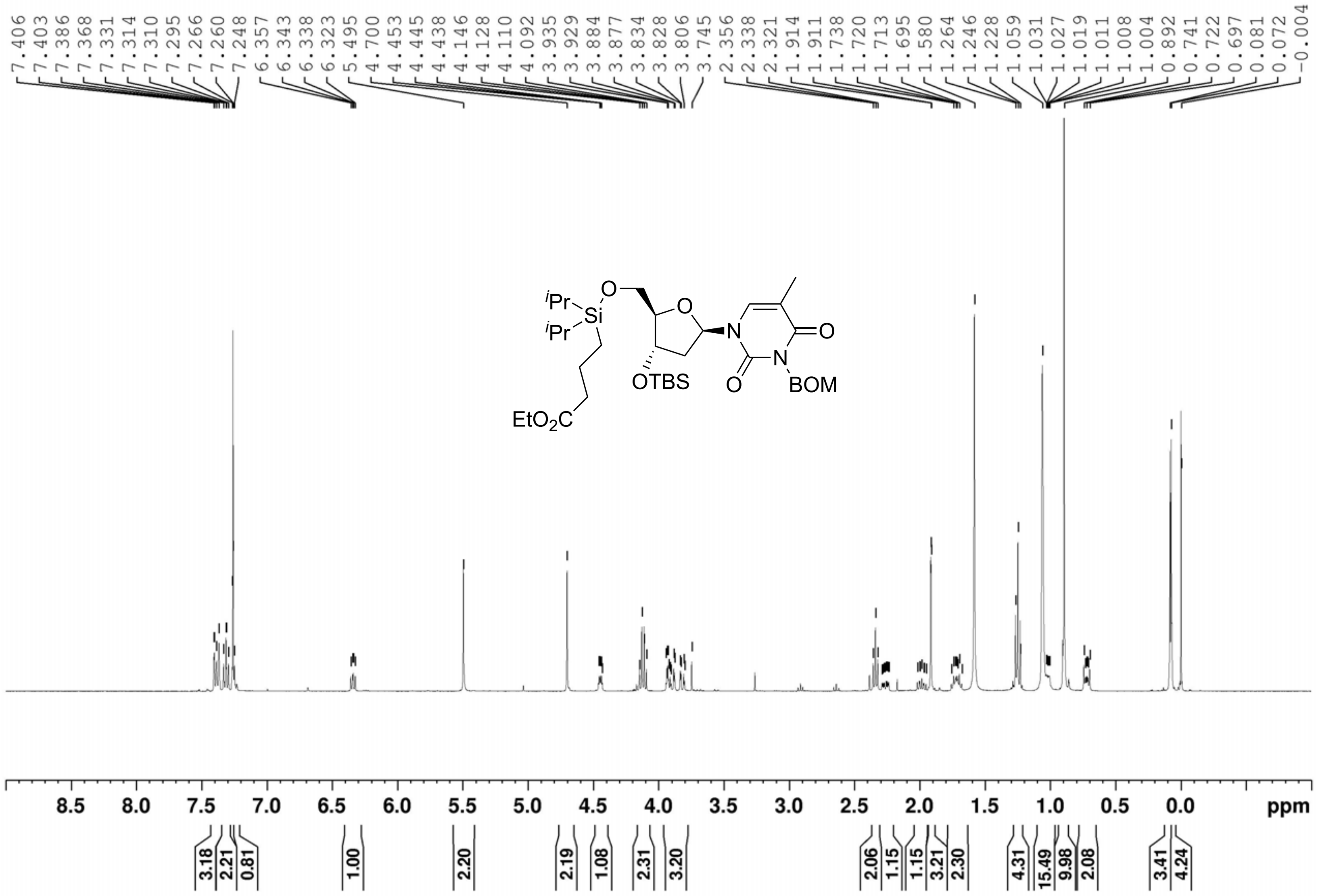

^{1}H NMR (400 MHz, $CDCl_3$) spectrum of **3ha-1**

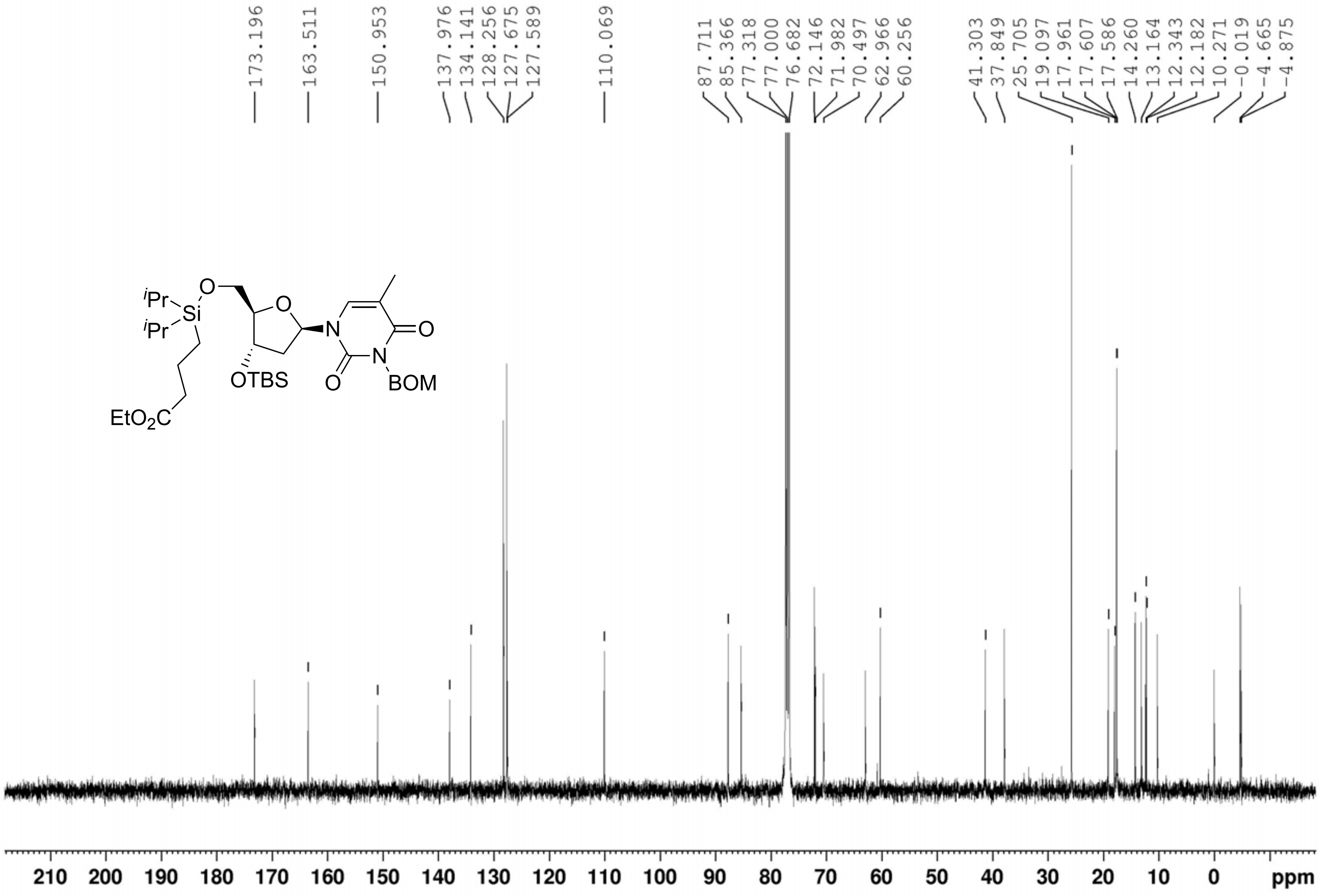

^{13}C NMR (100.6 MHz, $CDCl_3$) spectrum of **3ha-1**

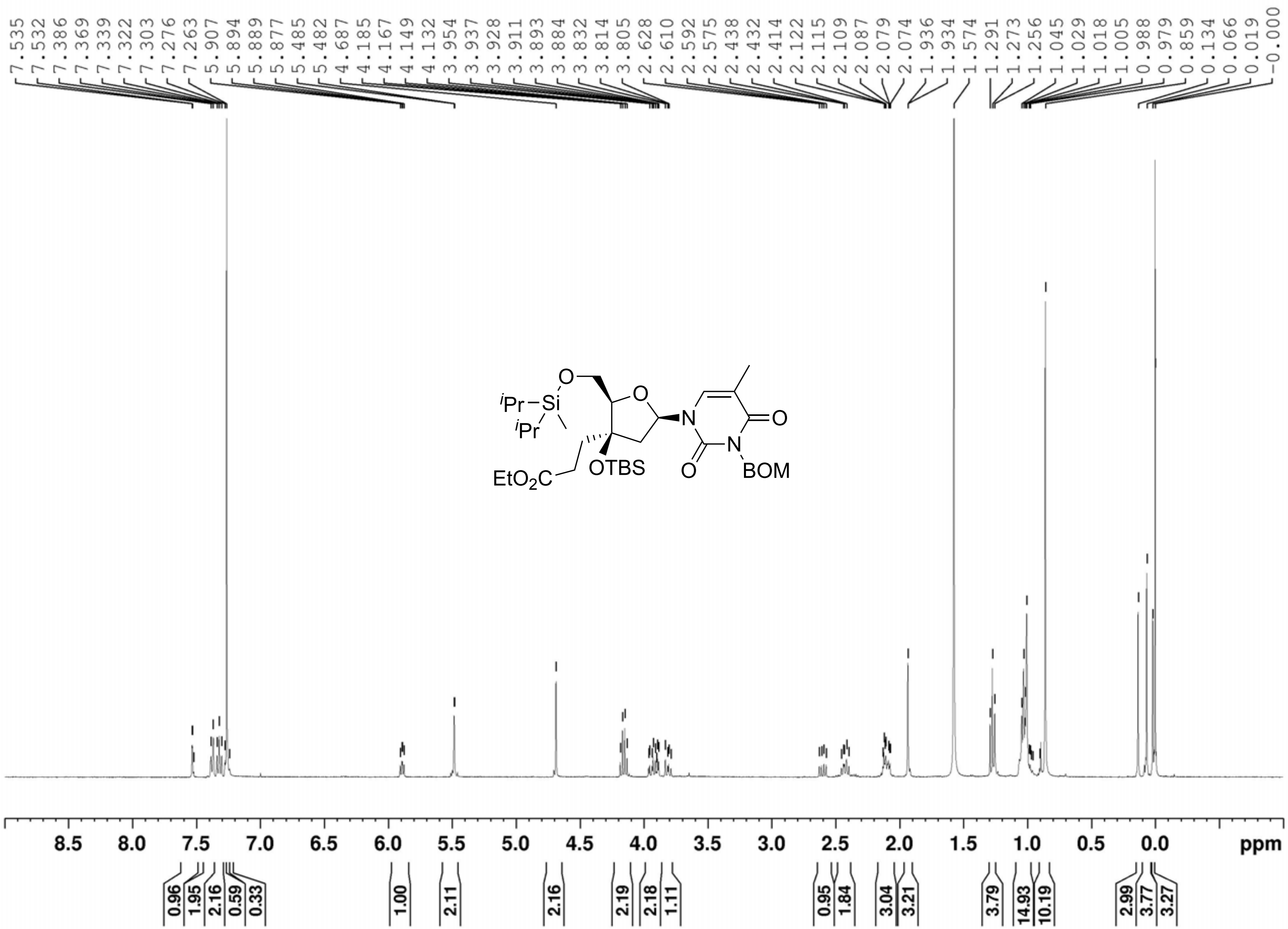

^{1}H NMR (400 MHz, $CDCl_3$) spectrum of **3ha-2**

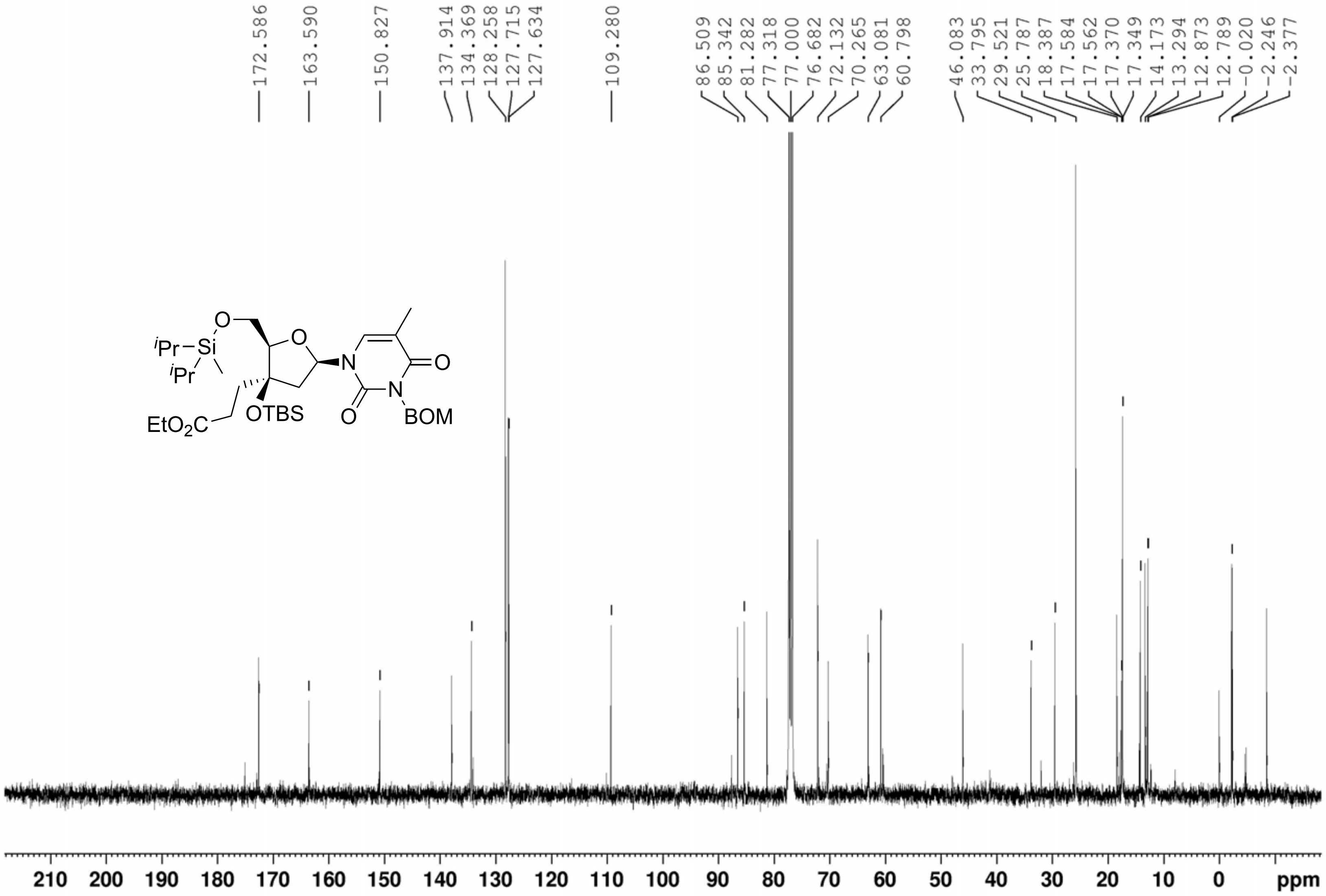

^{13}C NMR (100.6 MHz, $CDCl_3$) spectrum of **3ha-2**

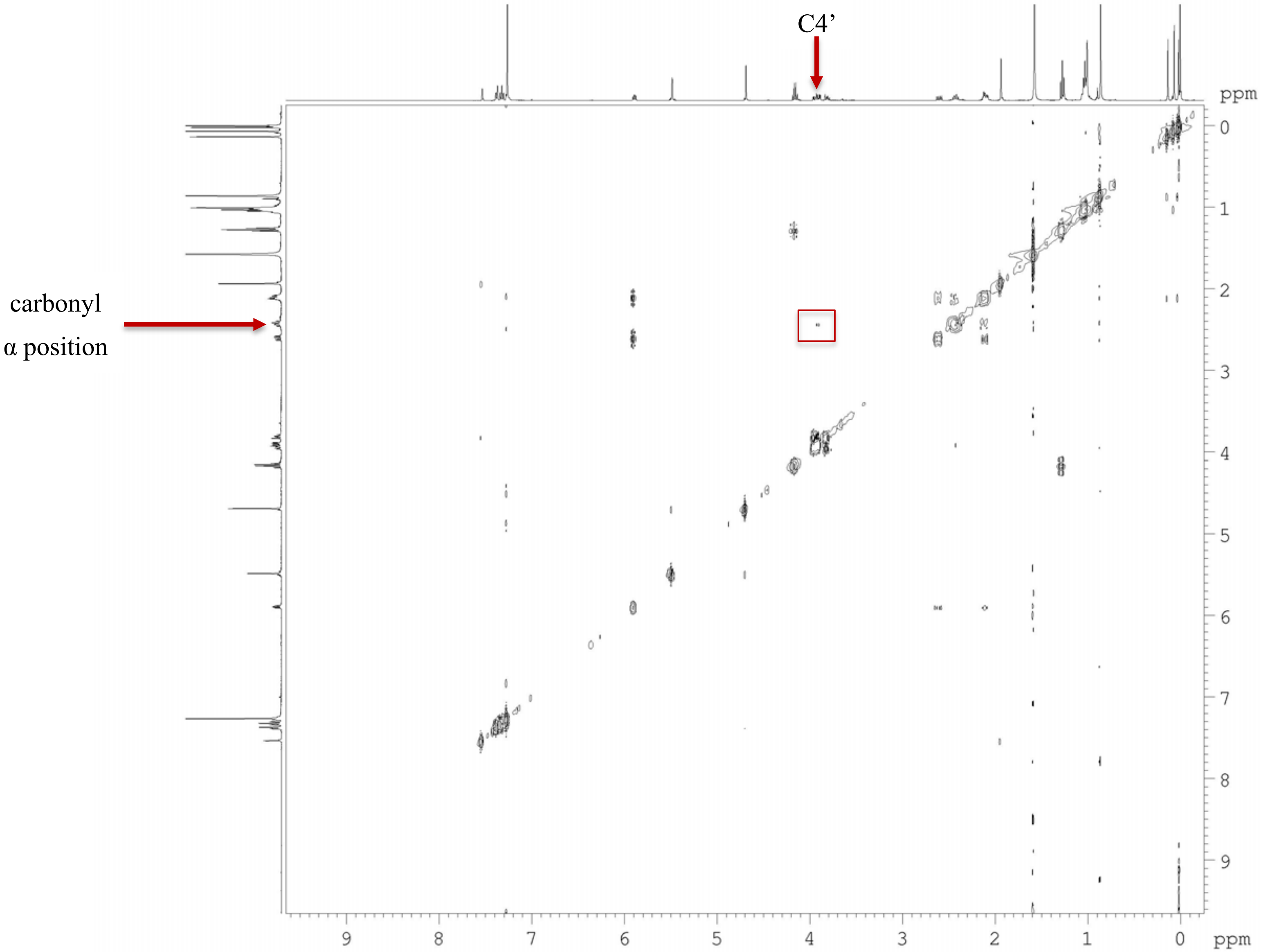

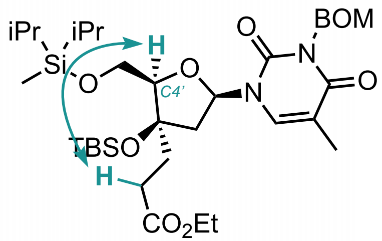

NOESY spectrum of **3ha-2**
